# Factors associated with university students’ knowledge about HIV and pre- and post-exposure prophylaxis

**DOI:** 10.1590/0034-7167-2024-0092

**Published:** 2024-08-30

**Authors:** Ana Luísa Serrano Lima, Heitor Hortensi Sesnik, Lucas Vinícius de Lima, Gabriel Pavinati, Maria de Fátima Garcia Lopes Merino, Marcelle Paiano, Nelly Lopes de Moraes Gil, Gabriela Tavares Magnabosco

**Affiliations:** I Universidade Estadual de Maringá. Maringá, Paraná, Brazil

**Keywords:** HIV, Knowledge, Pre-Exposure Prophylaxis, Post-Exposure Prophylaxis, Students, VIH, Conocimiento, Profilaxis Pre-Exposición, Profilaxis Posexposición, Estudiantes

## Abstract

**Objectives:**

to analyze the factors associated with university students’ knowledge about HIV and pre- and post-exposure prophylaxis.

**Methods:**

a cross-sectional study was conducted with 503 university students from a southern state in Brazil; data were collected using a characterization tool and a questionnaire containing 16 statements about the topic; descriptive measures and Poisson regression models with robust variance were used for analysis.

**Results:**

the prevalence of adequate knowledge (i.e., scoring more than 12 correct answers) was 27.83%; students older than 24 years, enrolled in health-related courses, who had not engaged in sexual relations in the last quarter, with a history of rapid HIV testing, and who knew or had heard about the prophylaxes showed a higher likelihood of scoring more than 12 correct answers.

**Conclusions:**

generally, the knowledge of young people about HIV and its prophylaxes was found to be inadequate and influenced by sociodemographic, educational, and behavioral factors.

## RESUMEN

**Objetivos:**

analizar los factores asociados con el conocimiento de los estudiantes universitarios sobre el VIH y la profilaxis pre y post exposición.

**Métodos:**

se realizó un estudio transversal con 503 estudiantes universitarios de un estado del sur de Brasil; los datos se recopilaron utilizando una herramienta de caracterización y un cuestionario que contenía 16 declaraciones sobre el tema; se utilizaron medidas descriptivas y modelos de regresión de Poisson con varianza robusta para el análisis.

**Resultados:**

la prevalencia de conocimiento adecuado (es decir, obtener más de 12 respuestas correctas) fue del 27.83%; los estudiantes mayores de 24 años, matriculados en cursos relacionados con la salud, que no habían tenido relaciones sexuales en el último trimestre, con antecedentes de pruebas rápidas de VIH y que conocían o habían oído hablar sobre las profilaxis mostraron una mayor probabilidad de obtener más de 12 respuestas correctas.

**Conclusiones:**

en general, se encontró que el conocimiento de los jóvenes sobre el VIH y sus profilaxis era inadecuado e influenciado por factores sociodemográficos, educativos y conductuales.

## INTRODUCTION

The infection caused by the human immunodeficiency virus (HIV) constitutes a severe public health issue with social, economic, and epidemiological impacts on individuals and communities around the globe. The Pan American Health Organization (PAHO) estimated that there were 38 million people living with HIV globally, and in 2019, there was a 21% increase in incidence in the Latin American region compared to 2010 data^(1)^.

In Brazil, in 2021, more than 40,000 new HIV diagnoses were reported; of these, 39% occurred in individuals between 20 and 29 years old^(2)^. Moreover, the infection disproportionately affected men, with a ratio of 2.8 new HIV cases among men for every woman^(2)^. This indicates the presence of groups more vulnerable to infection in the country, which should therefore be prioritized in public policies.

Factors such as early sexual initiation, engagement in unprotected sexual practices with multiple partners, and low risk perception among this demographic increase susceptibility to HIV infection^(3-4)^. Furthermore, when infected, young people with HIV often find themselves in dynamic and subjective contexts of vulnerability, facing situations of stigma and discrimination, denial of diagnosis, and low adherence to antiretroviral therapy (ART)^(5)^.

Over the years, global advancements have been observed in the policies for the prevention, diagnosis, and treatment of HIV. In 2010, the Joint United Nations Programme on HIV/AIDS (UNAIDS) suggested that strategies should be based on combined HIV prevention, defined as a set of biomedical, behavioral, and structural interventions aimed at reducing new infections and tailored to meet the needs of individuals and communities^(6)^.

In 2017, Brazil’s Ministry of Health proposed guidelines for implementing combined prevention within the Unified Health System (*Sistema Único de Saúde*, SUS), emphasizing priority groups and key populations, notably gay men, men who have sex with men (MSM), and young people^(7)^. This initiative aimed to strengthen the response to HIV to achieve the elimination goals by 2030, as established in the Sustainable Development Goals (SDGs).

Within the framework of combined HIV prevention, pre-exposure prophylaxis (PrEP) and post-exposure prophylaxis (PEP)-classified as biomedical interventions-emerge as potential tools for infection control, especially when integrated with behavioral and structural actions^(7)^. PrEP and PEP are provided for free through the SUS and involve, respectively, the use of antiretrovirals before and after exposure to HIV as a form of prevention^(7)^.

For prophylaxes to be effectively and assertively implemented, users must be informed about the strategies to choose those that best suit their specific needs and circumstances^(8)^. However, recent research indicates a lack of dissemination and recommendation of PrEP and PEP, suggesting that young people’s knowledge about these prophylaxes is sometimes fragile and erroneous, which hinders the expansion of these methods’ usage^(9-11)^.

In this context, it is recognized that schools and universities play a crucial role in disseminating information about sexually transmitted infections (STIs) and their preventive measures among students^(9-10)^. Through educational programs, awareness campaigns, and student health services, these institutions can support knowledge about the prevention, detection, and treatment of STIs, thus promoting health within the student community.

Therefore, the necessity of identifying the knowledge held by students is undeniable, as the adoption (or non-adoption) of preventive practices depends on it. In this regard, this research is anticipated to contribute to the implementation of targeted action plans, aimed at enhancing knowledge on the subject, encouraging the adoption of practices aligned with combined prevention, and consequently, reducing the spread of HIV among this group.

## OBJECTIVES

To analyze the factors associated with university students’ knowledge about HIV and pre- and post-exposure prophylaxis.

## METHODS

### Ethical aspects

Approval was obtained from the Research Ethics Committee of the *Universidade Estadual de Maringá* (UEM). The ethical guidelines of Resolutions No. 466/2012 and No. 674/2022 of the National Health Council, as well as Circular Letter No. 002/2021, were adhered to. Informed consent was electronically secured from all participants, who selected the option: “I declare that I have been informed and agree to voluntarily participate in this research”.

### Study design, period, and location

This is a descriptive and analytical cross-sectional study^(12)^, guided by the recommendations of the Strengthening the Reporting of Observational Studies in Epidemiology (STROBE) checklist. The research was conducted from September to October 2023 among students at UEM, located in the northwest region of Paraná state, Brazil. According to the 2022 census data, the city of Maringá has a population of 409,657^(13)^.

UEM is a state public university with a presence throughout Paraná, supported by activities related to teaching, research, and extension projects developed at the main campus in Maringá and six regional campuses. With approximately 15,000 students, UEM offers over 70 undergraduate programs across various campuses, spanning diverse fields such as agricultural, biological, exact sciences, humanities, technological, health, and social sciences^(14)^.

### Population or sample; inclusion and exclusion criteria

The study population consisted of all students enrolled in on-campus undergraduate courses at the UEM. The inclusion criteria specified that participants must be aged 18 years or older and properly enrolled in the 2023 academic year. The sole exclusion criterion was failure to fully complete the data collection questionnaire. In total, the study population included 15,199 students, of whom 14,672 met the inclusion criteria.

Given the eligible population for this study, the sample size was determined by setting (i) a margin of error at 5%, (ii) a confidence level at 95%, and (iii) accounting for the heterogeneous distribution of the population, resulting in a sample of 375 participants. To accommodate potential losses and errors in the data collection process, an additional 10% was added to define the minimum number of participants, totaling 413 students.

### Study protocol

A structured, self-administered questionnaire was employed, designed by the authors based on similar studies^(11,15-19)^, containing statements to assess knowledge about HIV, PrEP, and PEP. Each question offered three response options: agree, disagree, or don’t know (Chart 1). In addition, a characterization questionnaire was administered with the following variables of interest^(11,15-19)^, each including an open field for exceptions:

**Chart 1:** Structured and self-administered questionnaire for data collection on knowledge about HIV infection and pre- and post-exposure prophylaxis, Maringá, Paraná, Brazil, 2023

Variable		Category
HIV	Q1: The only way to get infected with HIV is through unprotected sexual relations.	Agree; disagree^*^; don’t know
	Q2: The use of a condom is the only measure capable of preventing HIV infection.	Agree; disagree^*^; don’t know
	Q3: HIV infection depends on the sex and/or sexual orientation of the person.	Agree; disagree^*^; don’t know
	Q4: A person with HIV stops transmitting the virus with the correct use of oral antiretroviral medications.	Agree^*^; disagree; don’t know
PrEP	Q5: PrEP consists of the use of oral antiretrovirals before exposure to the virus, as a prevention strategy to reduce the risk of infection.	Agree^*^; disagree; don’t know
	Q6: I can take PrEP at any time before having sexual intercourse.	Agree; disagree^*^; don’t know
	Q7: PrEP is available to the entire sexually active population over 15 years old.	Agree^*^; disagree; don’t know
	Q8: A person using PrEP is also protected from other sexually transmitted infections, in addition to HIV.	Agree; disagree^*^; don’t know
	Q9: PrEP is offered for free in the Unified Health System.	Agree^*^; disagree; don’t know
	Q10: Considering that a person using PrEP is not sick, they do not need monitoring by health professionals.	Agree; disagree^*^; don’t know
PEP	Q11: PEP consists of the use of oral antiretrovirals after exposure to the virus, as a prevention strategy to reduce the risk of infection.	Agree^*^; disagree; don’t know
	Q12: PEP is indicated for situations such as accidents with sharp objects, sexual violence, etc., or on demand after perceiving a risk.	Agree^*^; disagree; don’t know
	Q13: PEP must be taken for 28 days uninterrupted.	Agree^*^; disagree; don’t know
	Q14: If a condom breaks during my sexual intercourse, I can request the use of PEP from the Unified Health System.	Agree^*^; disagree; don’t know
	Q15: The sooner PEP is started, the greater the chances of preventing HIV infection.	Agree^*^; disagree; don’t know
	Q16: The maximum timeframe to start PEP is 72 hours after exposure to HIV.	Agree^*^; disagree; don’t know

HIV - human immunodeficiency virus; PrEP - pre-exposure prophylaxis; PEP - post-exposure prophylaxis;

* correct answer.

**Table 1:** Descriptive measures and bivariate regression analysis with crude prevalence ratios of factors associated with adequate knowledge of university students about HIV infection and pre- and post-exposure prophylaxis, Maringá, Paraná, Brazil, 2023

Variable	n (%)	Prevalence	cPR (95%CI)	*p* value^*^
Gender				< 0.01
Male	170 (33.80)	37.65	Reference	
Female	333 (66.20)	22.82	0.89 (0.83-0.95)	
Age group				< 0.01
18 to 21 years	239 (47.52)	20.92	Reference	
22 to 24 years	156 (31.01)	29.49	1.07 (0.99-1.14)	
> 24 years	108 (21.47)	40.74	1.16 (1.07-1.25)	
Sexual orientation				< 0.01
Heterosexual	300 (59.65)	20.33	Reference	
Homosexual	72 (14.31)	51.39	1.25 (1.15-1.37)	
Bisexual/pansexual/asexual	131 (26.04)	32.06	1.09 (1.02-1.17)	
Race/color				0.53
Brown	91 (18.09)	21.98	Reference	
Black	29 (5.77)	31.03	1.07 (0.92-1.24)	
White	361 (71.77)	28.81	1.05 (0.97-1.14)	
Yellow	22 (4.37)	31.82	1.08 (0.91-1.27)	
Religious belief				0.02
Has religion	336 (66.80)	24.70	Reference	
Does not have religion	167 (33.20)	34.13	1.07 (1.00-1.14)	
Romantic relationship				0.77
No	275 (54.67)	28.36	Reference	
Yes	228 (45.33)	27.19	0.99 (0.93-1.05)	
Family income^†^				0.40
< 2 minimum wages	110 (21.87)	27.27	Reference	
2 to 4 minimum wages	179 (35.59)	31.28	1.03 (0.94-1.21)	
> 4 minimum wages	214 (42.54)	25.23	0.98 (0.90-1.06)	
Field of knowledge				< 0.01
Health	107 (21.27)	49.53	Reference	
Agricultural and biological	61 (12.13)	18.03	0.78 (0.71-0.87)	
Exact sciences and technology	116 (23.06)	19.83	0.80 (0.73-0.87)	
Humanities and social sciences	219 (43.54)	24.20	0.83 (0.76-0.89)	
Campus of the institution				< 0.01
Main	447 (88.87)	29.75	Reference	
Other	56 (11.13)	12.50	0.86 (0.79-0.94)	
Time at the institution				0.20
< 1 year	98 (19.48)	23.47	Reference	
1 to 2 years	182 (36.18)	28.02	1.03 (0.95-1.12)	
3 to 5 years	199 (39.57)	27.64	1.03 (0.95-1.12)	
> 5 years	24 (4.77)	45.83	1.18 (1.01-1.37)	
Sexual relation (last three months)				< 0.01
Had sexual relations	156 (31.01)	17.31	Reference	
Did not have sexual relations	347 (68.99)	32.56	1.13 (1.06-1.20)	
Sexual partnership (last three months)				< 0.01
Steady partnership	263 (52.29)	27.76	Reference	
Multiple partnerships	121 (24.06)	41.32	1.10 (1.02-1.19)	
No partnership	119 (23.66)	14.29	0.89 (0.83-0.95)	
Underwent rapid HIV test				< 0.01
No	347 (68.99)	17.87	Reference	
Yes	156 (31.01)	50.00	1.27 (1.19-1.35)	
Heard about PrEP				< 0.01
No	270 (53.68)	4.81	Reference	
Yes	233 (46.32)	54.51	1.47 (1.40-1.54)	
Heard about PEP				< 0.01
No	249 (49.50)	5.62	Reference	
Yes	254 (50.50)	49.61	1.41 (1.34-1.48)	
Used PrEP				0.02
No	483 (96.02)	26.92	Reference	
Yes	20 (3.98)	50.00	1.18 (1.01-1.37)	
Used PEP				< 0.01
No	483 (96.02)	25.88	Reference	
Yes	20 (3.98)	75.00	1.39 (1.24-1.55)	
Knows what HIV is				< 0.01
No	6 (1.19)	0.00	Reference	
Yes	497 (98.81)	28.17	1.28 (1.24-1.32)	
Knows what PrEP is				< 0.01
No	277 (55.07)	3.61	Reference	
Yes	226 (44.93)	57.52	1.52 (1.45-1.59)	
Knows what PEP is				< 0.01
No	250 (49.70)	3.20	Reference	
Yes	253 (50.30)	52.17	1.47 (1.40-1.54)	

cPR - crude prevalence ratio; 95%CI - 95% confidence interval (lower limit-upper limit); HIV - human immunodeficiency virus; PrEP - pre-exposure prophylaxis; PEP - post-exposure prophylaxis;

* p value obtained by the Wald test; †minimum wage equal to 1,320.00 reais.

- sociodemographic data: gender (male; female), age range (in years old: 18 to 21; 22 to 24; > 24), sexual orientation (heterosexual; homosexual; bisexual, pansexual, and asexual), race/ethnicity (brown; black; white; yellow), religion (has; does not have), family income (in minimum wages: < 2; 2 to 4; > 4), and romantic relationship status (no; yes);
- educational data: field of knowledge (health; agricultural and biological; exact sciences and technology; humanities and social sciences), campus of study (main; other), and time at the institution (in years: < 1; 1 to 2; 3 to 5; > 5);
- behavioral data: sexual relations in the last quarter (yes; no), type of sexual partnership in the last quarter (steady; multiple; none), and history of undergoing rapid HIV testing at any point (no; yes);
- additional data: awareness of PrEP (no; yes), awareness of PEP (no; yes), usage of PrEP (no; yes), usage of PEP (no; yes), understanding of what HIV is (no; yes), understanding of what PrEP is (no; yes), and understanding of what PEP is (no; yes).

This instrument was evaluated by a panel of judges on (i) the appropriateness of the language for the target audience, (ii) the absence of leading questions, (iii) the comprehensiveness of the content, (iv) the clarity of a single interpretation, and (v) the number of questions^(20)^. The panel included two professionals from specialized care services (SCS), two technical consultants from the Ministry of Health associated with the HIV program, and a faculty member from the UEM nursing course.

It should be noted that the questionnaire (Chart 1) was developed by the researchers of this study through discussions within the Group for Study and Research on HIV/AIDS and Tuberculosis Surveillance (GEPVHAT/UEM), in collaboration with both undergraduate and graduate nursing students (master’s and doctoral). However, the judges played an essential role in the instrument’s adaptation process, resulting in the final version presented in this research.

Data were collected using Google Forms^®^. To prevent duplication, the form was restricted to a single submission per email. An invitation was sent directly to participants via Gmail^®^, using the “blind carbon copy” option to ensure confidentiality. Within the form link, students were provided access to the informed consent form. Throughout the data collection phase, 503 responses were compiled, with no exclusions due to incomplete submissions.

### Analysis of results and statistics

Descriptive analysis was conducted using the arithmetic mean and both absolute (n) and relative (%) frequencies of the characterization data and the knowledge questionnaire. Questions with incorrect/uncertain and correct answers were scored as zero and one, respectively, generating a score range from zero to sixteen. Based on studies^(11,18-19)^, knowledge was categorized by percentiles into: inadequate (≤ 12 questions or ≤ 75% correct) and adequate (> 12 questions or > 75% correct).

From this, the prevalence of adequate knowledge about HIV, PrEP, and PEP among university students was calculated, and also for each category of independent variables. The calculation was performed by dividing the number of people who obtained 13 or more correct answers on the questionnaire by the total number of respondents for the respective question and variable category; the result of this division was then multiplied by 100.

To identify associated factors, a Poisson regression model with robust variance^(21)^ was used, in which the dependent variable was the category of knowledge (inadequate; adequate) and the characterization data were the independent variables. Initially, a bivariate (crude) analysis was conducted with each variable, and to control for potential confounding factors, a multivariate (adjusted) analysis was performed using the stepwise backward method^(22)^.

Before proceeding with the multiple models, multicollinearity was assessed and ruled out based on the variance inflation factor and the tolerance test^(22)^. Variables with a p-value ≤ 0.20 in the Wald test in bivariate analysis were included and, depending on their significance, were removed one by one until only those with a p-value ≤ 0.05 remained. As a measure of association in the models, prevalence ratios (PRs) and their 95% confidence intervals (CI95%) were calculated.

The PRs represented the likelihood that a response category of an independent variable would positively or negatively influence the adequate knowledge-that is, more than 12 correct answers-of the students, compared to the reference category in the same variable. For the final model, the quality of the fit was assessed by the likelihood ratio chi-square test (p-value ≤ 0.05). All analyses were performed using SPSS Statistics^®^ software, version 25.

## RESULTS

**Figure 1:**
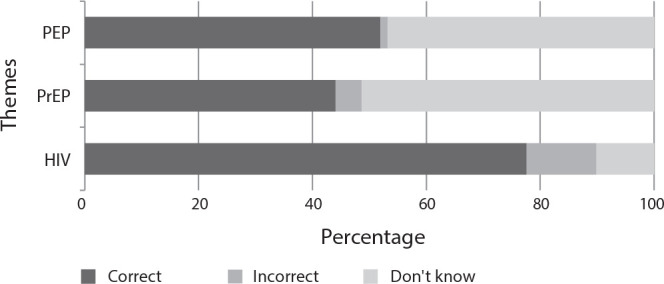
Distribution of university students’ responses to questions, categorized by themes related to HIV infection and pre- and post-exposure prophylaxis, Maringá, Paraná, Brazil, 2023 PEP - post-exposure prophylaxis; PrEP - pre-exposure prophylaxis; HIV - human immunodeficiency virus.

**Figure 2:**
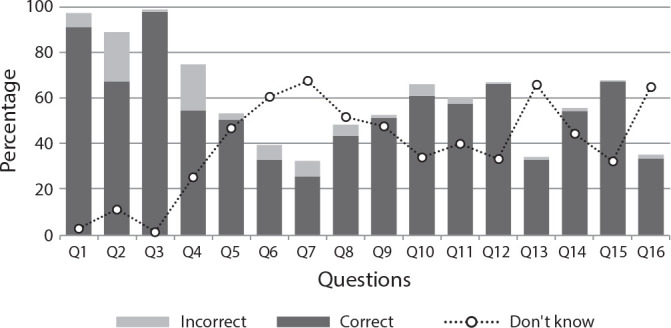
Distribution of university students’ responses to questions related to HIV infection and pre- and post-exposure prophylaxis, Maringá, Paraná, Brazil, 2023

Upon analyzing the average percentage of responses for each thematic axis of the questionnaire, it was observed that the majority of correct responses pertained to HIV (77.63%), followed by responses about PEP (51.92%), and lastly, about PrEP (44.07%) (Figure 1). The responses to each question in the questionnaire are displayed in Figure 2; the questions with the fewest correct responses were: Q6 (n = 166; 33.00%), Q7 (n = 129; 25.65%), Q13 (n = 166; 33.00%), and Q16 (n = 168; 33.40%).

The characteristics of the 503 participants were presented in Table 1. The prevalence of adequate knowledge among participants about HIV infection, PrEP, and PEP was 27.83%. A higher prevalence of correct answers (more than 12) on the questionnaire was observed among males, individuals over 24 years old, of homosexual orientation, of yellow and black race/color, without religious beliefs, and those who did not have a partner (Table 1).

The prevalence of adequate knowledge was also higher among individuals with a family income of 2 to 4 minimum wages, from health-related courses, from the main campus, with more than 5 years at the institution, among those who had not had sexual relations in the last quarter, and those with multiple partnerships. The history of undergoing rapid HIV testing and having heard of, knowing what it is, and having used PrEP and PEP also contributed to a higher prevalence of adequate knowledge (Table 1).

In the final model, individuals over the age of 24 demonstrated greater adequate knowledge (adjusted PR - aPR = 1.08; 95%CI 1.02-1.15) compared to those aged 18 to 21. Health students performed better in the questionnaire than those in agricultural/biological (aPR = 0.90; 95%CI 0.83-0.98) and humanities/social sciences (aPR = 0.93; 95%CI 0.87-0.99). Young people who had not had sexual relations also showed higher knowledge (aPR = 1.07; 95%CI 1.02-1.13) (Table 2).

**Table 2:** Multivariate regression analysis with adjusted prevalence ratios of factors associated with adequate knowledge among university students on HIV infection and pre- and post-exposure prophylaxis, Maringá, Paraná, Brazil, 2023

Variable	aPR (95%CI)	*p* value^*^
Age group		0.02
18 to 21 years	Reference	
22 to 24 years	1.01 (0.96-1.07)	
> 24 years	1.08 (1.02-1.15)	
Field of knowledge		0.04
Health	Reference	
Agricultural and biological	0.90 (0.83-0.98)	
Exact and technology	0.93 (0.86-1.00)	
Humanities and social sciences	0.93 (0.87-0.99)	
Sexual relations (last three months)		< 0.01
Had sexual relations	Reference	
Did not have sexual relations	1.07 (1.02-1.13)	
Rapid HIV test		< 0.01
No	Reference	
Yes	1.09 (1.03-1.15)	
Heard about PrEP		< 0.01
No	Reference	
Yes	1.11 (1.03-1.20)	
Knows what PrEP is		< 0.01
No	Reference	
Yes	1.18 (1.08-1.30)	
Knows what PEP is		< 0.01
No	Reference	
Yes	1.14 (1.07-1.22)	

aPR - adjusted prevalence ratio; CI95% - 95% confidence interval (lower bound-upper bound); HIV - human immunodeficiency virus; PrEP - pre-exposure prophylaxis; PEP - post-exposure prophylaxis;

* p value obtained by the Wald test.

University students who had undergone rapid HIV testing had a higher prevalence of knowledge (aPR = 1.09; 95%CI 1.03-1.15) compared to those who had never done so. Those who perceived they knew what PrEP (aPR = 1.18; 95%CI 1.08-1.30) and PEP (aPR = 1.14; 95%CI 1.07-1.22) were, and those who had heard about PrEP (aPR = 1.11; 95%CI 1.03-1.20) also showed a higher probability of having adequate knowledge (Table 2).

## DISCUSSION

This cross-sectional study involving more than 500 university students demonstrated a low prevalence of adequate knowledge about HIV and its prophylaxis. Knowledge varied according to the sociodemographic, educational, and behavioral characteristics of the students. Those who were older, enrolled in health-related courses, not sexually active in the past three months, had previously taken a rapid HIV test, and knew what PrEP and PEP were had higher accuracy rates.

The limited knowledge on this topic has already been reported in the literature. In the national context, a cross-sectional study at a university in Bahia state found that only 28.5% of students were aware of PEP^(15)^. Internationally, at a rural institution in South Africa, 42.0% of students had adequate knowledge about HIV^(16)^; in Thailand, research indicated that only 20.8% of university students had heard of PrEP^(17)^.

This scenario signals a warning about the potentially limited education in higher education, and even before it, regarding HIV and its prevention. Despite the inclusion of preventive policies in the strategies of the SUS-particularly internal and external condoms and HIV prophylaxes^(23)^-it is imperative to expand the dissemination campaigns of these technologies to target audiences, starting in their school and university environments.

This becomes even more alarming considering that, in this study, most young people had correct answers primarily related to HIV itself and not its preventive measures. Participants did not recognize the minimum age for using PrEP and did not know when the medications could be taken. Regarding PEP, they were unaware of the duration of use and the appropriate time frame to start medications after a risk situation.

Among the associated factors, university students over 24 years old provided more correct answers compared to those aged 18 to 21. This finding may be attributed to the extended period spent in the university environment, which fosters discussions on the subject^(10)^. However, interest in and knowledge of HIV and its prevention methods may be more strongly linked to behavioral and sociocultural factors than to age alone.

Historically, the inclusion of courses on communicable diseases has paralleled political developments and gained prominence with the onset of the HIV epidemic^(24)^. In this context, health-related academic programs have increasingly aligned with public policies on HIV^(24)^. This could explain why students enrolled in health-related fields tend to have a deeper understanding of this topic compared to their peers in other disciplines^(15)^.

Interestingly, young individuals who had not engaged in sexual activity in the past three months showed better knowledge in the survey. In contrast, a study involving MSM observed that those who had sexual relations with multiple partners, as opposed to a single partner or none, demonstrated better knowledge about PrEP^(25)^. This may be due to more extensive information dissemination among this group or their heightened awareness of prevention due to frequent exposure.

This research assumes that university students, theoretically having greater access to information than the general population-especially those in health-related programs^(26)^ - sought to improve their understanding of preventive methods before engaging in sexual activity. However, despite assurances of confidentiality, the potential for information bias cannot be ruled out due to the discomfort some may feel in disclosing their sexual activity to researchers.

A history of undergoing rapid HIV testing was also linked to a higher number of correct responses. A Portuguese study found that individuals who had been tested for HIV were 3.68 times more likely (95%CI 3.11-4.36) to possess knowledge about PrEP^(27)^. It is believed that closer engagement with testing and counseling services can enhance access to information about HIV and current prevention strategies.

In Brazil, testing and counseling centers (TCCs) have consistently advocated for providing tests along with health education on STIs and their prevention methods since their establishment^(28)^. Thus, this study supports the effectiveness of this national public policy, highlighting the need to expand and strengthen it throughout all healthcare services to ensure timely and consistent information dissemination to the public.

It is also worth noting that at the educational institution in question, besides having a clinic staffed with medical and nursing teams, educational campaigns have been conducted in partnership with the municipal TCC. These strategies may focus on disseminating information about STIs preventive methods and expanding the availability and access to rapid testing, which could have contributed to greater knowledge among students who have undergone testing.

The reported perception of familiarity with PrEP and PEP also increased the likelihood of achieving more correct answers on the questionnaire of our study. This finding aligns with the literature^(25,27)^, but raises concerns highlighted in a previous research where 91.1% of participants had heard of PrEP; however, only 7.5% of them reported having used it, despite 52.1% expressing interest in using it-if better informed^(25)^.

In this context, it is necessary to do more than discuss available strategies for combating HIV. We must rethink behaviors to facilitate informed individual choices regarding the use of PrEP and PEP. This includes sensitizing and training professionals because, despite the potential benefits of educational guidance concurrent with testing and consultations, the performance of TCCs has been inadequate in certain areas of the country^(29)^.

Moreover, it is important to consider that actions to be developed with students need to take into account the social representation of STIs within this group. Young people recognize unprotected sexual practices as risk factors and understand the necessity of using condoms as a method of preventing infections; however, fear and stigma are embedded in their perceptions, making it difficult to adopt other current preventive practices^(30)^.

Therefore, collaboration between academia and professionals involved in care, management, and surveillance is encouraged, focusing on promoting campaigns that facilitate access to knowledge within the university environment. Additionally, there is a need to review the internal actions of the university in its role as an educator, ensuring that efforts are expanded to include students from all fields, especially those not enrolled in health-related courses.

### Study limitations

This study exhibits several significant limitations beyond those inherent to cross-sectional designs, including: (i) the sampling restricted to a single location, (ii) the aggregation of students from various disciplines into sub-variables, (iii) the reliance on a self-administered, online questionnaire, (iv) the potential for information bias in responses, (v) the absence of validation for the assessment instrument, and (vi) the consolidation of questions on disparate topics (HIV, PrEP, and PEP) into a single dependent variable.

### Contributions to the field of nursing, health, or public policy

The findings are of substantial relevance to public health and education, serving as a call to action for educators, administrators, and health professionals to enhance educational and awareness programs within university settings, as well as to integrate these topics into the compulsory curricula of all courses. This effort is crucial to expanding knowledge and improving access to and adherence to HIV preventive measures in this group.

Furthermore, this research is particularly notable for addressing a critical issue for adolescents and young adults in light of the rising HIV incidence within this demographic. It posits that strategies aimed at equipping university students with comprehensive knowledge of combined prevention methods and technologies are vital for hastening the response to HIV, focusing on achieving the ambitious SDGs by 2030.

## CONCLUSIONS

The knowledge of young individuals about HIV, PrEP, and PEP was generally found to be inadequate, highlighting the need for enhanced educational efforts among this vulnerable group. Notably, older youths, those enrolled in health-related courses, individuals inactive sexually in the past three months, those who had undergone rapid HIV testing, and those familiar with PrEP and PEP demonstrated higher scores.

Given this, it is imperative to develop educational strategies aimed at raising awareness of the topic within the university, which constitutes an important space for the exchange of knowledge and practices among peers. To this end, it is essential to consider the particularities embedded in the context of youth, so that the strategies can address taboos and sociocultural constructs that may influence habits and behaviors, especially those of individuals in greater vulnerability.

## Supplementary Material

0034-7167-reben-77-s2-e20240092-suppl01

## Data Availability

https://doi.org/10.17632/7jmv72wjxm.1
