# Supplementary material for: Factors associated with university students’ knowledge about HIV and pre- and post-exposure prophylaxis
Source: Rev Bras Enferm. 2024 Aug 30;77(Suppl 2):e20240092. doi: 10.1590/0034-7167-2024-0092 (PMC11370771; doi:10.1590/0034-7167-2024-0092)
Supplement: 0034-7167-reben-77-s2-e20240092-suppl01 [file 0034-7167-reben-77-s2-e20240092-suppl01.pdf]

OrientaçãoSexual	EtniaCor	CrençaReligiosa	EstadoCivil	Curso
Heterossexual	Branca	Ateísmo	amoro/em relacionamer	Agronomia
Bissexual	Preta	Agnosticismo	Solteiro(a)	Direito
Heterossexual	Parda	Catolicismo	amoro/em relacionamer	Geografia
Bissexual	Branca	Ateísmo	Solteiro(a)	Ciências Biológicas
Bissexual	Branca	Evangelismo	amoro/em relacionamer	Psicologia
Bissexual	Branca	Ateísmo	amoro/em relacionamer	Direito
Heterossexual	Amarela	Evangelismo	Solteiro(a)	Química
Heterossexual	Amarela	Agnosticismo	amoro/em relacionamer	Enfermagem
Homossexual	Parda	Catolicismo	amoro/em relacionamer	Educação Física
Homossexual	Branca	Catolicismo	Solteiro(a)	Biomedicina
Heterossexual	Branca	Catolicismo	amoro/em relacionamer	Psicologia
Bissexual	Branca	Ateísmo	amoro/em relacionamer	Psicologia
Bissexual	Branca	Ateísmo	Solteiro(a)	Psicologia
Bissexual	Branca	Agnosticismo	Solteiro(a)	Enfermagem
Heterossexual	Branca	Ateísmo	amoro/em relacionamer	Artes Cênicas
Heterossexual	Preta	Evangelismo	amoro/em relacionamer	Ciências Contábeis
Heterossexual	Parda	Espiritismo	Solteiro(a)	Enfermagem
Heterossexual	Branca	Espiritismo	Solteiro(a)	Psicologia
Heterossexual	Branca	Catolicismo	amoro/em relacionamer	Direito
Bissexual	Branca	Catolicismo	amoro/em relacionamer	Enfermagem
Heterossexual	Branca	Catolicismo	amoro/em relacionamer	Farmácia
Pansexual	Branca	Agnosticismo	amoro/em relacionamer	Enfermagem
Heterossexual	Parda	Catolicismo	amoro/em relacionamer	Enfermagem
Heterossexual	Branca	Catolicismo	amoro/em relacionamer	Odontologia
Heterossexual	Branca	Catolicismo	Solteiro(a)	Enfermagem
Heterossexual	Branca	Evangelismo	amoro/em relacionamer	Enfermagem
Bissexual	Branca	Catolicismo	amoro/em relacionamer	Ciências Biológicas
Heterossexual	Preta	Ateísmo	amoro/em relacionamer	Ciências Biológicas
Heterossexual	Branca	Evangelismo	Casamento/união estáve	Enfermagem
Bissexual	Branca	Catolicismo	Solteiro(a)	Enfermagem
Homossexual	Branca	Agnosticismo	Solteiro(a)	Ciências Biológicas
Heterossexual	Parda	Evangelismo	Solteiro(a)	Enfermagem
Heterossexual	Branca	Catolicismo	amoro/em relacionamer	Enfermagem
Heterossexual	Branca	Evangelismo	Solteiro(a)	Bioquímica
Heterossexual	Branca	Catolicismo	amoro/em relacionamer	Enfermagem
Homossexual	Branca	Ateísmo	amoro/em relacionamer	Psicologia
Bissexual	Branca	Espiritismo	Solteiro(a)	Psicologia
Heterossexual	Preta	Catolicismo	amoro/em relacionamer	Enfermagem
Bissexual	Branca	Agnosticismo	Solteiro(a)	Biomedicina
Homossexual	Parda	Agnosticismo	amoro/em relacionamer	Psicologia
Homossexual	Branca	Evangelismo	Solteiro(a)	Enfermagem
Heterossexual	Parda	Evangelismo	Solteiro(a)	Administração
Heterossexual	Branca	Catolicismo	amoro/em relacionamer	Ciência da Computaçã
Heterossexual	Branca	Catolicismo	Casamento/união estáve	Ciências Contábeis
Homossexual	Parda	Agnosticismo	Solteiro(a)	Ciências Biológicas
Bissexual	Parda	Evangelismo	amoro/em relacionamer	Odontologia
Heterossexual	Branca	Catolicismo	Solteiro(a)	Enfermagem
Heterossexual	Branca	Evangelismo	Solteiro(a)	Enfermagem
Heterossexual	Parda	Evangelismo	amoro/em relacionamer	Enfermagem
Heterossexual	Branca	Catolicismo	Solteiro(a)	Administração
Bissexual	Branca	Agnosticismo	Solteiro(a)	etariado Executivo Trilí
Heterossexual	Branca	Catolicismo	amoro/em relacionamer	Medicina
Homossexual	Branca	Ateísmo	amoro/em relacionamer	Ciência da Computaçã

Heterossexual	Branca	Catolicismo	amoro/em relacionamer	Bioquímica
Heterossexual	Branca	Umbandismo	Solteiro(a)	Direito
Heterossexual	Parda	Catolicismo	amoro/em relacionamer	etariado Executivo Trilí
Homossexual	Branca	Catolicismo	Solteiro(a)	Engenharia Química
Homossexual	Parda	Umbandismo	Solteiro(a)	Psicologia
Heterossexual	Branca	Catolicismo	Solteiro(a)	Enfermagem
Heterossexual	Parda	Catolicismo	Solteiro(a)	Física
Heterossexual	Branca	Agnosticismo	Solteiro(a)	Química
Heterossexual	Amarela	Catolicismo	Solteiro(a)	Enfermagem
Bissexual	Branca	Agnosticismo	Solteiro(a)	Direito
Bissexual	Branca	Catolicismo	amoro/em relacionamer	Odontologia
Bissexual	Parda	Catolicismo	Solteiro(a)	Enfermagem
Homossexual	Parda	Ateísmo	amoro/em relacionamer	Pedagogia
Heterossexual	Branca	Catolicismo	amoro/em relacionamer	Odontologia
Bissexual	Parda	Evangelismo	Solteiro(a)	Letras
Heterossexual	Branca	Catolicismo	amoro/em relacionamer	Odontologia
Bissexual	Branca	Agnosticismo	Solteiro(a)	Odontologia
Bissexual	Branca	Agnosticismo	Solteiro(a)	Psicologia
Bissexual	Preta	Umbandismo	Solteiro(a)	Psicologia
Heterossexual	Branca	Catolicismo	amoro/em relacionamer	Odontologia
Heterossexual	Branca	Catolicismo	amoro/em relacionamer	Enfermagem
Heterossexual	Branca	Catolicismo	amoro/em relacionamer	Odontologia
Bissexual	Branca	Catolicismo	Solteiro(a)	História
Heterossexual	Branca	Catolicismo	amoro/em relacionamer	Odontologia
Heterossexual	Branca	Catolicismo	Solteiro(a)	Odontologia
Bissexual	Branca	Catolicismo	Solteiro(a)	Odontologia
Heterossexual	Preta	Catolicismo	amoro/em relacionamer	Odontologia
Bissexual	Branca	Ateísmo	amoro/em relacionamer	Artes Visuais
Heterossexual	Parda	Catolicismo	amoro/em relacionamer	ologia em Construção
Bissexual	Branca	Agnosticismo	Solteiro(a)	Enfermagem
Homossexual	Parda	Ateísmo	Solteiro(a)	Ciências Contábeis
Heterossexual	Branca	Catolicismo	Solteiro(a)	Ciências Biológicas
Bissexual	Parda	Agnosticismo	Solteiro(a)	Pedagogia
Heterossexual	Branca	Catolicismo	amoro/em relacionamer	Artes Visuais
Heterossexual	Branca	Evangelismo	Solteiro(a)	Física
Heterossexual	Preta	Catolicismo	amoro/em relacionamer	Direito
Homossexual	Branca	Ateísmo	Solteiro(a)	Psicologia
Homossexual	Amarela	Agnosticismo	Solteiro(a)	Psicologia
Heterossexual	Branca	Catolicismo	amoro/em relacionamer	Engenharia de Produçã
Heterossexual	Branca	Catolicismo	amoro/em relacionamer	Ciências Econômicas
Heterossexual	Branca	Catolicismo	amoro/em relacionamer	Direito
Heterossexual	Branca	Agnosticismo	amoro/em relacionamer	Agronomia
Heterossexual	Branca	Catolicismo	amoro/em relacionamer	Medicina
Heterossexual	Branca	Ateísmo	Solteiro(a)	Engenharia de Produçã
Heterossexual	Branca	Catolicismo	Solteiro(a)	Direito
Heterossexual	Amarela	Ateísmo	Solteiro(a)	Ciências Econômicas
Heterossexual	Parda	Catolicismo	Solteiro(a)	História
Heterossexual	Branca	Catolicismo	Solteiro(a)	Engenharia de Produçã
Bissexual	Branca	Ateísmo	amoro/em relacionamer	Psicologia
Bissexual	Branca	Agnosticismo	Solteiro(a)	Geografia
Bissexual	Parda	Catolicismo	Solteiro(a)	Ciências Contábeis
Bissexual	Branca	Agnosticismo	Solteiro(a)	Artes Visuais
Heterossexual	Branca	Catolicismo	amoro/em relacionamer	Psicologia
Heterossexual	Branca	Catolicismo	Solteiro(a)	Geografia

Heterossexual	Amarela	Catolicismo	Solteiro(a)	Medicina
Heterossexual	Branca	Agnosticismo	amoro/em relacionamer	Informática
Heterossexual	Branca	Ateísmo	Solteiro(a)	Ciências Biológicas
Heterossexual	Branca	Catolicismo	amoro/em relacionamer	Ciência da Computaçãc
Heterossexual	Amarela	Catolicismo	amoro/em relacionamer	Engenharia Elétrica
Bissexual	Branca	Catolicismo	amoro/em relacionamer	Farmácia
Bissexual	Branca	Ateísmo	Solteiro(a)	Medicina Veterinária
Heterossexual	Parda	Catolicismo	amoro/em relacionamer	Ciências Contábeis
Heterossexual	Branca	Evangelismo	amoro/em relacionamer	Psicologia
Heterossexual	Branca	Catolicismo	Solteiro(a)	Pedagogia
Heterossexual	Branca	Catolicismo	amoro/em relacionamer	Pedagogia
Homossexual	Branca	Catolicismo	Solteiro(a)	Engenharia de Alimento
Heterossexual	Branca	Catolicismo	amoro/em relacionamer	Psicologia
Heterossexual	Preta	Catolicismo	Solteiro(a)	Geografia
Heterossexual	Amarela	Agnosticismo	Solteiro(a)	Direito
Heterossexual	Amarela	Evangelismo	Solteiro(a)	Psicologia
Heterossexual	Branca	Ateísmo	amoro/em relacionamer	Física
Bissexual	Amarela	Ateísmo	Solteiro(a)	Engenharia Têxtil
Homossexual	Branca	Agnosticismo	Solteiro(a)	Matemática
Homossexual	Branca	Catolicismo	Solteiro(a)	Geografia
Assexual	Parda	Evangelismo	Solteiro(a)	omunicação e Multimei
Bissexual	Branca	Agnosticismo	amoro/em relacionamer	Arquitetura e Urbanismc
Pansexual	Branca	Agnosticismo	amoro/em relacionamer	Psicologia
Heterossexual	Parda	Catolicismo	amoro/em relacionamer	Zootecnia
Heterossexual	Branca	Agnosticismo	amoro/em relacionamer	Ciência da Computaçãc
Heterossexual	Branca	Catolicismo	amoro/em relacionamer	Direito
Heterossexual	Preta	Evangelismo	amoro/em relacionamer	Direito
Pansexual	Branca	Paganismo	Solteiro(a)	Zootecnia
Homossexual	Parda	Catolicismo	amoro/em relacionamer	História
Bissexual	Branca	Ateísmo	amoro/em relacionamer	Direito
Homossexual	Branca	Ateísmo	Solteiro(a)	Direito
Heterossexual	Branca	Agnosticismo	amoro/em relacionamer	Matemática
Heterossexual	Branca	Evangelismo	Solteiro(a)	Bioquímica
Bissexual	Branca	Ateísmo	Solteiro(a)	Letras
Bissexual	Parda	Agnosticismo	Solteiro(a)	Ciências Biológicas
Heterossexual	Branca	Agnosticismo	amoro/em relacionamer	Matemática
Heterossexual	Parda	Catolicismo	Solteiro(a)	Química
Homossexual	Amarela	Agnosticismo	Solteiro(a)	Biomedicina
Heterossexual	Parda	Catolicismo	Solteiro(a)	Ciência da Computaçãc
Heterossexual	Branca	Catolicismo	amoro/em relacionamer	Engenharia Civil
Heterossexual	Branca	Ateísmo	Solteiro(a)	Ciências Econômicas
Bissexual	Branca	Catolicismo	amoro/em relacionamer	Física
Bissexual	Branca	Agnosticismo	Solteiro(a)	Psicologia
Heterossexual	Parda	Catolicismo	amoro/em relacionamer	Arquitetura e Urbanismc
Bissexual	Branca	Catolicismo	Solteiro(a)	Enfermagem
Heterossexual	Branca	Catolicismo	Solteiro(a)	Zootecnia
Heterossexual	Branca	Evangelismo	amoro/em relacionamer	Biomedicina
Heterossexual	Preta	Ceticismo	Solteiro(a)	Artes Cênicas
Heterossexual	Branca	Catolicismo	Solteiro(a)	Psicologia
Bissexual	Parda	Agnosticismo	Solteiro(a)	Ciências Contábeis
Heterossexual	Branca	Evangelismo	amoro/em relacionamer	Bioquímica
Heterossexual	Branca	Catolicismo	amoro/em relacionamer	Enfermagem
Heterossexual	Branca	Catolicismo	Solteiro(a)	Ciências Contábeis
Homossexual	Branca	Evangelismo	Solteiro(a)	Farmácia

Heterossexual	Branca	Catolicismo	amoro/em relacionamen	Ciência da Computaçã
Heterossexual	Branca	Catolicismo	Solteiro(a)	Agronomia
Heterossexual	Branca	Catolicismo	amoro/em relacionamen	ngenharia de Produçã
Pansexual	Branca	Agnosticismo	Solteiro(a)	Farmácia
Heterossexual	Branca	Catolicismo	amoro/em relacionamen	Artes Cênicas
Heterossexual	Branca	Catolicismo	amoro/em relacionamen	Engenharia Elétrica
Heterossexual	Branca	Evangelismo	Solteiro(a)	Zootecnia
Heterossexual	Parda	Evangelismo	amoro/em relacionamen	Administração
Heterossexual	Branca	Catolicismo	Solteiro(a)	Letras
Homossexual	Branca	Ateísmo	Solteiro(a)	ngenharia de Produçã
Heterossexual	Preta	Catolicismo	Solteiro(a)	Engenharia Civil
Heterossexual	Branca	Catolicismo	amoro/em relacionamen	Pedagogia
Heterossexual	Branca	Catolicismo	Solteiro(a)	Direito
Bissexual	Branca	Agnosticismo	Solteiro(a)	Direito
Heterossexual	Branca	Catolicismo	amoro/em relacionamen	Direito
Heterossexual	Branca	Espiritismo	Solteiro(a)	rquitetura e Urbanism
Heterossexual	Branca	Agnosticismo	asamento/união estáve	Ciências Contábeis
Bissexual	Branca	Catolicismo	Solteiro(a)	Serviço Social
Heterossexual	Branca	Evangelismo	amoro/em relacionamen	rquitetura e Urbanism
Heterossexual	Branca	Catolicismo	amoro/em relacionamen	Pedagogia
Pansexual	Preta	Candombleismo	amoro/em relacionamen	Ciências Biológicas
Heterossexual	Branca	Catolicismo	Solteiro(a)	Agronomia
Heterossexual	Parda	Catolicismo	Solteiro(a)	Psicologia
Heterossexual	Parda	Catolicismo	Solteiro(a)	Enfermagem
Heterossexual	Branca	Ateísmo	amoro/em relacionamen	Farmácia
Heterossexual	Branca	Catolicismo	amoro/em relacionamen	Serviço Social
Heterossexual	Parda	Evangelismo	amoro/em relacionamen	Engenharia Civil
Bissexual	Branca	Catolicismo	Solteiro(a)	Psicologia
Assexual	Branca	Agnosticismo	Solteiro(a)	Farmácia
Heterossexual	Branca	Catolicismo	Solteiro(a)	Agronomia
Heterossexual	Preta	Catolicismo	Solteiro(a)	Biomedicina
Homossexual	Branca	Catolicismo	Solteiro(a)	rquitetura e Urbanism
Heterossexual	Branca	Evangelismo	Solteiro(a)	Enfermagem
Bissexual	Branca	Agnosticismo	amoro/em relacionamen	História
Heterossexual	Parda	Catolicismo	amoro/em relacionamen	Medicina Veterinária
Homossexual	Branca	Catolicismo	Solteiro(a)	Serviço Social
Heterossexual	Preta	Catolicismo	Solteiro(a)	Medicina Veterinária
Bissexual	Branca	Ateísmo	Solteiro(a)	Artes Visuais
Heterossexual	Branca	Catolicismo	Solteiro(a)	Medicina Veterinária
Heterossexual	Branca	Evangelismo	amoro/em relacionamen	Enfermagem
Heterossexual	Parda	Evangelismo	amoro/em relacionamen	Farmácia
Bissexual	Branca	Agnosticismo	Solteiro(a)	Letras
Heterossexual	Branca	Catolicismo	amoro/em relacionamen	Física
Bissexual	Branca	Sincretismo	Solteiro(a)	Engenharia Civil
Bissexual	Parda	Agnosticismo	amoro/em relacionamen	Ciências Biológicas
Bissexual	Branca	Catolicismo	Solteiro(a)	Psicologia
Bissexual	Branca	Agnosticismo	Solteiro(a)	História
Heterossexual	Branca	Catolicismo	amoro/em relacionamen	Ciências Econômicas
Homossexual	Branca	Ateísmo	Solteiro(a)	história
Heterossexual	Branca	Espiritismo	Solteiro(a)	Engenharia Mecânica
Heterossexual	Branca	Catolicismo	Solteiro(a)	rquitetura e Urbanism
Heterossexual	Branca	Catolicismo	amoro/em relacionamen	Direito
Heterossexual	Parda	Catolicismo	Solteiro(a)	ngenharia de Produçã
Bissexual	Parda	Agnosticismo	Solteiro(a)	Pedagogia

Heterossexual	Branca	Catolicismo	amoro/em relacionamer	Engenharia Química
Heterossexual	Branca	Catolicismo	amoro/em relacionamer	Administração
Bissexual	Parda	Catolicismo	amoro/em relacionamer	Matemática
Heterossexual	Preta	Catolicismo	amoro/em relacionamer	Letras
Bissexual	Branca	Sincretismo	amoro/em relacionamer	Artes Visuais
Heterossexual	Branca	Catolicismo	Solteiro(a)	Física
Heterossexual	Parda	Catolicismo	amoro/em relacionamer	Ciências Contábeis
Heterossexual	Branca	Agnosticismo	Solteiro(a)	Artes Visuais
Homossexual	Branca	Ateísmo	Solteiro(a)	Letras
Bissexual	Branca	Catolicismo	amoro/em relacionamer	Psicologia
Heterossexual	Branca	Evangelismo	Solteiro(a)	Matemática
Heterossexual	Branca	Catolicismo	amoro/em relacionamer	Ciências Contábeis
Heterossexual	Branca	Catolicismo	Solteiro(a)	Engenharia Elétrica
Heterossexual	Branca	Agnosticismo	Solteiro(a)	Pedagogia
Heterossexual	Branca	Espiritismo	Solteiro(a)	Psicologia
Heterossexual	Branca	Catolicismo	amoro/em relacionamer	Engenharia de Produçã
Heterossexual	Branca	Ateísmo	amoro/em relacionamer	Ciência da Computaçã
Homossexual	Branca	Ateísmo	Solteiro(a)	Psicologia
Pansexual	Branca	Evangelismo	amoro/em relacionamer	Pós graduação
Heterossexual	Branca	Espiritismo	Solteiro(a)	Ciências Contábeis
Pansexual	Branca	Agnosticismo	Solteiro(a)	Administração
Heterossexual	Branca	Agnosticismo	Solteiro(a)	Engenharia Química
Homossexual	Branca	Catolicismo	Solteiro(a)	Medicina
Heterossexual	Preta	Ateísmo	Casamento/união estáve	Física
Bissexual	Branca	Agnosticismo	amoro/em relacionamer	Artes Cênicas
Heterossexual	Branca	Catolicismo	Solteiro(a)	Bioquímica
Heterossexual	Branca	Catolicismo	Solteiro(a)	Engenharia de Produçã
Heterossexual	Parda	Catolicismo	amoro/em relacionamer	Engenharia Civil
Heterossexual	Branca	Ateísmo	Solteiro(a)	Direito
Bissexual	Branca	Catolicismo	Solteiro(a)	Engenharia Química
Heterossexual	Parda	Catolicismo	amoro/em relacionamer	Física
Heterossexual	Branca	Ateísmo	Solteiro(a)	Historia
Heterossexual	Branca	Evangelismo	Solteiro(a)	etariado Executivo Trilí
Pansexual	Branca	Ceticismo	Solteiro(a)	Farmácia
Homossexual	Branca	Espiritismo	Solteiro(a)	Biomedicina
Heterossexual	Branca	Evangelismo	Solteiro(a)	Ciências Biológicas
Homossexual	Branca	Catolicismo	amoro/em relacionamer	Direito
Heterossexual	Branca	Catolicismo	amoro/em relacionamer	Matemática
Heterossexual	Branca	Catolicismo	Solteiro(a)	Direito
Heterossexual	Branca	Agnosticismo	Solteiro(a)	Ciências Econômicas
Heterossexual	Branca	Agnosticismo	Solteiro(a)	Física
Homossexual	Branca	Espiritismo	Solteiro(a)	Odontologia
Bissexual	Branca	Evangelismo	amoro/em relacionamer	Pedagogia
Pansexual	Branca	Ateísmo	amoro/em relacionamer	Direito
Heterossexual	Branca	Catolicismo	amoro/em relacionamer	Farmácia
Heterossexual	Branca	Agnosticismo	amoro/em relacionamer	Bioquímica
Heterossexual	Branca	Catolicismo	Solteiro(a)	História
Homossexual	Branca	Catolicismo	Solteiro(a)	Engenharia Civil
Heterossexual	Branca	Catolicismo	Solteiro(a)	Física
Bissexual	Branca	Agnosticismo	Solteiro(a)	Física Médica
Heterossexual	Branca	Catolicismo	Solteiro(a)	Bioquímica
Heterossexual	Branca	Evangelismo	Solteiro(a)	Química
Heterossexual	Branca	Catolicismo	amoro/em relacionamer	Farmácia
Bissexual	Parda	Evangelismo	amoro/em relacionamer	Biomedicina

Homossexual	Parda	Agnosticismo	Solteiro(a)	Música
Bissexual	Parda	Catolicismo	amoro/em relacionamer	Matemática
Heterossexual	Parda	Catolicismo	Solteiro(a)	Medicina
Heterossexual	Parda	Evangelismo	Solteiro(a)	Engenharia Ambiental
Heterossexual	Branca	Espiritismo	amoro/em relacionamer	Letras
Bissexual	Amarela	Catolicismo	Solteiro(a)	Engenharia de Produçã
Bissexual	Branca	Agnosticismo	Solteiro(a)	Química
Bissexual	Parda	Agnosticismo	amoro/em relacionamer	Pedagogia
Heterossexual	Branca	Ateísmo	Solteiro(a)	Engenharia de Alimento
Heterossexual	Parda	Evangelismo	Solteiro(a)	Educação Física
Heterossexual	Amarela	Catolicismo	Solteiro(a)	Geografia
Bissexual	Parda	Agnosticismo	amoro/em relacionamer	omunicação e Multimei
Heterossexual	Amarela	Budismo	amoro/em relacionamer	Farmácia
Homossexual	Branca	Agnosticismo	amoro/em relacionamer	Geografia
Bissexual	Branca	Agnosticismo	Solteiro(a)	Medicina
Bissexual	Branca	Agnosticismo	Solteiro(a)	Ciências Biológicas
Heterossexual	Branca	Catolicismo	Solteiro(a)	Ciências Contábeis
Bissexual	Amarela	Agnosticismo	Solteiro(a)	Enfermagem
Bissexual	Branca	Evangelismo	Solteiro(a)	Psicologia
Homossexual	Branca	Agnosticismo	Solteiro(a)	Engenharia de Produçã
Heterossexual	Branca	Evangelismo	Solteiro(a)	Ciências Contábeis
Homossexual	Branca	Catolicismo	amoro/em relacionamer	Engenharia Química
Bissexual	Branca	Ateísmo	Solteiro(a)	Biomedicina
Homossexual	Parda	Ateísmo	Solteiro(a)	Ciências Biológicas
Heterossexual	Branca	Ateísmo	Solteiro(a)	Filosofia
Heterossexual	Branca	Catolicismo	amoro/em relacionamer	Medicina
Heterossexual	Parda	Espiritismo	amoro/em relacionamer	Administração
Heterossexual	Branca	Agnosticismo	amoro/em relacionamer	Engenharia Civil
Heterossexual	Branca	Evangelismo	Casamento/união estáve	Engenharia Civil
Homossexual	Parda	Catolicismo	Solteiro(a)	Farmácia
Assexual	Branca	Agnosticismo	Solteiro(a)	Biomedicina
Homossexual	Branca	Budismo	Solteiro(a)	Letras
Bissexual	Branca	Umbandismo	amoro/em relacionamer	Letras
Homossexual	Branca	Ateísmo	amoro/em relacionamer	Estatística
Bissexual	Branca	Catolicismo	amoro/em relacionamer	Ciências Biológicas
Homossexual	Branca	Ateísmo	amoro/em relacionamer	Letras
Heterossexual	Parda	Catolicismo	Casamento/união estáve	Zootecnia
Heterossexual	Branca	Catolicismo	amoro/em relacionamer	Ciências Biológicas
Heterossexual	Branca	Agnosticismo	amoro/em relacionamer	Biomedicina
Homossexual	Branca	Agnosticismo	Solteiro(a)	Engenharia Química
Bissexual	Branca	Agnosticismo	amoro/em relacionamer	Ciências Biológicas
Homossexual	Branca	Catolicismo	Solteiro(a)	Ciências Contábeis
Heterossexual	Branca	Evangelismo	amoro/em relacionamer	Ciências Econômicas
Heterossexual	Parda	Evangelismo	Solteiro(a)	História
Bissexual	Branca	Ateísmo	Solteiro(a)	Psicologia
Heterossexual	Branca	Agnosticismo	Solteiro(a)	Ciência da Computação
Bissexual	Branca	Catolicismo	Solteiro(a)	Direito
Heterossexual	Branca	Ateísmo	Solteiro(a)	Medicina
Heterossexual	Branca	Catolicismo	Solteiro(a)	Agronomia
Heterossexual	Branca	Catolicismo	Casamento/união estáve	Engenharia de Alimento
Bissexual	Branca	Agnosticismo	Solteiro(a)	Filosofia
Heterossexual	Branca	Catolicismo	Solteiro(a)	Informática
Heterossexual	Branca	Catolicismo	Solteiro(a)	Ciências Contábeis
Heterossexual	Branca	Ateísmo	amoro/em relacionamer	Ciências Contábeis

Heterossexual	Branca	Evangelismo	Solteiro(a)	Química
Heterossexual	Branca	Catolicismo	amoro/em relacionamer	Agronomia
Bissexual	Branca	Agnosticismo	Solteiro(a)	Engenharia Civil
Bissexual	Parda	Catolicismo	Solteiro(a)	Engenharia de Produçã
Heterossexual	Branca	Evangelismo	Casamento/união estáve	Medicina
Heterossexual	Branca	Catolicismo	amoro/em relacionamer	Medicina Veterinária
Pansexual	Branca	Ateísmo	Solteiro(a)	História
Heterossexual	Branca	Agnosticismo	Solteiro(a)	Bioquímica
Heterossexual	Branca	Catolicismo	amoro/em relacionamer	Agronomia
Heterossexual	Branca	Catolicismo	Solteiro(a)	Farmácia
Bissexual	Branca	Ateísmo	Solteiro(a)	Psicologia
Bissexual	Branca	Evangelismo	amoro/em relacionamer	História
Heterossexual	Amarela	Catolicismo	Solteiro(a)	Ciência da Computaçãc
Heterossexual	Parda	Catolicismo	Casamento/união estáve	Zootecnia
Homossexual	Branca	Evangelismo	amoro/em relacionamer	Farmácia
Heterossexual	Amarela	Catolicismo	Solteiro(a)	Engenharia Mecânica
Heterossexual	Branca	Catolicismo	Solteiro(a)	Medicina Veterinária
Heterossexual	Branca	Catolicismo	amoro/em relacionamer	Direito
Heterossexual	Parda	Catolicismo	amoro/em relacionamer	Educação Física
Heterossexual	Parda	Catolicismo	Solteiro(a)	Bioquímica
Heterossexual	Branca	Agnosticismo	Solteiro(a)	Arquitetura e Urbanismo
Heterossexual	Branca	Ateísmo	Solteiro(a)	etariado Executivo Trilí
Homossexual	Branca	Ateísmo	Solteiro(a)	Geografia
Heterossexual	Parda	Evangelismo	Casamento/união estáve	Ciências Contábeis
Heterossexual	Branca	Agnosticismo	amoro/em relacionamer	Artes Visuais
Homossexual	Branca	Ateísmo	Solteiro(a)	Ciências Econômicas
Bissexual	Amarela	Agnosticismo	Solteiro(a)	Biomedicina
Pansexual	Branca	Agnosticismo	Solteiro(a)	Matemática
Heterossexual	Parda	Catolicismo	Solteiro(a)	Engenharia Química
Bissexual	Preta	Agnosticismo	Solteiro(a)	Ciências Contábeis
Homossexual	Parda	Ateísmo	Solteiro(a)	Administração
Heterossexual	Branca	Evangelismo	Solteiro(a)	Zootecnia
Heterossexual	Branca	Evangelismo	Solteiro(a)	Enfermagem
Heterossexual	Branca	Agnosticismo	amoro/em relacionamer	Engenharia de Produçã
Bissexual	Branca	Catolicismo	amoro/em relacionamer	Ciências Contábeis
Bissexual	Preta	Agnosticismo	Solteiro(a)	Direito
Heterossexual	Parda	Catolicismo	amoro/em relacionamer	Enfermagem
Bissexual	Amarela	Catolicismo	amoro/em relacionamer	Design
Heterossexual	Branca	Evangelismo	amoro/em relacionamer	Enfermagem
Heterossexual	Branca	Evangelismo	amoro/em relacionamer	Enfermagem
Homossexual	Branca	Agnosticismo	Solteiro(a)	Farmácia
Heterossexual	Amarela	Ateísmo	Solteiro(a)	Farmácia
Bissexual	Parda	Agnosticismo	amoro/em relacionamer	Educação Física
Bissexual	Branca	Catolicismo	Solteiro(a)	Educação Física
Heterossexual	Amarela	Candombleismo	Solteiro(a)	Enfermagem
Homossexual	Branca	Agnosticismo	Solteiro(a)	Enfermagem
Bissexual	Parda	Evangelismo	Solteiro(a)	Enfermagem
Bissexual	Branca	Umbandismo	Solteiro(a)	Ciências Sociais
Heterossexual	Branca	Catolicismo	Casamento/união estáve	Medicina Veterinária
Heterossexual	Parda	Catolicismo	amoro/em relacionamer	Engenharia Elétrica
Heterossexual	Branca	Agnosticismo	Solteiro(a)	Psicologia
Heterossexual	Branca	Evangelismo	Solteiro(a)	Física Médica
Bissexual	Branca	Ateísmo	Solteiro(a)	Ciências Biológicas
Bissexual	Branca	Catolicismo	amoro/em relacionamer	Administração

Heterossexual	Branca	Catolicismo	amoro/em relacionamer	Biomedicina
Heterossexual	Branca	Evangelismo	amoro/em relacionamer	Engenharia de Produçã
Heterossexual	Branca	Agnosticismo	Solteiro(a)	Letras
Homossexual	Branca	Agnosticismo	Solteiro(a)	Física
Heterossexual	Branca	Catolicismo	amoro/em relacionamer	Educação Física
Heterossexual	Branca	Catolicismo	Solteiro(a)	Medicina
Heterossexual	Branca	Catolicismo	Solteiro(a)	Ciências Contábeis
Heterossexual	Branca	Catolicismo	Solteiro(a)	Odontologia
Heterossexual	Branca	Catolicismo	Solteiro(a)	Direito
Bissexual	Preta	Catolicismo	amoro/em relacionamer	Engenharia de Produçã
Bissexual	Branca	Agnosticismo	Solteiro(a)	Ciências Sociais
Homossexual	Branca	Catolicismo	Solteiro(a)	Psicologia
Homossexual	Branca	Agnosticismo	amoro/em relacionamer	Psicologia
Bissexual	Branca	Agnosticismo	Solteiro(a)	Direito
Pansexual	Preta	Ateísmo	Solteiro(a)	Ciências Econômicas
Heterossexual	Parda	Evangelismo	Solteiro(a)	Enfermagem
Heterossexual	Branca	Catolicismo	Solteiro(a)	Física
Bissexual	Branca	Espiritismo	amoro/em relacionamer	Medicina Veterinária
Bissexual	Branca	Catolicismo	amoro/em relacionamer	Pedagogia
Heterossexual	Branca	Adventistismo	amoro/em relacionamer	Engenharia têxtil
Heterossexual	Branca	Agnosticismo	amoro/em relacionamer	Física
Heterossexual	Branca	Agnosticismo	Solteiro(a)	Informática
Homossexual	Branca	Catolicismo	Solteiro(a)	Administração
Heterossexual	Branca	Catolicismo	Solteiro(a)	Engenharia Civil
Bissexual	Branca	Catolicismo	Solteiro(a)	Direito
Heterossexual	Parda	Catolicismo	Solteiro(a)	Farmácia
Heterossexual	Branca	Catolicismo	Solteiro(a)	Biomedicina
Heterossexual	Branca	Catolicismo	Solteiro(a)	Medicina
Bissexual	Parda	Catolicismo	amoro/em relacionamer	etariado Executivo Trilí
Heterossexual	Parda	Catolicismo	Solteiro(a)	Medicina Veterinária
Heterossexual	Branca	Catolicismo	amoro/em relacionamer	Física médica
Bissexual	Branca	Agnosticismo	Solteiro(a)	Psicologia
Heterossexual	Branca	Ateísmo	Solteiro(a)	Letras
Heterossexual	Branca	Evangelismo	amoro/em relacionamer	Pedagogia
Heterossexual	Branca	Catolicismo	Casamento/união estáve	Administração
Heterossexual	Branca	Catolicismo	amoro/em relacionamer	Administração
Heterossexual	Parda	Evangelismo	Casamento/união estáve	Pedagogia
Homossexual	Parda	Evangelismo	Solteiro(a)	Pedagogia
Heterossexual	Parda	Catolicismo	Solteiro(a)	Química
Bissexual	Branca	Agnosticismo	Casamento/união estáve	Medicina
Bissexual	Branca	Ateísmo	Solteiro(a)	Biomedicina
Heterossexual	Branca	Ateísmo	Solteiro(a)	Pedagogia
Heterossexual	Branca	Catolicismo	amoro/em relacionamer	cnologia em Biotecnolo
Bissexual	Preta	Catolicismo	amoro/em relacionamer	Ciências Sociais
Pansexual	Preta	Agnosticismo	Casamento/união estáve	Física
Heterossexual	Branca	Catolicismo	Solteiro(a)	Artes Cênicas
Bissexual	Parda	Agnosticismo	Solteiro(a)	Biomedicina
Homossexual	Preta	Catolicismo	Solteiro(a)	Música
Bissexual	Parda	Catolicismo	Casamento/união estáve	nologia em Gestão Púb
Heterossexual	Preta	Evangelismo	Solteiro(a)	Ciências Biológicas
Bissexual	Parda	Catolicismo	Casamento/união estáve	Ciências Biológicas
Heterossexual	Branca	Catolicismo	amoro/em relacionamer	Medicina
Heterossexual	Branca	Catolicismo	amoro/em relacionamer	Psicologia
Heterossexual	Branca	Evangelismo	Solteiro(a)	Engenharia Mecânica

Heterossexual	Branca	Catolicismo	amoro/em relacionamer	Medicina
Heterossexual	Branca	Catolicismo	Solteiro(a)	Odontologia
Heterossexual	Branca	Ateísmo	amoro/em relacionamer	Ciências Biológicas
Heterossexual	Parda	Evangelismo	Solteiro(a)	Ciências Contábeis
Homossexual	Branca	Agnosticismo	Casamento/união estáve	Direito
Homossexual	Branca	Catolicismo	amoro/em relacionamer	Ciências Biológicas
Homossexual	Branca	Agnosticismo	Solteiro(a)	Biotecnologia
Heterossexual	Branca	Ateísmo	Solteiro(a)	Administração
Bissexual	Branca	Ateísmo	Solteiro(a)	Pedagogia
Heterossexual	Branca	Catolicismo	Solteiro(a)	Ciências Econômicas
Bissexual	Branca	Ateísmo	Solteiro(a)	Administração
Heterossexual	Branca	Catolicismo	Separação/divórcio	História
Heterossexual	Branca	Catolicismo	Casamento/união estáve	Pedagogia
Homossexual	Branca	Agnosticismo	Solteiro(a)	Letras
Homossexual	Branca	Umbandismo	Solteiro(a)	Engenharia de Alimento
Heterossexual	Parda	Evangelismo	Solteiro(a)	Medicina
Heterossexual	Branca	Espiritismo	Solteiro(a)	Medicina
Homossexual	Branca	Ateísmo	amoro/em relacionamer	Física
Heterossexual	Branca	Catolicismo	Solteiro(a)	Pedagogia
Heterossexual	Amarela	Ateísmo	amoro/em relacionamer	Letras
Pansexual	Branca	Catolicismo	Solteiro(a)	Engenharia Civil
Heterossexual	Branca	Evangelismo	Casamento/união estáve	Engenharia de Alimento
Bissexual	Preta	Agnosticismo	amoro/em relacionamer	Geografia
Heterossexual	Parda	Catolicismo	Casamento/união estáve	Geografia
Heterossexual	Branca	Catolicismo	Solteiro(a)	Química
Homossexual	Parda	Evangelismo	Casamento/união estáve	Pedagogia
Homossexual	Branca	Agnosticismo	Solteiro(a)	Direito
Bissexual	Parda	Catolicismo	Casamento/união estáve	Administração
Heterossexual	Branca	Catolicismo	Casamento/união estáve	Ciências Contábeis
Homossexual	Branca	Catolicismo	Separação/divórcio	Matemática
Heterossexual	Branca	Ateísmo	amoro/em relacionamer	Ciências Sociais
Heterossexual	Branca	Catolicismo	Casamento/união estáve	Pedagogia
Heterossexual	Branca	Catolicismo	Casamento/união estáve	Direito
Homossexual	Branca	Catolicismo	Solteiro(a)	Ciências Contábeis
Homossexual	Preta	Catolicismo	amoro/em relacionamer	Geografia
Heterossexual	Branca	Ateísmo	amoro/em relacionamer	Enfermagem
Heterossexual	Branca	Ateísmo	amoro/em relacionamer	Direito
Heterossexual	Branca	Catolicismo	Solteiro(a)	Administração
Heterossexual	Branca	Catolicismo	Casamento/união estáve	ologia em Construção (
Homossexual	Branca	Catolicismo	Solteiro(a)	História
Bissexual	Branca	Catolicismo	amoro/em relacionamer	Física
Homossexual	Branca	Evangelismo	Casamento/união estáve	Artes Visuais
Heterossexual	Parda	Catolicismo	amoro/em relacionamer	Geografia
Bissexual	Branca	Ateísmo	amoro/em relacionamer	Pedagogia
Heterossexual	Parda	Evangelismo	Separação/divórcio	Geografia
Bissexual	Branca	Agnosticismo	Casamento/união estáve	Ciências Contábeis
Heterossexual	Parda	Agnosticismo	Solteiro(a)	Direito
Heterossexual	Branca	Ateísmo	Casamento/união estáve	História
Bissexual	Branca	Catolicismo	Solteiro(a)	Estatística
Heterossexual	Parda	Espiritismo	amoro/em relacionamer	Pedagogia
Heterossexual	Branca	Agnosticismo	amoro/em relacionamer	Engenharia Química
Heterossexual	Branca	Catolicismo	Casamento/união estáve	Ciências Contábeis
Heterossexual	Branca	Espiritismo	Casamento/união estáve	etariado Executivo Trilí
Heterossexual	Branca	Agnosticismo	Solteiro(a)	Historia

Heterossexual	Branca	Evangelismo	:asamento/união estáve	Pedagogia
Heterossexual	Parda	Evangelismo	:asamento/união estáve	Tècnologia em Alimento
Homossexual	Parda	Catolicismo	:asamento/união estáve	nologia em Meio Ambie
Heterossexual	Branca	Evangelismo	:asamento/união estáve	Música
Heterossexual	Parda	Evangelismo	Solteiro(a)	Química
Pansexual	Branca	Agnosticismo	Solteiro(a)	Pedagogia
Heterossexual	Branca	Catolicismo	Separação/divórcio	Pedagogia
Heterossexual	Branca	Catolicismo	:asamento/união estáve	Pedagogia
Heterossexual	Preta	Evangelismo	amoro/em relacionamer	Geografia
Heterossexual	Preta	Catolicismo	:asamento/união estáve	Ciências Sociais
Homossexual	Branca	Agnosticismo	Solteiro(a)	Medicina
Heterossexual	Branca	Evangelismo	Solteiro(a)	Ciências Econômicas
Heterossexual	Preta	Catolicismo	:asamento/união estáve	Psicologia
Heterossexual	Parda	Catolicismo	Solteiro(a)	Administração
Homossexual	Branca	Catolicismo	amoro/em relacionamer	Pedagogia
Heterossexual	Parda	Catolicismo	Solteiro(a)	Física
Homossexual	Branca	Catolicismo	Solteiro(a)	Pedagogia
Heterossexual	Branca	Catolicismo	Solteiro(a)	Ciências Contábeis

Centro	Campus	TempoUEM	RendaFamiliar	HistóricoRelaçãoSexua
o de Ciências Agrárias (	Maringá	< 1 ano	R$5.281 - R$6.600	Sim
Ciências Sociais Aplica	Maringá	< 1 ano	R$3.961 - R$5.280	Sim
ncias Humanas, Letras	Maringá	1 a 2 anos	R$3.961 - R$5.280	Sim
de Ciências Biológicas	Maringá	1 a 2 anos	R$7.921 ou mais	Não
ncias Humanas, Letras	Maringá	< 1 ano	R$6.601 - R$7.920	Sim
Ciências Sociais Aplica	Maringá	< 1 ano	R$7.921 ou mais	Sim
ro de Ciências Exatas (	Maringá	< 1 ano	R$3.961 - R$5.280	Não
de Ciências da Saúde	Maringá	3 a 5 anos	R$1.321 - R$2.640	Sim
de Ciências da Saúde	Maringá	3 a 5 anos	R$7.921 ou mais	Sim
de Ciências da Saúde	Maringá	3 a 5 anos	R$6.601 - R$7.920	Sim
ncias Humanas, Letras	Maringá	1 a 2 anos	R$3.961 - R$5.280	Sim
ncias Humanas, Letras	Maringá	1 a 2 anos	R$7.921 ou mais	Sim
ncias Humanas, Letras	Maringá	1 a 2 anos	R$2.641 - R$3.960	Sim
de Ciências da Saúde	Maringá	1 a 2 anos	R$5.281 - R$6.600	Sim
ncias Humanas, Letras	Maringá	3 a 5 anos	R$7.921 ou mais	Sim
Ciências Sociais Aplica	Maringá	< 1 ano	R$3.961 - R$5.280	Sim
de Ciências da Saúde	Maringá	3 a 5 anos	R$6.601 - R$7.920	Sim
ncias Humanas, Letras	Maringá	3 a 5 anos	R$7.921 ou mais	Não
Ciências Sociais Aplica	Maringá	1 a 2 anos	R$1.321 - R$2.640	Sim
de Ciências Biológicas	Maringá	3 a 5 anos	R$3.961 - R$5.280	Sim
de Ciências da Saúde	Maringá	1 a 2 anos	R$5.281 - R$6.600	Sim
de Ciências da Saúde	Maringá	1 a 2 anos	R$6.601 - R$7.920	Sim
de Ciências da Saúde	Maringá	3 a 5 anos	R$5.281 - R$6.600	Sim
de Ciências da Saúde	Maringá	3 a 5 anos	R$2.641 - R$3.960	Sim
de Ciências da Saúde	Maringá	1 a 2 anos	R$3.961 - R$5.280	Sim
de Ciências da Saúde	Maringá	3 a 5 anos	R$2.641 - R$3.960	Sim
de Ciências Biológicas	Maringá	3 a 5 anos	R$5.281 - R$6.600	Sim
de Ciências Biológicas	Maringá	< 1 ano	R$1.321 - R$2.640	Sim
de Ciências da Saúde	Maringá	1 a 2 anos	R$5.281 - R$6.600	Sim
de Ciências da Saúde	Maringá	3 a 5 anos	R$7.921 ou mais	Não
de Ciências Biológicas	Maringá	3 a 5 anos	R$2.641 - R$3.960	Sim
de Ciências da Saúde	Maringá	1 a 2 anos	R$2.641 - R$3.960	Não
de Ciências da Saúde	Maringá	1 a 2 anos	R$7.921 ou mais	Sim
de Ciências Biológicas	Maringá	3 a 5 anos	R$2.641 - R$3.960	Não
de Ciências da Saúde	Maringá	1 a 2 anos	R$2.641 - R$3.960	Sim
ncias Humanas, Letras	Maringá	3 a 5 anos	R$6.601 - R$7.920	Sim
ncias Humanas, Letras	Maringá	1 a 2 anos	R$7.921 ou mais	Não
de Ciências da Saúde	Maringá	3 a 5 anos	R$3.961 - R$5.280	Sim
de Ciências da Saúde	Maringá	3 a 5 anos	R$7.921 ou mais	Sim
ncias Humanas, Letras	Maringá	3 a 5 anos	R$1.321 - R$2.640	Sim
de Ciências da Saúde	Maringá	1 a 2 anos	R$7.921 ou mais	Sim
Ciências Sociais Aplica	Maringá	1 a 2 anos	R$5.281 - R$6.600	Não
entro de Tecnologia (CT	Maringá	< 1 ano	R$6.601 - R$7.920	Não
Ciências Sociais Aplica	Maringá	1 a 2 anos	R$1.321 - R$2.640	Sim
de Ciências Biológicas	Maringá	< 1 ano	R$0 - R$1.320	Sim
de Ciências da Saúde	Maringá	1 a 2 anos	R$2.641 - R$3.960	Sim
de Ciências da Saúde	Maringá	1 a 2 anos	R$7.921 ou mais	Não
de Ciências da Saúde	Maringá	1 a 2 anos	R$5.281 - R$6.600	Não
de Ciências da Saúde	Maringá	3 a 5 anos	R$3.961 - R$5.280	Sim
Ciências Sociais Aplica	Maringá	3 a 5 anos	R$6.601 - R$7.920	Não
ncias Humanas, Letras	Maringá	< 1 ano	R$7.921 ou mais	Não
de Ciências da Saúde	Maringá	1 a 2 anos	R$7.921 ou mais	Não
entro de Tecnologia (CT	Maringá	3 a 5 anos	R$7.921 ou mais	Sim

de Ciências Biológicas	Maringá	1 a 2 anos	R$6.601 - R$7.920	Sim
ncias Humanas, Letras	Maringá	< 1 ano	R$1.321 - R$2.640	Não
ncias Humanas, Letras	Maringá	< 1 ano	R$7.921 ou mais	Sim
ro de Ciências Exatas (	Maringá	3 a 5 anos	R$7.921 ou mais	Não
ncias Humanas, Letras	Maringá	1 a 2 anos	R$7.921 ou mais	Sim
de Ciências da Saúde	Maringá	3 a 5 anos	R$7.921 ou mais	Sim
ro de Ciências Exatas (	Goioerê	1 a 2 anos	R$3.961 - R$5.280	Não
ro de Ciências Exatas (	Maringá	3 a 5 anos	R$7.921 ou mais	Sim
de Ciências da Saúde	Maringá	3 a 5 anos	R$2.641 - R$3.960	Sim
Ciências Sociais Aplica	Maringá	1 a 2 anos	R$7.921 ou mais	Sim
de Ciências da Saúde	Maringá	1 a 2 anos	R$7.921 ou mais	Sim
de Ciências da Saúde	Maringá	3 a 5 anos	R$2.641 - R$3.960	Sim
ncias Humanas, Letras	Maringá	1 a 2 anos	R$0 - R$1.320	Não
de Ciências da Saúde	Maringá	1 a 2 anos	R$2.641 - R$3.960	Sim
ncias Humanas, Letras	Maringá	< 1 ano	R$1.321 - R$2.640	Não
de Ciências da Saúde	Maringá	< 1 ano	R$3.961 - R$5.280	Sim
de Ciências da Saúde	Maringá	3 a 5 anos	R$7.921 ou mais	Não
ncias Humanas, Letras	Maringá	< 1 ano	R$6.601 - R$7.920	Não
ncias Humanas, Letras	Maringá	1 a 2 anos	R$0 - R$1.320	Sim
de Ciências da Saúde	Maringá	< 1 ano	R$3.961 - R$5.280	Sim
de Ciências da Saúde	Maringá	< 1 ano	R$1.321 - R$2.640	Sim
de Ciências da Saúde	Maringá	1 a 2 anos	R$7.921 ou mais	Sim
ncias Humanas, Letras	Maringá	1 a 2 anos	R$6.601 - R$7.920	Não
de Ciências da Saúde	Maringá	3 a 5 anos	R$5.281 - R$6.600	Sim
de Ciências da Saúde	Maringá	3 a 5 anos	R$2.641 - R$3.960	Sim
de Ciências da Saúde	Maringá	1 a 2 anos	R$1.321 - R$2.640	Não
de Ciências da Saúde	Maringá	1 a 2 anos	R$7.921 ou mais	Sim
ncias Humanas, Letras	Maringá	< 1 ano	R$6.601 - R$7.920	Sim
ntro de Tecnologia (CT	Umuarama	< 1 ano	R$0 - R$1.320	Sim
de Ciências da Saúde	Maringá	< 1 ano	R$3.961 - R$5.280	Não
Ciências Sociais Aplica	Maringá	1 a 2 anos	R$2.641 - R$3.960	Sim
de Ciências Biológicas	Maringá	< 1 ano	R$3.961 - R$5.280	Não
ncias Humanas, Letras	Cianorte	1 a 2 anos	R$2.641 - R$3.960	Não
ncias Humanas, Letras	Maringá	< 1 ano	R$2.641 - R$3.960	Sim
ro de Ciências Exatas (	Maringá	< 1 ano	R$5.281 - R$6.600	Não
Ciências Sociais Aplica	Maringá	< 1 ano	R$1.321 - R$2.640	Sim
ncias Humanas, Letras	Maringá	1 a 2 anos	R$6.601 - R$7.920	Não
ncias Humanas, Letras	Maringá	1 a 2 anos	R$6.601 - R$7.920	Sim
ntro de Tecnologia (CT	Maringá	< 1 ano	R$7.921 ou mais	Sim
Ciências Sociais Aplica	Maringá	1 a 2 anos	R$2.641 - R$3.960	Não
Ciências Sociais Aplica	Maringá	3 a 5 anos	R$2.641 - R$3.960	Sim
o de Ciências Agrárias (	Maringá	3 a 5 anos	R$6.601 - R$7.920	Sim
de Ciências da Saúde	Maringá	< 1 ano	R$7.921 ou mais	Sim
ntro de Tecnologia (CT	Maringá	3 a 5 anos	R$7.921 ou mais	Não
Ciências Sociais Aplica	Maringá	3 a 5 anos	R$5.281 - R$6.600	Não
Ciências Sociais Aplica	Maringá	1 a 2 anos	R$3.961 - R$5.280	Sim
ncias Humanas, Letras	Maringá	3 a 5 anos	R$0 - R$1.320	Sim
ntro de Tecnologia (CT	Maringá	3 a 5 anos	R$7.921 ou mais	Sim
ncias Humanas, Letras	Maringá	1 a 2 anos	R$7.921 ou mais	Sim
ncias Humanas, Letras	Maringá	3 a 5 anos	R$3.961 - R$5.280	Sim
Ciências Sociais Aplica	Cianorte	3 a 5 anos	R$3.961 - R$5.280	Não
ncias Humanas, Letras	Maringá	3 a 5 anos	R$2.641 - R$3.960	Não
ncias Humanas, Letras	Maringá	1 a 2 anos	R$5.281 - R$6.600	Sim
ncias Humanas, Letras	Maringá	1 a 2 anos	R$5.281 - R$6.600	Sim

› de Ciências da Saúde	Maringá	1 a 2 anos	R$7.921 ou mais	Sim
:ntro de Tecnologia (CT	Maringá	3 a 5 anos	R$2.641 - R$3.960	Sim
de Ciências Biológicas	Maringá	1 a 2 anos	R$3.961 - R$5.280	Não
ro de Ciências Exatas (	Maringá	3 a 5 anos	R$6.601 - R$7.920	Não
ro de Ciências Exatas (	Maringá	3 a 5 anos	R$5.281 - R$6.600	Sim
› de Ciências da Saúde	Maringá	1 a 2 anos	R$1.321 - R$2.640	Não
o de Ciências Agrárias (	Umuarama	1 a 2 anos	R$1.321 - R$2.640	Sim
Ciências Sociais Aplica	Cianorte	3 a 5 anos	R$2.641 - R$3.960	Sim
ncias Humanas, Letras	Maringá	1 a 2 anos	R$6.601 - R$7.920	Não
ro de Ciências Exatas (	Cianorte	1 a 2 anos	R$1.321 - R$2.640	Sim
ncias Humanas, Letras	Maringá	3 a 5 anos	R$1.321 - R$2.640	Sim
:ntro de Tecnologia (CT	Umuarama	1 a 2 anos	R$6.601 - R$7.920	Sim
ncias Humanas, Letras	Maringá	3 a 5 anos	R$6.601 - R$7.920	Sim
ncias Humanas, Letras	Maringá	1 a 2 anos	R$2.641 - R$3.960	Sim
Ciências Sociais Aplica	Maringá	1 a 2 anos	R$7.921 ou mais	Não
ncias Humanas, Letras	Maringá	1 a 2 anos	R$6.601 - R$7.920	Não
ro de Ciências Exatas (	Maringá	3 a 5 anos	R$7.921 ou mais	Sim
ro de Ciências Exatas (	Goioerê	3 a 5 anos	R$5.281 - R$6.600	Não
ro de Ciências Exatas (	Maringá	3 a 5 anos	R$2.641 - R$3.960	Não
ncias Humanas, Letras	Maringá	1 a 2 anos	R$1.321 - R$2.640	Sim
ncias Humanas, Letras	Maringá	1 a 2 anos	R$3.961 - R$5.280	Não
ncias Humanas, Letras	Maringá	< 1 ano	R$7.921 ou mais	Sim
ncias Humanas, Letras	Maringá	3 a 5 anos	R$3.961 - R$5.280	Sim
o de Ciências Agrárias (	Maringá	3 a 5 anos	R$2.641 - R$3.960	Sim
ro de Ciências Exatas (	Maringá	3 a 5 anos	R$3.961 - R$5.280	Sim
Ciências Sociais Aplica	Maringá	1 a 2 anos	R$7.921 ou mais	Não
Ciências Sociais Aplica	Maringá	1 a 2 anos	R$2.641 - R$3.960	Sim
o de Ciências Agrárias (	Maringá	1 a 2 anos	R$1.321 - R$2.640	Não
ncias Humanas, Letras	Maringá	3 a 5 anos	R$1.321 - R$2.640	Sim
Ciências Sociais Aplica	Maringá	< 1 ano	R$3.961 - R$5.280	Sim
Ciências Sociais Aplica	Maringá	3 a 5 anos	R$7.921 ou mais	Sim
ro de Ciências Exatas (	Maringá	1 a 2 anos	R$2.641 - R$3.960	Sim
de Ciências Biológicas	Maringá	3 a 5 anos	R$6.601 - R$7.920	Não
ncias Humanas, Letras	Maringá	1 a 2 anos	R$7.921 ou mais	Sim
de Ciências Biológicas	Maringá	3 a 5 anos	R$7.921 ou mais	Não
ro de Ciências Exatas (	Maringá	1 a 2 anos	R$2.641 - R$3.960	Sim
ro de Ciências Exatas (	Maringá	3 a 5 anos	R$2.641 - R$3.960	Sim
› de Ciências da Saúde	Maringá	1 a 2 anos	R$3.961 - R$5.280	Não
:ntro de Tecnologia (CT	Maringá	1 a 2 anos	R$5.281 - R$6.600	Sim
:ntro de Tecnologia (CT	Maringá	3 a 5 anos	R$7.921 ou mais	Não
Ciências Sociais Aplica	Maringá	3 a 5 anos	R$5.281 - R$6.600	Sim
ro de Ciências Exatas (	Maringá	3 a 5 anos	R$3.961 - R$5.280	Sim
ncias Humanas, Letras	Maringá	< 1 ano	R$5.281 - R$6.600	Sim
:ntro de Tecnologia (CT	Maringá	1 a 2 anos	R$0 - R$1.320	Sim
› de Ciências da Saúde	Maringá	3 a 5 anos	R$7.921 ou mais	Sim
o de Ciências Agrárias (	Maringá	1 a 2 anos	R$2.641 - R$3.960	Não
› de Ciências da Saúde	Maringá	1 a 2 anos	R$3.961 - R$5.280	Sim
ncias Humanas, Letras	Maringá	3 a 5 anos	R$2.641 - R$3.960	Sim
ncias Humanas, Letras	Maringá	3 a 5 anos	R$6.601 - R$7.920	Sim
Ciências Sociais Aplica	Maringá	1 a 2 anos	R$2.641 - R$3.960	Sim
de Ciências Biológicas	Maringá	3 a 5 anos	R$0 - R$1.320	Não
› de Ciências da Saúde	Maringá	< 1 ano	R$1.321 - R$2.640	Sim
ro de Ciências Exatas (	Cianorte	3 a 5 anos	R$3.961 - R$5.280	Não
› de Ciências da Saúde	Maringá	3 a 5 anos	R$7.921 ou mais	Sim

ɜntro de Tecnologia (CT	Maringá	1 a 2 anos	R$7.921 ou mais	Sim
ɔ de Ciências Agrárias (	Maringá	3 a 5 anos	R$7.921 ou mais	Sim
ro de Ciências Exatas ((	Maringá	3 a 5 anos	R$5.281 - R$6.600	Sim
) de Ciências da Saúde	Maringá	1 a 2 anos	R$2.641 - R$3.960	Não
ncias Humanas, Letras	Maringá	1 a 2 anos	R$3.961 - R$5.280	Sim
ɜntro de Tecnologia (CT	Maringá	3 a 5 anos	R$6.601 - R$7.920	Sim
ɔ de Ciências Agrárias (	Maringá	1 a 2 anos	R$2.641 - R$3.960	Não
Ciências Sociais Aplica	Maringá	< 1 ano	R$2.641 - R$3.960	Sim
ncias Humanas, Letras	Maringá	3 a 5 anos	R$1.321 - R$2.640	Sim
ro de Ciências Exatas ((	Maringá	1 a 2 anos	R$7.921 ou mais	Sim
ɜntro de Tecnologia (CT	Umuarama	< 1 ano	R$5.281 - R$6.600	Sim
ncias Humanas, Letras	Maringá	3 a 5 anos	R$5.281 - R$6.600	Sim
Ciências Sociais Aplica	Maringá	1 a 2 anos	R$2.641 - R$3.960	Não
Ciências Sociais Aplica	Maringá	3 a 5 anos	R$7.921 ou mais	Sim
ncias Humanas, Letras	Maringá	3 a 5 anos	R$3.961 - R$5.280	Sim
ɜntro de Tecnologia (CT	Maringá	3 a 5 anos	R$2.641 - R$3.960	Não
Ciências Sociais Aplica	Maringá	< 1 ano	R$1.321 - R$2.640	Sim
ncias Humanas, Letras	Ivaiporã	1 a 2 anos	R$2.641 - R$3.960	Não
ɜntro de Tecnologia (CT	Maringá	1 a 2 anos	R$7.921 ou mais	Sim
ncias Humanas, Letras	Maringá	1 a 2 anos	R$7.921 ou mais	Sim
de Ciências Biológicas	Maringá	3 a 5 anos	R$7.921 ou mais	Sim
ɔ de Ciências Agrárias (	Maringá	3 a 5 anos	R$7.921 ou mais	Sim
ncias Humanas, Letras	Maringá	1 a 2 anos	R$3.961 - R$5.280	Sim
) de Ciências da Saúde	Maringá	1 a 2 anos	R$6.601 - R$7.920	Não
) de Ciências da Saúde	Maringá	3 a 5 anos	R$6.601 - R$7.920	Sim
Ciências Sociais Aplica	Ivaiporã	3 a 5 anos	R$3.961 - R$5.280	Sim
ɜntro de Tecnologia (CT	Maringá	3 a 5 anos	R$7.921 ou mais	Sim
ncias Humanas, Letras	Maringá	< 1 ano	R$7.921 ou mais	Não
) de Ciências da Saúde	Maringá	3 a 5 anos	R$7.921 ou mais	Não
ɔ de Ciências Agrárias (	Maringá	1 a 2 anos	R$6.601 - R$7.920	Não
) de Ciências da Saúde	Maringá	1 a 2 anos	R$0 - R$1.320	Não
ɜntro de Tecnologia (CT	Maringá	3 a 5 anos	R$1.321 - R$2.640	Sim
) de Ciências da Saúde	Maringá	1 a 2 anos	R$7.921 ou mais	Não
ncias Humanas, Letras	Umuarama	< 1 ano	R$1.321 - R$2.640	Sim
ɔ de Ciências Agrárias (	Umuarama	1 a 2 anos	R$1.321 - R$2.640	Sim
Ciências Sociais Aplica	Ivaiporã	1 a 2 anos	R$5.281 - R$6.600	Sim
) de Ciências da Saúde	Umuarama	< 1 ano	R$1.321 - R$2.640	Sim
ncias Humanas, Letras	Maringá	3 a 5 anos	R$2.641 - R$3.960	Sim
ɔ de Ciências Agrárias (	Umuarama	< 1 ano	R$5.281 - R$6.600	Não
) de Ciências da Saúde	Maringá	1 a 2 anos	R$1.321 - R$2.640	Sim
) de Ciências da Saúde	Maringá	1 a 2 anos	R$3.961 - R$5.280	Não
ncias Humanas, Letras	Maringá	< 1 ano	R$2.641 - R$3.960	Não
ro de Ciências Exatas ((	Maringá	3 a 5 anos	R$6.601 - R$7.920	Sim
ɜntro de Tecnologia (CT	Maringá	1 a 2 anos	R$2.641 - R$3.960	Não
de Ciências Biológicas	Maringá	3 a 5 anos	R$3.961 - R$5.280	Sim
ncias Humanas, Letras	Maringá	1 a 2 anos	R$7.921 ou mais	Sim
ncias Humanas, Letras	Maringá	3 a 5 anos	R$7.921 ou mais	Sim
Ciências Sociais Aplica	Maringá	3 a 5 anos	R$1.321 - R$2.640	Sim
ncias Humanas, Letras	Maringá	< 1 ano	R$2.641 - R$3.960	Não
ɜntro de Tecnologia (CT	Maringá	1 a 2 anos	R$7.921 ou mais	Não
ncias Humanas, Letras	Maringá	1 a 2 anos	R$1.321 - R$2.640	Sim
Ciências Sociais Aplica	Maringá	3 a 5 anos	R$7.921 ou mais	Sim
ɜntro de Tecnologia (CT	Maringá	< 1 ano	R$7.921 ou mais	Não
ncias Humanas, Letras	Cianorte	1 a 2 anos	R$1.321 - R$2.640	Sim

ntro de Tecnologia (CT	Maringá	1 a 2 anos	R$7.921 ou mais	Sim
Ciências Sociais Aplica	Maringá	3 a 5 anos	R$3.961 - R$5.280	Sim
o de Ciências Exatas (	Maringá	3 a 5 anos	R$1.321 - R$2.640	Sim
ncias Humanas, Letras	Maringá	3 a 5 anos	R$6.601 - R$7.920	Não
ncias Humanas, Letras	Maringá	1 a 2 anos	R$2.641 - R$3.960	Sim
o de Ciências Exatas (	Goioerê	1 a 2 anos	R$5.281 - R$6.600	Não
Ciências Sociais Aplica	Cianorte	1 a 2 anos	R$3.961 - R$5.280	Sim
ncias Humanas, Letras	Maringá	1 a 2 anos	R$2.641 - R$3.960	Não
ncias Humanas, Letras	Maringá	3 a 5 anos	R$7.921 ou mais	Não
ncias Humanas, Letras	Maringá	1 a 2 anos	R$0 - R$1.320	Sim
o de Ciências Exatas (	Maringá	1 a 2 anos	R$3.961 - R$5.280	Não
Ciências Sociais Aplica	Maringá	1 a 2 anos	R$5.281 - R$6.600	Sim
o de Ciências Exatas (	Maringá	3 a 5 anos	R$6.601 - R$7.920	Não
ncias Humanas, Letras	Maringá	1 a 2 anos	R$2.641 - R$3.960	Sim
ncias Humanas, Letras	Maringá	1 a 2 anos	R$7.921 ou mais	Não
o de Ciências Exatas (	Maringá	< 1 ano	R$7.921 ou mais	Sim
ntro de Tecnologia (CT	Maringá	1 a 2 anos	R$7.921 ou mais	Sim
ncias Humanas, Letras	Maringá	1 a 2 anos	R$3.961 - R$5.280	Sim
de Ciências da Saúde	Maringá	1 a 2 anos	R$1.321 - R$2.640	Sim
Ciências Sociais Aplica	Maringá	3 a 5 anos	R$5.281 - R$6.600	Sim
Ciências Sociais Aplica	Maringá	1 a 2 anos	R$2.641 - R$3.960	Não
o de Ciências Exatas (	Maringá	3 a 5 anos	R$7.921 ou mais	Sim
de Ciências da Saúde	Maringá	1 a 2 anos	R$7.921 ou mais	Sim
o de Ciências Exatas (	Maringá	3 a 5 anos	R$2.641 - R$3.960	Sim
ncias Humanas, Letras	Maringá	1 a 2 anos	R$3.961 - R$5.280	Sim
de Ciências Biológicas	Maringá	1 a 2 anos	R$2.641 - R$3.960	Não
ntro de Tecnologia (CT	Goioerê	3 a 5 anos	R$2.641 - R$3.960	Não
ntro de Tecnologia (CT	Umuarama	1 a 2 anos	R$6.601 - R$7.920	Sim
Ciências Sociais Aplica	Maringá	1 a 2 anos	R$7.921 ou mais	Sim
ntro de Tecnologia (CT	Maringá	1 a 2 anos	R$6.601 - R$7.920	Não
o de Ciências Exatas (	Maringá	3 a 5 anos	R$3.961 - R$5.280	Sim
ncias Humanas, Letras	Maringá	1 a 2 anos	R$1.321 - R$2.640	Sim
ncias Humanas, Letras	Maringá	< 1 ano	R$1.321 - R$2.640	Não
de Ciências da Saúde	Maringá	1 a 2 anos	R$3.961 - R$5.280	Sim
de Ciências da Saúde	Maringá	1 a 2 anos	R$5.281 - R$6.600	Sim
de Ciências Biológicas	Maringá	3 a 5 anos	R$1.321 - R$2.640	Sim
Ciências Sociais Aplica	Maringá	1 a 2 anos	R$7.921 ou mais	Sim
o de Ciências Exatas (	Maringá	3 a 5 anos	R$1.321 - R$2.640	Sim
Ciências Sociais Aplica	Maringá	1 a 2 anos	R$7.921 ou mais	Sim
Ciências Sociais Aplica	Maringá	3 a 5 anos	R$7.921 ou mais	Não
o de Ciências Exatas (	Maringá	3 a 5 anos	R$0 - R$1.320	Sim
de Ciências da Saúde	Maringá	3 a 5 anos	R$7.921 ou mais	Sim
ncias Humanas, Letras	Maringá	3 a 5 anos	R$5.281 - R$6.600	Sim
Ciências Sociais Aplica	Maringá	< 1 ano	R$5.281 - R$6.600	Sim
de Ciências da Saúde	Maringá	3 a 5 anos	R$2.641 - R$3.960	Sim
de Ciências Biológicas	Maringá	3 a 5 anos	R$1.321 - R$2.640	Sim
ncias Humanas, Letras	Cianorte	< 1 ano	R$3.961 - R$5.280	Não
ntro de Tecnologia (CT	Maringá	1 a 2 anos	R$5.281 - R$6.600	Sim
o de Ciências Exatas (	Maringá	< 1 ano	R$7.921 ou mais	Não
o de Ciências Exatas (	Goioerê	< 1 ano	R$0 - R$1.320	Sim
de Ciências Biológicas	Maringá	1 a 2 anos	R$3.961 - R$5.280	Não
o de Ciências Exatas (	Maringá	< 1 ano	R$1.321 - R$2.640	Não
de Ciências da Saúde	Maringá	3 a 5 anos	R$7.921 ou mais	Sim
de Ciências Biológicas	Maringá	1 a 2 anos	R$7.921 ou mais	Sim

ncias Humanas, Letras	Maringá	3 a 5 anos	R$1.321 - R$2.640	Sim
ro de Ciências Exatas (	Maringá	3 a 5 anos	R$1.321 - R$2.640	Sim
de Ciências da Saúde	Maringá	1 a 2 anos	R$3.961 - R$5.280	Sim
entro de Tecnologia (CT	Umuarama	3 a 5 anos	R$1.321 - R$2.640	Sim
ncias Humanas, Letras	Maringá	< 1 ano	R$2.641 - R$3.960	Sim
entro de Tecnologia (CT	Maringá	3 a 5 anos	R$5.281 - R$6.600	Sim
ro de Ciências Exatas (	Maringá	> 5 anos	R$7.921 ou mais	Sim
ncias Humanas, Letras	Maringá	3 a 5 anos	R$3.961 - R$5.280	Sim
ro de Ciências Exatas (	Maringá	3 a 5 anos	R$3.961 - R$5.280	Sim
de Ciências da Saúde	Maringá	1 a 2 anos	R$2.641 - R$3.960	Não
ncias Humanas, Letras	Maringá	3 a 5 anos	R$5.281 - R$6.600	Não
ncias Humanas, Letras	Maringá	3 a 5 anos	R$1.321 - R$2.640	Sim
de Ciências da Saúde	Maringá	1 a 2 anos	R$3.961 - R$5.280	Sim
ncias Humanas, Letras	Maringá	< 1 ano	R$2.641 - R$3.960	Sim
de Ciências da Saúde	Maringá	1 a 2 anos	R$6.601 - R$7.920	Não
de Ciências Biológicas	Maringá	3 a 5 anos	R$2.641 - R$3.960	Sim
Ciências Sociais Aplica	Maringá	3 a 5 anos	R$7.921 ou mais	Sim
de Ciências da Saúde	Maringá	1 a 2 anos	R$5.281 - R$6.600	Não
ncias Humanas, Letras	Maringá	3 a 5 anos	R$2.641 - R$3.960	Sim
entro de Tecnologia (CT	Maringá	3 a 5 anos	R$1.321 - R$2.640	Sim
o de Ciências Agrárias (	Maringá	< 1 ano	R$1.321 - R$2.640	Sim
ro de Ciências Exatas (	Maringá	3 a 5 anos	R$6.601 - R$7.920	Sim
de Ciências da Saúde	Maringá	3 a 5 anos	R$3.961 - R$5.280	Não
de Ciências Biológicas	Maringá	3 a 5 anos	R$0 - R$1.320	Sim
ncias Humanas, Letras	Maringá	< 1 ano	R$0 - R$1.320	Sim
de Ciências da Saúde	Maringá	3 a 5 anos	R$7.921 ou mais	Sim
Ciências Sociais Aplica	Maringá	3 a 5 anos	R$3.961 - R$5.280	Sim
entro de Tecnologia (CT	Maringá	> 5 anos	R$7.921 ou mais	Sim
entro de Tecnologia (CT	Maringá	3 a 5 anos	R$5.281 - R$6.600	Sim
de Ciências da Saúde	Maringá	> 5 anos	R$1.321 - R$2.640	Não
de Ciências da Saúde	Maringá	1 a 2 anos	R$7.921 ou mais	Não
ncias Humanas, Letras	Maringá	> 5 anos	R$1.321 - R$2.640	Sim
ncias Humanas, Letras	Maringá	3 a 5 anos	R$2.641 - R$3.960	Sim
ro de Ciências Exatas (	Maringá	< 1 ano	R$2.641 - R$3.960	Sim
de Ciências Biológicas	Maringá	3 a 5 anos	R$5.281 - R$6.600	Sim
ncias Humanas, Letras	Maringá	> 5 anos	R$3.961 - R$5.280	Sim
o de Ciências Agrárias (	Maringá	> 5 anos	R$2.641 - R$3.960	Sim
de Ciências Biológicas	Goioerê	< 1 ano	R$5.281 - R$6.600	Sim
de Ciências da Saúde	Maringá	3 a 5 anos	R$7.921 ou mais	Sim
entro de Tecnologia (CT	Maringá	> 5 anos	R$2.641 - R$3.960	Sim
de Ciências Biológicas	Maringá	3 a 5 anos	R$0 - R$1.320	Sim
Ciências Sociais Aplica	Cianorte	> 5 anos	R$3.961 - R$5.280	Não
Ciências Sociais Aplica	Maringá	3 a 5 anos	R$3.961 - R$5.280	Não
ncias Humanas, Letras	Maringá	1 a 2 anos	R$2.641 - R$3.960	Sim
ncias Humanas, Letras	Maringá	> 5 anos	R$7.921 ou mais	Não
entro de Tecnologia (CT	Maringá	> 5 anos	R$7.921 ou mais	Não
Ciências Sociais Aplica	Maringá	1 a 2 anos	R$6.601 - R$7.920	Não
de Ciências da Saúde	Maringá	3 a 5 anos	R$7.921 ou mais	Sim
o de Ciências Agrárias (	Umuarama	< 1 ano	R$3.961 - R$5.280	Não
entro de Tecnologia (CT	Umuarama	3 a 5 anos	R$3.961 - R$5.280	Sim
ncias Humanas, Letras	Maringá	> 5 anos	R$3.961 - R$5.280	Não
ro de Ciências Exatas (	Maringá	3 a 5 anos	R$3.961 - R$5.280	Não
Ciências Sociais Aplica	Maringá	> 5 anos	R$5.281 - R$6.600	Não
Ciências Sociais Aplica	Maringá	< 1 ano	R$7.921 ou mais	Sim

ro de Ciências Exatas ((	Maringá	3 a 5 anos	R$5.281 - R$6.600	Sim
o de Ciências Agrárias (	Maringá	3 a 5 anos	R$2.641 - R$3.960	Sim
entro de Tecnologia (CT	Maringá	3 a 5 anos	R$3.961 - R$5.280	Sim
entro de Tecnologia (CT	Maringá	< 1 ano	R$5.281 - R$6.600	Não
de Ciências da Saúde	Maringá	1 a 2 anos	R$1.321 - R$2.640	Sim
o de Ciências Agrárias (	Umuarama	3 a 5 anos	R$3.961 - R$5.280	Sim
ncias Humanas, Letras	Maringá	< 1 ano	R$3.961 - R$5.280	Não
de Ciências Biológicas	Maringá	1 a 2 anos	R$7.921 ou mais	Não
o de Ciências Agrárias (	Maringá	3 a 5 anos	R$6.601 - R$7.920	Sim
de Ciências da Saúde	Maringá	< 1 ano	R$7.921 ou mais	Sim
ncias Humanas, Letras	Maringá	3 a 5 anos	R$7.921 ou mais	Sim
ncias Humanas, Letras	Ivaiporã	< 1 ano	R$3.961 - R$5.280	Não
entro de Tecnologia (CT	Maringá	3 a 5 anos	R$1.321 - R$2.640	Não
o de Ciências Agrárias (	Maringá	3 a 5 anos	R$2.641 - R$3.960	Sim
de Ciências da Saúde	Maringá	< 1 ano	R$1.321 - R$2.640	Sim
entro de Tecnologia (CT	Maringá	> 5 anos	R$2.641 - R$3.960	Não
o de Ciências Agrárias (	Umuarama	3 a 5 anos	R$0 - R$1.320	Não
Ciências Sociais Aplica	Maringá	1 a 2 anos	R$5.281 - R$6.600	Sim
de Ciências da Saúde	Maringá	3 a 5 anos	R$5.281 - R$6.600	Sim
de Ciências Biológicas	Maringá	1 a 2 anos	R$7.921 ou mais	Sim
entro de Tecnologia (CT	Maringá	3 a 5 anos	R$7.921 ou mais	Sim
ncias Humanas, Letras	Maringá	3 a 5 anos	R$2.641 - R$3.960	Não
ncias Humanas, Letras	Maringá	< 1 ano	R$1.321 - R$2.640	Sim
Ciências Sociais Aplica	Maringá	1 a 2 anos	R$2.641 - R$3.960	Sim
ncias Humanas, Letras	Maringá	3 a 5 anos	R$1.321 - R$2.640	Sim
Ciências Sociais Aplica	Maringá	3 a 5 anos	R$7.921 ou mais	Sim
de Ciências da Saúde	Maringá	3 a 5 anos	R$7.921 ou mais	Sim
ro de Ciências Exatas ((	Maringá	3 a 5 anos	R$2.641 - R$3.960	Não
entro de Tecnologia (CT	Maringá	1 a 2 anos	R$3.961 - R$5.280	Não
Ciências Sociais Aplica	Cianorte	1 a 2 anos	R$2.641 - R$3.960	Não
Ciências Sociais Aplica	Maringá	1 a 2 anos	R$0 - R$1.320	Sim
o de Ciências Agrárias (	Maringá	1 a 2 anos	R$5.281 - R$6.600	Sim
de Ciências da Saúde	Maringá	1 a 2 anos	R$3.961 - R$5.280	Não
entro de Tecnologia (CT	Maringá	3 a 5 anos	R$7.921 ou mais	Sim
Ciências Sociais Aplica	Cianorte	3 a 5 anos	R$5.281 - R$6.600	Sim
Ciências Sociais Aplica	Maringá	1 a 2 anos	R$7.921 ou mais	Não
de Ciências da Saúde	Maringá	1 a 2 anos	R$2.641 - R$3.960	Sim
entro de Tecnologia (CT	Cianorte	3 a 5 anos	R$5.281 - R$6.600	Sim
de Ciências da Saúde	Maringá	1 a 2 anos	R$5.281 - R$6.600	Sim
de Ciências da Saúde	Maringá	1 a 2 anos	R$2.641 - R$3.960	Sim
de Ciências da Saúde	Maringá	1 a 2 anos	R$2.641 - R$3.960	Sim
de Ciências da Saúde	Maringá	> 5 anos	R$7.921 ou mais	Não
de Ciências da Saúde	Ivaiporã	3 a 5 anos	R$2.641 - R$3.960	Sim
de Ciências da Saúde	Maringá	1 a 2 anos	R$5.281 - R$6.600	Sim
de Ciências da Saúde	Maringá	3 a 5 anos	R$0 - R$1.320	Sim
de Ciências da Saúde	Maringá	3 a 5 anos	R$6.601 - R$7.920	Não
de Ciências da Saúde	Maringá	1 a 2 anos	R$3.961 - R$5.280	Sim
ncias Humanas, Letras	Maringá	> 5 anos	R$0 - R$1.320	Não
o de Ciências Agrárias (	Umuarama	3 a 5 anos	R$1.321 - R$2.640	Sim
entro de Tecnologia (CT	Maringá	3 a 5 anos	R$7.921 ou mais	Não
ncias Humanas, Letras	Maringá	1 a 2 anos	R$3.961 - R$5.280	Não
ro de Ciências Exatas ((	Goioerê	< 1 ano	R$7.921 ou mais	Não
de Ciências Biológicas	Maringá	1 a 2 anos	R$2.641 - R$3.960	Não
ncias Humanas, Letras	Maringá	3 a 5 anos	R$5.281 - R$6.600	Sim

de Ciências Biológicas	Maringá	1 a 2 anos	R$3.961 - R$5.280	Sim
ntro de Tecnologia (CT	Maringá	3 a 5 anos	R$2.641 - R$3.960	Sim
ncias Humanas, Letras	Maringá	3 a 5 anos	R$6.601 - R$7.920	Não
ro de Ciências Exatas (	Maringá	< 1 ano	R$1.321 - R$2.640	Sim
de Ciências da Saúde	Maringá	< 1 ano	R$1.321 - R$2.640	Sim
de Ciências da Saúde	Maringá	3 a 5 anos	R$7.921 ou mais	Sim
Ciências Sociais Aplica	Maringá	3 a 5 anos	R$2.641 - R$3.960	Sim
de Ciências da Saúde	Maringá	3 a 5 anos	R$3.961 - R$5.280	Não
Ciências Sociais Aplica	Maringá	3 a 5 anos	R$7.921 ou mais	Não
ntro de Tecnologia (CT	Maringá	1 a 2 anos	R$7.921 ou mais	Sim
Ciências Sociais Aplica	Maringá	> 5 anos	R$3.961 - R$5.280	Sim
ncias Humanas, Letras	Maringá	1 a 2 anos	R$2.641 - R$3.960	Não
ncias Humanas, Letras	Maringá	1 a 2 anos	R$1.321 - R$2.640	Sim
Ciências Sociais Aplica	Maringá	3 a 5 anos	R$5.281 - R$6.600	Não
Ciências Sociais Aplica	Maringá	1 a 2 anos	R$2.641 - R$3.960	Sim
de Ciências da Saúde	Maringá	3 a 5 anos	R$5.281 - R$6.600	Não
ro de Ciências Exatas (	Maringá	1 a 2 anos	R$2.641 - R$3.960	Sim
o de Ciências Agrárias (	Umuarama	3 a 5 anos	R$3.961 - R$5.280	Sim
ncias Humanas, Letras	Maringá	1 a 2 anos	R$6.601 - R$7.920	Sim
ntro de Tecnologia (CT	Goioerê	< 1 ano	R$1.321 - R$2.640	Não
ro de Ciências Exatas (	Maringá	3 a 5 anos	R$7.921 ou mais	Sim
ntro de Tecnologia (CT	Maringá	3 a 5 anos	R$6.601 - R$7.920	Não
Ciências Sociais Aplica	Maringá	< 1 ano	R$5.281 - R$6.600	Não
ntro de Tecnologia (CT	Maringá	> 5 anos	R$6.601 - R$7.920	Não
Ciências Sociais Aplica	Maringá	< 1 ano	R$2.641 - R$3.960	Não
de Ciências da Saúde	Maringá	< 1 ano	R$3.961 - R$5.280	Sim
de Ciências da Saúde	Maringá	3 a 5 anos	R$7.921 ou mais	Sim
de Ciências da Saúde	Maringá	1 a 2 anos	R$3.961 - R$5.280	Não
ncias Humanas, Letras	Maringá	3 a 5 anos	R$1.321 - R$2.640	Sim
o de Ciências Agrárias (	Umuarama	3 a 5 anos	R$1.321 - R$2.640	Sim
ro de Ciências Exatas (	Goioerê	1 a 2 anos	R$7.921 ou mais	Sim
ncias Humanas, Letras	Maringá	1 a 2 anos	R$7.921 ou mais	Sim
ncias Humanas, Letras	Maringá	3 a 5 anos	R$7.921 ou mais	Não
ncias Humanas, Letras	Maringá	1 a 2 anos	R$2.641 - R$3.960	Não
ro de Ciências Exatas (	Diamante do Norte	1 a 2 anos	R$1.321 - R$2.640	Sim
Ciências Sociais Aplica	Maringá	< 1 ano	R$1.321 - R$2.640	Não
ncias Humanas, Letras	Maringá	< 1 ano	R$2.641 - R$3.960	Sim
ncias Humanas, Letras	Maringá	< 1 ano	R$1.321 - R$2.640	Não
ro de Ciências Exatas (	Maringá	3 a 5 anos	R$5.281 - R$6.600	Não
de Ciências da Saúde	Maringá	3 a 5 anos	R$3.961 - R$5.280	Sim
de Ciências da Saúde	Maringá	3 a 5 anos	R$7.921 ou mais	Sim
ncias Humanas, Letras	Maringá	3 a 5 anos	R$1.321 - R$2.640	Sim
de Ciências Biológicas	Maringá	3 a 5 anos	R$1.321 - R$2.640	Sim
ncias Humanas, Letras	Maringá	1 a 2 anos	R$1.321 - R$2.640	Sim
ro de Ciências Exatas (	Goioerê	< 1 ano	R$0 - R$1.320	Sim
ncias Humanas, Letras	Maringá	< 1 ano	R$1.321 - R$2.640	Não
de Ciências da Saúde	Maringá	3 a 5 anos	R$7.921 ou mais	Não
ncias Humanas, Letras	Maringá	< 1 ano	R$0 - R$1.320	Não
ntro de Tecnologia (CT	Maringá	< 1 ano	R$3.961 - R$5.280	Sim
de Ciências Biológicas	Maringá	< 1 ano	R$1.321 - R$2.640	Sim
de Ciências Biológicas	Diamante do Norte	1 a 2 anos	R$2.641 - R$3.960	Sim
de Ciências da Saúde	Maringá	> 5 anos	R$3.961 - R$5.280	Sim
ncias Humanas, Letras	Maringá	1 a 2 anos	R$5.281 - R$6.600	Sim
ro de Ciências Exatas (	Maringá	3 a 5 anos	R$1.321 - R$2.640	Sim

de Ciências da Saúde	Maringá	3 a 5 anos	R$1.321 - R$2.640	Não
de Ciências da Saúde	Maringá	1 a 2 anos	R$3.961 - R$5.280	Não
de Ciências Biológicas	Maringá	< 1 ano	R$2.641 - R$3.960	Sim
Ciências Sociais Aplica	Maringá	3 a 5 anos	R$1.321 - R$2.640	Não
Ciências Sociais Aplica	Maringá	1 a 2 anos	R$6.601 - R$7.920	Sim
de Ciências Biológicas	Maringá	< 1 ano	R$1.321 - R$2.640	Sim
de Ciências Biológicas	Maringá	3 a 5 anos	R$3.961 - R$5.280	Sim
Ciências Sociais Aplica	Maringá	3 a 5 anos	R$2.641 - R$3.960	Não
ncias Humanas, Letras	Maringá	3 a 5 anos	R$7.921 ou mais	Não
Ciências Sociais Aplica	Maringá	1 a 2 anos	R$1.321 - R$2.640	Sim
Ciências Sociais Aplica	Maringá	1 a 2 anos	R$1.321 - R$2.640	Sim
ncias Humanas, Letras	Umuarama	< 1 ano	R$5.281 - R$6.600	Sim
ncias Humanas, Letras	Maringá	3 a 5 anos	R$6.601 - R$7.920	Sim
ncias Humanas, Letras	Maringá	1 a 2 anos	R$1.321 - R$2.640	Sim
ro de Ciências Exatas (	Maringá	< 1 ano	R$5.281 - R$6.600	Sim
de Ciências da Saúde	Maringá	1 a 2 anos	R$7.921 ou mais	Sim
de Ciências da Saúde	Maringá	3 a 5 anos	R$3.961 - R$5.280	Sim
ro de Ciências Exatas (	Maringá	3 a 5 anos	R$2.641 - R$3.960	Sim
ncias Humanas, Letras	Maringá	1 a 2 anos	R$0 - R$1.320	Sim
ncias Humanas, Letras	Maringá	< 1 ano	R$3.961 - R$5.280	Sim
entro de Tecnologia (CT	Maringá	3 a 5 anos	R$0 - R$1.320	Não
entro de Tecnologia (CT	Umuarama	1 a 2 anos	R$7.921 ou mais	Sim
ncias Humanas, Letras	Maringá	< 1 ano	R$2.641 - R$3.960	Sim
ncias Humanas, Letras	Maringá	> 5 anos	R$5.281 - R$6.600	Sim
ro de Ciências Exatas (	Maringá	< 1 ano	R$3.961 - R$5.280	Sim
ncias Humanas, Letras	Maringá	< 1 ano	R$7.921 ou mais	Sim
Ciências Sociais Aplica	Maringá	3 a 5 anos	R$2.641 - R$3.960	Sim
ro de Ciências Exatas (	Maringá	3 a 5 anos	R$6.601 - R$7.920	Sim
Ciências Sociais Aplica	Maringá	1 a 2 anos	R$6.601 - R$7.920	Sim
ro de Ciências Exatas (	Maringá	3 a 5 anos	R$3.961 - R$5.280	Sim
ncias Humanas, Letras	Maringá	> 5 anos	R$1.321 - R$2.640	Sim
ncias Humanas, Letras	Cianorte	3 a 5 anos	R$3.961 - R$5.280	Sim
Ciências Sociais Aplica	Maringá	1 a 2 anos	R$3.961 - R$5.280	Sim
Ciências Sociais Aplica	Maringá	1 a 2 anos	R$2.641 - R$3.960	Não
ncias Humanas, Letras	Maringá	< 1 ano	R$1.321 - R$2.640	Sim
de Ciências da Saúde	Maringá	< 1 ano	R$3.961 - R$5.280	Sim
Ciências Sociais Aplica	Maringá	> 5 anos	R$1.321 - R$2.640	Sim
Ciências Sociais Aplica	Maringá	1 a 2 anos	R$1.321 - R$2.640	Não
entro de Tecnologia (CT	Umuarama	1 a 2 anos	R$3.961 - R$5.280	Sim
ncias Humanas, Letras	Umuarama	< 1 ano	R$1.321 - R$2.640	Não
ro de Ciências Exatas (	Maringá	> 5 anos	R$5.281 - R$6.600	Sim
ncias Humanas, Letras	Maringá	< 1 ano	R$3.961 - R$5.280	Sim
ncias Humanas, Letras	Maringá	> 5 anos	R$0 - R$1.320	Sim
ncias Humanas, Letras	Maringá	> 5 anos	R$2.641 - R$3.960	Sim
ncias Humanas, Letras	Maringá	3 a 5 anos	R$2.641 - R$3.960	Sim
ro de Ciências Exatas (	Maringá	< 1 ano	R$1.321 - R$2.640	Sim
Ciências Sociais Aplica	Maringá	1 a 2 anos	R$7.921 ou mais	Sim
ncias Humanas, Letras	Maringá	1 a 2 anos	R$3.961 - R$5.280	Sim
ro de Ciências Exatas (	Maringá	3 a 5 anos	R$1.321 - R$2.640	Não
ncias Humanas, Letras	Maringá	< 1 ano	R$1.321 - R$2.640	Sim
entro de Tecnologia (CT	Maringá	3 a 5 anos	R$1.321 - R$2.640	Sim
Ciências Sociais Aplica	Maringá	3 a 5 anos	R$7.921 ou mais	Sim
ncias Humanas, Letras	Maringá	1 a 2 anos	R$1.321 - R$2.640	Sim
ncias Humanas, Letras	Maringá	1 a 2 anos	R$3.961 - R$5.280	Não

o de Ciências Agrárias (	Maringá	< 1 ano	R$3.961 - R$5.280	Sim
entro de Tecnologia (CT	Umuarama	< 1 ano	R$2.641 - R$3.960	Sim
o de Ciências Agrárias (	Umuarama	< 1 ano	R$1.321 - R$2.640	Sim
ncias Humanas, Letras	Maringá	< 1 ano	R$3.961 - R$5.280	Sim
ro de Ciências Exatas ((	Maringá	3 a 5 anos	R$2.641 - R$3.960	Sim
ncias Humanas, Letras	Maringá	3 a 5 anos	R$1.321 - R$2.640	Sim
ncias Humanas, Letras	Maringá	1 a 2 anos	R$1.321 - R$2.640	Sim
entro de Tecnologia (CT	Cianorte	< 1 ano	R$3.961 - R$5.280	Sim
ncias Humanas, Letras	Maringá	< 1 ano	R$0 - R$1.320	Sim
Ciências Sociais Aplica	Ivaiporã	3 a 5 anos	R$0 - R$1.320	Sim
o de Ciências da Saúde	Maringá	3 a 5 anos	R$7.921 ou mais	Não
Ciências Sociais Aplica	Maringá	3 a 5 anos	R$7.921 ou mais	Não
ncias Humanas, Letras	Maringá	1 a 2 anos	R$2.641 - R$3.960	Sim
Ciências Sociais Aplica	Maringá	< 1 ano	R$1.321 - R$2.640	Sim
ncias Humanas, Letras	Maringá	1 a 2 anos	R$7.921 ou mais	Sim
ro de Ciências Exatas ((	Umuarama	1 a 2 anos	R$7.921 ou mais	Sim
ncias Humanas, Letras	Maringá	< 1 ano	R$3.961 - R$5.280	Sim
Ciências Sociais Aplica	Maringá	1 a 2 anos	R$2.641 - R$3.960	Não

ParceriaFixa3meses	TesteRápido	OuviuPrEP	OuviuPEP	UsouPrEP
Sim	Não	Não	Não	Não
Não	Não	Não	Não	Não
Sim	Não	Não	Não	Não
Não tenho relações	Não	Não	Não	Não
Sim	Não	Não	Sim	Não
Sim	Não	Não	Não	Não
Não tenho relações	Não	Não	Não	Não
Sim	Sim	Sim	Sim	Não
Sim	Sim	Não	Não	Não
Sim	Sim	Sim	Sim	Não
Sim	Não	Sim	Sim	Não
Não	Não	Não	Não	Não
Não	Não	Sim	Sim	Não
Sim	Sim	Sim	Sim	Não
Sim	Sim	Não	Não	Não
Sim	Sim	Não	Não	Não
Não	Sim	Sim	Sim	Não
Não tenho relações	Não	Não	Não	Não
Sim	Não	Não	Não	Não
Sim	Sim	Sim	Sim	Não
Sim	Não	Não	Não	Não
Sim	Sim	Sim	Não	Não
Sim	Sim	Não	Sim	Não
Sim	Não	Não	Sim	Não
Sim	Não	Sim	Sim	Não
Sim	Sim	Não	Não	Não
Sim	Não	Sim	Sim	Não
Sim	Não	Não	Não	Não
Sim	Sim	Sim	Sim	Sim
Não	Sim	Sim	Sim	Não
Não	Não	Sim	Não	Não
Não tenho relações	Não	Sim	Sim	Não
Sim	Não	Sim	Sim	Não
Não tenho relações	Não	Sim	Sim	Não
Sim	Sim	Sim	Sim	Não
Sim	Não	Sim	Não	Não
Não tenho relações	Não	Sim	Sim	Não
Sim	Sim	Sim	Sim	Não
Sim	Sim	Sim	Sim	Não
Sim	Não	Sim	Sim	Não
Não	Sim	Sim	Sim	Não
Não	Não	Sim	Sim	Não
Não tenho relações	Não	Não	Não	Não
Sim	Não	Não	Não	Não
Não	Não	Sim	Não	Não
Sim	Não	Não	Sim	Não
Não tenho relações	Não	Sim	Sim	Não
Não tenho relações	Não	Sim	Sim	Não
Sim	Sim	Sim	Sim	Não
Não tenho relações	Não	Não	Não	Não
Não	Não	Não	Não	Não
Não tenho relações	Não	Não	Sim	Não
Sim	Não	Não	Não	Não

Sim	Não	Sim	Sim	Não
Não tenho relações	Não	Não	Não	Não
Sim	Não	Não	Não	Não
Não tenho relações	Não	Não	Não	Não
Sim	Não	Sim	Sim	Não
Sim	Não	Sim	Sim	Não
Não tenho relações	Não	Não	Não	Não
Não	Sim	Não	Não	Não
Não	Sim	Sim	Sim	Não
Sim	Sim	Sim	Sim	Não
Sim	Não	Não	Sim	Não
Sim	Não	Sim	Sim	Não
Sim	Não	Não	Não	Não
Sim	Não	Não	Sim	Não
Não	Não	Sim	Sim	Não
Sim	Não	Não	Não	Não
Não tenho relações	Não	Não	Sim	Não
Não tenho relações	Não	Não	Não	Não
Não	Não	Não	Não	Não
Sim	Não	Não	Não	Não
Sim	Não	Sim	Sim	Não
Sim	Não	Sim	Não	Não
Não tenho relações	Não	Não	Não	Não
Sim	Não	Sim	Sim	Não
Sim	Não	Não	Sim	Não
Não tenho relações	Não	Não	Sim	Não
Sim	Não	Sim	Sim	Não
Sim	Não	Não	Não	Não
Sim	Não	Não	Não	Não
Não tenho relações	Não	Sim	Sim	Não
Sim	Não	Sim	Sim	Não
Não tenho relações	Não	Não	Sim	Não
Não	Sim	Não	Não	Não
Sim	Não	Não	Não	Não
Não tenho relações	Não	Não	Não	Não
Sim	Sim	Sim	Sim	Não
Não tenho relações	Não	Sim	Sim	Não
Não	Não	Sim	Sim	Não
Sim	Sim	Sim	Sim	Não
Sim	Não	Não	Não	Não
Sim	Não	Não	Não	Não
Sim	Não	Não	Sim	Não
Sim	Não	Não	Não	Não
Não tenho relações	Não	Não	Não	Não
Não tenho relações	Sim	Sim	Sim	Não
Sim	Não	Não	Não	Não
Não	Sim	Sim	Não	Sim
Não	Não	Não	Não	Não
Sim	Não	Sim	Sim	Não
Sim	Não	Não	Não	Não
Não tenho relações	Não	Sim	Sim	Não
Não tenho relações	Não	Sim	Sim	Não
Sim	Não	Não	Não	Não
Não	Não	Não	Não	Não

Sim	Não	Sim	Sim	Não
Sim	Não	Não	Não	Não
Não	Não	Não	Não	Não
Não tenho relações	Não	Não	Não	Não
Sim	Não	Não	Não	Não
Não tenho relações	Não	Sim	Sim	Não
Não	Não	Sim	Sim	Não
Sim	Não	Não	Não	Não
Não tenho relações	Não	Não	Não	Não
Sim	Sim	Não	Não	Não
Sim	Não	Não	Não	Não
Não	Sim	Sim	Sim	Sim
Sim	Não	Não	Não	Não
Sim	Não	Sim	Sim	Não
Não	Não	Sim	Não	Não
Não tenho relações	Não	Não	Não	Não
Sim	Não	Sim	Sim	Não
Não tenho relações	Não	Não	Não	Não
Não tenho relações	Não	Não	Não	Não
Não	Sim	Sim	Sim	Não
Não tenho relações	Não	Sim	Não	Não
Sim	Não	Não	Não	Não
Sim	Não	Sim	Sim	Não
Sim	Não	Não	Sim	Não
Sim	Não	Não	Não	Não
Não tenho relações	Não	Não	Não	Não
Sim	Não	Não	Não	Não
Não tenho relações	Não	Não	Não	Não
Sim	Sim	Sim	Sim	Não
Sim	Não	Sim	Sim	Não
Não	Sim	Sim	Sim	Não
Sim	Sim	Não	Não	Não
Não	Sim	Não	Não	Não
Não	Não	Sim	Sim	Não
Não	Não	Não	Sim	Não
Sim	Não	Não	Não	Não
Não	Não	Não	Não	Não
Não tenho relações	Não	Não	Não	Não
Sim	Sim	Não	Sim	Não
Não tenho relações	Não	Não	Sim	Não
Sim	Não	Não	Não	Não
Sim	Não	Não	Sim	Não
Não	Não	Sim	Sim	Não
Sim	Não	Não	Não	Não
Não	Sim	Sim	Sim	Não
Não tenho relações	Sim	Sim	Sim	Não
Sim	Não	Não	Sim	Não
Sim	Não	Sim	Sim	Não
Não	Sim	Não	Não	Não
Sim	Não	Não	Não	Não
Não tenho relações	Não	Sim	Sim	Não
Sim	Não	Sim	Sim	Não
Não tenho relações	Não	Não	Não	Não
Não	Sim	Sim	Sim	Não

Sim	Não	Não	Não	Não
Sim	Não	Não	Não	Não
Sim	Não	Sim	Sim	Não
Não tenho relações	Não	Sim	Sim	Não
Sim	Não	Não	Não	Não
Sim	Não	Não	Sim	Não
Não tenho relações	Não	Não	Não	Não
Sim	Não	Não	Não	Não
Sim	Sim	Sim	Sim	Não
Não	Sim	Não	Não	Não
Não	Não	Sim	Não	Não
Sim	Não	Não	Não	Não
Não tenho relações	Não	Não	Não	Não
Não	Não	Sim	Sim	Não
Sim	Não	Sim	Sim	Não
Não	Não	Não	Não	Não
Sim	Não	Não	Não	Não
Não	Não	Não	Não	Não
Sim	Não	Não	Não	Não
Sim	Não	Sim	Não	Não
Sim	Sim	Não	Não	Não
Sim	Não	Sim	Sim	Sim
Sim	Não	Não	Não	Não
Não	Não	Não	Não	Não
Sim	Não	Sim	Sim	Não
Sim	Não	Não	Sim	Não
Não	Não	Não	Não	Não
Não tenho relações	Não	Sim	Sim	Não
Não tenho relações	Sim	Sim	Sim	Não
Não tenho relações	Não	Não	Não	Não
Não tenho relações	Não	Não	Não	Não
Não	Sim	Sim	Sim	Não
Não tenho relações	Sim	Sim	Sim	Não
Sim	Não	Sim	Sim	Não
Sim	Não	Não	Não	Não
Não	Não	Sim	Sim	Não
Não	Não	Não	Não	Não
Sim	Não	Sim	Sim	Não
Não tenho relações	Não	Não	Não	Não
Sim	Não	Sim	Sim	Não
Não tenho relações	Não	Sim	Sim	Não
Não	Não	Sim	Sim	Não
Sim	Não	Não	Não	Não
Não tenho relações	Não	Sim	Sim	Não
Sim	Não	Sim	Sim	Não
Não	Sim	Sim	Sim	Não
Não	Não	Sim	Sim	Não
Sim	Não	Não	Não	Não
Não tenho relações	Não	Não	Não	Não
Não tenho relações	Não	Sim	Sim	Não
Não	Não	Não	Não	Não
Sim	Não	Não	Não	Não
Não tenho relações	Não	Não	Não	Não
Não	Não	Não	Não	Não

Sim	Não	Não	Não	Não
Sim	Não	Não	Não	Não
Sim	Não	Não	Não	Não
Não tenho relações	Não	Não	Não	Não
Sim	Não	Não	Não	Não
Não tenho relações	Não	Sim	Sim	Não
Sim	Não	Sim	Não	Não
Não tenho relações	Não	Não	Não	Não
Não	Sim	Sim	Sim	Não
Sim	Sim	Sim	Sim	Sim
Não tenho relações	Não	Não	Sim	Não
Sim	Não	Não	Sim	Não
Não tenho relações	Sim	Não	Não	Não
Sim	Não	Não	Não	Não
Não tenho relações	Não	Não	Sim	Não
Sim	Não	Não	Não	Não
Sim	Não	Sim	Sim	Não
Não	Sim	Sim	Sim	Não
Sim	Sim	Sim	Sim	Sim
Sim	Não	Não	Não	Não
Não tenho relações	Não	Sim	Sim	Não
Não	Não	Não	Não	Não
Não	Sim	Sim	Sim	Não
Sim	Não	Sim	Sim	Não
Sim	Não	Não	Não	Não
Não	Sim	Sim	Sim	Não
Não tenho relações	Não	Não	Não	Não
Sim	Não	Não	Não	Não
Sim	Não	Sim	Sim	Não
Não	Não	Sim	Sim	Não
Sim	Sim	Sim	Sim	Não
Não	Sim	Não	Não	Não
Não tenho relações	Sim	Não	Não	Não
Sim	Não	Não	Sim	Não
Não	Sim	Sim	Sim	Não
Sim	Sim	Sim	Sim	Sim
Sim	Sim	Sim	Sim	Não
Sim	Não	Não	Não	Não
Não	Não	Sim	Sim	Não
Não tenho relações	Não	Não	Sim	Não
Sim	Sim	Não	Sim	Não
Sim	Sim	Sim	Sim	Não
Sim	Sim	Não	Não	Não
Sim	Não	Não	Não	Não
Sim	Sim	Não	Não	Não
Sim	Sim	Sim	Sim	Não
Não tenho relações	Não	Não	Não	Não
Não	Não	Não	Não	Não
Não tenho relações	Não	Sim	Sim	Não
Não	Sim	Sim	Sim	Não
Não tenho relações	Não	Sim	Sim	Não
Não tenho relações	Não	Não	Não	Não
Sim	Não	Sim	Sim	Não
Sim	Não	Sim	Sim	Não

Não	Sim	Não	Não	Não
Sim	Sim	Sim	Sim	Sim
Não	Não	Sim	Sim	Não
Sim	Sim	Não	Não	Não
Sim	Sim	Não	Não	Não
Sim	Não	Não	Sim	Não
Sim	Sim	Sim	Sim	Não
Sim	Sim	Sim	Sim	Não
Sim	Sim	Sim	Sim	Não
Não	Não	Não	Sim	Não
Não tenho relações	Não	Sim	Sim	Sim
Sim	Sim	Sim	Sim	Sim
Sim	Não	Sim	Sim	Não
Sim	Sim	Sim	Sim	Não
Não	Sim	Sim	Sim	Não
Não	Não	Sim	Sim	Não
Não	Não	Não	Não	Não
Não tenho relações	Não	Sim	Sim	Não
Não	Não	Não	Sim	Não
Não	Não	Sim	Não	Não
Sim	Não	Não	Não	Não
Não	Sim	Sim	Sim	Sim
Não	Sim	Sim	Sim	Não
Não	Sim	Sim	Sim	Não
Não	Sim	Não	Sim	Não
Sim	Não	Sim	Sim	Não
Sim	Não	Não	Não	Não
Sim	Não	Não	Não	Não
Sim	Não	Sim	Não	Sim
Não tenho relações	Sim	Sim	Sim	Sim
Não tenho relações	Não	Sim	Sim	Não
Não	Não	Sim	Sim	Sim
Sim	Sim	Sim	Sim	Não
Não	Sim	Sim	Sim	Não
Sim	Sim	Não	Não	Não
Sim	Sim	Sim	Sim	Não
Sim	Não	Não	Não	Não
Sim	Não	Não	Não	Não
Sim	Não	Sim	Sim	Não
Não	Sim	Sim	Sim	Não
Sim	Não	Não	Sim	Não
Não	Sim	Sim	Sim	Não
Não tenho relações	Não	Não	Sim	Não
Sim	Não	Sim	Não	Não
Não tenho relações	Não	Sim	Sim	Não
Não tenho relações	Não	Não	Não	Não
Não	Não	Sim	Sim	Não
Não	Não	Sim	Sim	Não
Não tenho relações	Não	Não	Não	Não
Sim	Não	Não	Não	Não
Não	Não	Não	Não	Não
Não tenho relações	Sim	Sim	Sim	Não
Não tenho relações	Não	Não	Não	Não
Sim	Não	Não	Sim	Não

Sim	Não	Não	Não	Não
Sim	Sim	Não	Não	Não
Sim	Sim	Sim	Sim	Não
Não tenho relações	Não	Sim	Não	Não
Sim	Não	Sim	Sim	Não
Sim	Não	Não	Não	Não
Não	Não	Não	Não	Não
Não	Não	Não	Não	Não
Sim	Não	Não	Não	Não
Sim	Não	Sim	Sim	Não
Sim	Sim	Sim	Sim	Não
Não tenho relações	Não	Não	Não	Não
Não	Não	Não	Não	Não
Sim	Não	Não	Não	Não
Sim	Não	Não	Não	Não
Não tenho relações	Não	Sim	Não	Não
Não tenho relações	Não	Não	Não	Não
Sim	Não	Não	Sim	Não
Sim	Não	Sim	Sim	Não
Não tenho relações	Sim	Não	Não	Não
Não	Não	Não	Não	Não
Não tenho relações	Não	Não	Não	Não
Sim	Sim	Sim	Sim	Não
Sim	Sim	Não	Não	Não
Sim	Não	Não	Não	Não
Sim	Sim	Não	Não	Não
Sim	Sim	Não	Não	Não
Não tenho relações	Não	Sim	Sim	Não
Não tenho relações	Não	Não	Não	Não
Não tenho relações	Não	Sim	Sim	Não
Sim	Sim	Sim	Sim	Sim
Não	Não	Não	Não	Não
Não tenho relações	Não	Sim	Sim	Não
Sim	Não	Sim	Sim	Não
Sim	Sim	Sim	Sim	Não
Não tenho relações	Não	Não	Não	Não
Sim	Não	Sim	Sim	Não
Sim	Não	Não	Não	Não
Sim	Sim	Não	Não	Não
Sim	Sim	Sim	Sim	Não
Não	Sim	Sim	Sim	Não
Não tenho relações	Não	Sim	Sim	Não
Sim	Não	Não	Não	Não
Não	Não	Sim	Sim	Não
Não	Sim	Sim	Sim	Não
Não tenho relações	Sim	Sim	Sim	Não
Não	Não	Sim	Sim	Não
Não	Sim	Sim	Sim	Não
Sim	Não	Não	Não	Não
Não tenho relações	Não	Não	Não	Não
Não tenho relações	Não	Sim	Sim	Não
Não	Não	Não	Não	Não
Não tenho relações	Não	Sim	Sim	Sim
Sim	Não	Não	Não	Não

Sim	Não	Sim	Sim	Não
Sim	Não	Não	Não	Não
Não tenho relações	Sim	Não	Não	Não
Não	Sim	Sim	Sim	Não
Sim	Não	Não	Não	Não
Sim	Sim	Sim	Sim	Não
Sim	Não	Não	Não	Não
Não tenho relações	Não	Sim	Sim	Não
Não tenho relações	Não	Não	Não	Não
Sim	Sim	Não	Não	Não
Não	Não	Sim	Sim	Não
Não	Sim	Sim	Sim	Não
Sim	Não	Sim	Sim	Não
Não tenho relações	Não	Não	Não	Não
Não	Não	Sim	Sim	Não
Não tenho relações	Não	Sim	Sim	Não
Sim	Não	Sim	Sim	Não
Não	Não	Não	Não	Não
Sim	Não	Sim	Sim	Não
Não tenho relações	Não	Não	Não	Não
Sim	Não	Não	Não	Não
Não	Sim	Sim	Sim	Não
Não tenho relações	Não	Não	Não	Não
Não tenho relações	Não	Não	Sim	Não
Sim	Não	Sim	Não	Não
Sim	Não	Não	Não	Não
Não	Não	Não	Sim	Não
Não tenho relações	Não	Sim	Sim	Não
Sim	Sim	Sim	Sim	Não
Sim	Não	Não	Não	Não
Sim	Sim	Não	Não	Não
Sim	Sim	Não	Não	Não
Não tenho relações	Não	Não	Não	Não
Não tenho relações	Não	Não	Não	Não
Sim	Sim	Não	Não	Não
Não tenho relações	Não	Sim	Sim	Não
Sim	Sim	Não	Não	Não
Não	Não	Não	Não	Não
Não tenho relações	Não	Não	Não	Não
Sim	Não	Sim	Sim	Não
Não	Sim	Sim	Sim	Não
Sim	Não	Não	Não	Não
Sim	Não	Não	Não	Não
Sim	Não	Não	Não	Não
Sim	Não	Sim	Sim	Não
Não tenho relações	Não	Sim	Sim	Não
Não tenho relações	Sim	Sim	Sim	Não
Não tenho relações	Não	Não	Não	Não
Sim	Não	Não	Sim	Não
Não	Não	Não	Não	Não
Sim	Sim	Não	Não	Não
Sim	Sim	Sim	Sim	Não
Sim	Não	Não	Não	Não
Sim	Não	Não	Não	Não

Não tenho relações	Não	Sim	Sim	Não
Não tenho relações	Não	Sim	Sim	Não
Sim	Sim	Sim	Sim	Não
Não tenho relações	Não	Não	Não	Não
Sim	Não	Sim	Sim	Não
Sim	Sim	Sim	Sim	Não
Não	Não	Sim	Sim	Não
Não tenho relações	Não	Sim	Sim	Não
Não	Sim	Não	Sim	Não
Não	Sim	Não	Sim	Não
Sim	Sim	Sim	Sim	Sim
Não	Não	Não	Não	Não
Sim	Não	Sim	Sim	Não
Não	Sim	Sim	Sim	Não
Não	Não	Sim	Sim	Não
Não	Sim	Sim	Sim	Não
Não	Sim	Sim	Sim	Não
Não	Não	Não	Não	Não
Não	Sim	Não	Não	Não
Não	Não	Sim	Sim	Não
Não	Sim	Sim	Sim	Não
Sim	Sim	Não	Não	Não
Não	Sim	Sim	Sim	Não
Sim	Sim	Não	Não	Não
Não	Sim	Não	Não	Não
Sim	Sim	Sim	Sim	Não
Não	Sim	Sim	Sim	Não
Sim	Não	Não	Não	Não
Sim	Não	Não	Não	Não
Não	Sim	Sim	Sim	Não
Sim	Sim	Sim	Sim	Não
Sim	Sim	Não	Não	Não
Sim	Sim	Não	Não	Não
Não tenho relações	Não	Sim	Não	Não
Sim	Sim	Sim	Sim	Sim
Sim	Sim	Sim	Sim	Não
Sim	Sim	Não	Não	Não
Não tenho relações	Não	Não	Não	Não
Sim	Não	Sim	Sim	Não
Não tenho relações	Sim	Sim	Sim	Não
Sim	Não	Sim	Sim	Não
Sim	Sim	Sim	Sim	Sim
Sim	Sim	Não	Não	Não
Não	Sim	Não	Não	Não
Sim	Não	Não	Não	Não
Sim	Não	Não	Não	Não
Não	Não	Não	Sim	Não
Sim	Não	Não	Não	Não
Não	Não	Não	Não	Não
Sim	Sim	Sim	Sim	Não
Sim	Não	Não	Não	Não
Sim	Não	Não	Não	Não
Sim	Não	Não	Não	Não
Não tenho relações	Não	Não	Não	Não

Sim	Sim	Não	Não	Não
Sim	Não	Não	Não	Não
Sim	Não	Não	Não	Não
Sim	Não	Sim	Não	Não
Sim	Sim	Sim	Sim	Não
Sim	Sim	Não	Não	Não
Sim	Sim	Não	Não	Não
Sim	Sim	Não	Não	Não
Sim	Sim	Sim	Sim	Não
Sim	Não	Não	Não	Não
Não	Sim	Sim	Sim	Não
Não tenho relações	Não	Não	Não	Não
Sim	Sim	Não	Não	Não
Sim	Não	Não	Não	Não
Sim	Sim	Sim	Sim	Sim
Sim	Sim	Não	Não	Não
Não	Sim	Sim	Sim	Não
Não	Não	Não	Não	Não

UsouPEP	abe o que é a infecção	Q1	Q2	Q3
Não	Sim	Errado	Errado	Correto
Não	Sim	Correto	Correto	Correto
Não	Sim	Correto	Correto	Correto
Não	Sim	Correto	Correto	Correto
Não	Sim	Correto	Errado	Correto
Não	Sim	Correto	Não sei dizer	Correto
Não	Sim	Não sei dizer	Correto	Não sei dizer
Não	Sim	Correto	Correto	Correto
Não	Sim	Correto	Correto	Correto
Não	Sim	Correto	Correto	Correto
Não	Sim	Correto	Errado	Correto
Não	Sim	Correto	Errado	Correto
Não	Sim	Correto	Correto	Correto
Não	Sim	Correto	Correto	Correto
Não	Sim	Correto	Correto	Correto
Não	Sim	Correto	Errado	Correto
Não	Sim	Correto	Correto	Correto
Não	Sim	Correto	Correto	Correto
Não	Sim	Correto	Correto	Correto
Não	Sim	Correto	Correto	Correto
Não	Sim	Correto	Correto	Correto
Não	Sim	Correto	Correto	Correto
Não	Sim	Correto	Correto	Correto
Não	Sim	Correto	Não sei dizer	Correto
Não	Sim	Correto	Correto	Correto
Não	Sim	Correto	Correto	Correto
Não	Sim	Correto	Correto	Correto
Não	Sim	Correto	Errado	Correto
Não	Sim	Correto	Correto	Correto
Não	Sim	Correto	Correto	Correto
Não	Sim	Correto	Não sei dizer	Correto
Não	Sim	Errado	Errado	Correto
Não	Sim	Correto	Correto	Correto
Não	Sim	Correto	Correto	Correto
Não	Sim	Correto	Errado	Correto
Não	Sim	Correto	Correto	Correto
Não	Sim	Correto	Correto	Correto
Não	Sim	Correto	Correto	Correto
Não	Sim	Correto	Correto	Correto
Não	Sim	Correto	Correto	Correto
Não	Sim	Correto	Correto	Correto
Não	Sim	Errado	Errado	Correto
Não	Sim	Correto	Correto	Correto
Não	Sim	Correto	Correto	Correto
Não	Sim	Correto	Correto	Correto
Não	Sim	Correto	Correto	Correto
Não	Sim	Correto	Correto	Correto
Não	Sim	Correto	Correto	Correto
Não	Sim	Errado	Correto	Correto
Não	Sim	Correto	Correto	Correto
Não	Sim	Correto	Correto	Correto
Não	Sim	Correto	Correto	Correto
Não	Sim	Correto	Correto	Correto

Não	Sim	Correto	Correto	Correto
Não	Sim	Não sei dizer	Não sei dizer	Correto
Não	Sim	Correto	Não sei dizer	Correto
Não	Sim	Correto	Correto	Correto
Não	Sim	Correto	Correto	Correto
Não	Sim	Correto	Correto	Correto
Não	Sim	Correto	Errado	Correto
Não	Sim	Correto	Correto	Correto
Não	Sim	Correto	Correto	Correto
Não	Sim	Correto	Correto	Correto
Não	Sim	Correto	Correto	Correto
Não	Sim	Correto	Correto	Correto
Não	Sim	Correto	Não sei dizer	Correto
Não	Sim	Correto	Correto	Correto
Não	Sim	Errado	Não sei dizer	Correto
Não	Sim	Correto	Errado	Correto
Não	Sim	Correto	Errado	Correto
Não	Sim	Correto	Correto	Correto
Não	Sim	Correto	Correto	Correto
Não	Sim	Correto	Errado	Correto
Não	Sim	Correto	Não sei dizer	Correto
Não	Sim	Correto	Correto	Correto
Não	Sim	Correto	Correto	Correto
Não	Sim	Correto	Correto	Correto
Não	Sim	Correto	Correto	Correto
Não	Sim	Correto	Correto	Correto
Não	Sim	Correto	Correto	Correto
Não	Sim	Correto	Errado	Correto
Não	Sim	Correto	Errado	Correto
Não	Sim	Correto	Correto	Correto
Não	Sim	Correto	Correto	Correto
Não	Sim	Correto	Não sei dizer	Correto
Não	Sim	Correto	Errado	Correto
Não	Sim	Correto	Correto	Correto
Não	Sim	Correto	Correto	Correto
Não	Sim	Correto	Errado	Correto
Não	Sim	Correto	Correto	Correto
Não	Sim	Correto	Correto	Correto
Não	Sim	Correto	Correto	Correto
Não	Sim	Correto	Correto	Correto
Não	Sim	Correto	Errado	Correto
Não	Sim	Correto	Errado	Correto
Não	Sim	Correto	Correto	Errado
Não	Sim	Correto	Errado	Correto
Não	Sim	Correto	Correto	Correto
Não	Sim	Correto	Errado	Correto
Não	Sim	Correto	Errado	Correto
Não	Sim	Não sei dizer	Não sei dizer	Correto
Não	Sim	Correto	Não sei dizer	Correto
Não	Sim	Correto	Correto	Correto
Não	Sim	Correto	Errado	Correto
Não	Sim	Errado	Correto	Correto
Não	Sim	Correto	Correto	Correto
Não	Sim	Não sei dizer	Correto	Correto

Não	Sim	Correto	Errado	Correto
Não	Sim	Correto	Não sei dizer	Correto
Não	Sim	Correto	Correto	Correto
Não	Não	Não sei dizer	Não sei dizer	Correto
Não	Sim	Correto	Correto	Correto
Não	Sim	Correto	Correto	Correto
Não	Sim	Errado	Correto	Correto
Não	Sim	Correto	Errado	Correto
Não	Sim	Correto	Correto	Correto
Não	Sim	Correto	Correto	Correto
Não	Sim	Correto	Errado	Correto
Não	Sim	Correto	Correto	Correto
Não	Sim	Correto	Correto	Correto
Não	Sim	Correto	Errado	Correto
Não	Sim	Correto	Correto	Correto
Não	Sim	Correto	Correto	Correto
Não	Sim	Correto	Correto	Correto
Não	Sim	Correto	Errado	Correto
Não	Sim	Correto	Correto	Correto
Não	Sim	Correto	Correto	Correto
Não	Sim	Correto	Correto	Correto
Não	Sim	Correto	Não sei dizer	Correto
Não	Sim	Correto	Correto	Correto
Não	Sim	Correto	Errado	Correto
Não	Sim	Correto	Não sei dizer	Não sei dizer
Não	Sim	Correto	Correto	Correto
Não	Sim	Não sei dizer	Correto	Correto
Não	Sim	Correto	Correto	Correto
Sim	Sim	Correto	Correto	Correto
Não	Sim	Correto	Correto	Correto
Não	Sim	Correto	Correto	Correto
Não	Sim	Errado	Errado	Correto
Não	Sim	Correto	Correto	Correto
Não	Sim	Correto	Não sei dizer	Correto
Não	Sim	Correto	Correto	Correto
Não	Sim	Correto	Errado	Correto
Não	Sim	Errado	Não sei dizer	Correto
Não	Sim	Correto	Correto	Correto
Não	Sim	Correto	Correto	Correto
Não	Sim	Correto	Correto	Correto
Não	Sim	Correto	Não sei dizer	Correto
Não	Sim	Correto	Errado	Correto
Não	Sim	Correto	Correto	Correto
Não	Sim	Correto	Errado	Correto
Não	Sim	Correto	Correto	Correto
Não	Sim	Correto	Correto	Correto
Não	Sim	Correto	Correto	Correto
Não	Sim	Correto	Correto	Correto
Não	Sim	Correto	Correto	Correto
Não	Sim	Errado	Correto	Correto
Não	Sim	Correto	Correto	Correto
Não	Sim	Correto	Correto	Correto
Não	Sim	Não sei dizer	Não sei dizer	Correto
Não	Sim	Correto	Correto	Correto

Não	Sim	Correto	Não sei dizer	Correto
Não	Sim	Correto	Correto	Correto
Não	Sim	Correto	Não sei dizer	Correto
Não	Sim	Correto	Não sei dizer	Correto
Não	Sim	Correto	Correto	Correto
Não	Sim	Correto	Não sei dizer	Correto
Não	Sim	Correto	Correto	Correto
Não	Sim	Não sei dizer	Não sei dizer	Correto
Sim	Sim	Correto	Não sei dizer	Correto
Não	Sim	Correto	Correto	Correto
Não	Sim	Correto	Correto	Correto
Não	Sim	Correto	Correto	Correto
Não	Sim	Correto	Correto	Correto
Não	Sim	Correto	Correto	Correto
Não	Sim	Correto	Correto	Correto
Não	Sim	Correto	Correto	Correto
Não	Sim	Correto	Correto	Correto
Não	Sim	Correto	Correto	Correto
Não	Sim	Correto	Não sei dizer	Correto
Não	Sim	Correto	Correto	Correto
Não	Sim	Correto	Correto	Correto
Não	Sim	Correto	Correto	Correto
Não	Sim	Correto	Errado	Correto
Não	Sim	Correto	Errado	Correto
Não	Sim	Correto	Correto	Correto
Não	Sim	Correto	Correto	Correto
Não	Sim	Correto	Correto	Correto
Não	Sim	Correto	Correto	Correto
Não	Sim	Correto	Correto	Correto
Não	Sim	Não sei dizer	Não sei dizer	Correto
Não	Sim	Correto	Correto	Correto
Não	Sim	Correto	Correto	Correto
Não	Sim	Correto	Correto	Correto
Não	Sim	Correto	Errado	Correto
Não	Sim	Correto	Correto	Correto
Não	Sim	Correto	Correto	Correto
Não	Sim	Correto	Correto	Correto
Não	Sim	Correto	Errado	Correto
Não	Sim	Correto	Correto	Correto
Não	Sim	Correto	Correto	Correto
Não	Sim	Correto	Errado	Correto
Não	Sim	Correto	Correto	Correto
Não	Sim	Correto	Não sei dizer	Correto
Não	Sim	Correto	Não sei dizer	Correto
Não	Sim	Correto	Correto	Correto
Sim	Sim	Correto	Correto	Correto
Não	Sim	Correto	Correto	Correto
Não	Sim	Correto	Correto	Correto
Não	Sim	Correto	Correto	Correto
Não	Sim	Correto	Errado	Correto
Não	Sim	Correto	Não sei dizer	Correto
Não	Sim	Correto	Correto	Correto
Não	Sim	Correto	Correto	Correto
Não	Sim	Correto	Não sei dizer	Correto

Não	Sim	Correto	Correto	Correto
Não	Sim	Errado	Correto	Correto
Não	Sim	Errado	Errado	Correto
Não	Sim	Correto	Correto	Correto
Não	Sim	Correto	Correto	Correto
Não	Sim	Correto	Correto	Correto
Não	Sim	Correto	Errado	Correto
Não	Sim	Não sei dizer	Não sei dizer	Não sei dizer
Sim	Sim	Correto	Correto	Correto
Sim	Sim	Correto	Errado	Correto
Não	Sim	Correto	Correto	Correto
Não	Sim	Correto	Correto	Correto
Não	Sim	Correto	Errado	Correto
Não	Sim	Errado	Errado	Correto
Não	Sim	Errado	Errado	Correto
Não	Sim	Correto	Errado	Correto
Não	Sim	Correto	Correto	Correto
Não	Sim	Correto	Correto	Correto
Não	Sim	Correto	Correto	Correto
Não	Sim	Correto	Errado	Correto
Não	Sim	Correto	Correto	Correto
Não	Sim	Correto	Errado	Correto
Não	Sim	Correto	Correto	Correto
Não	Sim	Correto	Correto	Correto
Não	Sim	Correto	Correto	Correto
Não	Sim	Correto	Correto	Correto
Não	Sim	Correto	Correto	Correto
Não	Sim	Correto	Não sei dizer	Correto
Não	Sim	Correto	Correto	Correto
Não	Sim	Correto	Correto	Correto
Não	Sim	Correto	Correto	Correto
Não	Sim	Errado	Correto	Correto
Não	Sim	Correto	Errado	Correto
Não	Sim	Correto	Correto	Correto
Sim	Sim	Correto	Correto	Correto
Não	Sim	Correto	Correto	Correto
Sim	Sim	Correto	Correto	Correto
Não	Sim	Correto	Errado	Correto
Não	Sim	Correto	Correto	Correto
Não	Sim	Correto	Correto	Correto
Sim	Sim	Correto	Correto	Correto
Não	Sim	Correto	Correto	Correto
Não	Sim	Correto	Correto	Correto
Não	Sim	Correto	Errado	Correto
Não	Sim	Correto	Correto	Correto
Não	Sim	Correto	Correto	Correto
Não	Sim	Errado	Errado	Correto
Não	Sim	Correto	Não sei dizer	Correto
Não	Sim	Correto	Errado	Correto
Não	Sim	Correto	Correto	Correto
Não	Sim	Correto	Correto	Correto
Não	Sim	Correto	Errado	Correto
Não	Sim	Correto	Correto	Correto
Não	Sim	Correto	Correto	Correto

Não	Sim	Errado	Errado	Correto
Sim	Sim	Correto	Correto	Correto
Não	Sim	Correto	Correto	Correto
Não	Sim	Correto	Errado	Correto
Não	Sim	Correto	Correto	Correto
Não	Sim	Correto	Errado	Correto
Não	Sim	Correto	Correto	Correto
Não	Não	Correto	Errado	Correto
Não	Sim	Correto	Errado	Correto
Não	Não	Não sei dizer	Errado	Errado
Não	Sim	Correto	Correto	Correto
Sim	Sim	Correto	Errado	Correto
Não	Sim	Correto	Errado	Correto
Não	Sim	Correto	Correto	Correto
Não	Sim	Correto	Correto	Correto
Não	Sim	Correto	Correto	Correto
Não	Sim	Correto	Correto	Correto
Não	Sim	Correto	Correto	Correto
Não	Sim	Correto	Não sei dizer	Correto
Não	Sim	Correto	Correto	Correto
Não	Sim	Errado	Errado	Correto
Sim	Sim	Correto	Correto	Correto
Sim	Sim	Correto	Correto	Correto
Sim	Sim	Correto	Correto	Correto
Não	Sim	Correto	Correto	Correto
Não	Sim	Correto	Correto	Correto
Não	Sim	Errado	Errado	Correto
Não	Sim	Correto	Correto	Correto
Não	Sim	Correto	Correto	Correto
Não	Sim	Correto	Correto	Correto
Não	Sim	Correto	Correto	Correto
Não	Sim	Correto	Correto	Correto
Não	Sim	Correto	Correto	Correto
Sim	Sim	Correto	Correto	Correto
Não	Sim	Correto	Correto	Correto
Não	Sim	Correto	Correto	Correto
Não	Sim	Correto	Correto	Correto
Não	Sim	Correto	Correto	Correto
Não	Sim	Correto	Correto	Correto
Não	Sim	Correto	Correto	Correto
Não	Sim	Correto	Correto	Correto
Não	Sim	Correto	Correto	Correto
Não	Sim	Correto	Não sei dizer	Correto
Não	Sim	Correto	Errado	Correto
Não	Sim	Correto	Correto	Correto
Não	Sim	Correto	Correto	Correto
Não	Sim	Correto	Correto	Correto
Não	Sim	Correto	Correto	Correto
Não	Não	Correto	Correto	Correto
Não	Sim	Correto	Errado	Correto
Não	Sim	Correto	Errado	Correto
Não	Sim	Correto	Não sei dizer	Correto
Não	Sim	Correto	Errado	Correto
Não	Sim	Correto	Correto	Correto

Não	Sim	Correto	Correto	Correto
Não	Sim	Correto	Correto	Correto
Não	Sim	Correto	Correto	Correto
Não	Sim	Correto	Correto	Correto
Não	Sim	Correto	Correto	Correto
Não	Sim	Correto	Não sei dizer	Correto
Não	Sim	Correto	Correto	Correto
Não	Sim	Correto	Correto	Correto
Não	Sim	Correto	Errado	Correto
Não	Sim	Correto	Errado	Correto
Não	Sim	Correto	Correto	Correto
Não	Sim	Não sei dizer	Não sei dizer	Correto
Não	Sim	Correto	Correto	Correto
Não	Sim	Correto	Não sei dizer	Correto
Não	Sim	Correto	Errado	Correto
Não	Sim	Correto	Correto	Correto
Não	Sim	Correto	Correto	Correto
Não	Sim	Correto	Não sei dizer	Correto
Não	Sim	Correto	Correto	Correto
Não	Sim	Correto	Não sei dizer	Correto
Não	Sim	Correto	Não sei dizer	Correto
Não	Sim	Correto	Correto	Correto
Não	Sim	Correto	Correto	Correto
Não	Sim	Correto	Correto	Correto
Não	Sim	Correto	Errado	Correto
Não	Sim	Correto	Errado	Correto
Não	Sim	Correto	Correto	Correto
Não	Sim	Correto	Correto	Correto
Não	Sim	Correto	Não sei dizer	Correto
Não	Sim	Correto	Não sei dizer	Correto
Sim	Sim	Errado	Errado	Correto
Não	Sim	Correto	Correto	Correto
Não	Sim	Correto	Correto	Correto
Não	Sim	Correto	Errado	Correto
Não	Sim	Correto	Correto	Correto
Não	Sim	Correto	Correto	Correto
Não	Sim	Correto	Correto	Correto
Não	Sim	Errado	Correto	Correto
Não	Sim	Correto	Correto	Correto
Não	Sim	Correto	Correto	Correto
Não	Sim	Correto	Correto	Correto
Não	Sim	Correto	Correto	Correto
Não	Sim	Correto	Não sei dizer	Correto
Não	Sim	Correto	Correto	Correto
Não	Sim	Correto	Correto	Correto
Não	Sim	Correto	Correto	Correto
Não	Sim	Correto	Correto	Correto
Não	Sim	Correto	Correto	Correto
Não	Sim	Correto	Errado	Correto
Não	Sim	Correto	Correto	Correto
Não	Sim	Correto	Correto	Correto
Não	Sim	Errado	Correto	Correto
Sim	Sim	Correto	Correto	Correto
Não	Sim	Correto	Correto	Correto

Não	Sim	Correto	Correto	Correto
Não	Sim	Correto	Correto	Correto
Não	Sim	Correto	Correto	Correto
Não	Sim	Correto	Correto	Correto
Não	Sim	Correto	Correto	Correto
Não	Sim	Correto	Errado	Correto
Não	Sim	Correto	Errado	Correto
Não	Sim	Correto	Correto	Correto
Não	Sim	Correto	Correto	Correto
Não	Sim	Correto	Errado	Correto
Não	Sim	Correto	Correto	Correto
Não	Sim	Correto	Correto	Correto
Não	Sim	Correto	Correto	Correto
Não	Sim	Correto	Correto	Correto
Não	Sim	Correto	Correto	Correto
Não	Sim	Correto	Errado	Correto
Não	Sim	Correto	Correto	Correto
Não	Sim	Correto	Correto	Correto
Não	Sim	Correto	Correto	Correto
Não	Sim	Correto	Não sei dizer	Correto
Não	Sim	Correto	Não sei dizer	Correto
Sim	Sim	Correto	Errado	Correto
Não	Sim	Correto	Correto	Correto
Não	Sim	Correto	Correto	Correto
Não	Não	Correto	Não sei dizer	Correto
Não	Sim	Correto	Correto	Correto
Não	Sim	Correto	Correto	Correto
Não	Sim	Correto	Correto	Correto
Não	Sim	Correto	Correto	Correto
Não	Sim	Correto	Correto	Correto
Não	Sim	Correto	Errado	Correto
Não	Sim	Correto	Correto	Correto
Não	Sim	Correto	Correto	Correto
Não	Sim	Correto	Não sei dizer	Correto
Não	Sim	Correto	Errado	Correto
Não	Sim	Correto	Correto	Correto
Não	Sim	Correto	Correto	Correto
Não	Sim	Correto	Errado	Correto
Não	Sim	Errado	Errado	Correto
Não	Sim	Correto	Correto	Correto
Não	Sim	Correto	Correto	Correto
Não	Sim	Correto	Correto	Correto
Não	Sim	Correto	Correto	Correto
Não	Sim	Correto	Correto	Correto
Não	Sim	Correto	Correto	Correto
Não	Sim	Correto	Errado	Correto
Não	Sim	Correto	Correto	Correto
Não	Não	Errado	Errado	Não sei dizer
Não	Sim	Correto	Correto	Correto
Não	Sim	Correto	Errado	Correto
Não	Sim	Errado	Errado	Correto
Não	Sim	Correto	Correto	Correto
Não	Sim	Correto	Errado	Correto
Não	Sim	Correto	Errado	Correto

Não	Sim	Correto	Correto	Correto
Não	Sim	Correto	Correto	Correto
Não	Sim	Correto	Correto	Correto
Não	Sim	Correto	Errado	Correto
Não	Sim	Correto	Correto	Correto
Não	Sim	Correto	Correto	Correto
Não	Sim	Correto	Correto	Correto
Não	Sim	Correto	Errado	Correto
Não	Sim	Correto	Correto	Correto
Não	Sim	Correto	Correto	Correto
Sim	Sim	Errado	Errado	Errado
Não	Sim	Correto	Correto	Correto
Não	Sim	Correto	Errado	Correto
Não	Sim	Correto	Correto	Correto
Não	Sim	Correto	Correto	Correto
Não	Sim	Correto	Correto	Correto
Não	Sim	Correto	Errado	Correto
Não	Sim	Correto	Errado	Correto
Não	Sim	Não sei dizer	Não sei dizer	Correto
Não	Sim	Correto	Correto	Correto
Não	Sim	Correto	Correto	Correto
Não	Sim	Correto	Correto	Correto
Não	Sim	Correto	Correto	Correto
Não	Sim	Correto	Correto	Errado
Não	Sim	Correto	Errado	Correto
Não	Sim	Correto	Correto	Correto
Não	Sim	Correto	Correto	Correto
Não	Sim	Correto	Correto	Correto
Não	Sim	Correto	Correto	Correto
Não	Sim	Correto	Correto	Correto
Não	Sim	Correto	Correto	Correto
Não	Sim	Correto	Correto	Correto
Não	Sim	Correto	Correto	Correto
Não	Sim	Correto	Correto	Correto
Sim	Sim	Correto	Errado	Correto
Não	Sim	Correto	Correto	Correto
Não	Sim	Correto	Correto	Correto
Não	Sim	Errado	Não sei dizer	Correto
Não	Sim	Correto	Correto	Correto
Não	Sim	Correto	Correto	Correto
Não	Sim	Correto	Correto	Correto
Sim	Sim	Correto	Correto	Correto
Não	Sim	Correto	Correto	Correto
Não	Sim	Correto	Correto	Correto
Não	Sim	Errado	Errado	Correto
Não	Sim	Correto	Errado	Correto
Não	Sim	Correto	Correto	Correto
Não	Sim	Correto	Correto	Correto
Não	Sim	Correto	Correto	Correto
Não	Sim	Correto	Errado	Correto
Não	Sim	Correto	Correto	Correto
Não	Sim	Errado	Errado	Correto
Não	Sim	Não sei dizer	Não sei dizer	Correto
Não	Sim	Correto	Correto	Errado

Não	Sim	Correto	Não sei dizer	Não sei dizer
Não	Sim	Correto	Não sei dizer	Correto
Não	Sim	Correto	Errado	Correto
Não	Sim	Correto	Correto	Correto
Não	Sim	Errado	Errado	Correto
Não	Sim	Correto	Correto	Correto
Não	Sim	Correto	Correto	Correto
Não	Sim	Correto	Errado	Correto
Não	Sim	Correto	Errado	Correto
Não	Sim	Correto	Correto	Correto
Não	Sim	Correto	Correto	Correto
Não	Sim	Correto	Correto	Correto
Não	Sim	Correto	Correto	Correto
Não	Sim	Correto	Correto	Correto
Não	Sim	Correto	Correto	Correto
Não	Sim	Correto	Errado	Correto
Não	Sim	Correto	Não sei dizer	Não sei dizer
Não	Sim	Errado	Errado	Correto

Q4	ue é a profilaxia pré-ex	Q5	Q6	Q7
Correto	Não	Não sei dizer	Não sei dizer	Não sei dizer
Errado	Não	Não sei dizer	Não sei dizer	Não sei dizer
Não sei dizer	Não	Correto	Não sei dizer	Não sei dizer
Não sei dizer	Não	Não sei dizer	Não sei dizer	Não sei dizer
Correto	Não	Não sei dizer	Não sei dizer	Não sei dizer
Não sei dizer	Não	Não sei dizer	Não sei dizer	Não sei dizer
Não sei dizer	Não	Não sei dizer	Não sei dizer	Errado
Correto	Sim	Correto	Correto	Errado
Correto	Não	Não sei dizer	Não sei dizer	Não sei dizer
Errado	Sim	Correto	Correto	Errado
Correto	Sim	Correto	Não sei dizer	Correto
Correto	Não	Não sei dizer	Não sei dizer	Não sei dizer
Correto	Sim	Errado	Correto	Correto
Correto	Sim	Correto	Correto	Não sei dizer
Não sei dizer	Não	Correto	Errado	Correto
Errado	Não	Não sei dizer	Não sei dizer	Não sei dizer
Correto	Sim	Correto	Correto	Correto
Errado	Não	Não sei dizer	Não sei dizer	Não sei dizer
Correto	Não	Não sei dizer	Correto	Correto
Correto	Sim	Correto	Correto	Correto
Errado	Não	Não sei dizer	Não sei dizer	Não sei dizer
Correto	Não	Correto	Não sei dizer	Não sei dizer
Errado	Sim	Correto	Correto	Não sei dizer
Não sei dizer	Não	Correto	Não sei dizer	Não sei dizer
Correto	Sim	Correto	Não sei dizer	Não sei dizer
Correto	Não	Correto	Correto	Não sei dizer
Correto	Sim	Correto	Não sei dizer	Correto
Não sei dizer	Não	Correto	Correto	Não sei dizer
Correto	Sim	Errado	Correto	Correto
Errado	Sim	Correto	Errado	Correto
Correto	Sim	Não sei dizer	Correto	Correto
Correto	Sim	Não sei dizer	Correto	Não sei dizer
Correto	Sim	Correto	Correto	Errado
Errado	Sim	Não sei dizer	Correto	Não sei dizer
Correto	Sim	Correto	Correto	Não sei dizer
Correto	Não	Não sei dizer	Não sei dizer	Correto
Errado	Sim	Errado	Correto	Correto
Correto	Sim	Correto	Errado	Errado
Correto	Sim	Não sei dizer	Correto	Não sei dizer
Não sei dizer	Sim	Correto	Não sei dizer	Correto
Correto	Sim	Correto	Não sei dizer	Correto
Não sei dizer	Sim	Correto	Não sei dizer	Correto
Correto	Não	Não sei dizer	Não sei dizer	Não sei dizer
Correto	Não	Correto	Correto	Errado
Não sei dizer	Sim	Correto	Não sei dizer	Correto
Correto	Não	Correto	Não sei dizer	Não sei dizer
Errado	Sim	Correto	Correto	Não sei dizer
Correto	Não	Correto	Não sei dizer	Não sei dizer
Correto	Sim	Correto	Correto	Não sei dizer
Não sei dizer	Não	Não sei dizer	Não sei dizer	Não sei dizer
Não sei dizer	Não	Não sei dizer	Não sei dizer	Não sei dizer
Não sei dizer	Não	Errado	Correto	Correto
Correto	Não	Não sei dizer	Não sei dizer	Não sei dizer

Errado	Sim	Correto	Correto	Não sei dizer
Correto	Não	Correto	Não sei dizer	Não sei dizer
Errado	Não	Não sei dizer	Não sei dizer	Não sei dizer
Correto	Não	Correto	Não sei dizer	Não sei dizer
Correto	Sim	Correto	Correto	Errado
Correto	Sim	Correto	Correto	Correto
Não sei dizer	Não	Não sei dizer	Não sei dizer	Não sei dizer
Não sei dizer	Não	Não sei dizer	Não sei dizer	Não sei dizer
Errado	Sim	Correto	Correto	Correto
Correto	Sim	Correto	Correto	Não sei dizer
Correto	Não	Não sei dizer	Não sei dizer	Não sei dizer
Correto	Sim	Correto	Correto	Errado
Não sei dizer	Não	Não sei dizer	Não sei dizer	Não sei dizer
Não sei dizer	Não	Não sei dizer	Não sei dizer	Não sei dizer
Correto	Não	Não sei dizer	Não sei dizer	Não sei dizer
Correto	Não	Correto	Não sei dizer	Correto
Correto	Não	Não sei dizer	Correto	Não sei dizer
Não sei dizer	Não	Não sei dizer	Não sei dizer	Não sei dizer
Não sei dizer	Não	Correto	Correto	Não sei dizer
Errado	Não	Não sei dizer	Não sei dizer	Não sei dizer
Não sei dizer	Sim	Correto	Não sei dizer	Não sei dizer
Correto	Sim	Correto	Correto	Correto
Não sei dizer	Não	Não sei dizer	Não sei dizer	Não sei dizer
Correto	Sim	Errado	Correto	Não sei dizer
Errado	Não	Não sei dizer	Não sei dizer	Não sei dizer
Errado	Não	Correto	Correto	Não sei dizer
Errado	Sim	Correto	Correto	Correto
Errado	Não	Não sei dizer	Não sei dizer	Não sei dizer
Não sei dizer	Não	Não sei dizer	Correto	Não sei dizer
Correto	Sim	Correto	Correto	Correto
Correto	Sim	Correto	Errado	Não sei dizer
Não sei dizer	Não	Não sei dizer	Não sei dizer	Não sei dizer
Errado	Não	Não sei dizer	Não sei dizer	Não sei dizer
Não sei dizer	Não	Não sei dizer	Não sei dizer	Não sei dizer
Errado	Não	Não sei dizer	Não sei dizer	Não sei dizer
Correto	Sim	Correto	Correto	Correto
Correto	Não	Correto	Errado	Não sei dizer
Correto	Sim	Não sei dizer	Não sei dizer	Não sei dizer
Correto	Sim	Correto	Correto	Correto
Correto	Não	Não sei dizer	Não sei dizer	Não sei dizer
Não sei dizer	Não	Não sei dizer	Não sei dizer	Correto
Errado	Não	Correto	Não sei dizer	Não sei dizer
Correto	Não	Não sei dizer	Correto	Não sei dizer
Não sei dizer	Não	Não sei dizer	Não sei dizer	Não sei dizer
Correto	Sim	Correto	Não sei dizer	Não sei dizer
Não sei dizer	Não	Não sei dizer	Não sei dizer	Não sei dizer
Errado	Não	Não sei dizer	Não sei dizer	Não sei dizer
Não sei dizer	Não	Correto	Não sei dizer	Não sei dizer
Correto	Sim	Correto	Não sei dizer	Correto
Não sei dizer	Não	Não sei dizer	Não sei dizer	Não sei dizer
Não sei dizer	Sim	Correto	Não sei dizer	Correto
Correto	Sim	Correto	Correto	Errado
Correto	Não	Não sei dizer	Correto	Correto
Não sei dizer	Não	Não sei dizer	Não sei dizer	Não sei dizer

Errado	Sim	Correto	Correto	Não sei dizer
Não sei dizer	Não	Não sei dizer	Não sei dizer	Não sei dizer
Errado	Não	Não sei dizer	Não sei dizer	Não sei dizer
Não sei dizer	Não	Não sei dizer	Não sei dizer	Não sei dizer
Não sei dizer	Não	Não sei dizer	Não sei dizer	Não sei dizer
Correto	Sim	Correto	Não sei dizer	Correto
Correto	Sim	Correto	Não sei dizer	Não sei dizer
Correto	Não	Não sei dizer	Não sei dizer	Não sei dizer
Não sei dizer	Não	Não sei dizer	Não sei dizer	Não sei dizer
Correto	Não	Não sei dizer	Não sei dizer	Não sei dizer
Correto	Não	Correto	Correto	Não sei dizer
Correto	Sim	Correto	Correto	Não sei dizer
Errado	Não	Não sei dizer	Não sei dizer	Não sei dizer
Correto	Sim	Correto	Correto	Correto
Não sei dizer	Não	Não sei dizer	Não sei dizer	Errado
Correto	Não	Não sei dizer	Não sei dizer	Não sei dizer
Correto	Sim	Errado	Correto	Correto
Não sei dizer	Não	Não sei dizer	Não sei dizer	Não sei dizer
Errado	Não	Não sei dizer	Não sei dizer	Não sei dizer
Correto	Sim	Correto	Errado	Correto
Correto	Não	Não sei dizer	Não sei dizer	Não sei dizer
Correto	Não	Não sei dizer	Não sei dizer	Não sei dizer
Errado	Sim	Correto	Correto	Correto
Correto	Sim	Correto	Não sei dizer	Não sei dizer
Não sei dizer	Não	Não sei dizer	Não sei dizer	Não sei dizer
Não sei dizer	Não	Não sei dizer	Não sei dizer	Não sei dizer
Correto	Não	Não sei dizer	Não sei dizer	Não sei dizer
Não sei dizer	Não	Não sei dizer	Não sei dizer	Não sei dizer
Correto	Sim	Correto	Correto	Correto
Correto	Sim	Correto	Não sei dizer	Errado
Correto	Sim	Correto	Correto	Correto
Correto	Não	Não sei dizer	Não sei dizer	Não sei dizer
Errado	Sim	Correto	Não sei dizer	Correto
Correto	Sim	Correto	Não sei dizer	Não sei dizer
Correto	Sim	Correto	Errado	Não sei dizer
Correto	Não	Não sei dizer	Não sei dizer	Correto
Não sei dizer	Não	Não sei dizer	Não sei dizer	Não sei dizer
Não sei dizer	Não	Não sei dizer	Não sei dizer	Não sei dizer
Errado	Sim	Correto	Correto	Não sei dizer
Correto	Não	Não sei dizer	Não sei dizer	Não sei dizer
Correto	Não	Não sei dizer	Não sei dizer	Correto
Correto	Não	Não sei dizer	Correto	Não sei dizer
Não sei dizer	Sim	Não sei dizer	Não sei dizer	Não sei dizer
Não sei dizer	Não	Não sei dizer	Não sei dizer	Não sei dizer
Correto	Sim	Correto	Não sei dizer	Correto
Errado	Sim	Errado	Não sei dizer	Errado
Correto	Não	Correto	Correto	Não sei dizer
Correto	Sim	Correto	Correto	Correto
Não sei dizer	Não	Não sei dizer	Não sei dizer	Não sei dizer
Não sei dizer	Não	Não sei dizer	Não sei dizer	Não sei dizer
Não sei dizer	Sim	Correto	Errado	Não sei dizer
Correto	Não	Errado	Não sei dizer	Não sei dizer
Não sei dizer	Não	Não sei dizer	Não sei dizer	Não sei dizer
Correto	Sim	Correto	Correto	Não sei dizer

Errado	Não	Não sei dizer	Não sei dizer	Não sei dizer
Não sei dizer	Não	Correto	Não sei dizer	Errado
Não sei dizer	Não	Não sei dizer	Não sei dizer	Não sei dizer
Correto	Sim	Correto	Correto	Correto
Não sei dizer	Não	Não sei dizer	Não sei dizer	Correto
Não sei dizer	Não	Não sei dizer	Não sei dizer	Não sei dizer
Correto	Sim	Correto	Não sei dizer	Não sei dizer
Correto	Não	Não sei dizer	Não sei dizer	Não sei dizer
Correto	Sim	Correto	Correto	Não sei dizer
Correto	Não	Não sei dizer	Não sei dizer	Não sei dizer
Correto	Não	Não sei dizer	Não sei dizer	Não sei dizer
Não sei dizer	Não	Não sei dizer	Correto	Não sei dizer
Correto	Não	Errado	Não sei dizer	Não sei dizer
Correto	Sim	Correto	Não sei dizer	Correto
Correto	Não	Não sei dizer	Não sei dizer	Não sei dizer
Errado	Não	Não sei dizer	Correto	Não sei dizer
Errado	Não	Não sei dizer	Não sei dizer	Não sei dizer
Correto	Não	Não sei dizer	Não sei dizer	Não sei dizer
Não sei dizer	Não	Correto	Correto	Não sei dizer
Correto	Sim	Correto	Não sei dizer	Não sei dizer
Correto	Não	Não sei dizer	Não sei dizer	Não sei dizer
Correto	Não	Não sei dizer	Não sei dizer	Não sei dizer
Não sei dizer	Não	Não sei dizer	Não sei dizer	Não sei dizer
Não sei dizer	Não	Correto	Não sei dizer	Correto
Correto	Sim	Correto	Correto	Não sei dizer
Correto	Não	Correto	Não sei dizer	Não sei dizer
Correto	Não	Não sei dizer	Não sei dizer	Não sei dizer
Não sei dizer	Sim	Correto	Correto	Não sei dizer
Correto	Sim	Correto	Correto	Correto
Não sei dizer	Não	Correto	Não sei dizer	Não sei dizer
Não sei dizer	Não	Não sei dizer	Não sei dizer	Não sei dizer
Correto	Sim	Correto	Correto	Correto
Não sei dizer	Não	Correto	Não sei dizer	Não sei dizer
Correto	Sim	Correto	Correto	Correto
Não sei dizer	Não	Não sei dizer	Não sei dizer	Não sei dizer
Não sei dizer	Sim	Não sei dizer	Errado	Não sei dizer
Correto	Sim	Correto	Errado	Errado
Correto	Sim	Correto	Errado	Correto
Não sei dizer	Não	Não sei dizer	Não sei dizer	Não sei dizer
Não sei dizer	Não	Não sei dizer	Não sei dizer	Não sei dizer
Não sei dizer	Sim	Não sei dizer	Correto	Não sei dizer
Não sei dizer	Não	Não sei dizer	Não sei dizer	Não sei dizer
Não sei dizer	Não	Não sei dizer	Não sei dizer	Não sei dizer
Correto	Sim	Correto	Correto	Não sei dizer
Correto	Sim	Correto	Correto	Não sei dizer
Errado	Sim	Errado	Não sei dizer	Não sei dizer
Correto	Sim	Correto	Não sei dizer	Não sei dizer
Correto	Não	Não sei dizer	Não sei dizer	Não sei dizer
Não sei dizer	Não	Não sei dizer	Não sei dizer	Não sei dizer
Correto	Sim	Correto	Correto	Correto
Não sei dizer	Não	Não sei dizer	Não sei dizer	Não sei dizer
Errado	Não	Não sei dizer	Não sei dizer	Não sei dizer
Não sei dizer	Não	Não sei dizer	Não sei dizer	Não sei dizer
Não sei dizer	Não	Não sei dizer	Não sei dizer	Não sei dizer

Correto	Não	Não sei dizer	Não sei dizer	Não sei dizer
Correto	Não	Correto	Não sei dizer	Não sei dizer
Correto	Não	Correto	Não sei dizer	Correto
Correto	Não	Não sei dizer	Não sei dizer	Não sei dizer
Correto	Não	Não sei dizer	Não sei dizer	Não sei dizer
Correto	Sim	Correto	Correto	Correto
Correto	Não	Não sei dizer	Não sei dizer	Não sei dizer
Correto	Não	Não sei dizer	Não sei dizer	Não sei dizer
Correto	Sim	Correto	Correto	Não sei dizer
Errado	Sim	Correto	Errado	Correto
Errado	Não	Não sei dizer	Não sei dizer	Não sei dizer
Não sei dizer	Não	Correto	Não sei dizer	Não sei dizer
Errado	Não	Não sei dizer	Não sei dizer	Não sei dizer
Correto	Não	Não sei dizer	Não sei dizer	Não sei dizer
Errado	Não	Não sei dizer	Não sei dizer	Não sei dizer
Não sei dizer	Não	Não sei dizer	Não sei dizer	Não sei dizer
Correto	Sim	Correto	Não sei dizer	Não sei dizer
Correto	Sim	Correto	Correto	Correto
Errado	Sim	Correto	Correto	Não sei dizer
Correto	Não	Não sei dizer	Não sei dizer	Não sei dizer
Correto	Sim	Correto	Não sei dizer	Não sei dizer
Correto	Não	Correto	Correto	Correto
Correto	Sim	Correto	Correto	Correto
Correto	Sim	Correto	Correto	Não sei dizer
Correto	Não	Não sei dizer	Não sei dizer	Não sei dizer
Correto	Sim	Correto	Correto	Errado
Correto	Não	Não sei dizer	Não sei dizer	Não sei dizer
Não sei dizer	Não	Não sei dizer	Não sei dizer	Não sei dizer
Não sei dizer	Sim	Correto	Correto	Não sei dizer
Correto	Sim	Correto	Correto	Correto
Não sei dizer	Sim	Correto	Não sei dizer	Não sei dizer
Errado	Não	Não sei dizer	Não sei dizer	Não sei dizer
Não sei dizer	Não	Não sei dizer	Não sei dizer	Correto
Correto	Não	Não sei dizer	Não sei dizer	Não sei dizer
Correto	Sim	Correto	Correto	Correto
Correto	Sim	Correto	Não sei dizer	Não sei dizer
Correto	Sim	Correto	Correto	Errado
Correto	Não	Não sei dizer	Não sei dizer	Não sei dizer
Correto	Sim	Correto	Correto	Não sei dizer
Não sei dizer	Não	Não sei dizer	Não sei dizer	Não sei dizer
Correto	Sim	Correto	Errado	Correto
Correto	Sim	Correto	Não sei dizer	Não sei dizer
Errado	Não	Não sei dizer	Não sei dizer	Não sei dizer
Correto	Sim	Correto	Correto	Não sei dizer
Errado	Não	Correto	Errado	Errado
Errado	Não	Não sei dizer	Não sei dizer	Não sei dizer
Não sei dizer	Não	Correto	Correto	Não sei dizer
Correto	Não	Não sei dizer	Não sei dizer	Não sei dizer
Correto	Sim	Correto	Não sei dizer	Correto
Correto	Sim	Correto	Correto	Correto
Correto	Sim	Correto	Não sei dizer	Correto
Correto	Não	Não sei dizer	Não sei dizer	Não sei dizer
Errado	Sim	Correto	Errado	Correto
Correto	Não	Correto	Correto	Não sei dizer

Não sei dizer	Não	Não sei dizer	Não sei dizer	Não sei dizer
Correto	Sim	Correto	Errado	Correto
Correto	Sim	Correto	Errado	Correto
Correto	Não	Correto	Não sei dizer	Não sei dizer
Correto	Não	Correto	Não sei dizer	Correto
Correto	Não	Não sei dizer	Não sei dizer	Não sei dizer
Correto	Sim	Correto	Não sei dizer	Não sei dizer
Correto	Não	Correto	Não sei dizer	Não sei dizer
Correto	Sim	Correto	Correto	Correto
Errado	Não	Errado	Correto	Errado
Correto	Não	Correto	Não sei dizer	Não sei dizer
Correto	Sim	Correto	Correto	Não sei dizer
Errado	Sim	Correto	Errado	Correto
Correto	Sim	Correto	Correto	Não sei dizer
Correto	Sim	Correto	Correto	Correto
Correto	Sim	Correto	Não sei dizer	Não sei dizer
Correto	Não	Não sei dizer	Não sei dizer	Não sei dizer
Não sei dizer	Sim	Correto	Correto	Correto
Não sei dizer	Não	Não sei dizer	Não sei dizer	Não sei dizer
Correto	Sim	Correto	Correto	Correto
Não sei dizer	Não	Não sei dizer	Não sei dizer	Não sei dizer
Correto	Sim	Correto	Correto	Correto
Correto	Sim	Correto	Correto	Não sei dizer
Correto	Sim	Correto	Correto	Correto
Correto	Não	Não sei dizer	Não sei dizer	Não sei dizer
Correto	Sim	Correto	Correto	Não sei dizer
Correto	Não	Não sei dizer	Não sei dizer	Não sei dizer
Correto	Não	Não sei dizer	Não sei dizer	Não sei dizer
Não sei dizer	Sim	Correto	Correto	Não sei dizer
Correto	Sim	Correto	Errado	Errado
Correto	Sim	Correto	Correto	Não sei dizer
Correto	Sim	Correto	Correto	Correto
Correto	Sim	Não sei dizer	Correto	Não sei dizer
Correto	Sim	Correto	Correto	Correto
Errado	Não	Não sei dizer	Não sei dizer	Não sei dizer
Correto	Sim	Correto	Errado	Não sei dizer
Correto	Não	Correto	Não sei dizer	Correto
Correto	Não	Não sei dizer	Não sei dizer	Não sei dizer
Correto	Sim	Correto	Correto	Correto
Correto	Sim	Correto	Correto	Correto
Não sei dizer	Sim	Correto	Correto	Não sei dizer
Correto	Sim	Correto	Correto	Correto
Correto	Não	Não sei dizer	Não sei dizer	Não sei dizer
Errado	Sim	Correto	Errado	Não sei dizer
Correto	Sim	Correto	Não sei dizer	Não sei dizer
Correto	Não	Não sei dizer	Não sei dizer	Não sei dizer
Não sei dizer	Sim	Correto	Correto	Não sei dizer
Correto	Sim	Correto	Correto	Correto
Correto	Não	Correto	Correto	Não sei dizer
Correto	Não	Correto	Não sei dizer	Não sei dizer
Correto	Não	Não sei dizer	Não sei dizer	Não sei dizer
Não sei dizer	Sim	Correto	Correto	Não sei dizer
Errado	Não	Não sei dizer	Não sei dizer	Não sei dizer
Correto	Não	Não sei dizer	Não sei dizer	Não sei dizer

Correto	Sim	Correto	Não sei dizer	Não sei dizer
Não sei dizer	Não	Não sei dizer	Não sei dizer	Não sei dizer
Não sei dizer	Sim	Correto	Não sei dizer	Correto
Correto	Sim	Correto	Não sei dizer	Correto
Correto	Sim	Correto	Correto	Correto
Correto	Não	Não sei dizer	Não sei dizer	Não sei dizer
Correto	Sim	Não sei dizer	Não sei dizer	Não sei dizer
Correto	Não	Não sei dizer	Não sei dizer	Não sei dizer
Correto	Não	Correto	Não sei dizer	Não sei dizer
Correto	Sim	Correto	Correto	Errado
Não sei dizer	Sim	Não sei dizer	Correto	Não sei dizer
Errado	Não	Não sei dizer	Não sei dizer	Não sei dizer
Correto	Não	Não sei dizer	Não sei dizer	Não sei dizer
Correto	Não	Não sei dizer	Não sei dizer	Não sei dizer
Errado	Não	Não sei dizer	Não sei dizer	Não sei dizer
Errado	Sim	Correto	Não sei dizer	Não sei dizer
Correto	Não	Correto	Não sei dizer	Não sei dizer
Correto	Não	Não sei dizer	Não sei dizer	Não sei dizer
Errado	Sim	Correto	Correto	Não sei dizer
Correto	Não	Não sei dizer	Não sei dizer	Não sei dizer
Não sei dizer	Não	Não sei dizer	Não sei dizer	Não sei dizer
Não sei dizer	Não	Correto	Não sei dizer	Não sei dizer
Correto	Sim	Correto	Correto	Não sei dizer
Correto	Não	Não sei dizer	Não sei dizer	Não sei dizer
Errado	Não	Não sei dizer	Não sei dizer	Não sei dizer
Não sei dizer	Não	Não sei dizer	Não sei dizer	Não sei dizer
Errado	Não	Correto	Não sei dizer	Correto
Não sei dizer	Sim	Correto	Não sei dizer	Correto
Não sei dizer	Não	Não sei dizer	Não sei dizer	Não sei dizer
Correto	Sim	Correto	Errado	Não sei dizer
Correto	Sim	Correto	Correto	Correto
Errado	Não	Não sei dizer	Não sei dizer	Não sei dizer
Não sei dizer	Sim	Correto	Não sei dizer	Correto
Correto	Sim	Correto	Não sei dizer	Não sei dizer
Correto	Sim	Correto	Correto	Não sei dizer
Correto	Não	Não sei dizer	Não sei dizer	Não sei dizer
Errado	Sim	Correto	Correto	Correto
Não sei dizer	Não	Não sei dizer	Não sei dizer	Correto
Errado	Não	Correto	Não sei dizer	Não sei dizer
Errado	Sim	Correto	Errado	Correto
Correto	Sim	Correto	Errado	Não sei dizer
Correto	Sim	Correto	Correto	Não sei dizer
Correto	Não	Não sei dizer	Não sei dizer	Não sei dizer
Errado	Sim	Correto	Não sei dizer	Não sei dizer
Correto	Sim	Correto	Correto	Não sei dizer
Correto	Sim	Não sei dizer	Não sei dizer	Não sei dizer
Correto	Sim	Correto	Correto	Não sei dizer
Correto	Sim	Correto	Correto	Não sei dizer
Errado	Não	Não sei dizer	Não sei dizer	Não sei dizer
Errado	Não	Não sei dizer	Não sei dizer	Não sei dizer
Correto	Sim	Correto	Não sei dizer	Não sei dizer
Não sei dizer	Não	Não sei dizer	Não sei dizer	Não sei dizer
Correto	Sim	Correto	Não sei dizer	Correto
Correto	Não	Não sei dizer	Não sei dizer	Correto

Correto	Sim	Correto	Correto	Correto
Errado	Não	Não sei dizer	Não sei dizer	Não sei dizer
Errado	Não	Não sei dizer	Não sei dizer	Não sei dizer
Correto	Sim	Correto	Correto	Correto
Correto	Não	Não sei dizer	Não sei dizer	Não sei dizer
Correto	Sim	Correto	Não sei dizer	Correto
Correto	Não	Não sei dizer	Não sei dizer	Não sei dizer
Não sei dizer	Sim	Correto	Correto	Não sei dizer
Correto	Não	Não sei dizer	Não sei dizer	Não sei dizer
Correto	Não	Não sei dizer	Não sei dizer	Não sei dizer
Correto	Sim	Correto	Não sei dizer	Não sei dizer
Correto	Sim	Correto	Correto	Correto
Correto	Sim	Correto	Errado	Correto
Errado	Não	Não sei dizer	Não sei dizer	Não sei dizer
Correto	Sim	Correto	Não sei dizer	Correto
Correto	Sim	Correto	Errado	Correto
Correto	Sim	Correto	Não sei dizer	Não sei dizer
Não sei dizer	Não	Não sei dizer	Não sei dizer	Não sei dizer
Correto	Sim	Correto	Não sei dizer	Correto
Não sei dizer	Não	Correto	Não sei dizer	Não sei dizer
Errado	Não	Errado	Correto	Não sei dizer
Correto	Sim	Não sei dizer	Correto	Não sei dizer
Correto	Não	Não sei dizer	Não sei dizer	Não sei dizer
Correto	Não	Não sei dizer	Não sei dizer	Não sei dizer
Errado	Sim	Correto	Não sei dizer	Não sei dizer
Não sei dizer	Não	Não sei dizer	Não sei dizer	Não sei dizer
Errado	Não	Correto	Correto	Correto
Errado	Sim	Não sei dizer	Não sei dizer	Não sei dizer
Correto	Sim	Correto	Não sei dizer	Correto
Errado	Não	Não sei dizer	Não sei dizer	Não sei dizer
Não sei dizer	Não	Não sei dizer	Não sei dizer	Não sei dizer
Errado	Não	Correto	Correto	Correto
Errado	Não	Não sei dizer	Não sei dizer	Não sei dizer
Não sei dizer	Não	Não sei dizer	Não sei dizer	Não sei dizer
Não sei dizer	Não	Não sei dizer	Não sei dizer	Não sei dizer
Errado	Sim	Correto	Correto	Correto
Correto	Sim	Correto	Não sei dizer	Não sei dizer
Errado	Não	Não sei dizer	Não sei dizer	Não sei dizer
Não sei dizer	Não	Não sei dizer	Não sei dizer	Não sei dizer
Correto	Sim	Correto	Correto	Não sei dizer
Correto	Sim	Correto	Correto	Correto
Correto	Não	Não sei dizer	Não sei dizer	Não sei dizer
Errado	Não	Não sei dizer	Não sei dizer	Não sei dizer
Não sei dizer	Não	Não sei dizer	Não sei dizer	Não sei dizer
Correto	Sim	Correto	Correto	Não sei dizer
Errado	Não	Não sei dizer	Não sei dizer	Não sei dizer
Correto	Sim	Correto	Não sei dizer	Não sei dizer
Não sei dizer	Não	Não sei dizer	Não sei dizer	Não sei dizer
Não sei dizer	Não	Não sei dizer	Não sei dizer	Não sei dizer
Não sei dizer	Não	Não sei dizer	Não sei dizer	Não sei dizer
Não sei dizer	Sim	Correto	Não sei dizer	Não sei dizer
Errado	Sim	Correto	Correto	Errado
Errado	Não	Não sei dizer	Não sei dizer	Não sei dizer
Errado	Sim	Não sei dizer	Não sei dizer	Errado

Errado	Sim	Correto	Correto	Errado
Correto	Sim	Correto	Correto	Correto
Correto	Sim	Correto	Correto	Correto
Errado	Não	Não sei dizer	Não sei dizer	Não sei dizer
Correto	Sim	Não sei dizer	Não sei dizer	Não sei dizer
Correto	Sim	Correto	Não sei dizer	Errado
Correto	Sim	Correto	Correto	Correto
Correto	Sim	Correto	Correto	Não sei dizer
Não sei dizer	Não	Não sei dizer	Não sei dizer	Não sei dizer
Correto	Não	Correto	Correto	Não sei dizer
Correto	Sim	Correto	Errado	Não sei dizer
Errado	Não	Não sei dizer	Não sei dizer	Não sei dizer
Errado	Sim	Não sei dizer	Correto	Correto
Correto	Sim	Correto	Correto	Correto
Correto	Sim	Correto	Errado	Correto
Correto	Sim	Correto	Correto	Correto
Correto	Sim	Correto	Correto	Correto
Errado	Não	Correto	Correto	Correto
Não sei dizer	Não	Não sei dizer	Não sei dizer	Não sei dizer
Correto	Sim	Correto	Correto	Correto
Errado	Sim	Correto	Correto	Errado
Não sei dizer	Não	Não sei dizer	Não sei dizer	Não sei dizer
Correto	Sim	Correto	Errado	Errado
Errado	Não	Não sei dizer	Não sei dizer	Não sei dizer
Não sei dizer	Não	Não sei dizer	Não sei dizer	Não sei dizer
Correto	Sim	Correto	Correto	Errado
Errado	Sim	Correto	Correto	Correto
Errado	Não	Correto	Correto	Errado
Correto	Sim	Correto	Errado	Correto
Correto	Sim	Correto	Errado	Correto
Correto	Sim	Correto	Correto	Correto
Errado	Não	Não sei dizer	Não sei dizer	Não sei dizer
Errado	Não	Correto	Correto	Correto
Não sei dizer	Sim	Correto	Não sei dizer	Errado
Correto	Sim	Correto	Correto	Correto
Correto	Sim	Correto	Correto	Errado
Correto	Não	Não sei dizer	Não sei dizer	Não sei dizer
Errado	Não	Correto	Correto	Errado
Errado	Sim	Correto	Correto	Errado
Correto	Sim	Correto	Errado	Correto
Correto	Sim	Correto	Correto	Não sei dizer
Correto	Sim	Correto	Correto	Correto
Correto	Não	Errado	Correto	Correto
Correto	Não	Não sei dizer	Não sei dizer	Não sei dizer
Errado	Não	Não sei dizer	Não sei dizer	Errado
Não sei dizer	Não	Não sei dizer	Não sei dizer	Não sei dizer
Não sei dizer	Não	Não sei dizer	Não sei dizer	Não sei dizer
Errado	Não	Não sei dizer	Não sei dizer	Não sei dizer
Errado	Não	Não sei dizer	Não sei dizer	Não sei dizer
Errado	Sim	Correto	Correto	Correto
Não sei dizer	Não	Não sei dizer	Não sei dizer	Não sei dizer
Errado	Não	Não sei dizer	Não sei dizer	Não sei dizer
Não sei dizer	Não	Não sei dizer	Não sei dizer	Não sei dizer
Não sei dizer	Não	Não sei dizer	Não sei dizer	Não sei dizer

Não sei dizer	Não	Não sei dizer	Não sei dizer	Não sei dizer
Correto	Não	Não sei dizer	Não sei dizer	Não sei dizer
Correto	Não	Não sei dizer	Não sei dizer	Não sei dizer
Errado	Sim	Correto	Correto	Não sei dizer
Não sei dizer	Não	Não sei dizer	Não sei dizer	Não sei dizer
Correto	Não	Correto	Não sei dizer	Não sei dizer
Errado	Não	Não sei dizer	Correto	Não sei dizer
Errado	Não	Errado	Correto	Errado
Correto	Sim	Correto	Correto	Não sei dizer
Correto	Não	Não sei dizer	Não sei dizer	Não sei dizer
Correto	Sim	Correto	Correto	Correto
Errado	Não	Não sei dizer	Não sei dizer	Não sei dizer
Não sei dizer	Não	Não sei dizer	Não sei dizer	Não sei dizer
Não sei dizer	Não	Não sei dizer	Não sei dizer	Não sei dizer
Correto	Sim	Correto	Correto	Correto
Não sei dizer	Não	Não sei dizer	Não sei dizer	Não sei dizer
Correto	Sim	Não sei dizer	Não sei dizer	Não sei dizer
Não sei dizer	Não	Não sei dizer	Não sei dizer	Correto

Q8	Q9	Q10	que é a profilaxia pós-e	Q11
Não sei dizer	Não sei dizer	Não sei dizer	Não	Não sei dizer
Correto	Correto	Não sei dizer	Não	Não sei dizer
Não sei dizer	Não sei dizer	Não sei dizer	Não	Não sei dizer
Não sei dizer	Não sei dizer	Correto	Não	Não sei dizer
Não sei dizer	Correto	Correto	Sim	Correto
Não sei dizer	Não sei dizer	Correto	Não	Não sei dizer
Correto	Correto	Correto	Não	Não sei dizer
Correto	Correto	Correto	Sim	Correto
Não sei dizer	Não sei dizer	Correto	Não	Correto
Correto	Correto	Correto	Sim	Errado
Não sei dizer	Não sei dizer	Errado	Sim	Correto
Errado	Correto	Errado	Não	Não sei dizer
Não sei dizer	Correto	Correto	Sim	Correto
Correto	Correto	Correto	Sim	Correto
Não sei dizer	Correto	Errado	Não	Correto
Não sei dizer	Não sei dizer	Não sei dizer	Não	Não sei dizer
Correto	Correto	Correto	Sim	Correto
Não sei dizer	Não sei dizer	Não sei dizer	Não	Não sei dizer
Correto	Correto	Não sei dizer	Não	Não sei dizer
Correto	Correto	Correto	Sim	Correto
Correto	Correto	Correto	Não	Não sei dizer
Correto	Não sei dizer	Correto	Não	Correto
Correto	Correto	Correto	Sim	Correto
Não sei dizer	Correto	Correto	Sim	Correto
Correto	Correto	Correto	Sim	Correto
Correto	Correto	Correto	Não	Correto
Correto	Correto	Correto	Sim	Correto
Não sei dizer	Não sei dizer	Correto	Não	Não sei dizer
Errado	Correto	Correto	Sim	Correto
Errado	Correto	Correto	Sim	Correto
Correto	Correto	Correto	Não	Não sei dizer
Correto	Correto	Correto	Sim	Correto
Correto	Correto	Correto	Sim	Correto
Errado	Não sei dizer	Correto	Sim	Correto
Não sei dizer	Correto	Correto	Sim	Correto
Correto	Correto	Correto	Não	Não sei dizer
Correto	Correto	Correto	Sim	Correto
Correto	Correto	Correto	Sim	Correto
Correto	Não sei dizer	Correto	Sim	Correto
Não sei dizer	Correto	Correto	Sim	Correto
Correto	Correto	Correto	Sim	Correto
Não sei dizer	Correto	Correto	Sim	Correto
Não sei dizer	Não sei dizer	Correto	Não	Não sei dizer
Correto	Errado	Correto	Não	Errado
Não sei dizer	Correto	Correto	Sim	Correto
Correto	Não sei dizer	Correto	Não	Correto
Correto	Não sei dizer	Correto	Sim	Correto
Não sei dizer	Correto	Não sei dizer	Não	Correto
Correto	Correto	Correto	Sim	Correto
Não sei dizer	Não sei dizer	Não sei dizer	Não	Não sei dizer
Não sei dizer	Não sei dizer	Não sei dizer	Não	Não sei dizer
Correto	Correto	Correto	Não	Não sei dizer
Não sei dizer	Não sei dizer	Não sei dizer	Não	Não sei dizer

Não sei dizer	Correto	Errado	Sim	Correto
Correto	Não sei dizer	Correto	Não	Não sei dizer
Não sei dizer	Errado	Não sei dizer	Não	Não sei dizer
Correto	Não sei dizer	Correto	Não	Não sei dizer
Não sei dizer	Correto	Correto	Sim	Correto
Correto	Correto	Correto	Sim	Correto
Não sei dizer	Não sei dizer	Não sei dizer	Não	Não sei dizer
Não sei dizer	Não sei dizer	Não sei dizer	Sim	Não sei dizer
Correto	Correto	Correto	Sim	Correto
Correto	Correto	Correto	Sim	Correto
Errado	Não sei dizer	Correto	Não	Correto
Correto	Correto	Correto	Sim	Correto
Não sei dizer	Não sei dizer	Não sei dizer	Não	Não sei dizer
Correto	Não sei dizer	Não sei dizer	Sim	Correto
Não sei dizer	Não sei dizer	Não sei dizer	Não	Correto
Não sei dizer	Não sei dizer	Correto	Não	Errado
Não sei dizer	Não sei dizer	Correto	Sim	Correto
Não sei dizer	Não sei dizer	Não sei dizer	Não	Não sei dizer
Não sei dizer	Não sei dizer	Errado	Não	Correto
Não sei dizer	Não sei dizer	Correto	Não	Não sei dizer
Não sei dizer	Correto	Correto	Sim	Correto
Correto	Correto	Correto	Não	Correto
Correto	Não sei dizer	Correto	Não	Não sei dizer
Correto	Correto	Correto	Sim	Correto
Não sei dizer	Não sei dizer	Correto	Sim	Correto
Não sei dizer	Correto	Errado	Sim	Correto
Correto	Correto	Correto	Sim	Correto
Não sei dizer	Não sei dizer	Não sei dizer	Não	Não sei dizer
Correto	Correto	Correto	Não	Correto
Correto	Correto	Errado	Sim	Correto
Não sei dizer	Não sei dizer	Não sei dizer	Sim	Correto
Correto	Não sei dizer	Errado	Sim	Correto
Correto	Não sei dizer	Correto	Não	Correto
Não sei dizer	Não sei dizer	Correto	Não	Não sei dizer
Não sei dizer	Não sei dizer	Não sei dizer	Não	Não sei dizer
Não sei dizer	Correto	Correto	Sim	Correto
Errado	Não sei dizer	Correto	Sim	Correto
Correto	Correto	Correto	Sim	Não sei dizer
Não sei dizer	Correto	Correto	Sim	Correto
Não sei dizer	Não sei dizer	Correto	Não	Não sei dizer
Não sei dizer	Não sei dizer	Correto	Sim	Correto
Não sei dizer	Correto	Não sei dizer	Sim	Correto
Correto	Não sei dizer	Correto	Não	Correto
Não sei dizer	Não sei dizer	Não sei dizer	Não	Não sei dizer
Não sei dizer	Correto	Correto	Sim	Correto
Não sei dizer	Não sei dizer	Não sei dizer	Não	Correto
Correto	Correto	Correto	Não	Não sei dizer
Não sei dizer	Não sei dizer	Correto	Não	Errado
Não sei dizer	Correto	Correto	Sim	Correto
Correto	Não sei dizer	Não sei dizer	Não	Não sei dizer
Correto	Correto	Não sei dizer	Sim	Correto
Correto	Não sei dizer	Correto	Sim	Correto
Não sei dizer	Correto	Correto	Não	Correto
Não sei dizer	Não sei dizer	Não sei dizer	Não	Não sei dizer

Correto	Correto	Correto	Sim	Correto
Não sei dizer	Não sei dizer	Não sei dizer	Não	Não sei dizer
Não sei dizer	Não sei dizer	Não sei dizer	Não	Não sei dizer
Não sei dizer	Correto	Errado	Não	Não sei dizer
Não sei dizer	Não sei dizer	Correto	Não	Não sei dizer
Não sei dizer	Correto	Errado	Sim	Correto
Correto	Correto	Errado	Sim	Correto
Correto	Correto	Não sei dizer	Não	Não sei dizer
Não sei dizer	Não sei dizer	Não sei dizer	Não	Correto
Não sei dizer	Não sei dizer	Não sei dizer	Não	Não sei dizer
Não sei dizer	Correto	Errado	Não	Não sei dizer
Correto	Correto	Correto	Sim	Correto
Não sei dizer	Não sei dizer	Não sei dizer	Não	Não sei dizer
Correto	Correto	Não sei dizer	Sim	Correto
Não sei dizer	Não sei dizer	Correto	Não	Não sei dizer
Não sei dizer	Não sei dizer	Não sei dizer	Não	Não sei dizer
Errado	Correto	Correto	Sim	Correto
Não sei dizer	Não sei dizer	Não sei dizer	Não	Não sei dizer
Não sei dizer	Não sei dizer	Não sei dizer	Não	Não sei dizer
Correto	Correto	Correto	Sim	Correto
Não sei dizer	Não sei dizer	Correto	Não	Não sei dizer
Não sei dizer	Correto	Correto	Sim	Errado
Correto	Correto	Correto	Sim	Correto
Correto	Não sei dizer	Correto	Sim	Correto
Não sei dizer	Não sei dizer	Não sei dizer	Não	Não sei dizer
Não sei dizer	Não sei dizer	Não sei dizer	Não	Não sei dizer
Não sei dizer	Não sei dizer	Não sei dizer	Não	Não sei dizer
Não sei dizer	Não sei dizer	Não sei dizer	Não	Não sei dizer
Não sei dizer	Correto	Não sei dizer	Sim	Correto
Correto	Correto	Correto	Sim	Correto
Correto	Correto	Correto	Sim	Correto
Não sei dizer	Correto	Não sei dizer	Não	Não sei dizer
Correto	Correto	Correto	Sim	Errado
Não sei dizer	Correto	Correto	Sim	Correto
Não sei dizer	Correto	Não sei dizer	Sim	Correto
Não sei dizer	Não sei dizer	Não sei dizer	Não	Correto
Não sei dizer	Não sei dizer	Não sei dizer	Não	Não sei dizer
Errado	Correto	Correto	Sim	Correto
Correto	Correto	Correto	Sim	Correto
Não sei dizer	Não sei dizer	Não sei dizer	Sim	Correto
Não sei dizer	Correto	Correto	Não	Não sei dizer
Não sei dizer	Não sei dizer	Não sei dizer	Sim	Correto
Correto	Correto	Não sei dizer	Sim	Não sei dizer
Não sei dizer	Não sei dizer	Não sei dizer	Não	Não sei dizer
Correto	Correto	Correto	Sim	Correto
Não sei dizer	Não sei dizer	Não sei dizer	Sim	Errado
Não sei dizer	Não sei dizer	Correto	Sim	Correto
Errado	Correto	Correto	Sim	Correto
Não sei dizer	Não sei dizer	Não sei dizer	Não	Não sei dizer
Não sei dizer	Não sei dizer	Não sei dizer	Não	Não sei dizer
Não sei dizer	Correto	Correto	Sim	Correto
Não sei dizer	Não sei dizer	Correto	Não	Não sei dizer
Não sei dizer	Não sei dizer	Não sei dizer	Não	Não sei dizer
Correto	Correto	Correto	Sim	Correto

Não sei dizer	Não sei dizer	Não sei dizer	Não	Não sei dizer
Não sei dizer	Não sei dizer	Errado	Não	Correto
Correto	Correto	Correto	Não	Não sei dizer
Correto	Correto	Correto	Sim	Correto
Não sei dizer	Não sei dizer	Correto	Não	Não sei dizer
Correto	Correto	Correto	Sim	Correto
Correto	Correto	Correto	Sim	Correto
Não sei dizer	Não sei dizer	Não sei dizer	Não	Não sei dizer
Correto	Correto	Correto	Sim	Correto
Não sei dizer	Não sei dizer	Não sei dizer	Não	Não sei dizer
Não sei dizer	Não sei dizer	Correto	Não	Não sei dizer
Não sei dizer	Não sei dizer	Correto	Não	Não sei dizer
Correto	Não sei dizer	Correto	Não	Correto
Correto	Correto	Correto	Sim	Correto
Correto	Correto	Correto	Sim	Correto
Correto	Não sei dizer	Correto	Sim	Correto
Correto	Correto	Correto	Não	Não sei dizer
Não sei dizer	Não sei dizer	Não sei dizer	Não	Não sei dizer
Não sei dizer	Não sei dizer	Correto	Não	Correto
Correto	Não sei dizer	Não sei dizer	Não	Correto
Correto	Correto	Correto	Não	Não sei dizer
Correto	Não sei dizer	Não sei dizer	Não	Não sei dizer
Não sei dizer	Não sei dizer	Não sei dizer	Não	Não sei dizer
Não sei dizer	Correto	Não sei dizer	Não	Correto
Não sei dizer	Correto	Correto	Sim	Correto
Errado	Correto	Não sei dizer	Sim	Correto
Não sei dizer	Não sei dizer	Não sei dizer	Não	Não sei dizer
Não sei dizer	Correto	Correto	Sim	Correto
Correto	Correto	Correto	Sim	Correto
Não sei dizer	Correto	Correto	Não	Correto
Não sei dizer	Não sei dizer	Não sei dizer	Não	Não sei dizer
Não sei dizer	Correto	Correto	Sim	Não sei dizer
Correto	Correto	Correto	Não	Correto
Correto	Correto	Correto	Sim	Correto
Correto	Não sei dizer	Não sei dizer	Não	Não sei dizer
Não sei dizer	Correto	Errado	Sim	Não sei dizer
Correto	Errado	Correto	Sim	Correto
Não sei dizer	Correto	Correto	Sim	Correto
Não sei dizer	Não sei dizer	Não sei dizer	Não	Não sei dizer
Não sei dizer	Não sei dizer	Não sei dizer	Não	Não sei dizer
Correto	Correto	Correto	Não	Correto
Não sei dizer	Não sei dizer	Não sei dizer	Não	Não sei dizer
Correto	Não sei dizer	Correto	Não	Não sei dizer
Não sei dizer	Não sei dizer	Correto	Sim	Correto
Correto	Correto	Correto	Sim	Correto
Correto	Correto	Correto	Sim	Correto
Não sei dizer	Correto	Não sei dizer	Sim	Correto
Não sei dizer	Não sei dizer	Não sei dizer	Não	Não sei dizer
Não sei dizer	Correto	Não sei dizer	Não	Correto
Correto	Correto	Correto	Sim	Correto
Não sei dizer	Não sei dizer	Não sei dizer	Não	Não sei dizer
Não sei dizer	Não sei dizer	Não sei dizer	Não	Correto
Não sei dizer	Não sei dizer	Não sei dizer	Não	Não sei dizer
Não sei dizer	Não sei dizer	Não sei dizer	Não	Não sei dizer

Não sei dizer	Não sei dizer	Não sei dizer	Não	Não sei dizer
Correto	Não sei dizer	Correto	Não	Correto
Correto	Correto	Correto	Não	Correto
Não sei dizer	Não sei dizer	Não sei dizer	Não	Não sei dizer
Não sei dizer	Correto	Não sei dizer	Não	Não sei dizer
Errado	Errado	Errado	Sim	Correto
Não sei dizer	Não sei dizer	Não sei dizer	Não	Não sei dizer
Não sei dizer	Não sei dizer	Não sei dizer	Não	Não sei dizer
Correto	Correto	Correto	Sim	Correto
Correto	Correto	Errado	Sim	Correto
Não sei dizer	Não sei dizer	Não sei dizer	Sim	Correto
Correto	Não sei dizer	Correto	Sim	Correto
Correto	Correto	Errado	Não	Não sei dizer
Não sei dizer	Não sei dizer	Não sei dizer	Não	Não sei dizer
Correto	Não sei dizer	Correto	Sim	Correto
Correto	Correto	Correto	Não	Não sei dizer
Correto	Correto	Correto	Sim	Errado
Correto	Correto	Correto	Sim	Correto
Correto	Correto	Correto	Sim	Correto
Não sei dizer	Correto	Correto	Não	Não sei dizer
Correto	Não sei dizer	Correto	Não	Correto
Errado	Correto	Correto	Não	Correto
Correto	Correto	Correto	Sim	Correto
Não sei dizer	Correto	Correto	Sim	Correto
Não sei dizer	Não sei dizer	Não sei dizer	Não	Não sei dizer
Correto	Não sei dizer	Correto	Sim	Correto
Não sei dizer	Não sei dizer	Não sei dizer	Não	Não sei dizer
Não sei dizer	Não sei dizer	Correto	Não	Não sei dizer
Correto	Não sei dizer	Errado	Não	Não sei dizer
Correto	Correto	Correto	Sim	Correto
Correto	Correto	Correto	Sim	Correto
Não sei dizer	Não sei dizer	Não sei dizer	Não	Não sei dizer
Não sei dizer	Não sei dizer	Correto	Não	Não sei dizer
Não sei dizer	Não sei dizer	Correto	Sim	Errado
Correto	Correto	Correto	Sim	Correto
Correto	Correto	Correto	Não	Não sei dizer
Correto	Correto	Correto	Sim	Correto
Não sei dizer	Não sei dizer	Não sei dizer	Não	Não sei dizer
Não sei dizer	Correto	Correto	Sim	Correto
Não sei dizer	Não sei dizer	Não sei dizer	Sim	Correto
Correto	Correto	Correto	Sim	Correto
Correto	Correto	Correto	Sim	Correto
Não sei dizer	Não sei dizer	Não sei dizer	Não	Não sei dizer
Errado	Correto	Correto	Sim	Correto
Errado	Não sei dizer	Correto	Não	Correto
Não sei dizer	Correto	Correto	Não	Não sei dizer
Não sei dizer	Não sei dizer	Correto	Não	Correto
Não sei dizer	Não sei dizer	Não sei dizer	Não	Não sei dizer
Correto	Correto	Correto	Sim	Correto
Correto	Correto	Correto	Sim	Correto
Correto	Não sei dizer	Correto	Sim	Correto
Não sei dizer	Correto	Não sei dizer	Sim	Não sei dizer
Não sei dizer	Correto	Correto	Sim	Correto
Não sei dizer	Não sei dizer	Não sei dizer	Não	Correto

Não sei dizer	Não sei dizer	Não sei dizer	Não	Não sei dizer
Não sei dizer	Correto	Correto	Sim	Correto
Correto	Correto	Correto	Sim	Correto
Não sei dizer	Não sei dizer	Não sei dizer	Não	Correto
Não sei dizer	Correto	Correto	Sim	Correto
Não sei dizer	Não sei dizer	Não sei dizer	Sim	Correto
Correto	Correto	Correto	Sim	Correto
Errado	Correto	Correto	Não	Correto
Correto	Correto	Correto	Sim	Correto
Correto	Correto	Errado	Não	Não sei dizer
Não sei dizer	Não sei dizer	Correto	Sim	Correto
Correto	Correto	Correto	Sim	Correto
Correto	Errado	Correto	Sim	Correto
Correto	Correto	Correto	Sim	Correto
Correto	Correto	Correto	Sim	Correto
Correto	Correto	Correto	Sim	Correto
Não sei dizer	Não sei dizer	Correto	Não	Não sei dizer
Correto	Correto	Correto	Sim	Correto
Não sei dizer	Não sei dizer	Correto	Sim	Correto
Correto	Correto	Correto	Não	Não sei dizer
Não sei dizer	Não sei dizer	Não sei dizer	Não	Não sei dizer
Correto	Correto	Correto	Sim	Correto
Correto	Correto	Correto	Sim	Correto
Correto	Correto	Não sei dizer	Sim	Correto
Não sei dizer	Não sei dizer	Não sei dizer	Sim	Correto
Correto	Correto	Correto	Sim	Correto
Correto	Não sei dizer	Não sei dizer	Não	Não sei dizer
Não sei dizer	Não sei dizer	Não sei dizer	Não	Não sei dizer
Correto	Não sei dizer	Correto	Não	Não sei dizer
Correto	Correto	Correto	Sim	Correto
Correto	Correto	Correto	Sim	Correto
Errado	Correto	Correto	Não	Não sei dizer
Correto	Correto	Correto	Sim	Correto
Correto	Correto	Correto	Sim	Correto
Não sei dizer	Não sei dizer	Não sei dizer	Não	Não sei dizer
Correto	Correto	Correto	Sim	Correto
Não sei dizer	Correto	Errado	Não	Correto
Não sei dizer	Não sei dizer	Não sei dizer	Não	Não sei dizer
Correto	Correto	Correto	Sim	Correto
Correto	Correto	Correto	Sim	Correto
Correto	Não sei dizer	Correto	Sim	Não sei dizer
Correto	Correto	Correto	Sim	Correto
Não sei dizer	Não sei dizer	Não sei dizer	Sim	Correto
Não sei dizer	Não sei dizer	Correto	Não	Não sei dizer
Correto	Não sei dizer	Correto	Sim	Correto
Não sei dizer	Correto	Correto	Não	Não sei dizer
Correto	Correto	Correto	Sim	Correto
Correto	Correto	Correto	Sim	Correto
Não sei dizer	Correto	Correto	Não	Correto
Não sei dizer	Não sei dizer	Correto	Não	Correto
Não sei dizer	Não sei dizer	Não sei dizer	Não	Não sei dizer
Correto	Correto	Correto	Sim	Correto
Não sei dizer	Não sei dizer	Não sei dizer	Não	Não sei dizer
Correto	Não sei dizer	Correto	Sim	Correto

Correto	Não sei dizer	Não sei dizer	Sim	Correto
Não sei dizer	Não sei dizer	Não sei dizer	Não	Não sei dizer
Errado	Correto	Correto	Sim	Correto
Correto	Correto	Não sei dizer	Não	Correto
Correto	Correto	Correto	Sim	Correto
Não sei dizer	Não sei dizer	Correto	Não	Não sei dizer
Correto	Correto	Correto	Não	Não sei dizer
Não sei dizer	Não sei dizer	Não sei dizer	Não	Não sei dizer
Não sei dizer	Não sei dizer	Não sei dizer	Não	Correto
Correto	Errado	Correto	Sim	Correto
Não sei dizer	Correto	Correto	Sim	Não sei dizer
Não sei dizer	Não sei dizer	Não sei dizer	Não	Não sei dizer
Não sei dizer	Não sei dizer	Não sei dizer	Não	Não sei dizer
Não sei dizer	Correto	Correto	Não	Correto
Não sei dizer	Não sei dizer	Não sei dizer	Não	Não sei dizer
Errado	Não sei dizer	Correto	Não	Correto
Não sei dizer	Correto	Não sei dizer	Sim	Correto
Correto	Não sei dizer	Correto	Sim	Correto
Não sei dizer	Não sei dizer	Correto	Sim	Correto
Não sei dizer	Não sei dizer	Correto	Não	Não sei dizer
Não sei dizer	Não sei dizer	Não sei dizer	Não	Não sei dizer
Não sei dizer	Não sei dizer	Correto	Não	Correto
Correto	Correto	Correto	Sim	Correto
Não sei dizer	Não sei dizer	Não sei dizer	Não	Não sei dizer
Correto	Não sei dizer	Não sei dizer	Não	Não sei dizer
Não sei dizer	Não sei dizer	Não sei dizer	Não	Não sei dizer
Errado	Não sei dizer	Correto	Não	Correto
Correto	Não sei dizer	Correto	Sim	Correto
Não sei dizer	Não sei dizer	Não sei dizer	Não	Não sei dizer
Correto	Correto	Correto	Não	Não sei dizer
Correto	Correto	Correto	Sim	Correto
Não sei dizer	Não sei dizer	Não sei dizer	Não	Correto
Correto	Correto	Correto	Sim	Correto
Não sei dizer	Correto	Correto	Sim	Correto
Correto	Correto	Correto	Sim	Correto
Não sei dizer	Não sei dizer	Não sei dizer	Não	Não sei dizer
Correto	Correto	Correto	Sim	Correto
Não sei dizer	Correto	Não sei dizer	Não	Correto
Não sei dizer	Não sei dizer	Correto	Não	Correto
Errado	Correto	Correto	Sim	Correto
Correto	Correto	Não sei dizer	Sim	Correto
Correto	Correto	Correto	Sim	Correto
Não sei dizer	Não sei dizer	Correto	Não	Não sei dizer
Correto	Correto	Correto	Sim	Correto
Correto	Correto	Correto	Sim	Correto
Não sei dizer	Correto	Correto	Sim	Errado
Correto	Correto	Correto	Sim	Correto
Não sei dizer	Correto	Correto	Sim	Não sei dizer
Não sei dizer	Não sei dizer	Não sei dizer	Não	Não sei dizer
Não sei dizer	Não sei dizer	Não sei dizer	Não	Não sei dizer
Correto	Correto	Correto	Sim	Correto
Não sei dizer	Não sei dizer	Correto	Sim	Correto
Não sei dizer	Correto	Correto	Sim	Correto
Não sei dizer	Correto	Correto	Não	Não sei dizer

Correto	Correto	Correto	Sim	Correto
Correto	Não sei dizer	Correto	Não	Correto
Não sei dizer	Não sei dizer	Não sei dizer	Não	Não sei dizer
Correto	Correto	Correto	Sim	Correto
Não sei dizer	Não sei dizer	Não sei dizer	Não	Não sei dizer
Correto	Correto	Correto	Sim	Correto
Não sei dizer	Não sei dizer	Não sei dizer	Não	Não sei dizer
Correto	Não sei dizer	Correto	Sim	Correto
Não sei dizer	Não sei dizer	Não sei dizer	Não	Não sei dizer
Não sei dizer	Não sei dizer	Não sei dizer	Não	Não sei dizer
Correto	Correto	Não sei dizer	Sim	Correto
Correto	Não sei dizer	Correto	Sim	Correto
Não sei dizer	Correto	Correto	Sim	Correto
Não sei dizer	Não sei dizer	Correto	Não	Não sei dizer
Não sei dizer	Correto	Correto	Sim	Errado
Errado	Correto	Errado	Sim	Correto
Correto	Não sei dizer	Correto	Sim	Correto
Errado	Não sei dizer	Correto	Não	Correto
Não sei dizer	Correto	Não sei dizer	Sim	Correto
Não sei dizer	Não sei dizer	Não sei dizer	Não	Correto
Não sei dizer	Não sei dizer	Correto	Não	Não sei dizer
Correto	Correto	Não sei dizer	Sim	Correto
Não sei dizer	Não sei dizer	Correto	Não	Não sei dizer
Não sei dizer	Correto	Correto	Sim	Correto
Não sei dizer	Correto	Correto	Não	Não sei dizer
Não sei dizer	Não sei dizer	Não sei dizer	Não	Não sei dizer
Não sei dizer	Não sei dizer	Correto	Sim	Correto
Correto	Não sei dizer	Correto	Não	Não sei dizer
Correto	Correto	Errado	Sim	Correto
Não sei dizer	Não sei dizer	Não sei dizer	Não	Não sei dizer
Não sei dizer	Não sei dizer	Não sei dizer	Não	Não sei dizer
Correto	Correto	Correto	Não	Errado
Não sei dizer	Não sei dizer	Não sei dizer	Não	Não sei dizer
Não sei dizer	Não sei dizer	Não sei dizer	Não	Não sei dizer
Não sei dizer	Não sei dizer	Correto	Não	Não sei dizer
Errado	Não sei dizer	Correto	Sim	Não sei dizer
Não sei dizer	Correto	Correto	Sim	Correto
Não sei dizer	Não sei dizer	Não sei dizer	Não	Não sei dizer
Não sei dizer	Correto	Correto	Não	Não sei dizer
Correto	Correto	Correto	Sim	Correto
Correto	Correto	Correto	Sim	Correto
Não sei dizer	Não sei dizer	Não sei dizer	Não	Não sei dizer
Não sei dizer	Correto	Não sei dizer	Não	Não sei dizer
Não sei dizer	Não sei dizer	Não sei dizer	Não	Não sei dizer
Não sei dizer	Correto	Correto	Sim	Correto
Correto	Não sei dizer	Correto	Não	Não sei dizer
Correto	Correto	Correto	Sim	Correto
Não sei dizer	Não sei dizer	Não sei dizer	Não	Não sei dizer
Não sei dizer	Correto	Correto	Sim	Correto
Não sei dizer	Não sei dizer	Não sei dizer	Não	Não sei dizer
Não sei dizer	Correto	Correto	Sim	Correto
Correto	Correto	Correto	Sim	Correto
Não sei dizer	Não sei dizer	Correto	Não	Não sei dizer
Correto	Correto	Correto	Sim	Correto

Correto	Correto	Correto	Sim	Correto
Correto	Correto	Correto	Sim	Correto
Correto	Correto	Correto	Sim	Correto
Não sei dizer	Não sei dizer	Não sei dizer	Não	Não sei dizer
Correto	Correto	Correto	Sim	Correto
Correto	Correto	Correto	Sim	Correto
Errado	Correto	Errado	Sim	Correto
Correto	Correto	Correto	Sim	Correto
Correto	Não sei dizer	Não sei dizer	Sim	Correto
Não sei dizer	Correto	Não sei dizer	Sim	Correto
Correto	Correto	Correto	Sim	Correto
Não sei dizer	Não sei dizer	Não sei dizer	Não	Não sei dizer
Correto	Correto	Correto	Sim	Correto
Correto	Correto	Correto	Sim	Correto
Correto	Correto	Correto	Sim	Correto
Correto	Correto	Correto	Sim	Correto
Correto	Correto	Correto	Sim	Correto
Correto	Correto	Correto	Não	Correto
Não sei dizer	Não sei dizer	Não sei dizer	Não	Não sei dizer
Correto	Correto	Correto	Sim	Correto
Não sei dizer	Correto	Correto	Sim	Correto
Não sei dizer	Não sei dizer	Correto	Não	Não sei dizer
Correto	Correto	Errado	Sim	Correto
Correto	Não sei dizer	Não sei dizer	Não	Não sei dizer
Não sei dizer	Não sei dizer	Não sei dizer	Não	Não sei dizer
Correto	Correto	Correto	Sim	Correto
Correto	Correto	Correto	Sim	Correto
Não sei dizer	Não sei dizer	Correto	Não	Não sei dizer
Correto	Correto	Correto	Sim	Correto
Correto	Correto	Correto	Sim	Correto
Não sei dizer	Correto	Correto	Sim	Correto
Não sei dizer	Não sei dizer	Não sei dizer	Não	Não sei dizer
Correto	Correto	Correto	Sim	Correto
Correto	Correto	Correto	Sim	Correto
Correto	Correto	Errado	Sim	Correto
Correto	Correto	Correto	Sim	Correto
Não sei dizer	Não sei dizer	Não sei dizer	Não	Não sei dizer
Correto	Errado	Correto	Não	Errado
Correto	Não sei dizer	Correto	Sim	Correto
Correto	Correto	Correto	Sim	Correto
Correto	Correto	Correto	Sim	Correto
Errado	Correto	Errado	Sim	Correto
Correto	Correto	Correto	Sim	Correto
Não sei dizer	Não sei dizer	Não sei dizer	Não	Não sei dizer
Não sei dizer	Não sei dizer	Não sei dizer	Não	Não sei dizer
Não sei dizer	Não sei dizer	Não sei dizer	Não	Não sei dizer
Não sei dizer	Não sei dizer	Correto	Sim	Correto
Não sei dizer	Não sei dizer	Não sei dizer	Não	Não sei dizer
Não sei dizer	Não sei dizer	Não sei dizer	Não	Não sei dizer
Correto	Correto	Correto	Sim	Correto
Não sei dizer	Não sei dizer	Não sei dizer	Não	Não sei dizer
Não sei dizer	Não sei dizer	Não sei dizer	Não	Não sei dizer
Não sei dizer	Não sei dizer	Não sei dizer	Não	Não sei dizer
Não sei dizer	Não sei dizer	Não sei dizer	Não	Não sei dizer

Não sei dizer	Não sei dizer	Correto	Não	Não sei dizer
Não sei dizer	Não sei dizer	Não sei dizer	Não	Não sei dizer
Não sei dizer	Não sei dizer	Não sei dizer	Não	Não sei dizer
Não sei dizer	Não sei dizer	Correto	Sim	Correto
Não sei dizer	Correto	Não sei dizer	Não	Não sei dizer
Correto	Correto	Correto	Não	Correto
Não sei dizer	Não sei dizer	Correto	Não	Não sei dizer
Correto	Correto	Correto	Não	Não sei dizer
Correto	Correto	Correto	Sim	Correto
Não sei dizer	Não sei dizer	Não sei dizer	Não	Não sei dizer
Correto	Correto	Correto	Sim	Correto
Não sei dizer	Não sei dizer	Não sei dizer	Não	Não sei dizer
Correto	Não sei dizer	Não sei dizer	Não	Não sei dizer
Não sei dizer	Não sei dizer	Não sei dizer	Não	Não sei dizer
Correto	Correto	Correto	Sim	Correto
Não sei dizer	Não sei dizer	Não sei dizer	Não	Não sei dizer
Não sei dizer	Não sei dizer	Não sei dizer	Sim	Correto
Correto	Correto	Correto	Não	Correto

Q12	Q13	Q14	Q15	Q16
Não sei dizer	Não sei dizer	Não sei dizer	Não sei dizer	Não sei dizer
Correto	Não sei dizer	Não sei dizer	Não sei dizer	Não sei dizer
Não sei dizer	Não sei dizer	Não sei dizer	Não sei dizer	Não sei dizer
Correto	Não sei dizer	Não sei dizer	Correto	Não sei dizer
Correto	Correto	Correto	Correto	Não sei dizer
Não sei dizer	Não sei dizer	Não sei dizer	Correto	Não sei dizer
Não sei dizer	Não sei dizer	Correto	Correto	Errado
Correto	Não sei dizer	Correto	Correto	Não sei dizer
Correto	Não sei dizer	Não sei dizer	Não sei dizer	Não sei dizer
Correto	Correto	Correto	Correto	Não sei dizer
Correto	Não sei dizer	Correto	Correto	Correto
Não sei dizer	Não sei dizer	Correto	Correto	Correto
Correto	Correto	Correto	Correto	Não sei dizer
Correto	Correto	Correto	Correto	Não sei dizer
Correto	Correto	Correto	Correto	Correto
Não sei dizer	Não sei dizer	Não sei dizer	Não sei dizer	Não sei dizer
Correto	Correto	Correto	Correto	Correto
Não sei dizer	Não sei dizer	Não sei dizer	Não sei dizer	Não sei dizer
Não sei dizer	Não sei dizer	Correto	Correto	Correto
Correto	Correto	Correto	Correto	Correto
Correto	Não sei dizer	Não sei dizer	Não sei dizer	Não sei dizer
Correto	Não sei dizer	Correto	Correto	Não sei dizer
Correto	Não sei dizer	Correto	Correto	Não sei dizer
Correto	Não sei dizer	Correto	Correto	Correto
Correto	Não sei dizer	Correto	Correto	Correto
Correto	Não sei dizer	Correto	Correto	Não sei dizer
Correto	Correto	Correto	Correto	Não sei dizer
Não sei dizer	Não sei dizer	Não sei dizer	Correto	Não sei dizer
Correto	Correto	Correto	Correto	Não sei dizer
Correto	Correto	Correto	Correto	Correto
Correto	Não sei dizer	Correto	Não sei dizer	Não sei dizer
Correto	Correto	Não sei dizer	Correto	Não sei dizer
Correto	Correto	Não sei dizer	Correto	Correto
Correto	Correto	Não sei dizer	Correto	Não sei dizer
Correto	Correto	Correto	Correto	Correto
Correto	Não sei dizer	Correto	Correto	Não sei dizer
Correto	Não sei dizer	Correto	Correto	Correto
Correto	Correto	Correto	Correto	Correto
Correto	Não sei dizer	Não sei dizer	Correto	Não sei dizer
Correto	Não sei dizer	Correto	Correto	Não sei dizer
Correto	Correto	Correto	Correto	Correto
Correto	Correto	Correto	Correto	Não sei dizer
Correto	Não sei dizer	Não sei dizer	Correto	Não sei dizer
Correto	Correto	Correto	Correto	Correto
Correto	Não sei dizer	Correto	Não sei dizer	Não sei dizer
Correto	Correto	Correto	Correto	Errado
Correto	Correto	Correto	Correto	Não sei dizer
Correto	Não sei dizer	Correto	Correto	Não sei dizer
Correto	Correto	Correto	Correto	Correto
Não sei dizer	Não sei dizer	Não sei dizer	Não sei dizer	Não sei dizer
Não sei dizer	Não sei dizer	Não sei dizer	Não sei dizer	Não sei dizer
Correto	Correto	Não sei dizer	Correto	Não sei dizer
Não sei dizer	Não sei dizer	Não sei dizer	Não sei dizer	Não sei dizer

Correto	Não sei dizer	Correto	Correto	Correto
Correto	Não sei dizer	Correto	Não sei dizer	Não sei dizer
Não sei dizer	Não sei dizer	Não sei dizer	Não sei dizer	Não sei dizer
Não sei dizer	Não sei dizer	Não sei dizer	Correto	Não sei dizer
Correto	Não sei dizer	Correto	Correto	Não sei dizer
Correto	Não sei dizer	Correto	Correto	Correto
Não sei dizer	Não sei dizer	Não sei dizer	Não sei dizer	Não sei dizer
Não sei dizer	Não sei dizer	Não sei dizer	Não sei dizer	Não sei dizer
Correto	Correto	Correto	Correto	Correto
Correto	Correto	Correto	Correto	Correto
Correto	Não sei dizer	Correto	Correto	Não sei dizer
Correto	Correto	Não sei dizer	Não sei dizer	Correto
Não sei dizer	Não sei dizer	Não sei dizer	Não sei dizer	Não sei dizer
Correto	Não sei dizer	Não sei dizer	Correto	Correto
Correto	Correto	Não sei dizer	Correto	Não sei dizer
Correto	Correto	Correto	Correto	Correto
Correto	Correto	Não sei dizer	Correto	Correto
Não sei dizer	Não sei dizer	Não sei dizer	Correto	Não sei dizer
Não sei dizer	Não sei dizer	Não sei dizer	Não sei dizer	Não sei dizer
Não sei dizer	Não sei dizer	Não sei dizer	Correto	Não sei dizer
Correto	Correto	Correto	Correto	Correto
Correto	Correto	Correto	Correto	Correto
Não sei dizer	Não sei dizer	Não sei dizer	Não sei dizer	Não sei dizer
Correto	Errado	Não sei dizer	Correto	Não sei dizer
Correto	Não sei dizer	Não sei dizer	Correto	Não sei dizer
Correto	Correto	Não sei dizer	Correto	Correto
Correto	Não sei dizer	Correto	Correto	Correto
Não sei dizer	Não sei dizer	Não sei dizer	Não sei dizer	Não sei dizer
Correto	Não sei dizer	Correto	Não sei dizer	Não sei dizer
Correto	Correto	Correto	Correto	Correto
Correto	Não sei dizer	Não sei dizer	Correto	Não sei dizer
Correto	Não sei dizer	Não sei dizer	Correto	Não sei dizer
Não sei dizer	Não sei dizer	Não sei dizer	Não sei dizer	Não sei dizer
Não sei dizer	Não sei dizer	Não sei dizer	Não sei dizer	Não sei dizer
Não sei dizer	Não sei dizer	Não sei dizer	Não sei dizer	Não sei dizer
Correto	Correto	Correto	Correto	Correto
Correto	Correto	Não sei dizer	Correto	Não sei dizer
Correto	Não sei dizer	Correto	Correto	Não sei dizer
Correto	Correto	Correto	Correto	Não sei dizer
Não sei dizer	Não sei dizer	Correto	Correto	Não sei dizer
Correto	Não sei dizer	Correto	Correto	Não sei dizer
Correto	Não sei dizer	Correto	Correto	Não sei dizer
Correto	Não sei dizer	Não sei dizer	Correto	Não sei dizer
Não sei dizer	Não sei dizer	Não sei dizer	Não sei dizer	Não sei dizer
Correto	Não sei dizer	Não sei dizer	Não sei dizer	Não sei dizer
Correto	Não sei dizer	Correto	Correto	Não sei dizer
Correto	Correto	Correto	Correto	Correto
Não sei dizer	Não sei dizer	Não sei dizer	Errado	Não sei dizer
Correto	Não sei dizer	Correto	Correto	Correto
Não sei dizer	Não sei dizer	Não sei dizer	Não sei dizer	Não sei dizer
Correto	Não sei dizer	Correto	Não sei dizer	Não sei dizer
Não sei dizer	Não sei dizer	Não sei dizer	Correto	Não sei dizer
Correto	Não sei dizer	Correto	Correto	Não sei dizer
Não sei dizer	Não sei dizer	Não sei dizer	Não sei dizer	Não sei dizer

Correto	Correto	Correto	Correto	Correto
Não sei dizer	Não sei dizer	Não sei dizer	Não sei dizer	Não sei dizer
Não sei dizer	Não sei dizer	Não sei dizer	Não sei dizer	Não sei dizer
Não sei dizer	Não sei dizer	Correto	Correto	Não sei dizer
Não sei dizer	Não sei dizer	Correto	Correto	Não sei dizer
Correto	Não sei dizer	Errado	Correto	Correto
Correto	Correto	Correto	Correto	Correto
Não sei dizer	Não sei dizer	Não sei dizer	Não sei dizer	Não sei dizer
Correto	Não sei dizer	Não sei dizer	Não sei dizer	Não sei dizer
Não sei dizer	Não sei dizer	Não sei dizer	Não sei dizer	Não sei dizer
Correto	Não sei dizer	Correto	Não sei dizer	Não sei dizer
Correto	Não sei dizer	Correto	Correto	Correto
Não sei dizer	Não sei dizer	Não sei dizer	Correto	Não sei dizer
Correto	Correto	Correto	Correto	Correto
Não sei dizer	Não sei dizer	Não sei dizer	Correto	Não sei dizer
Não sei dizer	Não sei dizer	Correto	Não sei dizer	Não sei dizer
Correto	Correto	Correto	Correto	Correto
Não sei dizer	Não sei dizer	Não sei dizer	Não sei dizer	Não sei dizer
Correto	Correto	Correto	Correto	Correto
Correto	Correto	Correto	Correto	Correto
Correto	Não sei dizer	Não sei dizer	Correto	Não sei dizer
Correto	Correto	Correto	Correto	Não sei dizer
Correto	Não sei dizer	Correto	Correto	Correto
Correto	Não sei dizer	Não sei dizer	Correto	Não sei dizer
Não sei dizer	Não sei dizer	Não sei dizer	Não sei dizer	Não sei dizer
Não sei dizer	Não sei dizer	Não sei dizer	Não sei dizer	Não sei dizer
Não sei dizer	Não sei dizer	Não sei dizer	Não sei dizer	Não sei dizer
Não sei dizer	Não sei dizer	Não sei dizer	Não sei dizer	Não sei dizer
Correto	Correto	Correto	Correto	Correto
Correto	Correto	Correto	Correto	Não sei dizer
Correto	Não sei dizer	Correto	Correto	Não sei dizer
Não sei dizer	Não sei dizer	Não sei dizer	Não sei dizer	Não sei dizer
Não sei dizer	Correto	Correto	Correto	Não sei dizer
Correto	Não sei dizer	Correto	Correto	Não sei dizer
Correto	Correto	Correto	Correto	Não sei dizer
Correto	Correto	Não sei dizer	Correto	Não sei dizer
Não sei dizer	Não sei dizer	Não sei dizer	Não sei dizer	Não sei dizer
Correto	Não sei dizer	Correto	Não sei dizer	Não sei dizer
Correto	Não sei dizer	Correto	Correto	Não sei dizer
Correto	Não sei dizer	Não sei dizer	Correto	Não sei dizer
Correto	Correto	Correto	Correto	Não sei dizer
Correto	Correto	Correto	Correto	Correto
Correto	Não sei dizer	Correto	Correto	Não sei dizer
Não sei dizer	Não sei dizer	Não sei dizer	Não sei dizer	Não sei dizer
Correto	Não sei dizer	Correto	Correto	Não sei dizer
Correto	Não sei dizer	Não sei dizer	Não sei dizer	Não sei dizer
Correto	Não sei dizer	Correto	Correto	Não sei dizer
Correto	Correto	Correto	Correto	Errado
Não sei dizer	Não sei dizer	Não sei dizer	Não sei dizer	Não sei dizer
Não sei dizer	Não sei dizer	Não sei dizer	Não sei dizer	Não sei dizer
Correto	Não sei dizer	Correto	Correto	Correto
Correto	Não sei dizer	Não sei dizer	Correto	Não sei dizer
Não sei dizer	Não sei dizer	Não sei dizer	Não sei dizer	Não sei dizer
Correto	Correto	Correto	Correto	Correto

Não sei dizer	Não sei dizer	Não sei dizer	Não sei dizer	Não sei dizer
Correto	Correto	Errado	Correto	Errado
Não sei dizer	Não sei dizer	Correto	Correto	Não sei dizer
Correto	Correto	Correto	Correto	Correto
Não sei dizer	Não sei dizer	Não sei dizer	Correto	Não sei dizer
Correto	Não sei dizer	Correto	Correto	Não sei dizer
Correto	Não sei dizer	Correto	Correto	Não sei dizer
Não sei dizer	Não sei dizer	Não sei dizer	Não sei dizer	Não sei dizer
Correto	Correto	Correto	Correto	Correto
Não sei dizer	Não sei dizer	Não sei dizer	Não sei dizer	Não sei dizer
Não sei dizer	Não sei dizer	Não sei dizer	Correto	Não sei dizer
Não sei dizer	Não sei dizer	Correto	Correto	Correto
Não sei dizer	Não sei dizer	Não sei dizer	Correto	Não sei dizer
Correto	Não sei dizer	Correto	Correto	Não sei dizer
Correto	Correto	Correto	Correto	Não sei dizer
Correto	Não sei dizer	Correto	Correto	Não sei dizer
Correto	Não sei dizer	Correto	Correto	Não sei dizer
Não sei dizer	Não sei dizer	Não sei dizer	Não sei dizer	Não sei dizer
Correto	Não sei dizer	Não sei dizer	Correto	Não sei dizer
Correto	Não sei dizer	Não sei dizer	Correto	Não sei dizer
Correto	Não sei dizer	Não sei dizer	Correto	Não sei dizer
Correto	Não sei dizer	Correto	Correto	Correto
Não sei dizer	Não sei dizer	Não sei dizer	Não sei dizer	Não sei dizer
Correto	Não sei dizer	Correto	Correto	Não sei dizer
Correto	Correto	Não sei dizer	Correto	Correto
Correto	Não sei dizer	Correto	Correto	Correto
Não sei dizer	Não sei dizer	Não sei dizer	Não sei dizer	Não sei dizer
Correto	Não sei dizer	Correto	Não sei dizer	Não sei dizer
Correto	Correto	Correto	Correto	Correto
Correto	Correto	Correto	Correto	Não sei dizer
Não sei dizer	Não sei dizer	Não sei dizer	Não sei dizer	Não sei dizer
Correto	Correto	Não sei dizer	Correto	Não sei dizer
Correto	Não sei dizer	Correto	Correto	Não sei dizer
Correto	Correto	Correto	Correto	Correto
Correto	Não sei dizer	Correto	Correto	Não sei dizer
Correto	Correto	Correto	Correto	Não sei dizer
Errado	Correto	Errado	Correto	Correto
Correto	Correto	Correto	Correto	Correto
Não sei dizer	Não sei dizer	Não sei dizer	Não sei dizer	Não sei dizer
Não sei dizer	Não sei dizer	Não sei dizer	Não sei dizer	Não sei dizer
Correto	Não sei dizer	Não sei dizer	Correto	Não sei dizer
Não sei dizer	Não sei dizer	Não sei dizer	Não sei dizer	Não sei dizer
Não sei dizer	Não sei dizer	Não sei dizer	Correto	Não sei dizer
Correto	Não sei dizer	Correto	Correto	Não sei dizer
Correto	Errado	Correto	Correto	Não sei dizer
Correto	Correto	Correto	Correto	Correto
Correto	Não sei dizer	Correto	Correto	Não sei dizer
Não sei dizer	Não sei dizer	Não sei dizer	Não sei dizer	Não sei dizer
Não sei dizer	Não sei dizer	Correto	Não sei dizer	Não sei dizer
Correto	Correto	Correto	Correto	Correto
Não sei dizer	Não sei dizer	Não sei dizer	Não sei dizer	Não sei dizer
Correto	Não sei dizer	Correto	Correto	Não sei dizer
Não sei dizer	Não sei dizer	Não sei dizer	Não sei dizer	Não sei dizer
Não sei dizer	Não sei dizer	Não sei dizer	Não sei dizer	Não sei dizer

Não sei dizer	Não sei dizer	Não sei dizer	Não sei dizer	Não sei dizer
Correto	Não sei dizer	Correto	Correto	Correto
Correto	Correto	Correto	Correto	Correto
Não sei dizer	Não sei dizer	Não sei dizer	Não sei dizer	Não sei dizer
Não sei dizer	Não sei dizer	Correto	Correto	Não sei dizer
Correto	Correto	Correto	Correto	Correto
Não sei dizer	Não sei dizer	Não sei dizer	Não sei dizer	Não sei dizer
Não sei dizer	Não sei dizer	Correto	Não sei dizer	Não sei dizer
Correto	Correto	Correto	Correto	Correto
Correto	Não sei dizer	Correto	Correto	Não sei dizer
Correto	Não sei dizer	Correto	Correto	Correto
Correto	Não sei dizer	Correto	Correto	Correto
Errado	Correto	Correto	Errado	Não sei dizer
Não sei dizer	Não sei dizer	Correto	Correto	Não sei dizer
Correto	Não sei dizer	Correto	Correto	Correto
Não sei dizer	Não sei dizer	Correto	Não sei dizer	Não sei dizer
Correto	Correto	Correto	Correto	Não sei dizer
Correto	Não sei dizer	Correto	Correto	Correto
Correto	Errado	Correto	Correto	Correto
Correto	Não sei dizer	Correto	Correto	Não sei dizer
Correto	Não sei dizer	Não sei dizer	Correto	Correto
Correto	Errado	Correto	Correto	Correto
Correto	Correto	Correto	Correto	Correto
Correto	Correto	Não sei dizer	Correto	Correto
Não sei dizer	Não sei dizer	Não sei dizer	Não sei dizer	Não sei dizer
Correto	Não sei dizer	Não sei dizer	Correto	Não sei dizer
Não sei dizer	Não sei dizer	Não sei dizer	Não sei dizer	Não sei dizer
Não sei dizer	Não sei dizer	Não sei dizer	Não sei dizer	Não sei dizer
Não sei dizer	Não sei dizer	Não sei dizer	Não sei dizer	Não sei dizer
Correto	Correto	Correto	Correto	Correto
Correto	Não sei dizer	Correto	Correto	Correto
Não sei dizer	Não sei dizer	Não sei dizer	Não sei dizer	Não sei dizer
Não sei dizer	Não sei dizer	Correto	Não sei dizer	Não sei dizer
Correto	Não sei dizer	Correto	Correto	Correto
Correto	Correto	Correto	Correto	Correto
Correto	Correto	Correto	Correto	Não sei dizer
Correto	Correto	Correto	Correto	Correto
Não sei dizer	Não sei dizer	Correto	Correto	Não sei dizer
Correto	Correto	Correto	Correto	Correto
Correto	Não sei dizer	Não sei dizer	Não sei dizer	Não sei dizer
Correto	Correto	Correto	Correto	Correto
Correto	Não sei dizer	Correto	Correto	Correto
Não sei dizer	Não sei dizer	Não sei dizer	Não sei dizer	Não sei dizer
Correto	Não sei dizer	Correto	Correto	Não sei dizer
Correto	Correto	Correto	Correto	Correto
Correto	Não sei dizer	Não sei dizer	Correto	Não sei dizer
Correto	Correto	Correto	Correto	Correto
Não sei dizer	Não sei dizer	Não sei dizer	Não sei dizer	Não sei dizer
Correto	Correto	Correto	Correto	Correto
Correto	Correto	Correto	Correto	Correto
Correto	Correto	Correto	Correto	Correto
Não sei dizer	Não sei dizer	Não sei dizer	Não sei dizer	Não sei dizer
Correto	Correto	Correto	Correto	Correto
Correto	Não sei dizer	Correto	Correto	Não sei dizer

Não sei dizer	Não sei dizer	Não sei dizer	Não sei dizer	Não sei dizer
Correto	Correto	Correto	Correto	Correto
Correto	Correto	Correto	Correto	Correto
Correto	Correto	Não sei dizer	Correto	Não sei dizer
Correto	Não sei dizer	Correto	Correto	Correto
Correto	Não sei dizer	Errado	Correto	Não sei dizer
Correto	Não sei dizer	Correto	Correto	Não sei dizer
Correto	Não sei dizer	Correto	Correto	Não sei dizer
Correto	Correto	Correto	Correto	Correto
Não sei dizer	Não sei dizer	Não sei dizer	Não sei dizer	Não sei dizer
Correto	Correto	Correto	Não sei dizer	Correto
Correto	Correto	Correto	Correto	Correto
Correto	Não sei dizer	Correto	Correto	Correto
Correto	Correto	Correto	Correto	Correto
Correto	Não sei dizer	Correto	Correto	Não sei dizer
Correto	Correto	Correto	Correto	Não sei dizer
Não sei dizer	Não sei dizer	Correto	Correto	Não sei dizer
Correto	Correto	Correto	Correto	Correto
Correto	Correto	Não sei dizer	Correto	Correto
Correto	Não sei dizer	Correto	Correto	Não sei dizer
Não sei dizer	Não sei dizer	Não sei dizer	Não sei dizer	Não sei dizer
Correto	Correto	Correto	Correto	Correto
Correto	Correto	Correto	Correto	Correto
Correto	Correto	Correto	Correto	Correto
Correto	Não sei dizer	Não sei dizer	Não sei dizer	Não sei dizer
Correto	Correto	Correto	Correto	Correto
Correto	Não sei dizer	Correto	Correto	Não sei dizer
Não sei dizer	Não sei dizer	Não sei dizer	Não sei dizer	Não sei dizer
Correto	Não sei dizer	Correto	Correto	Não sei dizer
Correto	Correto	Não sei dizer	Correto	Não sei dizer
Correto	Correto	Correto	Correto	Correto
Não sei dizer	Não sei dizer	Não sei dizer	Não sei dizer	Não sei dizer
Correto	Correto	Correto	Correto	Não sei dizer
Correto	Correto	Correto	Correto	Correto
Não sei dizer	Não sei dizer	Não sei dizer	Correto	Não sei dizer
Não sei dizer	Correto	Correto	Correto	Correto
Correto	Correto	Correto	Correto	Correto
Não sei dizer	Não sei dizer	Correto	Correto	Correto
Correto	Não sei dizer	Não sei dizer	Correto	Correto
Correto	Não sei dizer	Correto	Correto	Correto
Errado	Não sei dizer	Correto	Correto	Correto
Correto	Correto	Correto	Correto	Correto
Correto	Correto	Correto	Correto	Correto
Não sei dizer	Não sei dizer	Correto	Correto	Não sei dizer
Correto	Não sei dizer	Correto	Correto	Não sei dizer
Correto	Não sei dizer	Não sei dizer	Correto	Não sei dizer
Correto	Errado	Correto	Correto	Correto
Correto	Correto	Correto	Correto	Correto
Correto	Não sei dizer	Correto	Não sei dizer	Não sei dizer
Correto	Não sei dizer	Não sei dizer	Correto	Correto
Correto	Não sei dizer	Não sei dizer	Correto	Não sei dizer
Correto	Não sei dizer	Não sei dizer	Correto	Não sei dizer
Não sei dizer	Não sei dizer	Não sei dizer	Não sei dizer	Não sei dizer
Correto	Não sei dizer	Correto	Correto	Não sei dizer

Não sei dizer	Não sei dizer	Correto	Correto	Não sei dizer
Não sei dizer	Não sei dizer	Não sei dizer	Não sei dizer	Não sei dizer
Correto	Não sei dizer	Correto	Correto	Não sei dizer
Correto	Não sei dizer	Correto	Correto	Não sei dizer
Correto	Não sei dizer	Correto	Correto	Correto
Correto	Não sei dizer	Não sei dizer	Correto	Não sei dizer
Correto	Não sei dizer	Correto	Correto	Não sei dizer
Não sei dizer	Não sei dizer	Correto	Correto	Não sei dizer
Correto	Não sei dizer	Não sei dizer	Correto	Não sei dizer
Correto	Não sei dizer	Não sei dizer	Correto	Não sei dizer
Correto	Não sei dizer	Correto	Correto	Não sei dizer
Não sei dizer	Não sei dizer	Não sei dizer	Não sei dizer	Não sei dizer
Não sei dizer	Não sei dizer	Não sei dizer	Não sei dizer	Não sei dizer
Correto	Não sei dizer	Correto	Correto	Não sei dizer
Não sei dizer	Não sei dizer	Não sei dizer	Não sei dizer	Não sei dizer
Não sei dizer	Não sei dizer	Não sei dizer	Não sei dizer	Não sei dizer
Correto	Correto	Correto	Não sei dizer	Não sei dizer
Correto	Correto	Correto	Correto	Correto
Correto	Não sei dizer	Não sei dizer	Não sei dizer	Não sei dizer
Correto	Não sei dizer	Não sei dizer	Não sei dizer	Não sei dizer
Não sei dizer	Não sei dizer	Não sei dizer	Não sei dizer	Não sei dizer
Correto	Não sei dizer	Correto	Correto	Não sei dizer
Correto	Correto	Correto	Correto	Não sei dizer
Não sei dizer	Não sei dizer	Não sei dizer	Não sei dizer	Não sei dizer
Não sei dizer	Não sei dizer	Correto	Correto	Não sei dizer
Não sei dizer	Não sei dizer	Não sei dizer	Não sei dizer	Não sei dizer
Correto	Não sei dizer	Correto	Correto	Não sei dizer
Correto	Não sei dizer	Correto	Não sei dizer	Não sei dizer
Não sei dizer	Não sei dizer	Não sei dizer	Não sei dizer	Não sei dizer
Correto	Não sei dizer	Correto	Correto	Não sei dizer
Correto	Correto	Correto	Correto	Correto
Correto	Não sei dizer	Não sei dizer	Não sei dizer	Não sei dizer
Correto	Correto	Errado	Correto	Correto
Correto	Não sei dizer	Não sei dizer	Correto	Não sei dizer
Correto	Não sei dizer	Correto	Correto	Não sei dizer
Não sei dizer	Não sei dizer	Não sei dizer	Não sei dizer	Não sei dizer
Correto	Correto	Correto	Correto	Correto
Correto	Não sei dizer	Correto	Não sei dizer	Não sei dizer
Correto	Correto	Não sei dizer	Não sei dizer	Correto
Correto	Correto	Correto	Correto	Correto
Correto	Não sei dizer	Correto	Correto	Correto
Correto	Correto	Correto	Correto	Não sei dizer
Não sei dizer	Não sei dizer	Correto	Correto	Não sei dizer
Correto	Correto	Correto	Correto	Não sei dizer
Correto	Não sei dizer	Não sei dizer	Correto	Correto
Correto	Correto	Correto	Correto	Não sei dizer
Correto	Não sei dizer	Correto	Correto	Errado
Correto	Correto	Correto	Correto	Correto
Não sei dizer	Não sei dizer	Não sei dizer	Não sei dizer	Não sei dizer
Não sei dizer	Não sei dizer	Não sei dizer	Não sei dizer	Não sei dizer
Correto	Não sei dizer	Não sei dizer	Correto	Não sei dizer
Correto	Não sei dizer	Não sei dizer	Correto	Não sei dizer
Correto	Correto	Correto	Correto	Não sei dizer
Correto	Não sei dizer	Correto	Correto	Correto

Correto	Correto	Não sei dizer	Correto	Não sei dizer
Correto	Correto	Errado	Correto	Correto
Não sei dizer	Não sei dizer	Não sei dizer	Não sei dizer	Não sei dizer
Correto	Correto	Correto	Correto	Correto
Não sei dizer	Não sei dizer	Correto	Correto	Não sei dizer
Correto	Correto	Correto	Correto	Correto
Não sei dizer	Não sei dizer	Não sei dizer	Não sei dizer	Não sei dizer
Correto	Correto	Não sei dizer	Correto	Não sei dizer
Não sei dizer	Não sei dizer	Não sei dizer	Não sei dizer	Não sei dizer
Não sei dizer	Não sei dizer	Não sei dizer	Não sei dizer	Não sei dizer
Correto	Correto	Correto	Correto	Correto
Correto	Correto	Correto	Correto	Correto
Correto	Não sei dizer	Correto	Correto	Não sei dizer
Correto	Não sei dizer	Correto	Não sei dizer	Não sei dizer
Correto	Correto	Correto	Correto	Correto
Correto	Correto	Não sei dizer	Não sei dizer	Correto
Correto	Correto	Não sei dizer	Não sei dizer	Não sei dizer
Correto	Não sei dizer	Correto	Correto	Não sei dizer
Correto	Não sei dizer	Correto	Correto	Não sei dizer
Correto	Não sei dizer	Não sei dizer	Não sei dizer	Não sei dizer
Correto	Não sei dizer	Não sei dizer	Correto	Não sei dizer
Correto	Correto	Correto	Correto	Correto
Correto	Não sei dizer	Não sei dizer	Correto	Não sei dizer
Correto	Não sei dizer	Não sei dizer	Não sei dizer	Não sei dizer
Não sei dizer	Não sei dizer	Não sei dizer	Não sei dizer	Não sei dizer
Não sei dizer	Não sei dizer	Não sei dizer	Correto	Não sei dizer
Correto	Não sei dizer	Correto	Correto	Correto
Não sei dizer	Não sei dizer	Não sei dizer	Correto	Não sei dizer
Correto	Correto	Correto	Correto	Não sei dizer
Não sei dizer	Não sei dizer	Não sei dizer	Não sei dizer	Não sei dizer
Não sei dizer	Não sei dizer	Não sei dizer	Não sei dizer	Não sei dizer
Correto	Não sei dizer	Correto	Correto	Não sei dizer
Não sei dizer	Não sei dizer	Não sei dizer	Não sei dizer	Não sei dizer
Não sei dizer	Não sei dizer	Não sei dizer	Não sei dizer	Não sei dizer
Não sei dizer	Não sei dizer	Não sei dizer	Não sei dizer	Não sei dizer
Correto	Correto	Não sei dizer	Errado	Correto
Correto	Correto	Correto	Correto	Correto
Não sei dizer	Não sei dizer	Não sei dizer	Não sei dizer	Não sei dizer
Não sei dizer	Não sei dizer	Não sei dizer	Não sei dizer	Não sei dizer
Correto	Não sei dizer	Correto	Correto	Correto
Correto	Correto	Correto	Correto	Correto
Não sei dizer	Não sei dizer	Não sei dizer	Não sei dizer	Não sei dizer
Não sei dizer	Não sei dizer	Não sei dizer	Não sei dizer	Não sei dizer
Não sei dizer	Não sei dizer	Não sei dizer	Não sei dizer	Não sei dizer
Correto	Não sei dizer	Correto	Não sei dizer	Não sei dizer
Correto	Não sei dizer	Não sei dizer	Correto	Não sei dizer
Correto	Não sei dizer	Não sei dizer	Correto	Correto
Não sei dizer	Não sei dizer	Não sei dizer	Não sei dizer	Não sei dizer
Correto	Correto	Correto	Correto	Correto
Não sei dizer	Não sei dizer	Não sei dizer	Não sei dizer	Não sei dizer
Correto	Não sei dizer	Correto	Não sei dizer	Não sei dizer
Correto	Correto	Correto	Correto	Correto
Não sei dizer	Não sei dizer	Não sei dizer	Não sei dizer	Não sei dizer
Correto	Não sei dizer	Não sei dizer	Não sei dizer	Não sei dizer

Correto	Não sei dizer	Não sei dizer	Correto	Correto
Correto	Correto	Não sei dizer	Correto	Correto
Correto	Não sei dizer	Correto	Correto	Não sei dizer
Não sei dizer	Não sei dizer	Não sei dizer	Não sei dizer	Não sei dizer
Correto	Correto	Correto	Correto	Correto
Correto	Correto	Correto	Correto	Correto
Correto	Não sei dizer	Correto	Correto	Não sei dizer
Correto	Correto	Correto	Correto	Não sei dizer
Correto	Não sei dizer	Correto	Correto	Correto
Correto	Correto	Correto	Correto	Correto
Correto	Correto	Correto	Correto	Correto
Não sei dizer	Não sei dizer	Não sei dizer	Não sei dizer	Não sei dizer
Correto	Correto	Correto	Correto	Errado
Correto	Correto	Correto	Correto	Correto
Correto	Correto	Correto	Correto	Correto
Correto	Correto	Correto	Correto	Correto
Correto	Correto	Correto	Correto	Correto
Correto	Correto	Não sei dizer	Correto	Correto
Não sei dizer	Não sei dizer	Não sei dizer	Não sei dizer	Não sei dizer
Correto	Correto	Correto	Correto	Correto
Correto	Não sei dizer	Correto	Correto	Não sei dizer
Não sei dizer	Não sei dizer	Não sei dizer	Não sei dizer	Não sei dizer
Correto	Correto	Correto	Correto	Correto
Não sei dizer	Não sei dizer	Não sei dizer	Não sei dizer	Não sei dizer
Não sei dizer	Não sei dizer	Não sei dizer	Não sei dizer	Não sei dizer
Correto	Correto	Correto	Correto	Errado
Correto	Errado	Correto	Correto	Errado
Não sei dizer	Não sei dizer	Não sei dizer	Não sei dizer	Não sei dizer
Correto	Correto	Correto	Correto	Correto
Correto	Correto	Correto	Correto	Correto
Correto	Não sei dizer	Correto	Correto	Correto
Não sei dizer	Não sei dizer	Não sei dizer	Não sei dizer	Não sei dizer
Correto	Não sei dizer	Correto	Correto	Correto
Correto	Correto	Correto	Correto	Correto
Correto	Correto	Não sei dizer	Correto	Correto
Correto	Correto	Correto	Correto	Correto
Não sei dizer	Não sei dizer	Não sei dizer	Não sei dizer	Não sei dizer
Correto	Não sei dizer	Errado	Correto	Não sei dizer
Correto	Correto	Não sei dizer	Correto	Correto
Correto	Correto	Correto	Correto	Correto
Correto	Correto	Correto	Correto	Correto
Correto	Correto	Não sei dizer	Correto	Não sei dizer
Correto	Correto	Correto	Correto	Errado
Não sei dizer	Não sei dizer	Não sei dizer	Não sei dizer	Não sei dizer
Não sei dizer	Não sei dizer	Não sei dizer	Não sei dizer	Não sei dizer
Não sei dizer	Não sei dizer	Não sei dizer	Não sei dizer	Não sei dizer
Correto	Não sei dizer	Não sei dizer	Correto	Não sei dizer
Não sei dizer	Não sei dizer	Correto	Correto	Correto
Não sei dizer	Não sei dizer	Não sei dizer	Não sei dizer	Não sei dizer
Correto	Correto	Correto	Correto	Correto
Não sei dizer	Não sei dizer	Não sei dizer	Não sei dizer	Não sei dizer
Não sei dizer	Não sei dizer	Não sei dizer	Não sei dizer	Não sei dizer
Não sei dizer	Não sei dizer	Não sei dizer	Não sei dizer	Não sei dizer
Não sei dizer	Não sei dizer	Não sei dizer	Não sei dizer	Não sei dizer

Não sei dizer	Não sei dizer	Não sei dizer	Correto	Não sei dizer
Não sei dizer	Não sei dizer	Não sei dizer	Não sei dizer	Não sei dizer
Não sei dizer	Não sei dizer	Não sei dizer	Não sei dizer	Não sei dizer
Correto	Não sei dizer	Não sei dizer	Correto	Correto
Não sei dizer	Não sei dizer	Não sei dizer	Não sei dizer	Não sei dizer
Correto	Não sei dizer	Não sei dizer	Correto	Não sei dizer
Não sei dizer	Não sei dizer	Não sei dizer	Não sei dizer	Não sei dizer
Correto	Não sei dizer	Não sei dizer	Correto	Não sei dizer
Correto	Não sei dizer	Não sei dizer	Correto	Correto
Não sei dizer	Não sei dizer	Não sei dizer	Não sei dizer	Não sei dizer
Correto	Correto	Correto	Correto	Correto
Não sei dizer	Não sei dizer	Não sei dizer	Não sei dizer	Não sei dizer
Não sei dizer	Não sei dizer	Não sei dizer	Não sei dizer	Não sei dizer
Não sei dizer	Não sei dizer	Não sei dizer	Não sei dizer	Não sei dizer
Correto	Correto	Correto	Correto	Correto
Não sei dizer	Não sei dizer	Não sei dizer	Não sei dizer	Não sei dizer
Não sei dizer	Não sei dizer	Não sei dizer	Não sei dizer	Não sei dizer
Correto	Não sei dizer	Correto	Correto	Não sei dizer

Q1	Q2	Q3	Q4	Q5
Errado	Errado	Correto	Correto	Não sei dizer
Correto	Correto	Correto	Errado	Não sei dizer
Correto	Correto	Correto	Não sei dizer	Correto
Correto	Correto	Correto	Não sei dizer	Não sei dizer
Correto	Errado	Correto	Correto	Não sei dizer
Correto	Não sei dizer	Correto	Não sei dizer	Não sei dizer
Não sei dizer	Correto	Não sei dizer	Não sei dizer	Não sei dizer
Correto	Correto	Correto	Correto	Correto
Correto	Correto	Correto	Correto	Não sei dizer
Correto	Correto	Correto	Errado	Correto
Correto	Errado	Correto	Correto	Correto
Correto	Errado	Correto	Correto	Não sei dizer
Correto	Correto	Correto	Correto	Errado
Correto	Correto	Correto	Correto	Correto
Correto	Correto	Correto	Não sei dizer	Correto
Correto	Errado	Correto	Errado	Não sei dizer
Correto	Correto	Correto	Correto	Correto
Correto	Correto	Correto	Errado	Não sei dizer
Correto	Correto	Correto	Correto	Não sei dizer
Correto	Correto	Correto	Correto	Correto
Correto	Correto	Correto	Errado	Não sei dizer
Correto	Correto	Correto	Correto	Correto
Correto	Correto	Correto	Errado	Correto
Correto	Não sei dizer	Correto	Não sei dizer	Correto
Correto	Correto	Correto	Correto	Correto
Correto	Correto	Correto	Correto	Correto
Correto	Correto	Correto	Correto	Correto
Correto	Errado	Correto	Não sei dizer	Correto
Correto	Correto	Correto	Correto	Errado
Correto	Correto	Correto	Errado	Correto
Correto	Não sei dizer	Correto	Correto	Não sei dizer
Errado	Errado	Correto	Correto	Não sei dizer
Correto	Correto	Correto	Correto	Correto
Correto	Correto	Correto	Errado	Não sei dizer
Correto	Errado	Correto	Correto	Correto
Correto	Correto	Correto	Correto	Não sei dizer
Correto	Correto	Correto	Errado	Errado
Correto	Correto	Correto	Correto	Correto
Correto	Correto	Correto	Correto	Não sei dizer
Correto	Correto	Correto	Não sei dizer	Correto
Correto	Correto	Correto	Correto	Correto
Errado	Errado	Correto	Não sei dizer	Correto
Correto	Correto	Correto	Correto	Não sei dizer
Correto	Correto	Correto	Correto	Correto
Correto	Correto	Correto	Não sei dizer	Correto
Correto	Correto	Correto	Correto	Correto
Correto	Correto	Correto	Errado	Correto
Correto	Correto	Correto	Correto	Correto
Errado	Correto	Correto	Correto	Correto
Correto	Correto	Correto	Não sei dizer	Não sei dizer
Correto	Correto	Correto	Não sei dizer	Não sei dizer
Correto	Correto	Correto	Não sei dizer	Errado
Correto	Correto	Correto	Correto	Não sei dizer

Correto	Correto	Correto	Errado	Correto
Não sei dizer	Não sei dizer	Correto	Correto	Correto
Correto	Não sei dizer	Correto	Errado	Não sei dizer
Correto	Correto	Correto	Correto	Correto
Correto	Correto	Correto	Correto	Correto
Correto	Correto	Correto	Correto	Correto
Correto	Errado	Correto	Não sei dizer	Não sei dizer
Correto	Correto	Correto	Não sei dizer	Não sei dizer
Correto	Correto	Correto	Errado	Correto
Correto	Correto	Correto	Correto	Correto
Correto	Correto	Correto	Correto	Não sei dizer
Correto	Correto	Correto	Correto	Correto
Correto	Não sei dizer	Correto	Não sei dizer	Não sei dizer
Correto	Correto	Correto	Não sei dizer	Não sei dizer
Errado	Não sei dizer	Correto	Correto	Não sei dizer
Correto	Errado	Correto	Correto	Correto
Correto	Errado	Correto	Correto	Não sei dizer
Correto	Correto	Correto	Não sei dizer	Não sei dizer
Correto	Correto	Correto	Não sei dizer	Correto
Correto	Errado	Correto	Errado	Não sei dizer
Correto	Não sei dizer	Correto	Não sei dizer	Correto
Correto	Correto	Correto	Correto	Correto
Correto	Correto	Correto	Não sei dizer	Não sei dizer
Correto	Correto	Correto	Correto	Errado
Correto	Correto	Correto	Errado	Não sei dizer
Correto	Correto	Correto	Errado	Correto
Correto	Correto	Correto	Errado	Correto
Correto	Errado	Correto	Errado	Não sei dizer
Correto	Errado	Correto	Não sei dizer	Não sei dizer
Correto	Correto	Correto	Correto	Correto
Correto	Correto	Correto	Correto	Correto
Correto	Não sei dizer	Correto	Não sei dizer	Não sei dizer
Correto	Errado	Correto	Errado	Não sei dizer
Correto	Correto	Correto	Não sei dizer	Não sei dizer
Correto	Correto	Correto	Errado	Não sei dizer
Correto	Errado	Correto	Correto	Correto
Correto	Correto	Correto	Correto	Correto
Correto	Correto	Correto	Correto	Não sei dizer
Correto	Correto	Correto	Correto	Correto
Correto	Correto	Correto	Correto	Não sei dizer
Correto	Errado	Correto	Não sei dizer	Não sei dizer
Correto	Errado	Correto	Errado	Correto
Correto	Correto	Errado	Correto	Não sei dizer
Correto	Errado	Correto	Não sei dizer	Não sei dizer
Correto	Correto	Correto	Correto	Correto
Correto	Errado	Correto	Não sei dizer	Não sei dizer
Correto	Errado	Correto	Errado	Não sei dizer
Não sei dizer	Não sei dizer	Correto	Não sei dizer	Correto
Correto	Não sei dizer	Correto	Correto	Correto
Correto	Correto	Correto	Não sei dizer	Não sei dizer
Correto	Errado	Correto	Não sei dizer	Correto
Errado	Correto	Correto	Correto	Correto
Correto	Correto	Correto	Correto	Não sei dizer
Não sei dizer	Correto	Correto	Não sei dizer	Não sei dizer

Correto	Errado	Correto	Errado	Correto
Correto	Não sei dizer	Correto	Não sei dizer	Não sei dizer
Correto	Correto	Correto	Errado	Não sei dizer
Não sei dizer	Não sei dizer	Correto	Não sei dizer	Não sei dizer
Correto	Correto	Correto	Não sei dizer	Não sei dizer
Correto	Correto	Correto	Correto	Correto
Errado	Correto	Correto	Correto	Correto
Correto	Errado	Correto	Correto	Não sei dizer
Correto	Correto	Correto	Não sei dizer	Não sei dizer
Correto	Correto	Correto	Correto	Não sei dizer
Correto	Errado	Correto	Correto	Correto
Correto	Correto	Correto	Correto	Correto
Correto	Correto	Correto	Errado	Não sei dizer
Correto	Errado	Correto	Correto	Correto
Correto	Correto	Correto	Não sei dizer	Não sei dizer
Correto	Correto	Correto	Correto	Não sei dizer
Correto	Correto	Correto	Correto	Errado
Correto	Errado	Correto	Não sei dizer	Não sei dizer
Correto	Correto	Correto	Errado	Não sei dizer
Correto	Correto	Correto	Correto	Correto
Correto	Correto	Correto	Correto	Não sei dizer
Correto	Não sei dizer	Correto	Correto	Não sei dizer
Correto	Correto	Correto	Errado	Correto
Correto	Errado	Correto	Correto	Correto
Correto	Não sei dizer	Não sei dizer	Não sei dizer	Não sei dizer
Correto	Correto	Correto	Não sei dizer	Não sei dizer
Não sei dizer	Correto	Correto	Correto	Não sei dizer
Correto	Correto	Correto	Não sei dizer	Não sei dizer
Correto	Correto	Correto	Correto	Correto
Correto	Correto	Correto	Correto	Correto
Correto	Correto	Correto	Correto	Correto
Errado	Errado	Correto	Correto	Não sei dizer
Correto	Correto	Correto	Errado	Correto
Correto	Não sei dizer	Correto	Correto	Correto
Correto	Correto	Correto	Correto	Correto
Correto	Errado	Correto	Correto	Não sei dizer
Errado	Não sei dizer	Correto	Não sei dizer	Não sei dizer
Correto	Correto	Correto	Não sei dizer	Não sei dizer
Correto	Correto	Correto	Errado	Correto
Correto	Correto	Correto	Correto	Não sei dizer
Correto	Não sei dizer	Correto	Correto	Não sei dizer
Correto	Errado	Correto	Correto	Não sei dizer
Correto	Correto	Correto	Não sei dizer	Não sei dizer
Correto	Errado	Correto	Não sei dizer	Não sei dizer
Correto	Correto	Correto	Correto	Correto
Correto	Correto	Correto	Errado	Errado
Correto	Correto	Correto	Correto	Correto
Correto	Correto	Correto	Correto	Correto
Correto	Correto	Correto	Não sei dizer	Não sei dizer
Errado	Correto	Correto	Não sei dizer	Não sei dizer
Correto	Correto	Correto	Não sei dizer	Correto
Correto	Correto	Correto	Correto	Errado
Não sei dizer	Não sei dizer	Correto	Não sei dizer	Não sei dizer
Correto	Correto	Correto	Correto	Correto

Correto	Não sei dizer	Correto	Errado	Não sei dizer
Correto	Correto	Correto	Não sei dizer	Correto
Correto	Não sei dizer	Correto	Não sei dizer	Não sei dizer
Correto	Não sei dizer	Correto	Correto	Correto
Correto	Correto	Correto	Não sei dizer	Não sei dizer
Correto	Não sei dizer	Correto	Não sei dizer	Não sei dizer
Correto	Correto	Correto	Correto	Correto
Não sei dizer	Não sei dizer	Correto	Correto	Não sei dizer
Correto	Não sei dizer	Correto	Correto	Correto
Correto	Correto	Correto	Correto	Não sei dizer
Correto	Correto	Correto	Correto	Não sei dizer
Correto	Correto	Correto	Não sei dizer	Não sei dizer
Correto	Correto	Correto	Correto	Errado
Correto	Correto	Correto	Correto	Correto
Correto	Correto	Correto	Correto	Não sei dizer
Correto	Correto	Correto	Errado	Não sei dizer
Correto	Correto	Correto	Errado	Não sei dizer
Correto	Correto	Correto	Correto	Não sei dizer
Correto	Não sei dizer	Correto	Não sei dizer	Correto
Correto	Correto	Correto	Correto	Correto
Correto	Correto	Correto	Correto	Não sei dizer
Correto	Correto	Correto	Correto	Não sei dizer
Correto	Errado	Correto	Não sei dizer	Não sei dizer
Correto	Errado	Correto	Não sei dizer	Correto
Correto	Correto	Correto	Correto	Correto
Correto	Correto	Correto	Correto	Correto
Correto	Correto	Correto	Correto	Não sei dizer
Correto	Correto	Correto	Não sei dizer	Correto
Correto	Correto	Correto	Correto	Correto
Não sei dizer	Não sei dizer	Correto	Não sei dizer	Correto
Correto	Correto	Correto	Não sei dizer	Não sei dizer
Correto	Correto	Correto	Correto	Correto
Correto	Correto	Correto	Não sei dizer	Correto
Correto	Errado	Correto	Correto	Correto
Correto	Correto	Correto	Não sei dizer	Não sei dizer
Correto	Correto	Correto	Não sei dizer	Não sei dizer
Correto	Correto	Correto	Correto	Correto
Correto	Errado	Correto	Correto	Correto
Correto	Correto	Correto	Não sei dizer	Não sei dizer
Correto	Correto	Correto	Não sei dizer	Não sei dizer
Correto	Errado	Correto	Não sei dizer	Não sei dizer
Correto	Correto	Correto	Não sei dizer	Não sei dizer
Correto	Não sei dizer	Correto	Não sei dizer	Não sei dizer
Correto	Não sei dizer	Correto	Correto	Correto
Correto	Correto	Correto	Correto	Correto
Correto	Correto	Correto	Errado	Errado
Correto	Correto	Correto	Correto	Correto
Correto	Correto	Correto	Correto	Não sei dizer
Correto	Correto	Correto	Não sei dizer	Não sei dizer
Correto	Errado	Correto	Correto	Correto
Correto	Não sei dizer	Correto	Não sei dizer	Não sei dizer
Correto	Correto	Correto	Errado	Não sei dizer
Correto	Correto	Correto	Não sei dizer	Não sei dizer
Correto	Não sei dizer	Correto	Não sei dizer	Não sei dizer

Correto	Correto	Correto	Correto	Não sei dizer
Errado	Correto	Correto	Correto	Correto
Errado	Errado	Correto	Correto	Correto
Correto	Correto	Correto	Correto	Não sei dizer
Correto	Correto	Correto	Correto	Não sei dizer
Correto	Correto	Correto	Correto	Correto
Correto	Errado	Correto	Correto	Não sei dizer
Não sei dizer	Não sei dizer	Não sei dizer	Correto	Não sei dizer
Correto	Correto	Correto	Correto	Correto
Correto	Errado	Correto	Errado	Correto
Correto	Correto	Correto	Errado	Não sei dizer
Correto	Correto	Correto	Não sei dizer	Correto
Correto	Errado	Correto	Errado	Não sei dizer
Errado	Errado	Correto	Correto	Não sei dizer
Errado	Errado	Correto	Errado	Não sei dizer
Correto	Errado	Correto	Não sei dizer	Não sei dizer
Correto	Correto	Correto	Correto	Correto
Correto	Correto	Correto	Correto	Correto
Correto	Correto	Correto	Errado	Correto
Correto	Errado	Correto	Correto	Não sei dizer
Correto	Correto	Correto	Correto	Correto
Correto	Errado	Correto	Correto	Correto
Correto	Correto	Correto	Correto	Correto
Correto	Correto	Correto	Correto	Correto
Correto	Correto	Correto	Correto	Não sei dizer
Correto	Correto	Correto	Correto	Correto
Correto	Correto	Correto	Correto	Não sei dizer
Correto	Não sei dizer	Correto	Não sei dizer	Não sei dizer
Correto	Correto	Correto	Não sei dizer	Correto
Correto	Correto	Correto	Correto	Correto
Correto	Correto	Correto	Não sei dizer	Correto
Errado	Correto	Correto	Errado	Não sei dizer
Correto	Errado	Correto	Não sei dizer	Não sei dizer
Correto	Correto	Correto	Correto	Não sei dizer
Correto	Correto	Correto	Correto	Correto
Correto	Correto	Correto	Correto	Correto
Correto	Correto	Correto	Correto	Correto
Correto	Errado	Correto	Correto	Não sei dizer
Correto	Correto	Correto	Correto	Correto
Correto	Correto	Correto	Não sei dizer	Não sei dizer
Correto	Correto	Correto	Correto	Correto
Correto	Correto	Correto	Correto	Correto
Correto	Correto	Correto	Errado	Não sei dizer
Correto	Errado	Correto	Correto	Correto
Correto	Correto	Correto	Errado	Correto
Correto	Correto	Correto	Errado	Não sei dizer
Errado	Errado	Correto	Não sei dizer	Correto
Correto	Não sei dizer	Correto	Correto	Não sei dizer
Correto	Errado	Correto	Correto	Correto
Correto	Correto	Correto	Correto	Correto
Correto	Correto	Correto	Correto	Correto
Correto	Errado	Correto	Correto	Não sei dizer
Correto	Correto	Correto	Errado	Correto
Correto	Correto	Correto	Correto	Correto

Errado	Errado	Correto	Não sei dizer	Não sei dizer
Correto	Correto	Correto	Correto	Correto
Correto	Correto	Correto	Correto	Correto
Correto	Errado	Correto	Correto	Correto
Correto	Correto	Correto	Correto	Correto
Correto	Errado	Correto	Correto	Não sei dizer
Correto	Correto	Correto	Correto	Correto
Correto	Errado	Correto	Correto	Correto
Correto	Errado	Correto	Correto	Correto
Não sei dizer	Errado	Errado	Errado	Errado
Correto	Correto	Correto	Correto	Correto
Correto	Errado	Correto	Correto	Correto
Correto	Errado	Correto	Errado	Correto
Correto	Correto	Correto	Correto	Correto
Correto	Correto	Correto	Correto	Correto
Correto	Correto	Correto	Correto	Correto
Correto	Correto	Correto	Correto	Não sei dizer
Correto	Correto	Correto	Não sei dizer	Correto
Correto	Não sei dizer	Correto	Não sei dizer	Não sei dizer
Correto	Correto	Correto	Correto	Correto
Errado	Errado	Correto	Não sei dizer	Não sei dizer
Correto	Correto	Correto	Correto	Correto
Correto	Correto	Correto	Correto	Correto
Correto	Correto	Correto	Correto	Correto
Correto	Correto	Correto	Correto	Não sei dizer
Correto	Correto	Correto	Correto	Correto
Errado	Errado	Correto	Correto	Não sei dizer
Correto	Correto	Correto	Correto	Não sei dizer
Correto	Correto	Correto	Não sei dizer	Correto
Correto	Correto	Correto	Correto	Correto
Correto	Correto	Correto	Correto	Correto
Correto	Correto	Correto	Correto	Correto
Correto	Correto	Correto	Correto	Não sei dizer
Correto	Correto	Correto	Correto	Correto
Correto	Correto	Correto	Errado	Não sei dizer
Correto	Correto	Correto	Correto	Correto
Correto	Correto	Correto	Correto	Correto
Correto	Correto	Correto	Correto	Não sei dizer
Correto	Correto	Correto	Correto	Correto
Correto	Correto	Correto	Correto	Correto
Correto	Correto	Correto	Não sei dizer	Correto
Correto	Correto	Correto	Correto	Correto
Correto	Não sei dizer	Correto	Correto	Não sei dizer
Correto	Errado	Correto	Errado	Correto
Correto	Correto	Correto	Correto	Correto
Correto	Correto	Correto	Correto	Não sei dizer
Correto	Correto	Correto	Não sei dizer	Correto
Correto	Correto	Correto	Correto	Correto
Correto	Correto	Correto	Correto	Correto
Correto	Errado	Correto	Correto	Correto
Correto	Errado	Correto	Correto	Não sei dizer
Correto	Não sei dizer	Correto	Não sei dizer	Correto
Correto	Errado	Correto	Errado	Não sei dizer
Correto	Correto	Correto	Correto	Não sei dizer

Correto	Correto	Correto	Correto	Correto
Correto	Correto	Correto	Não sei dizer	Não sei dizer
Correto	Correto	Correto	Não sei dizer	Correto
Correto	Correto	Correto	Correto	Correto
Correto	Correto	Correto	Correto	Correto
Correto	Não sei dizer	Correto	Correto	Não sei dizer
Correto	Correto	Correto	Correto	Não sei dizer
Correto	Correto	Correto	Correto	Não sei dizer
Correto	Errado	Correto	Correto	Correto
Correto	Errado	Correto	Correto	Correto
Correto	Correto	Correto	Não sei dizer	Não sei dizer
Não sei dizer	Não sei dizer	Correto	Errado	Não sei dizer
Correto	Correto	Correto	Correto	Não sei dizer
Correto	Não sei dizer	Correto	Correto	Não sei dizer
Correto	Errado	Correto	Errado	Não sei dizer
Correto	Correto	Correto	Errado	Correto
Correto	Correto	Correto	Correto	Correto
Correto	Não sei dizer	Correto	Correto	Não sei dizer
Correto	Correto	Correto	Errado	Correto
Correto	Não sei dizer	Correto	Correto	Não sei dizer
Correto	Não sei dizer	Correto	Não sei dizer	Não sei dizer
Correto	Correto	Correto	Não sei dizer	Correto
Correto	Correto	Correto	Correto	Correto
Correto	Correto	Correto	Correto	Não sei dizer
Correto	Errado	Correto	Errado	Não sei dizer
Correto	Errado	Correto	Não sei dizer	Não sei dizer
Correto	Correto	Correto	Errado	Correto
Correto	Correto	Correto	Não sei dizer	Correto
Correto	Não sei dizer	Correto	Não sei dizer	Não sei dizer
Correto	Não sei dizer	Correto	Correto	Correto
Errado	Errado	Correto	Correto	Correto
Correto	Correto	Correto	Errado	Não sei dizer
Correto	Correto	Correto	Não sei dizer	Correto
Correto	Errado	Correto	Correto	Correto
Correto	Correto	Correto	Correto	Correto
Correto	Correto	Correto	Correto	Não sei dizer
Correto	Correto	Correto	Errado	Correto
Errado	Correto	Correto	Não sei dizer	Não sei dizer
Correto	Correto	Correto	Errado	Correto
Correto	Correto	Correto	Errado	Correto
Correto	Correto	Correto	Correto	Correto
Correto	Correto	Correto	Correto	Correto
Correto	Não sei dizer	Correto	Correto	Não sei dizer
Correto	Correto	Correto	Errado	Correto
Correto	Correto	Correto	Correto	Correto
Correto	Correto	Correto	Correto	Não sei dizer
Correto	Correto	Correto	Correto	Correto
Correto	Correto	Correto	Correto	Correto
Correto	Errado	Correto	Errado	Não sei dizer
Correto	Correto	Correto	Errado	Não sei dizer
Correto	Correto	Correto	Correto	Correto
Errado	Correto	Correto	Não sei dizer	Não sei dizer
Correto	Correto	Correto	Correto	Correto
Correto	Correto	Correto	Correto	Não sei dizer

Correto	Correto	Correto	Correto	Correto
Correto	Correto	Correto	Errado	Não sei dizer
Correto	Correto	Correto	Errado	Não sei dizer
Correto	Correto	Correto	Correto	Correto
Correto	Correto	Correto	Correto	Não sei dizer
Correto	Errado	Correto	Correto	Correto
Correto	Errado	Correto	Correto	Não sei dizer
Correto	Correto	Correto	Não sei dizer	Correto
Correto	Correto	Correto	Correto	Não sei dizer
Correto	Errado	Correto	Correto	Não sei dizer
Correto	Correto	Correto	Correto	Correto
Correto	Correto	Correto	Correto	Correto
Correto	Correto	Correto	Correto	Correto
Correto	Correto	Correto	Errado	Não sei dizer
Correto	Correto	Correto	Correto	Correto
Correto	Errado	Correto	Correto	Correto
Correto	Correto	Correto	Correto	Correto
Correto	Correto	Correto	Não sei dizer	Não sei dizer
Correto	Correto	Correto	Correto	Correto
Correto	Não sei dizer	Correto	Não sei dizer	Correto
Correto	Não sei dizer	Correto	Errado	Errado
Correto	Errado	Correto	Correto	Não sei dizer
Correto	Correto	Correto	Correto	Não sei dizer
Correto	Correto	Correto	Correto	Não sei dizer
Correto	Não sei dizer	Correto	Errado	Correto
Correto	Correto	Correto	Não sei dizer	Não sei dizer
Correto	Correto	Correto	Errado	Correto
Correto	Correto	Correto	Errado	Não sei dizer
Correto	Correto	Correto	Correto	Correto
Correto	Correto	Correto	Errado	Não sei dizer
Correto	Errado	Correto	Não sei dizer	Não sei dizer
Correto	Correto	Correto	Errado	Correto
Correto	Correto	Correto	Errado	Não sei dizer
Correto	Não sei dizer	Correto	Não sei dizer	Não sei dizer
Correto	Errado	Correto	Não sei dizer	Não sei dizer
Correto	Correto	Correto	Errado	Correto
Correto	Correto	Correto	Correto	Correto
Correto	Errado	Correto	Errado	Não sei dizer
Errado	Errado	Correto	Não sei dizer	Não sei dizer
Correto	Correto	Correto	Correto	Correto
Correto	Correto	Correto	Correto	Correto
Correto	Correto	Correto	Correto	Não sei dizer
Correto	Correto	Correto	Errado	Não sei dizer
Correto	Correto	Correto	Não sei dizer	Não sei dizer
Correto	Correto	Correto	Correto	Correto
Correto	Errado	Correto	Errado	Não sei dizer
Correto	Correto	Correto	Correto	Correto
Errado	Errado	Não sei dizer	Não sei dizer	Não sei dizer
Correto	Correto	Correto	Não sei dizer	Não sei dizer
Correto	Errado	Correto	Não sei dizer	Não sei dizer
Errado	Errado	Correto	Não sei dizer	Correto
Correto	Correto	Correto	Errado	Correto
Correto	Errado	Correto	Errado	Não sei dizer
Correto	Errado	Correto	Errado	Não sei dizer

Correto	Correto	Correto	Errado	Correto
Correto	Correto	Correto	Correto	Correto
Correto	Correto	Correto	Correto	Correto
Correto	Errado	Correto	Errado	Não sei dizer
Correto	Correto	Correto	Correto	Não sei dizer
Correto	Correto	Correto	Correto	Correto
Correto	Correto	Correto	Correto	Correto
Correto	Errado	Correto	Correto	Correto
Correto	Correto	Correto	Não sei dizer	Não sei dizer
Correto	Correto	Correto	Correto	Correto
Errado	Errado	Errado	Correto	Correto
Correto	Correto	Correto	Errado	Não sei dizer
Correto	Errado	Correto	Errado	Não sei dizer
Correto	Correto	Correto	Correto	Correto
Correto	Correto	Correto	Correto	Correto
Correto	Correto	Correto	Correto	Correto
Correto	Errado	Correto	Correto	Correto
Correto	Errado	Correto	Errado	Correto
Não sei dizer	Não sei dizer	Correto	Não sei dizer	Não sei dizer
Correto	Correto	Correto	Correto	Correto
Correto	Correto	Correto	Errado	Correto
Correto	Correto	Correto	Não sei dizer	Não sei dizer
Correto	Correto	Correto	Correto	Correto
Correto	Correto	Errado	Errado	Não sei dizer
Correto	Errado	Correto	Não sei dizer	Não sei dizer
Correto	Correto	Correto	Correto	Correto
Correto	Correto	Correto	Errado	Correto
Correto	Correto	Correto	Errado	Correto
Correto	Correto	Correto	Correto	Correto
Correto	Correto	Correto	Correto	Correto
Correto	Correto	Correto	Correto	Correto
Correto	Correto	Correto	Errado	Não sei dizer
Correto	Correto	Correto	Errado	Correto
Correto	Correto	Correto	Não sei dizer	Correto
Correto	Errado	Correto	Correto	Correto
Correto	Correto	Correto	Correto	Correto
Correto	Correto	Correto	Correto	Não sei dizer
Errado	Não sei dizer	Correto	Errado	Correto
Correto	Correto	Correto	Errado	Correto
Correto	Correto	Correto	Correto	Correto
Correto	Correto	Correto	Correto	Correto
Correto	Correto	Correto	Correto	Correto
Correto	Correto	Correto	Correto	Errado
Correto	Correto	Correto	Correto	Não sei dizer
Errado	Errado	Correto	Errado	Não sei dizer
Correto	Errado	Correto	Não sei dizer	Não sei dizer
Correto	Correto	Correto	Não sei dizer	Não sei dizer
Correto	Correto	Correto	Errado	Não sei dizer
Correto	Correto	Correto	Errado	Não sei dizer
Correto	Errado	Correto	Errado	Correto
Correto	Correto	Correto	Não sei dizer	Não sei dizer
Errado	Errado	Correto	Errado	Não sei dizer
Não sei dizer	Não sei dizer	Correto	Não sei dizer	Não sei dizer
Correto	Correto	Errado	Não sei dizer	Não sei dizer

Correto	Não sei dizer	Não sei dizer	Não sei dizer	Não sei dizer
Correto	Não sei dizer	Correto	Correto	Não sei dizer
Correto	Errado	Correto	Correto	Não sei dizer
Correto	Correto	Correto	Errado	Correto
Errado	Errado	Correto	Não sei dizer	Não sei dizer
Correto	Correto	Correto	Correto	Correto
Correto	Correto	Correto	Errado	Não sei dizer
Correto	Errado	Correto	Errado	Errado
Correto	Errado	Correto	Correto	Correto
Correto	Correto	Correto	Correto	Não sei dizer
Correto	Correto	Correto	Correto	Correto
Correto	Correto	Correto	Errado	Não sei dizer
Correto	Correto	Correto	Não sei dizer	Não sei dizer
Correto	Correto	Correto	Não sei dizer	Não sei dizer
Correto	Correto	Correto	Correto	Correto
Correto	Errado	Correto	Não sei dizer	Não sei dizer
Correto	Não sei dizer	Não sei dizer	Correto	Não sei dizer
Errado	Errado	Correto	Não sei dizer	Não sei dizer

Q6	Q7	Q8	Q9	Q10
Não sei dizer	Não sei dizer	Não sei dizer	Não sei dizer	Não sei dizer
Não sei dizer	Não sei dizer	Correto	Correto	Não sei dizer
Não sei dizer	Não sei dizer	Não sei dizer	Não sei dizer	Não sei dizer
Não sei dizer	Não sei dizer	Não sei dizer	Não sei dizer	Correto
Não sei dizer	Não sei dizer	Não sei dizer	Correto	Correto
Não sei dizer	Não sei dizer	Não sei dizer	Não sei dizer	Correto
Não sei dizer	Errado	Correto	Correto	Correto
Correto	Errado	Correto	Correto	Correto
Não sei dizer	Não sei dizer	Não sei dizer	Não sei dizer	Correto
Correto	Errado	Correto	Correto	Correto
Não sei dizer	Correto	Não sei dizer	Não sei dizer	Errado
Não sei dizer	Não sei dizer	Errado	Correto	Errado
Correto	Correto	Não sei dizer	Correto	Correto
Correto	Não sei dizer	Correto	Correto	Correto
Errado	Correto	Não sei dizer	Correto	Errado
Não sei dizer	Não sei dizer	Não sei dizer	Não sei dizer	Não sei dizer
Correto	Correto	Correto	Correto	Correto
Não sei dizer	Não sei dizer	Não sei dizer	Não sei dizer	Não sei dizer
Correto	Correto	Correto	Correto	Não sei dizer
Correto	Correto	Correto	Correto	Correto
Não sei dizer	Não sei dizer	Correto	Correto	Correto
Não sei dizer	Não sei dizer	Correto	Não sei dizer	Correto
Correto	Não sei dizer	Correto	Correto	Correto
Não sei dizer	Não sei dizer	Não sei dizer	Correto	Correto
Não sei dizer	Não sei dizer	Correto	Correto	Correto
Correto	Não sei dizer	Correto	Correto	Correto
Não sei dizer	Correto	Correto	Correto	Correto
Correto	Não sei dizer	Não sei dizer	Não sei dizer	Correto
Correto	Correto	Errado	Correto	Correto
Errado	Correto	Errado	Correto	Correto
Correto	Correto	Correto	Correto	Correto
Correto	Não sei dizer	Correto	Correto	Correto
Correto	Errado	Correto	Correto	Correto
Correto	Não sei dizer	Errado	Não sei dizer	Correto
Correto	Não sei dizer	Não sei dizer	Correto	Correto
Não sei dizer	Correto	Correto	Correto	Correto
Correto	Correto	Correto	Correto	Correto
Errado	Errado	Correto	Correto	Correto
Correto	Não sei dizer	Correto	Não sei dizer	Correto
Não sei dizer	Correto	Não sei dizer	Correto	Correto
Não sei dizer	Correto	Correto	Correto	Correto
Não sei dizer	Correto	Não sei dizer	Correto	Correto
Não sei dizer	Não sei dizer	Não sei dizer	Não sei dizer	Correto
Correto	Errado	Correto	Errado	Correto
Não sei dizer	Correto	Não sei dizer	Correto	Correto
Não sei dizer	Não sei dizer	Correto	Não sei dizer	Correto
Correto	Não sei dizer	Correto	Não sei dizer	Correto
Não sei dizer	Não sei dizer	Não sei dizer	Correto	Não sei dizer
Correto	Não sei dizer	Correto	Correto	Correto
Não sei dizer	Não sei dizer	Não sei dizer	Não sei dizer	Não sei dizer
Não sei dizer	Não sei dizer	Não sei dizer	Não sei dizer	Não sei dizer
Correto	Correto	Correto	Correto	Correto
Não sei dizer	Não sei dizer	Não sei dizer	Não sei dizer	Não sei dizer

Correto	Não sei dizer	Não sei dizer	Correto	Errado
Não sei dizer	Não sei dizer	Correto	Não sei dizer	Correto
Não sei dizer	Não sei dizer	Não sei dizer	Errado	Não sei dizer
Não sei dizer	Não sei dizer	Correto	Não sei dizer	Correto
Correto	Errado	Não sei dizer	Correto	Correto
Correto	Correto	Correto	Correto	Correto
Não sei dizer	Não sei dizer	Não sei dizer	Não sei dizer	Não sei dizer
Não sei dizer	Não sei dizer	Não sei dizer	Não sei dizer	Não sei dizer
Correto	Correto	Correto	Correto	Correto
Correto	Não sei dizer	Correto	Correto	Correto
Não sei dizer	Não sei dizer	Errado	Não sei dizer	Correto
Correto	Errado	Correto	Correto	Correto
Não sei dizer	Não sei dizer	Não sei dizer	Não sei dizer	Não sei dizer
Não sei dizer	Não sei dizer	Correto	Não sei dizer	Não sei dizer
Não sei dizer	Não sei dizer	Não sei dizer	Não sei dizer	Não sei dizer
Não sei dizer	Correto	Não sei dizer	Não sei dizer	Correto
Correto	Não sei dizer	Não sei dizer	Não sei dizer	Correto
Não sei dizer	Não sei dizer	Não sei dizer	Não sei dizer	Não sei dizer
Correto	Não sei dizer	Não sei dizer	Não sei dizer	Errado
Não sei dizer	Não sei dizer	Não sei dizer	Não sei dizer	Correto
Não sei dizer	Não sei dizer	Não sei dizer	Correto	Correto
Correto	Correto	Correto	Correto	Correto
Não sei dizer	Não sei dizer	Correto	Não sei dizer	Correto
Correto	Não sei dizer	Correto	Correto	Correto
Não sei dizer	Não sei dizer	Não sei dizer	Não sei dizer	Correto
Correto	Não sei dizer	Não sei dizer	Correto	Errado
Correto	Correto	Correto	Correto	Correto
Não sei dizer	Não sei dizer	Não sei dizer	Não sei dizer	Não sei dizer
Correto	Não sei dizer	Correto	Correto	Correto
Correto	Correto	Correto	Correto	Errado
Errado	Não sei dizer	Não sei dizer	Não sei dizer	Não sei dizer
Não sei dizer	Não sei dizer	Correto	Não sei dizer	Errado
Não sei dizer	Não sei dizer	Correto	Não sei dizer	Correto
Não sei dizer	Não sei dizer	Não sei dizer	Não sei dizer	Correto
Não sei dizer	Não sei dizer	Não sei dizer	Não sei dizer	Não sei dizer
Correto	Correto	Não sei dizer	Correto	Correto
Errado	Não sei dizer	Errado	Não sei dizer	Correto
Não sei dizer	Não sei dizer	Correto	Correto	Correto
Correto	Correto	Não sei dizer	Correto	Correto
Não sei dizer	Não sei dizer	Não sei dizer	Não sei dizer	Correto
Não sei dizer	Correto	Não sei dizer	Não sei dizer	Correto
Não sei dizer	Não sei dizer	Não sei dizer	Correto	Não sei dizer
Correto	Não sei dizer	Correto	Não sei dizer	Correto
Não sei dizer	Não sei dizer	Não sei dizer	Não sei dizer	Não sei dizer
Não sei dizer	Não sei dizer	Não sei dizer	Correto	Correto
Não sei dizer	Não sei dizer	Não sei dizer	Não sei dizer	Não sei dizer
Não sei dizer	Não sei dizer	Correto	Correto	Correto
Não sei dizer	Não sei dizer	Não sei dizer	Não sei dizer	Correto
Não sei dizer	Correto	Não sei dizer	Correto	Correto
Não sei dizer	Não sei dizer	Correto	Não sei dizer	Não sei dizer
Não sei dizer	Correto	Correto	Correto	Não sei dizer
Correto	Errado	Correto	Não sei dizer	Correto
Correto	Correto	Não sei dizer	Correto	Correto
Não sei dizer	Não sei dizer	Não sei dizer	Não sei dizer	Não sei dizer

Correto	Não sei dizer	Correto	Correto	Correto
Não sei dizer	Não sei dizer	Não sei dizer	Não sei dizer	Não sei dizer
Não sei dizer	Não sei dizer	Não sei dizer	Não sei dizer	Não sei dizer
Não sei dizer	Não sei dizer	Não sei dizer	Correto	Errado
Não sei dizer	Não sei dizer	Não sei dizer	Não sei dizer	Correto
Não sei dizer	Correto	Não sei dizer	Correto	Errado
Não sei dizer	Não sei dizer	Correto	Correto	Errado
Não sei dizer	Não sei dizer	Correto	Correto	Não sei dizer
Não sei dizer	Não sei dizer	Não sei dizer	Não sei dizer	Não sei dizer
Não sei dizer	Não sei dizer	Não sei dizer	Não sei dizer	Não sei dizer
Correto	Não sei dizer	Não sei dizer	Correto	Errado
Correto	Não sei dizer	Correto	Correto	Correto
Não sei dizer	Não sei dizer	Não sei dizer	Não sei dizer	Não sei dizer
Correto	Correto	Correto	Correto	Não sei dizer
Não sei dizer	Errado	Não sei dizer	Não sei dizer	Correto
Não sei dizer	Não sei dizer	Não sei dizer	Não sei dizer	Não sei dizer
Correto	Correto	Errado	Correto	Correto
Não sei dizer	Não sei dizer	Não sei dizer	Não sei dizer	Não sei dizer
Não sei dizer	Não sei dizer	Não sei dizer	Não sei dizer	Não sei dizer
Errado	Correto	Correto	Correto	Correto
Não sei dizer	Não sei dizer	Não sei dizer	Não sei dizer	Correto
Não sei dizer	Não sei dizer	Não sei dizer	Correto	Correto
Correto	Correto	Correto	Correto	Correto
Não sei dizer	Não sei dizer	Correto	Não sei dizer	Correto
Não sei dizer	Não sei dizer	Não sei dizer	Não sei dizer	Não sei dizer
Não sei dizer	Não sei dizer	Não sei dizer	Não sei dizer	Não sei dizer
Não sei dizer	Não sei dizer	Não sei dizer	Não sei dizer	Não sei dizer
Não sei dizer	Não sei dizer	Não sei dizer	Não sei dizer	Não sei dizer
Correto	Correto	Não sei dizer	Correto	Não sei dizer
Não sei dizer	Errado	Correto	Correto	Correto
Correto	Correto	Correto	Correto	Correto
Não sei dizer	Não sei dizer	Não sei dizer	Correto	Não sei dizer
Não sei dizer	Correto	Correto	Correto	Correto
Não sei dizer	Não sei dizer	Não sei dizer	Correto	Correto
Errado	Não sei dizer	Não sei dizer	Correto	Não sei dizer
Não sei dizer	Correto	Não sei dizer	Não sei dizer	Não sei dizer
Não sei dizer	Não sei dizer	Não sei dizer	Não sei dizer	Não sei dizer
Não sei dizer	Não sei dizer	Errado	Correto	Correto
Correto	Não sei dizer	Correto	Correto	Correto
Não sei dizer	Não sei dizer	Não sei dizer	Não sei dizer	Não sei dizer
Não sei dizer	Correto	Não sei dizer	Correto	Correto
Correto	Não sei dizer	Não sei dizer	Não sei dizer	Não sei dizer
Não sei dizer	Não sei dizer	Correto	Correto	Não sei dizer
Não sei dizer	Não sei dizer	Não sei dizer	Não sei dizer	Não sei dizer
Não sei dizer	Correto	Correto	Correto	Correto
Não sei dizer	Errado	Não sei dizer	Não sei dizer	Não sei dizer
Correto	Não sei dizer	Não sei dizer	Não sei dizer	Correto
Correto	Correto	Errado	Correto	Correto
Não sei dizer	Não sei dizer	Não sei dizer	Não sei dizer	Não sei dizer
Não sei dizer	Não sei dizer	Não sei dizer	Não sei dizer	Não sei dizer
Errado	Não sei dizer	Não sei dizer	Correto	Correto
Não sei dizer	Não sei dizer	Não sei dizer	Não sei dizer	Correto
Não sei dizer	Não sei dizer	Não sei dizer	Não sei dizer	Não sei dizer
Correto	Não sei dizer	Correto	Correto	Correto

Não sei dizer	Não sei dizer	Não sei dizer	Não sei dizer	Não sei dizer
Não sei dizer	Errado	Não sei dizer	Não sei dizer	Errado
Não sei dizer	Não sei dizer	Correto	Correto	Correto
Correto	Correto	Correto	Correto	Correto
Não sei dizer	Correto	Não sei dizer	Não sei dizer	Correto
Não sei dizer	Não sei dizer	Correto	Correto	Correto
Não sei dizer	Não sei dizer	Correto	Correto	Correto
Não sei dizer	Não sei dizer	Não sei dizer	Não sei dizer	Não sei dizer
Correto	Não sei dizer	Correto	Correto	Correto
Não sei dizer	Não sei dizer	Não sei dizer	Não sei dizer	Não sei dizer
Não sei dizer	Não sei dizer	Não sei dizer	Não sei dizer	Correto
Correto	Não sei dizer	Não sei dizer	Não sei dizer	Correto
Não sei dizer	Não sei dizer	Correto	Não sei dizer	Correto
Não sei dizer	Correto	Correto	Correto	Correto
Não sei dizer	Não sei dizer	Correto	Correto	Correto
Correto	Não sei dizer	Correto	Não sei dizer	Correto
Não sei dizer	Não sei dizer	Correto	Correto	Correto
Não sei dizer	Não sei dizer	Não sei dizer	Não sei dizer	Não sei dizer
Correto	Não sei dizer	Não sei dizer	Não sei dizer	Correto
Não sei dizer	Não sei dizer	Correto	Não sei dizer	Não sei dizer
Não sei dizer	Não sei dizer	Correto	Correto	Correto
Não sei dizer	Não sei dizer	Correto	Não sei dizer	Não sei dizer
Não sei dizer	Não sei dizer	Não sei dizer	Não sei dizer	Não sei dizer
Não sei dizer	Correto	Não sei dizer	Correto	Não sei dizer
Correto	Não sei dizer	Não sei dizer	Correto	Correto
Não sei dizer	Não sei dizer	Errado	Correto	Não sei dizer
Não sei dizer	Não sei dizer	Não sei dizer	Não sei dizer	Não sei dizer
Correto	Não sei dizer	Não sei dizer	Correto	Correto
Correto	Correto	Correto	Correto	Correto
Não sei dizer	Não sei dizer	Não sei dizer	Correto	Correto
Não sei dizer	Não sei dizer	Não sei dizer	Não sei dizer	Não sei dizer
Correto	Correto	Não sei dizer	Correto	Correto
Não sei dizer	Não sei dizer	Correto	Correto	Correto
Correto	Correto	Correto	Correto	Correto
Não sei dizer	Não sei dizer	Correto	Não sei dizer	Não sei dizer
Errado	Não sei dizer	Não sei dizer	Correto	Errado
Errado	Errado	Correto	Errado	Correto
Errado	Correto	Não sei dizer	Correto	Correto
Não sei dizer	Não sei dizer	Não sei dizer	Não sei dizer	Não sei dizer
Não sei dizer	Não sei dizer	Não sei dizer	Não sei dizer	Não sei dizer
Correto	Não sei dizer	Correto	Correto	Correto
Não sei dizer	Não sei dizer	Não sei dizer	Não sei dizer	Não sei dizer
Não sei dizer	Não sei dizer	Correto	Não sei dizer	Correto
Correto	Não sei dizer	Não sei dizer	Não sei dizer	Correto
Correto	Não sei dizer	Correto	Correto	Correto
Não sei dizer	Não sei dizer	Correto	Correto	Correto
Não sei dizer	Não sei dizer	Não sei dizer	Correto	Não sei dizer
Não sei dizer	Não sei dizer	Não sei dizer	Não sei dizer	Não sei dizer
Não sei dizer	Não sei dizer	Não sei dizer	Correto	Não sei dizer
Correto	Correto	Correto	Correto	Correto
Não sei dizer	Não sei dizer	Não sei dizer	Não sei dizer	Não sei dizer
Não sei dizer	Não sei dizer	Não sei dizer	Não sei dizer	Não sei dizer
Não sei dizer	Não sei dizer	Não sei dizer	Não sei dizer	Não sei dizer
Não sei dizer	Não sei dizer	Não sei dizer	Não sei dizer	Não sei dizer

Não sei dizer	Não sei dizer	Não sei dizer	Não sei dizer	Não sei dizer
Não sei dizer	Não sei dizer	Correto	Não sei dizer	Correto
Não sei dizer	Correto	Correto	Correto	Correto
Não sei dizer	Não sei dizer	Não sei dizer	Não sei dizer	Não sei dizer
Não sei dizer	Não sei dizer	Não sei dizer	Correto	Não sei dizer
Correto	Correto	Errado	Errado	Errado
Não sei dizer	Não sei dizer	Não sei dizer	Não sei dizer	Não sei dizer
Não sei dizer	Não sei dizer	Não sei dizer	Não sei dizer	Não sei dizer
Correto	Não sei dizer	Correto	Correto	Correto
Errado	Correto	Correto	Correto	Errado
Não sei dizer	Não sei dizer	Não sei dizer	Não sei dizer	Não sei dizer
Não sei dizer	Não sei dizer	Correto	Não sei dizer	Correto
Não sei dizer	Não sei dizer	Correto	Correto	Errado
Não sei dizer	Não sei dizer	Não sei dizer	Não sei dizer	Não sei dizer
Não sei dizer	Não sei dizer	Correto	Não sei dizer	Correto
Não sei dizer	Não sei dizer	Correto	Correto	Correto
Não sei dizer	Não sei dizer	Correto	Correto	Correto
Correto	Correto	Correto	Correto	Correto
Correto	Não sei dizer	Correto	Correto	Correto
Não sei dizer	Não sei dizer	Não sei dizer	Correto	Correto
Não sei dizer	Não sei dizer	Correto	Não sei dizer	Correto
Correto	Correto	Errado	Correto	Correto
Correto	Correto	Correto	Correto	Correto
Correto	Não sei dizer	Não sei dizer	Correto	Correto
Não sei dizer	Não sei dizer	Não sei dizer	Não sei dizer	Não sei dizer
Correto	Errado	Correto	Não sei dizer	Correto
Não sei dizer	Não sei dizer	Não sei dizer	Não sei dizer	Não sei dizer
Não sei dizer	Não sei dizer	Não sei dizer	Não sei dizer	Correto
Correto	Não sei dizer	Correto	Não sei dizer	Errado
Correto	Correto	Correto	Correto	Correto
Não sei dizer	Não sei dizer	Correto	Correto	Correto
Não sei dizer	Não sei dizer	Não sei dizer	Não sei dizer	Não sei dizer
Não sei dizer	Correto	Não sei dizer	Não sei dizer	Correto
Não sei dizer	Não sei dizer	Não sei dizer	Não sei dizer	Correto
Correto	Correto	Correto	Correto	Correto
Não sei dizer	Não sei dizer	Correto	Correto	Correto
Correto	Errado	Correto	Correto	Correto
Não sei dizer	Não sei dizer	Não sei dizer	Não sei dizer	Não sei dizer
Correto	Não sei dizer	Não sei dizer	Correto	Correto
Não sei dizer	Não sei dizer	Não sei dizer	Não sei dizer	Não sei dizer
Errado	Correto	Correto	Correto	Correto
Não sei dizer	Não sei dizer	Correto	Correto	Correto
Não sei dizer	Não sei dizer	Não sei dizer	Não sei dizer	Não sei dizer
Correto	Não sei dizer	Errado	Correto	Correto
Errado	Errado	Errado	Não sei dizer	Correto
Não sei dizer	Não sei dizer	Não sei dizer	Correto	Correto
Correto	Não sei dizer	Não sei dizer	Não sei dizer	Correto
Não sei dizer	Não sei dizer	Não sei dizer	Não sei dizer	Não sei dizer
Não sei dizer	Correto	Correto	Correto	Correto
Correto	Correto	Correto	Correto	Correto
Não sei dizer	Correto	Correto	Não sei dizer	Correto
Não sei dizer	Não sei dizer	Não sei dizer	Correto	Não sei dizer
Errado	Correto	Não sei dizer	Correto	Correto
Correto	Não sei dizer	Não sei dizer	Não sei dizer	Não sei dizer

Não sei dizer	Não sei dizer	Não sei dizer	Não sei dizer	Não sei dizer
Errado	Correto	Não sei dizer	Correto	Correto
Errado	Correto	Correto	Correto	Correto
Não sei dizer	Não sei dizer	Não sei dizer	Não sei dizer	Não sei dizer
Não sei dizer	Correto	Não sei dizer	Correto	Correto
Não sei dizer	Não sei dizer	Não sei dizer	Não sei dizer	Não sei dizer
Não sei dizer	Não sei dizer	Correto	Correto	Correto
Não sei dizer	Não sei dizer	Errado	Correto	Correto
Correto	Correto	Correto	Correto	Correto
Correto	Errado	Correto	Correto	Errado
Não sei dizer	Não sei dizer	Não sei dizer	Não sei dizer	Correto
Correto	Não sei dizer	Correto	Correto	Correto
Errado	Correto	Correto	Errado	Correto
Correto	Não sei dizer	Correto	Correto	Correto
Correto	Correto	Correto	Correto	Correto
Não sei dizer	Não sei dizer	Correto	Correto	Correto
Não sei dizer	Não sei dizer	Não sei dizer	Não sei dizer	Correto
Correto	Correto	Correto	Correto	Correto
Não sei dizer	Não sei dizer	Não sei dizer	Não sei dizer	Correto
Correto	Correto	Correto	Correto	Correto
Não sei dizer	Não sei dizer	Não sei dizer	Não sei dizer	Não sei dizer
Correto	Correto	Correto	Correto	Correto
Correto	Não sei dizer	Correto	Correto	Correto
Correto	Correto	Correto	Correto	Não sei dizer
Não sei dizer	Não sei dizer	Não sei dizer	Não sei dizer	Não sei dizer
Correto	Não sei dizer	Correto	Correto	Correto
Não sei dizer	Não sei dizer	Correto	Não sei dizer	Não sei dizer
Não sei dizer	Não sei dizer	Não sei dizer	Não sei dizer	Não sei dizer
Correto	Não sei dizer	Correto	Não sei dizer	Correto
Errado	Errado	Correto	Correto	Correto
Correto	Não sei dizer	Correto	Correto	Correto
Correto	Correto	Errado	Correto	Correto
Correto	Não sei dizer	Correto	Correto	Correto
Correto	Correto	Correto	Correto	Correto
Não sei dizer	Não sei dizer	Não sei dizer	Não sei dizer	Não sei dizer
Errado	Não sei dizer	Correto	Correto	Correto
Não sei dizer	Correto	Não sei dizer	Correto	Errado
Não sei dizer	Não sei dizer	Não sei dizer	Não sei dizer	Não sei dizer
Correto	Correto	Correto	Correto	Correto
Correto	Correto	Correto	Correto	Correto
Correto	Não sei dizer	Correto	Não sei dizer	Correto
Correto	Correto	Correto	Correto	Correto
Não sei dizer	Não sei dizer	Não sei dizer	Não sei dizer	Não sei dizer
Errado	Não sei dizer	Não sei dizer	Não sei dizer	Correto
Não sei dizer	Não sei dizer	Correto	Não sei dizer	Correto
Não sei dizer	Não sei dizer	Não sei dizer	Correto	Correto
Correto	Não sei dizer	Correto	Correto	Correto
Correto	Correto	Correto	Correto	Correto
Correto	Não sei dizer	Não sei dizer	Correto	Correto
Não sei dizer	Não sei dizer	Não sei dizer	Não sei dizer	Correto
Não sei dizer	Não sei dizer	Não sei dizer	Não sei dizer	Não sei dizer
Correto	Não sei dizer	Correto	Correto	Correto
Não sei dizer	Não sei dizer	Não sei dizer	Não sei dizer	Não sei dizer
Não sei dizer	Não sei dizer	Correto	Não sei dizer	Correto

Não sei dizer	Não sei dizer	Correto	Não sei dizer	Não sei dizer
Não sei dizer	Não sei dizer	Não sei dizer	Não sei dizer	Não sei dizer
Não sei dizer	Correto	Errado	Correto	Correto
Não sei dizer	Correto	Correto	Correto	Não sei dizer
Correto	Correto	Correto	Correto	Correto
Não sei dizer	Não sei dizer	Não sei dizer	Não sei dizer	Correto
Não sei dizer	Não sei dizer	Correto	Correto	Correto
Não sei dizer	Não sei dizer	Não sei dizer	Não sei dizer	Não sei dizer
Não sei dizer	Não sei dizer	Não sei dizer	Não sei dizer	Não sei dizer
Correto	Errado	Correto	Errado	Correto
Correto	Não sei dizer	Não sei dizer	Correto	Correto
Não sei dizer	Não sei dizer	Não sei dizer	Não sei dizer	Não sei dizer
Não sei dizer	Não sei dizer	Não sei dizer	Não sei dizer	Não sei dizer
Não sei dizer	Não sei dizer	Não sei dizer	Correto	Correto
Não sei dizer	Não sei dizer	Não sei dizer	Não sei dizer	Não sei dizer
Não sei dizer	Não sei dizer	Errado	Não sei dizer	Correto
Não sei dizer	Não sei dizer	Não sei dizer	Correto	Não sei dizer
Não sei dizer	Não sei dizer	Correto	Não sei dizer	Correto
Correto	Não sei dizer	Não sei dizer	Não sei dizer	Correto
Não sei dizer	Não sei dizer	Não sei dizer	Não sei dizer	Correto
Não sei dizer	Não sei dizer	Não sei dizer	Não sei dizer	Não sei dizer
Não sei dizer	Não sei dizer	Não sei dizer	Não sei dizer	Correto
Correto	Não sei dizer	Correto	Correto	Correto
Não sei dizer	Não sei dizer	Não sei dizer	Não sei dizer	Não sei dizer
Não sei dizer	Não sei dizer	Correto	Não sei dizer	Não sei dizer
Não sei dizer	Não sei dizer	Não sei dizer	Não sei dizer	Não sei dizer
Não sei dizer	Correto	Errado	Não sei dizer	Correto
Não sei dizer	Correto	Correto	Não sei dizer	Correto
Não sei dizer	Não sei dizer	Não sei dizer	Não sei dizer	Não sei dizer
Errado	Não sei dizer	Correto	Correto	Correto
Correto	Correto	Correto	Correto	Correto
Não sei dizer	Não sei dizer	Não sei dizer	Não sei dizer	Não sei dizer
Não sei dizer	Correto	Correto	Correto	Correto
Não sei dizer	Não sei dizer	Não sei dizer	Correto	Correto
Correto	Não sei dizer	Correto	Correto	Correto
Não sei dizer	Não sei dizer	Não sei dizer	Não sei dizer	Não sei dizer
Correto	Correto	Correto	Correto	Correto
Não sei dizer	Correto	Não sei dizer	Correto	Não sei dizer
Não sei dizer	Não sei dizer	Não sei dizer	Não sei dizer	Correto
Errado	Correto	Errado	Correto	Correto
Errado	Não sei dizer	Correto	Correto	Não sei dizer
Correto	Não sei dizer	Correto	Correto	Correto
Não sei dizer	Não sei dizer	Não sei dizer	Não sei dizer	Correto
Não sei dizer	Não sei dizer	Correto	Correto	Correto
Correto	Não sei dizer	Correto	Correto	Correto
Não sei dizer	Não sei dizer	Não sei dizer	Correto	Correto
Correto	Não sei dizer	Correto	Correto	Correto
Correto	Não sei dizer	Não sei dizer	Correto	Correto
Não sei dizer	Não sei dizer	Não sei dizer	Não sei dizer	Não sei dizer
Não sei dizer	Não sei dizer	Não sei dizer	Não sei dizer	Não sei dizer
Não sei dizer	Não sei dizer	Correto	Correto	Correto
Não sei dizer	Não sei dizer	Não sei dizer	Não sei dizer	Correto
Não sei dizer	Correto	Não sei dizer	Correto	Correto
Não sei dizer	Correto	Não sei dizer	Correto	Correto

Correto	Correto	Correto	Correto	Correto
Não sei dizer	Não sei dizer	Correto	Não sei dizer	Correto
Não sei dizer	Não sei dizer	Não sei dizer	Não sei dizer	Não sei dizer
Correto	Correto	Correto	Correto	Correto
Não sei dizer	Não sei dizer	Não sei dizer	Não sei dizer	Não sei dizer
Não sei dizer	Correto	Correto	Correto	Correto
Não sei dizer	Não sei dizer	Não sei dizer	Não sei dizer	Não sei dizer
Correto	Não sei dizer	Correto	Não sei dizer	Correto
Não sei dizer	Não sei dizer	Não sei dizer	Não sei dizer	Não sei dizer
Não sei dizer	Não sei dizer	Não sei dizer	Não sei dizer	Não sei dizer
Não sei dizer	Não sei dizer	Correto	Correto	Não sei dizer
Correto	Correto	Correto	Não sei dizer	Correto
Errado	Correto	Não sei dizer	Correto	Correto
Não sei dizer	Não sei dizer	Não sei dizer	Não sei dizer	Correto
Não sei dizer	Correto	Não sei dizer	Correto	Correto
Errado	Correto	Errado	Correto	Errado
Não sei dizer	Não sei dizer	Correto	Não sei dizer	Correto
Não sei dizer	Não sei dizer	Errado	Não sei dizer	Correto
Não sei dizer	Correto	Não sei dizer	Correto	Não sei dizer
Não sei dizer	Não sei dizer	Não sei dizer	Não sei dizer	Não sei dizer
Correto	Não sei dizer	Não sei dizer	Não sei dizer	Correto
Correto	Não sei dizer	Correto	Correto	Não sei dizer
Não sei dizer	Não sei dizer	Não sei dizer	Não sei dizer	Correto
Não sei dizer	Não sei dizer	Não sei dizer	Correto	Correto
Não sei dizer	Não sei dizer	Não sei dizer	Correto	Correto
Não sei dizer	Não sei dizer	Não sei dizer	Não sei dizer	Não sei dizer
Correto	Correto	Não sei dizer	Não sei dizer	Correto
Não sei dizer	Não sei dizer	Correto	Não sei dizer	Correto
Não sei dizer	Correto	Correto	Correto	Errado
Não sei dizer	Não sei dizer	Não sei dizer	Não sei dizer	Não sei dizer
Não sei dizer	Não sei dizer	Não sei dizer	Não sei dizer	Não sei dizer
Correto	Correto	Correto	Correto	Correto
Não sei dizer	Não sei dizer	Não sei dizer	Não sei dizer	Não sei dizer
Não sei dizer	Não sei dizer	Não sei dizer	Não sei dizer	Não sei dizer
Não sei dizer	Não sei dizer	Não sei dizer	Não sei dizer	Correto
Correto	Correto	Errado	Não sei dizer	Correto
Não sei dizer	Não sei dizer	Não sei dizer	Correto	Correto
Não sei dizer	Não sei dizer	Não sei dizer	Não sei dizer	Não sei dizer
Não sei dizer	Não sei dizer	Não sei dizer	Correto	Correto
Correto	Não sei dizer	Correto	Correto	Correto
Correto	Correto	Correto	Correto	Correto
Não sei dizer	Não sei dizer	Não sei dizer	Não sei dizer	Não sei dizer
Não sei dizer	Não sei dizer	Não sei dizer	Correto	Não sei dizer
Não sei dizer	Não sei dizer	Não sei dizer	Não sei dizer	Não sei dizer
Correto	Não sei dizer	Não sei dizer	Correto	Correto
Não sei dizer	Não sei dizer	Correto	Não sei dizer	Correto
Não sei dizer	Não sei dizer	Correto	Correto	Correto
Não sei dizer	Não sei dizer	Não sei dizer	Não sei dizer	Não sei dizer
Não sei dizer	Não sei dizer	Não sei dizer	Correto	Correto
Não sei dizer	Não sei dizer	Não sei dizer	Não sei dizer	Não sei dizer
Não sei dizer	Não sei dizer	Não sei dizer	Correto	Correto
Correto	Errado	Correto	Correto	Correto
Não sei dizer	Não sei dizer	Não sei dizer	Não sei dizer	Correto
Não sei dizer	Errado	Correto	Correto	Correto

Correto	Errado	Correto	Correto	Correto
Correto	Correto	Correto	Correto	Correto
Correto	Correto	Correto	Correto	Correto
Não sei dizer	Não sei dizer	Não sei dizer	Não sei dizer	Não sei dizer
Não sei dizer	Não sei dizer	Correto	Correto	Correto
Não sei dizer	Errado	Correto	Correto	Correto
Correto	Correto	Errado	Correto	Errado
Correto	Não sei dizer	Correto	Correto	Correto
Não sei dizer	Não sei dizer	Correto	Não sei dizer	Não sei dizer
Correto	Não sei dizer	Não sei dizer	Correto	Não sei dizer
Errado	Não sei dizer	Correto	Correto	Correto
Não sei dizer	Não sei dizer	Não sei dizer	Não sei dizer	Não sei dizer
Correto	Correto	Correto	Correto	Correto
Correto	Correto	Correto	Correto	Correto
Errado	Correto	Correto	Correto	Correto
Correto	Correto	Correto	Correto	Correto
Correto	Correto	Correto	Correto	Correto
Correto	Correto	Correto	Correto	Correto
Não sei dizer	Não sei dizer	Não sei dizer	Não sei dizer	Não sei dizer
Correto	Correto	Correto	Correto	Correto
Correto	Errado	Não sei dizer	Correto	Correto
Não sei dizer	Não sei dizer	Não sei dizer	Não sei dizer	Correto
Errado	Errado	Correto	Correto	Errado
Não sei dizer	Não sei dizer	Correto	Não sei dizer	Não sei dizer
Não sei dizer	Não sei dizer	Não sei dizer	Não sei dizer	Não sei dizer
Correto	Errado	Correto	Correto	Correto
Correto	Correto	Correto	Correto	Correto
Correto	Errado	Não sei dizer	Não sei dizer	Correto
Errado	Correto	Correto	Correto	Correto
Errado	Correto	Correto	Correto	Correto
Correto	Correto	Não sei dizer	Correto	Correto
Não sei dizer	Não sei dizer	Não sei dizer	Não sei dizer	Não sei dizer
Correto	Correto	Correto	Correto	Correto
Não sei dizer	Errado	Correto	Correto	Correto
Correto	Correto	Correto	Correto	Errado
Correto	Errado	Correto	Correto	Correto
Não sei dizer	Não sei dizer	Não sei dizer	Não sei dizer	Não sei dizer
Correto	Errado	Correto	Errado	Correto
Correto	Errado	Correto	Não sei dizer	Correto
Errado	Correto	Correto	Correto	Correto
Correto	Não sei dizer	Correto	Correto	Correto
Correto	Correto	Errado	Correto	Errado
Correto	Correto	Correto	Correto	Correto
Não sei dizer	Não sei dizer	Não sei dizer	Não sei dizer	Não sei dizer
Não sei dizer	Errado	Não sei dizer	Não sei dizer	Não sei dizer
Não sei dizer	Não sei dizer	Não sei dizer	Não sei dizer	Não sei dizer
Não sei dizer	Não sei dizer	Não sei dizer	Não sei dizer	Correto
Não sei dizer	Não sei dizer	Não sei dizer	Não sei dizer	Não sei dizer
Não sei dizer	Não sei dizer	Não sei dizer	Não sei dizer	Não sei dizer
Correto	Correto	Correto	Correto	Correto
Não sei dizer	Não sei dizer	Não sei dizer	Não sei dizer	Não sei dizer
Não sei dizer	Não sei dizer	Não sei dizer	Não sei dizer	Não sei dizer
Não sei dizer	Não sei dizer	Não sei dizer	Não sei dizer	Não sei dizer
Não sei dizer	Não sei dizer	Não sei dizer	Não sei dizer	Não sei dizer

Não sei dizer	Não sei dizer	Não sei dizer	Não sei dizer	Correto
Não sei dizer	Não sei dizer	Não sei dizer	Não sei dizer	Não sei dizer
Não sei dizer	Não sei dizer	Não sei dizer	Não sei dizer	Não sei dizer
Correto	Não sei dizer	Não sei dizer	Não sei dizer	Correto
Não sei dizer	Não sei dizer	Não sei dizer	Correto	Não sei dizer
Não sei dizer	Não sei dizer	Correto	Correto	Correto
Correto	Não sei dizer	Não sei dizer	Não sei dizer	Correto
Correto	Errado	Correto	Correto	Correto
Correto	Não sei dizer	Correto	Correto	Correto
Não sei dizer	Não sei dizer	Não sei dizer	Não sei dizer	Não sei dizer
Correto	Correto	Correto	Correto	Correto
Não sei dizer	Não sei dizer	Não sei dizer	Não sei dizer	Não sei dizer
Não sei dizer	Não sei dizer	Correto	Não sei dizer	Não sei dizer
Não sei dizer	Não sei dizer	Não sei dizer	Não sei dizer	Não sei dizer
Correto	Correto	Correto	Correto	Correto
Não sei dizer	Não sei dizer	Não sei dizer	Não sei dizer	Não sei dizer
Não sei dizer	Não sei dizer	Não sei dizer	Não sei dizer	Não sei dizer
Não sei dizer	Correto	Correto	Correto	Correto

Q11	Q12	Q13	Q14	Q15
Não sei dizer	Não sei dizer	Não sei dizer	Não sei dizer	Não sei dizer
Não sei dizer	Correto	Não sei dizer	Não sei dizer	Não sei dizer
Não sei dizer	Não sei dizer	Não sei dizer	Não sei dizer	Não sei dizer
Não sei dizer	Correto	Não sei dizer	Não sei dizer	Correto
Correto	Correto	Correto	Correto	Correto
Não sei dizer	Não sei dizer	Não sei dizer	Não sei dizer	Correto
Não sei dizer	Não sei dizer	Não sei dizer	Correto	Correto
Correto	Correto	Não sei dizer	Correto	Correto
Correto	Correto	Não sei dizer	Não sei dizer	Não sei dizer
Errado	Correto	Correto	Correto	Correto
Correto	Correto	Não sei dizer	Correto	Correto
Não sei dizer	Não sei dizer	Não sei dizer	Correto	Correto
Correto	Correto	Correto	Correto	Correto
Correto	Correto	Correto	Correto	Correto
Correto	Correto	Correto	Correto	Correto
Não sei dizer	Não sei dizer	Não sei dizer	Não sei dizer	Não sei dizer
Correto	Correto	Correto	Correto	Correto
Não sei dizer	Não sei dizer	Não sei dizer	Não sei dizer	Não sei dizer
Não sei dizer	Não sei dizer	Não sei dizer	Correto	Correto
Correto	Correto	Correto	Correto	Correto
Não sei dizer	Correto	Não sei dizer	Não sei dizer	Não sei dizer
Correto	Correto	Não sei dizer	Correto	Correto
Correto	Correto	Não sei dizer	Correto	Correto
Correto	Correto	Não sei dizer	Correto	Correto
Correto	Correto	Não sei dizer	Correto	Correto
Correto	Correto	Não sei dizer	Correto	Correto
Correto	Correto	Correto	Correto	Correto
Não sei dizer	Não sei dizer	Não sei dizer	Não sei dizer	Correto
Correto	Correto	Correto	Correto	Correto
Correto	Correto	Correto	Correto	Correto
Não sei dizer	Correto	Não sei dizer	Correto	Não sei dizer
Correto	Correto	Correto	Não sei dizer	Correto
Correto	Correto	Correto	Não sei dizer	Correto
Correto	Correto	Correto	Não sei dizer	Correto
Correto	Correto	Correto	Correto	Correto
Não sei dizer	Correto	Não sei dizer	Correto	Correto
Correto	Correto	Não sei dizer	Correto	Correto
Correto	Correto	Correto	Correto	Correto
Correto	Correto	Não sei dizer	Não sei dizer	Correto
Correto	Correto	Não sei dizer	Correto	Correto
Correto	Correto	Correto	Correto	Correto
Correto	Correto	Correto	Correto	Correto
Não sei dizer	Correto	Não sei dizer	Não sei dizer	Correto
Errado	Correto	Correto	Correto	Correto
Correto	Correto	Não sei dizer	Correto	Não sei dizer
Correto	Correto	Correto	Correto	Correto
Correto	Correto	Correto	Correto	Correto
Correto	Correto	Não sei dizer	Correto	Correto
Correto	Correto	Correto	Correto	Correto
Não sei dizer	Não sei dizer	Não sei dizer	Não sei dizer	Não sei dizer
Não sei dizer	Não sei dizer	Não sei dizer	Não sei dizer	Não sei dizer
Não sei dizer	Correto	Correto	Não sei dizer	Correto
Não sei dizer	Não sei dizer	Não sei dizer	Não sei dizer	Não sei dizer

Correto	Correto	Não sei dizer	Correto	Correto
Não sei dizer	Correto	Não sei dizer	Correto	Não sei dizer
Não sei dizer	Não sei dizer	Não sei dizer	Não sei dizer	Não sei dizer
Não sei dizer	Não sei dizer	Não sei dizer	Não sei dizer	Correto
Correto	Correto	Não sei dizer	Correto	Correto
Correto	Correto	Não sei dizer	Correto	Correto
Não sei dizer	Não sei dizer	Não sei dizer	Não sei dizer	Não sei dizer
Não sei dizer	Não sei dizer	Não sei dizer	Não sei dizer	Não sei dizer
Correto	Correto	Correto	Correto	Correto
Correto	Correto	Correto	Correto	Correto
Correto	Correto	Não sei dizer	Correto	Correto
Correto	Correto	Correto	Não sei dizer	Não sei dizer
Não sei dizer	Não sei dizer	Não sei dizer	Não sei dizer	Não sei dizer
Correto	Correto	Não sei dizer	Não sei dizer	Correto
Correto	Correto	Correto	Não sei dizer	Correto
Errado	Correto	Correto	Correto	Correto
Correto	Correto	Correto	Não sei dizer	Correto
Não sei dizer	Não sei dizer	Não sei dizer	Não sei dizer	Correto
Correto	Não sei dizer	Não sei dizer	Não sei dizer	Não sei dizer
Não sei dizer	Não sei dizer	Não sei dizer	Não sei dizer	Correto
Correto	Correto	Correto	Correto	Correto
Correto	Correto	Correto	Correto	Correto
Não sei dizer	Não sei dizer	Não sei dizer	Não sei dizer	Não sei dizer
Correto	Correto	Errado	Não sei dizer	Correto
Correto	Correto	Não sei dizer	Não sei dizer	Correto
Correto	Correto	Correto	Não sei dizer	Correto
Correto	Correto	Não sei dizer	Correto	Correto
Não sei dizer	Não sei dizer	Não sei dizer	Não sei dizer	Não sei dizer
Correto	Correto	Não sei dizer	Correto	Não sei dizer
Correto	Correto	Correto	Correto	Correto
Correto	Correto	Não sei dizer	Não sei dizer	Correto
Correto	Correto	Não sei dizer	Não sei dizer	Correto
Correto	Não sei dizer	Não sei dizer	Não sei dizer	Não sei dizer
Não sei dizer	Não sei dizer	Não sei dizer	Não sei dizer	Não sei dizer
Não sei dizer	Não sei dizer	Não sei dizer	Não sei dizer	Não sei dizer
Correto	Correto	Correto	Correto	Correto
Correto	Correto	Correto	Não sei dizer	Correto
Não sei dizer	Correto	Não sei dizer	Correto	Correto
Correto	Correto	Correto	Correto	Correto
Não sei dizer	Não sei dizer	Não sei dizer	Correto	Correto
Correto	Correto	Não sei dizer	Correto	Correto
Correto	Correto	Não sei dizer	Correto	Correto
Correto	Correto	Não sei dizer	Não sei dizer	Correto
Não sei dizer	Não sei dizer	Não sei dizer	Não sei dizer	Não sei dizer
Correto	Correto	Não sei dizer	Não sei dizer	Não sei dizer
Correto	Correto	Não sei dizer	Correto	Correto
Não sei dizer	Correto	Correto	Correto	Correto
Errado	Não sei dizer	Não sei dizer	Não sei dizer	Errado
Correto	Correto	Não sei dizer	Correto	Correto
Não sei dizer	Não sei dizer	Não sei dizer	Não sei dizer	Não sei dizer
Correto	Correto	Não sei dizer	Correto	Não sei dizer
Correto	Não sei dizer	Não sei dizer	Não sei dizer	Correto
Correto	Correto	Não sei dizer	Correto	Correto
Não sei dizer	Não sei dizer	Não sei dizer	Não sei dizer	Não sei dizer

Correto	Correto	Correto	Correto	Correto
Não sei dizer	Não sei dizer	Não sei dizer	Não sei dizer	Não sei dizer
Não sei dizer	Não sei dizer	Não sei dizer	Não sei dizer	Não sei dizer
Não sei dizer	Não sei dizer	Não sei dizer	Correto	Correto
Não sei dizer	Não sei dizer	Não sei dizer	Correto	Correto
Correto	Correto	Não sei dizer	Errado	Correto
Correto	Correto	Correto	Correto	Correto
Não sei dizer	Não sei dizer	Não sei dizer	Não sei dizer	Não sei dizer
Correto	Correto	Não sei dizer	Não sei dizer	Não sei dizer
Não sei dizer	Não sei dizer	Não sei dizer	Não sei dizer	Não sei dizer
Não sei dizer	Correto	Não sei dizer	Correto	Não sei dizer
Correto	Correto	Não sei dizer	Correto	Correto
Não sei dizer	Não sei dizer	Não sei dizer	Não sei dizer	Correto
Correto	Correto	Correto	Correto	Correto
Não sei dizer	Não sei dizer	Não sei dizer	Não sei dizer	Correto
Não sei dizer	Não sei dizer	Não sei dizer	Correto	Não sei dizer
Correto	Correto	Correto	Correto	Correto
Não sei dizer	Não sei dizer	Não sei dizer	Não sei dizer	Não sei dizer
Não sei dizer	Correto	Correto	Correto	Correto
Correto	Correto	Correto	Correto	Correto
Não sei dizer	Correto	Não sei dizer	Não sei dizer	Correto
Errado	Correto	Correto	Correto	Correto
Correto	Correto	Não sei dizer	Correto	Correto
Correto	Correto	Não sei dizer	Não sei dizer	Correto
Não sei dizer	Não sei dizer	Não sei dizer	Não sei dizer	Não sei dizer
Não sei dizer	Não sei dizer	Não sei dizer	Não sei dizer	Não sei dizer
Não sei dizer	Não sei dizer	Não sei dizer	Não sei dizer	Não sei dizer
Não sei dizer	Não sei dizer	Não sei dizer	Não sei dizer	Não sei dizer
Correto	Correto	Correto	Correto	Correto
Correto	Correto	Correto	Correto	Correto
Correto	Correto	Não sei dizer	Correto	Correto
Não sei dizer	Não sei dizer	Não sei dizer	Não sei dizer	Não sei dizer
Errado	Não sei dizer	Correto	Correto	Correto
Correto	Correto	Não sei dizer	Correto	Correto
Correto	Correto	Correto	Correto	Correto
Correto	Correto	Correto	Não sei dizer	Correto
Não sei dizer	Não sei dizer	Não sei dizer	Não sei dizer	Não sei dizer
Correto	Correto	Não sei dizer	Correto	Não sei dizer
Correto	Correto	Não sei dizer	Correto	Correto
Correto	Correto	Não sei dizer	Não sei dizer	Correto
Não sei dizer	Correto	Correto	Correto	Correto
Correto	Correto	Correto	Correto	Correto
Não sei dizer	Correto	Não sei dizer	Correto	Correto
Não sei dizer	Não sei dizer	Não sei dizer	Não sei dizer	Não sei dizer
Correto	Correto	Não sei dizer	Correto	Correto
Errado	Correto	Não sei dizer	Não sei dizer	Não sei dizer
Correto	Correto	Não sei dizer	Correto	Correto
Correto	Correto	Correto	Correto	Correto
Não sei dizer	Não sei dizer	Não sei dizer	Não sei dizer	Não sei dizer
Não sei dizer	Não sei dizer	Não sei dizer	Não sei dizer	Não sei dizer
Correto	Correto	Não sei dizer	Correto	Correto
Não sei dizer	Correto	Não sei dizer	Não sei dizer	Correto
Não sei dizer	Não sei dizer	Não sei dizer	Não sei dizer	Não sei dizer
Correto	Correto	Correto	Correto	Correto

Não sei dizer	Não sei dizer	Não sei dizer	Não sei dizer	Não sei dizer
Correto	Correto	Correto	Errado	Correto
Não sei dizer	Não sei dizer	Não sei dizer	Correto	Correto
Correto	Correto	Correto	Correto	Correto
Não sei dizer	Não sei dizer	Não sei dizer	Não sei dizer	Correto
Correto	Correto	Não sei dizer	Correto	Correto
Correto	Correto	Não sei dizer	Correto	Correto
Não sei dizer	Não sei dizer	Não sei dizer	Não sei dizer	Não sei dizer
Correto	Correto	Correto	Correto	Correto
Não sei dizer	Não sei dizer	Não sei dizer	Não sei dizer	Não sei dizer
Não sei dizer	Não sei dizer	Não sei dizer	Não sei dizer	Correto
Não sei dizer	Não sei dizer	Não sei dizer	Correto	Correto
Correto	Não sei dizer	Não sei dizer	Não sei dizer	Correto
Correto	Correto	Não sei dizer	Correto	Correto
Correto	Correto	Correto	Correto	Correto
Correto	Correto	Não sei dizer	Correto	Correto
Não sei dizer	Correto	Não sei dizer	Correto	Correto
Não sei dizer	Não sei dizer	Não sei dizer	Não sei dizer	Não sei dizer
Correto	Correto	Não sei dizer	Não sei dizer	Correto
Correto	Correto	Não sei dizer	Não sei dizer	Correto
Não sei dizer	Correto	Não sei dizer	Não sei dizer	Correto
Não sei dizer	Correto	Não sei dizer	Correto	Correto
Não sei dizer	Não sei dizer	Não sei dizer	Não sei dizer	Não sei dizer
Correto	Correto	Não sei dizer	Correto	Correto
Correto	Correto	Correto	Não sei dizer	Correto
Correto	Correto	Não sei dizer	Correto	Correto
Não sei dizer	Não sei dizer	Não sei dizer	Não sei dizer	Não sei dizer
Correto	Correto	Não sei dizer	Correto	Não sei dizer
Correto	Correto	Correto	Correto	Correto
Correto	Correto	Correto	Correto	Correto
Não sei dizer	Não sei dizer	Não sei dizer	Não sei dizer	Não sei dizer
Não sei dizer	Correto	Correto	Não sei dizer	Correto
Correto	Correto	Não sei dizer	Correto	Correto
Correto	Correto	Correto	Correto	Correto
Não sei dizer	Correto	Não sei dizer	Correto	Correto
Não sei dizer	Correto	Correto	Correto	Correto
Correto	Errado	Correto	Errado	Correto
Correto	Correto	Correto	Correto	Correto
Não sei dizer	Não sei dizer	Não sei dizer	Não sei dizer	Não sei dizer
Não sei dizer	Não sei dizer	Não sei dizer	Não sei dizer	Não sei dizer
Correto	Correto	Não sei dizer	Não sei dizer	Correto
Não sei dizer	Não sei dizer	Não sei dizer	Não sei dizer	Não sei dizer
Não sei dizer	Não sei dizer	Não sei dizer	Não sei dizer	Correto
Correto	Correto	Não sei dizer	Correto	Correto
Correto	Correto	Errado	Correto	Correto
Correto	Correto	Correto	Correto	Correto
Correto	Correto	Não sei dizer	Correto	Correto
Não sei dizer	Não sei dizer	Não sei dizer	Não sei dizer	Não sei dizer
Correto	Não sei dizer	Não sei dizer	Correto	Não sei dizer
Correto	Correto	Correto	Correto	Correto
Não sei dizer	Não sei dizer	Não sei dizer	Não sei dizer	Não sei dizer
Correto	Correto	Não sei dizer	Correto	Correto
Não sei dizer	Não sei dizer	Não sei dizer	Não sei dizer	Não sei dizer
Não sei dizer	Não sei dizer	Não sei dizer	Não sei dizer	Não sei dizer

Não sei dizer	Não sei dizer	Não sei dizer	Não sei dizer	Não sei dizer
Correto	Correto	Não sei dizer	Correto	Correto
Correto	Correto	Correto	Correto	Correto
Não sei dizer	Não sei dizer	Não sei dizer	Não sei dizer	Não sei dizer
Não sei dizer	Não sei dizer	Não sei dizer	Correto	Correto
Correto	Correto	Correto	Correto	Correto
Não sei dizer	Não sei dizer	Não sei dizer	Não sei dizer	Não sei dizer
Não sei dizer	Não sei dizer	Não sei dizer	Correto	Não sei dizer
Correto	Correto	Correto	Correto	Correto
Correto	Correto	Não sei dizer	Correto	Correto
Correto	Correto	Não sei dizer	Correto	Correto
Correto	Correto	Não sei dizer	Correto	Correto
Não sei dizer	Errado	Correto	Correto	Errado
Não sei dizer	Não sei dizer	Não sei dizer	Correto	Correto
Correto	Correto	Não sei dizer	Correto	Correto
Não sei dizer	Não sei dizer	Não sei dizer	Correto	Não sei dizer
Errado	Correto	Correto	Correto	Correto
Correto	Correto	Não sei dizer	Correto	Correto
Correto	Correto	Errado	Correto	Correto
Não sei dizer	Correto	Não sei dizer	Correto	Correto
Correto	Correto	Não sei dizer	Não sei dizer	Correto
Correto	Correto	Errado	Correto	Correto
Correto	Correto	Correto	Correto	Correto
Correto	Correto	Correto	Não sei dizer	Correto
Não sei dizer	Não sei dizer	Não sei dizer	Não sei dizer	Não sei dizer
Correto	Correto	Não sei dizer	Não sei dizer	Correto
Não sei dizer	Não sei dizer	Não sei dizer	Não sei dizer	Não sei dizer
Não sei dizer	Não sei dizer	Não sei dizer	Não sei dizer	Não sei dizer
Não sei dizer	Não sei dizer	Não sei dizer	Não sei dizer	Não sei dizer
Correto	Correto	Correto	Correto	Correto
Correto	Correto	Não sei dizer	Correto	Correto
Não sei dizer	Não sei dizer	Não sei dizer	Não sei dizer	Não sei dizer
Não sei dizer	Não sei dizer	Não sei dizer	Correto	Não sei dizer
Errado	Correto	Não sei dizer	Correto	Correto
Correto	Correto	Correto	Correto	Correto
Não sei dizer	Correto	Correto	Correto	Correto
Correto	Correto	Correto	Correto	Correto
Não sei dizer	Não sei dizer	Não sei dizer	Correto	Correto
Correto	Correto	Correto	Correto	Correto
Correto	Correto	Não sei dizer	Não sei dizer	Não sei dizer
Correto	Correto	Correto	Correto	Correto
Correto	Correto	Não sei dizer	Correto	Correto
Não sei dizer	Não sei dizer	Não sei dizer	Não sei dizer	Não sei dizer
Correto	Correto	Não sei dizer	Correto	Correto
Correto	Correto	Correto	Correto	Correto
Não sei dizer	Correto	Não sei dizer	Não sei dizer	Correto
Correto	Correto	Correto	Correto	Correto
Não sei dizer	Não sei dizer	Não sei dizer	Não sei dizer	Não sei dizer
Correto	Correto	Correto	Correto	Correto
Correto	Correto	Correto	Correto	Correto
Correto	Correto	Correto	Correto	Correto
Não sei dizer	Não sei dizer	Não sei dizer	Não sei dizer	Não sei dizer
Correto	Correto	Correto	Correto	Correto
Correto	Correto	Não sei dizer	Correto	Correto

Não sei dizer	Não sei dizer	Não sei dizer	Não sei dizer	Não sei dizer
Correto	Correto	Correto	Correto	Correto
Correto	Correto	Correto	Correto	Correto
Correto	Correto	Correto	Não sei dizer	Correto
Correto	Correto	Não sei dizer	Correto	Correto
Correto	Correto	Não sei dizer	Errado	Correto
Correto	Correto	Não sei dizer	Correto	Correto
Correto	Correto	Não sei dizer	Correto	Correto
Correto	Correto	Correto	Correto	Correto
Não sei dizer	Não sei dizer	Não sei dizer	Não sei dizer	Não sei dizer
Correto	Correto	Correto	Correto	Não sei dizer
Correto	Correto	Correto	Correto	Correto
Correto	Correto	Não sei dizer	Correto	Correto
Correto	Correto	Correto	Correto	Correto
Correto	Correto	Não sei dizer	Correto	Correto
Correto	Correto	Correto	Correto	Correto
Não sei dizer	Não sei dizer	Não sei dizer	Correto	Correto
Correto	Correto	Correto	Correto	Correto
Correto	Correto	Correto	Não sei dizer	Correto
Não sei dizer	Correto	Não sei dizer	Correto	Correto
Não sei dizer	Não sei dizer	Não sei dizer	Não sei dizer	Não sei dizer
Correto	Correto	Correto	Correto	Correto
Correto	Correto	Correto	Correto	Correto
Correto	Correto	Correto	Correto	Correto
Correto	Correto	Não sei dizer	Não sei dizer	Não sei dizer
Correto	Correto	Correto	Correto	Correto
Não sei dizer	Correto	Não sei dizer	Correto	Correto
Não sei dizer	Não sei dizer	Não sei dizer	Não sei dizer	Não sei dizer
Não sei dizer	Correto	Não sei dizer	Correto	Correto
Correto	Correto	Correto	Não sei dizer	Correto
Correto	Correto	Correto	Correto	Correto
Não sei dizer	Não sei dizer	Não sei dizer	Não sei dizer	Não sei dizer
Correto	Correto	Correto	Correto	Correto
Correto	Correto	Correto	Correto	Correto
Não sei dizer	Não sei dizer	Não sei dizer	Não sei dizer	Correto
Correto	Não sei dizer	Correto	Correto	Correto
Correto	Correto	Correto	Correto	Correto
Não sei dizer	Não sei dizer	Não sei dizer	Correto	Correto
Correto	Correto	Não sei dizer	Não sei dizer	Correto
Correto	Correto	Não sei dizer	Correto	Correto
Não sei dizer	Errado	Não sei dizer	Correto	Correto
Correto	Correto	Correto	Correto	Correto
Correto	Correto	Correto	Correto	Correto
Não sei dizer	Não sei dizer	Não sei dizer	Correto	Correto
Correto	Correto	Não sei dizer	Correto	Correto
Não sei dizer	Correto	Não sei dizer	Não sei dizer	Correto
Correto	Correto	Errado	Correto	Correto
Correto	Correto	Correto	Correto	Correto
Correto	Correto	Não sei dizer	Correto	Não sei dizer
Correto	Correto	Não sei dizer	Não sei dizer	Correto
Não sei dizer	Correto	Não sei dizer	Não sei dizer	Correto
Correto	Correto	Não sei dizer	Não sei dizer	Correto
Não sei dizer	Não sei dizer	Não sei dizer	Não sei dizer	Não sei dizer
Correto	Correto	Não sei dizer	Correto	Correto

Correto	Não sei dizer	Não sei dizer	Correto	Correto
Não sei dizer	Não sei dizer	Não sei dizer	Não sei dizer	Não sei dizer
Correto	Correto	Não sei dizer	Correto	Correto
Correto	Correto	Não sei dizer	Correto	Correto
Correto	Correto	Não sei dizer	Correto	Correto
Não sei dizer	Correto	Não sei dizer	Não sei dizer	Correto
Não sei dizer	Correto	Não sei dizer	Correto	Correto
Não sei dizer	Não sei dizer	Não sei dizer	Correto	Correto
Correto	Correto	Não sei dizer	Não sei dizer	Correto
Correto	Correto	Não sei dizer	Não sei dizer	Correto
Não sei dizer	Correto	Não sei dizer	Correto	Correto
Não sei dizer	Não sei dizer	Não sei dizer	Não sei dizer	Não sei dizer
Não sei dizer	Não sei dizer	Não sei dizer	Não sei dizer	Não sei dizer
Correto	Correto	Não sei dizer	Correto	Correto
Não sei dizer	Não sei dizer	Não sei dizer	Não sei dizer	Não sei dizer
Correto	Não sei dizer	Não sei dizer	Não sei dizer	Não sei dizer
Correto	Correto	Correto	Correto	Não sei dizer
Correto	Correto	Correto	Correto	Correto
Correto	Correto	Não sei dizer	Não sei dizer	Não sei dizer
Não sei dizer	Correto	Não sei dizer	Não sei dizer	Não sei dizer
Não sei dizer	Não sei dizer	Não sei dizer	Não sei dizer	Não sei dizer
Correto	Correto	Não sei dizer	Correto	Correto
Correto	Correto	Correto	Correto	Correto
Não sei dizer	Não sei dizer	Não sei dizer	Não sei dizer	Não sei dizer
Não sei dizer	Não sei dizer	Não sei dizer	Correto	Correto
Não sei dizer	Não sei dizer	Não sei dizer	Não sei dizer	Não sei dizer
Correto	Correto	Não sei dizer	Correto	Correto
Correto	Correto	Não sei dizer	Correto	Não sei dizer
Não sei dizer	Não sei dizer	Não sei dizer	Não sei dizer	Não sei dizer
Não sei dizer	Correto	Não sei dizer	Correto	Correto
Correto	Correto	Correto	Correto	Correto
Correto	Correto	Não sei dizer	Não sei dizer	Não sei dizer
Correto	Correto	Correto	Errado	Correto
Correto	Correto	Não sei dizer	Não sei dizer	Correto
Correto	Correto	Não sei dizer	Correto	Correto
Não sei dizer	Não sei dizer	Não sei dizer	Não sei dizer	Não sei dizer
Correto	Correto	Correto	Correto	Correto
Correto	Correto	Não sei dizer	Correto	Não sei dizer
Correto	Correto	Correto	Não sei dizer	Não sei dizer
Correto	Correto	Correto	Correto	Correto
Correto	Correto	Não sei dizer	Correto	Correto
Correto	Correto	Correto	Correto	Correto
Não sei dizer	Não sei dizer	Não sei dizer	Correto	Correto
Correto	Correto	Correto	Correto	Correto
Correto	Correto	Não sei dizer	Não sei dizer	Correto
Errado	Correto	Correto	Correto	Correto
Correto	Correto	Não sei dizer	Correto	Correto
Não sei dizer	Correto	Correto	Correto	Correto
Não sei dizer	Não sei dizer	Não sei dizer	Não sei dizer	Não sei dizer
Não sei dizer	Não sei dizer	Não sei dizer	Não sei dizer	Não sei dizer
Correto	Correto	Não sei dizer	Não sei dizer	Correto
Correto	Correto	Não sei dizer	Não sei dizer	Correto
Correto	Correto	Correto	Correto	Correto
Não sei dizer	Correto	Não sei dizer	Correto	Correto

Correto	Correto	Correto	Não sei dizer	Correto
Correto	Correto	Correto	Errado	Correto
Não sei dizer	Não sei dizer	Não sei dizer	Não sei dizer	Não sei dizer
Correto	Correto	Correto	Correto	Correto
Não sei dizer	Não sei dizer	Não sei dizer	Correto	Correto
Correto	Correto	Correto	Correto	Correto
Não sei dizer	Não sei dizer	Não sei dizer	Não sei dizer	Não sei dizer
Correto	Correto	Correto	Não sei dizer	Correto
Não sei dizer	Não sei dizer	Não sei dizer	Não sei dizer	Não sei dizer
Não sei dizer	Não sei dizer	Não sei dizer	Não sei dizer	Não sei dizer
Correto	Correto	Correto	Correto	Correto
Correto	Correto	Correto	Correto	Correto
Correto	Correto	Não sei dizer	Correto	Correto
Não sei dizer	Correto	Não sei dizer	Correto	Não sei dizer
Errado	Correto	Correto	Correto	Correto
Correto	Correto	Correto	Não sei dizer	Não sei dizer
Correto	Correto	Correto	Não sei dizer	Não sei dizer
Correto	Correto	Não sei dizer	Correto	Correto
Correto	Correto	Não sei dizer	Correto	Correto
Correto	Correto	Não sei dizer	Não sei dizer	Não sei dizer
Não sei dizer	Correto	Não sei dizer	Não sei dizer	Correto
Correto	Correto	Correto	Correto	Correto
Não sei dizer	Correto	Não sei dizer	Não sei dizer	Correto
Correto	Correto	Não sei dizer	Não sei dizer	Não sei dizer
Não sei dizer	Não sei dizer	Não sei dizer	Não sei dizer	Não sei dizer
Não sei dizer	Não sei dizer	Não sei dizer	Não sei dizer	Correto
Correto	Correto	Não sei dizer	Correto	Correto
Não sei dizer	Não sei dizer	Não sei dizer	Não sei dizer	Correto
Correto	Correto	Correto	Correto	Correto
Não sei dizer	Não sei dizer	Não sei dizer	Não sei dizer	Não sei dizer
Não sei dizer	Não sei dizer	Não sei dizer	Não sei dizer	Não sei dizer
Errado	Correto	Não sei dizer	Correto	Correto
Não sei dizer	Não sei dizer	Não sei dizer	Não sei dizer	Não sei dizer
Não sei dizer	Não sei dizer	Não sei dizer	Não sei dizer	Não sei dizer
Não sei dizer	Não sei dizer	Não sei dizer	Não sei dizer	Não sei dizer
Não sei dizer	Correto	Correto	Não sei dizer	Errado
Correto	Correto	Correto	Correto	Correto
Não sei dizer	Não sei dizer	Não sei dizer	Não sei dizer	Não sei dizer
Não sei dizer	Não sei dizer	Não sei dizer	Não sei dizer	Não sei dizer
Correto	Correto	Não sei dizer	Correto	Correto
Correto	Correto	Correto	Correto	Correto
Não sei dizer	Não sei dizer	Não sei dizer	Não sei dizer	Não sei dizer
Não sei dizer	Não sei dizer	Não sei dizer	Não sei dizer	Não sei dizer
Não sei dizer	Não sei dizer	Não sei dizer	Não sei dizer	Não sei dizer
Correto	Correto	Não sei dizer	Correto	Não sei dizer
Não sei dizer	Correto	Não sei dizer	Não sei dizer	Correto
Correto	Correto	Não sei dizer	Não sei dizer	Correto
Não sei dizer	Não sei dizer	Não sei dizer	Não sei dizer	Não sei dizer
Correto	Correto	Correto	Correto	Correto
Não sei dizer	Não sei dizer	Não sei dizer	Não sei dizer	Não sei dizer
Correto	Correto	Não sei dizer	Correto	Não sei dizer
Correto	Correto	Correto	Correto	Correto
Não sei dizer	Não sei dizer	Não sei dizer	Não sei dizer	Não sei dizer
Correto	Correto	Não sei dizer	Não sei dizer	Não sei dizer

Correto	Correto	Não sei dizer	Não sei dizer	Correto
Correto	Correto	Correto	Não sei dizer	Correto
Correto	Correto	Não sei dizer	Correto	Correto
Não sei dizer	Não sei dizer	Não sei dizer	Não sei dizer	Não sei dizer
Correto	Correto	Correto	Correto	Correto
Correto	Correto	Correto	Correto	Correto
Correto	Correto	Não sei dizer	Correto	Correto
Correto	Correto	Correto	Correto	Correto
Correto	Correto	Não sei dizer	Correto	Correto
Correto	Correto	Correto	Correto	Correto
Correto	Correto	Correto	Correto	Correto
Não sei dizer	Não sei dizer	Não sei dizer	Não sei dizer	Não sei dizer
Correto	Correto	Correto	Correto	Correto
Correto	Correto	Correto	Correto	Correto
Correto	Correto	Correto	Correto	Correto
Correto	Correto	Correto	Correto	Correto
Correto	Correto	Correto	Correto	Correto
Correto	Correto	Correto	Não sei dizer	Correto
Não sei dizer	Não sei dizer	Não sei dizer	Não sei dizer	Não sei dizer
Correto	Correto	Correto	Correto	Correto
Correto	Correto	Não sei dizer	Correto	Correto
Não sei dizer	Não sei dizer	Não sei dizer	Não sei dizer	Não sei dizer
Correto	Correto	Correto	Correto	Correto
Não sei dizer	Não sei dizer	Não sei dizer	Não sei dizer	Não sei dizer
Não sei dizer	Não sei dizer	Não sei dizer	Não sei dizer	Não sei dizer
Correto	Correto	Correto	Correto	Correto
Correto	Correto	Errado	Correto	Correto
Não sei dizer	Não sei dizer	Não sei dizer	Não sei dizer	Não sei dizer
Correto	Correto	Correto	Correto	Correto
Correto	Correto	Correto	Correto	Correto
Correto	Correto	Não sei dizer	Correto	Correto
Não sei dizer	Não sei dizer	Não sei dizer	Não sei dizer	Não sei dizer
Correto	Correto	Não sei dizer	Correto	Correto
Correto	Correto	Correto	Correto	Correto
Correto	Correto	Correto	Não sei dizer	Correto
Correto	Correto	Correto	Correto	Correto
Não sei dizer	Não sei dizer	Não sei dizer	Não sei dizer	Não sei dizer
Errado	Correto	Não sei dizer	Errado	Correto
Correto	Correto	Correto	Não sei dizer	Correto
Correto	Correto	Correto	Correto	Correto
Correto	Correto	Correto	Correto	Correto
Correto	Correto	Correto	Não sei dizer	Correto
Correto	Correto	Correto	Correto	Correto
Não sei dizer	Não sei dizer	Não sei dizer	Não sei dizer	Não sei dizer
Não sei dizer	Não sei dizer	Não sei dizer	Não sei dizer	Não sei dizer
Não sei dizer	Não sei dizer	Não sei dizer	Não sei dizer	Não sei dizer
Correto	Correto	Não sei dizer	Não sei dizer	Correto
Não sei dizer	Não sei dizer	Não sei dizer	Correto	Correto
Não sei dizer	Não sei dizer	Não sei dizer	Não sei dizer	Não sei dizer
Correto	Correto	Correto	Correto	Correto
Não sei dizer	Não sei dizer	Não sei dizer	Não sei dizer	Não sei dizer
Não sei dizer	Não sei dizer	Não sei dizer	Não sei dizer	Não sei dizer
Não sei dizer	Não sei dizer	Não sei dizer	Não sei dizer	Não sei dizer
Não sei dizer	Não sei dizer	Não sei dizer	Não sei dizer	Não sei dizer

Não sei dizer	Não sei dizer	Não sei dizer	Não sei dizer	Correto
Não sei dizer	Não sei dizer	Não sei dizer	Não sei dizer	Não sei dizer
Não sei dizer	Não sei dizer	Não sei dizer	Não sei dizer	Não sei dizer
Correto	Correto	Não sei dizer	Não sei dizer	Correto
Não sei dizer	Não sei dizer	Não sei dizer	Não sei dizer	Não sei dizer
Correto	Correto	Não sei dizer	Não sei dizer	Correto
Não sei dizer	Não sei dizer	Não sei dizer	Não sei dizer	Não sei dizer
Não sei dizer	Correto	Não sei dizer	Não sei dizer	Correto
Correto	Correto	Não sei dizer	Não sei dizer	Correto
Não sei dizer	Não sei dizer	Não sei dizer	Não sei dizer	Não sei dizer
Correto	Correto	Correto	Correto	Correto
Não sei dizer	Não sei dizer	Não sei dizer	Não sei dizer	Não sei dizer
Não sei dizer	Não sei dizer	Não sei dizer	Não sei dizer	Não sei dizer
Não sei dizer	Não sei dizer	Não sei dizer	Não sei dizer	Não sei dizer
Correto	Correto	Correto	Correto	Correto
Não sei dizer	Não sei dizer	Não sei dizer	Não sei dizer	Não sei dizer
Correto	Não sei dizer	Não sei dizer	Não sei dizer	Não sei dizer
Correto	Correto	Não sei dizer	Correto	Correto

Q16
Não sei dizer
Não sei dizer
Não sei dizer
Não sei dizer
Não sei dizer
Não sei dizer
Errado
Não sei dizer
Não sei dizer
Não sei dizer
Correto
Correto
Não sei dizer
Não sei dizer
Correto
Não sei dizer
Correto
Não sei dizer
Correto
Correto
Não sei dizer
Não sei dizer
Não sei dizer
Correto
Correto
Não sei dizer
Não sei dizer
Não sei dizer
Não sei dizer
Correto
Não sei dizer
Não sei dizer
Correto
Não sei dizer
Correto
Não sei dizer
Correto
Correto
Não sei dizer
Não sei dizer
Correto
Não sei dizer
Não sei dizer
Correto
Não sei dizer
Errado
Não sei dizer
Não sei dizer
Correto
Não sei dizer
Não sei dizer
Não sei dizer
Não sei dizer

Correto
Não sei dizer
Não sei dizer
Não sei dizer
Não sei dizer
Correto
Não sei dizer
Não sei dizer
Correto
Correto
Não sei dizer
Correto
Não sei dizer
Correto
Não sei dizer
Correto
Correto
Não sei dizer
Não sei dizer
Não sei dizer
Correto
Correto
Não sei dizer
Não sei dizer
Não sei dizer
Correto
Correto
Não sei dizer
Não sei dizer
Correto
Não sei dizer
Não sei dizer
Não sei dizer
Não sei dizer
Não sei dizer
Correto
Não sei dizer
Não sei dizer
Não sei dizer
Não sei dizer
Não sei dizer
Não sei dizer
Não sei dizer
Não sei dizer
Não sei dizer
Não sei dizer
Correto
Não sei dizer
Correto
Não sei dizer
Não sei dizer
Não sei dizer
Não sei dizer
Não sei dizer

Correto
Não sei dizer
Não sei dizer
Não sei dizer
Não sei dizer
Correto
Correto
Não sei dizer
Não sei dizer
Não sei dizer
Não sei dizer
Correto
Não sei dizer
Correto
Não sei dizer
Não sei dizer
Correto
Não sei dizer
Correto
Correto
Não sei dizer
Não sei dizer
Correto
Não sei dizer
Não sei dizer
Não sei dizer
Não sei dizer
Não sei dizer
Correto
Não sei dizer
Não sei dizer
Não sei dizer
Não sei dizer
Não sei dizer
Não sei dizer
Não sei dizer
Não sei dizer
Não sei dizer
Não sei dizer
Não sei dizer
Não sei dizer
Correto
Não sei dizer
Não sei dizer
Não sei dizer
Não sei dizer
Não sei dizer
Errado
Não sei dizer
Não sei dizer
Correto
Não sei dizer
Não sei dizer
Correto

Não sei dizer
Errado
Não sei dizer
Correto
Não sei dizer
Não sei dizer
Não sei dizer
Não sei dizer
Correto
Não sei dizer
Não sei dizer
Correto
Não sei dizer
Não sei dizer
Não sei dizer
Não sei dizer
Não sei dizer
Não sei dizer
Não sei dizer
Não sei dizer
Não sei dizer
Correto
Não sei dizer
Não sei dizer
Correto
Correto
Não sei dizer
Não sei dizer
Correto
Não sei dizer
Não sei dizer
Não sei dizer
Não sei dizer
Correto
Não sei dizer
Não sei dizer
Correto
Correto
Não sei dizer
Não sei dizer
Não sei dizer
Não sei dizer
Não sei dizer
Não sei dizer
Não sei dizer
Correto
Não sei dizer
Não sei dizer
Não sei dizer
Correto
Não sei dizer
Não sei dizer
Não sei dizer
Não sei dizer

Não sei dizer
Correto
Correto
Não sei dizer
Não sei dizer
Correto
Não sei dizer
Não sei dizer
Correto
Não sei dizer
Correto
Correto
Não sei dizer
Não sei dizer
Correto
Não sei dizer
Não sei dizer
Correto
Correto
Não sei dizer
Correto
Correto
Correto
Correto
Não sei dizer
Não sei dizer
Não sei dizer
Não sei dizer
Não sei dizer
Correto
Correto
Não sei dizer
Não sei dizer
Correto
Correto
Não sei dizer
Correto
Não sei dizer
Correto
Não sei dizer
Correto
Correto
Não sei dizer
Não sei dizer
Correto
Não sei dizer
Correto
Não sei dizer
Correto
Correto
Correto
Não sei dizer
Correto
Não sei dizer

Não sei dizer
Correto
Correto
Não sei dizer
Correto
Não sei dizer
Não sei dizer
Não sei dizer
Correto
Não sei dizer
Correto
Correto
Correto
Correto
Não sei dizer
Não sei dizer
Não sei dizer
Correto
Correto
Não sei dizer
Não sei dizer
Correto
Correto
Correto
Não sei dizer
Correto
Não sei dizer
Não sei dizer
Não sei dizer
Não sei dizer
Correto
Não sei dizer
Não sei dizer
Correto
Não sei dizer
Correto
Correto
Correto
Correto
Correto
Correto
Correto
Correto
Não sei dizer
Não sei dizer
Não sei dizer
Correto
Correto
Não sei dizer
Correto
Não sei dizer
Não sei dizer
Não sei dizer
Não sei dizer

Não sei dizer
Não sei dizer
Não sei dizer
Não sei dizer
Correto
Não sei dizer
Não sei dizer
Não sei dizer
Não sei dizer
Não sei dizer
Não sei dizer
Não sei dizer
Não sei dizer
Não sei dizer
Não sei dizer
Não sei dizer
Não sei dizer
Correto
Não sei dizer
Não sei dizer
Não sei dizer
Não sei dizer
Não sei dizer
Não sei dizer
Não sei dizer
Não sei dizer
Não sei dizer
Não sei dizer
Não sei dizer
Não sei dizer
Correto
Não sei dizer
Correto
Não sei dizer
Não sei dizer
Não sei dizer
Correto
Não sei dizer
Correto
Correto
Correto
Não sei dizer
Não sei dizer
Não sei dizer
Correto
Não sei dizer
Errado
Correto
Não sei dizer
Não sei dizer
Não sei dizer
Não sei dizer
Não sei dizer
Correto

Não sei dizer
Correto
Não sei dizer
Correto
Não sei dizer
Correto
Não sei dizer
Não sei dizer
Não sei dizer
Não sei dizer
Correto
Correto
Não sei dizer
Não sei dizer
Correto
Correto
Não sei dizer
Não sei dizer
Não sei dizer
Não sei dizer
Não sei dizer
Correto
Não sei dizer
Não sei dizer
Não sei dizer
Não sei dizer
Correto
Não sei dizer
Não sei dizer
Não sei dizer
Não sei dizer
Não sei dizer
Não sei dizer
Não sei dizer
Não sei dizer
Correto
Correto
Não sei dizer
Não sei dizer
Correto
Correto
Não sei dizer
Não sei dizer
Não sei dizer
Não sei dizer
Não sei dizer
Correto
Não sei dizer
Correto
Não sei dizer
Não sei dizer
Correto
Não sei dizer
Não sei dizer

Correto
Correto
Não sei dizer
Não sei dizer
Correto
Correto
Não sei dizer
Não sei dizer
Correto
Correto
Correto
Não sei dizer
Errado
Correto
Correto
Correto
Correto
Correto
Não sei dizer
Correto
Não sei dizer
Não sei dizer
Correto
Não sei dizer
Não sei dizer
Errado
Errado
Não sei dizer
Correto
Correto
Correto
Não sei dizer
Correto
Correto
Correto
Correto
Não sei dizer
Não sei dizer
Correto
Correto
Correto
Não sei dizer
Errado
Não sei dizer
Não sei dizer
Não sei dizer
Não sei dizer
Correto
Não sei dizer
Correto
Não sei dizer
Não sei dizer
Não sei dizer
Não sei dizer

Não sei dizer
Não sei dizer
Não sei dizer
Correto
Não sei dizer
Não sei dizer
Não sei dizer
Não sei dizer
Correto
Não sei dizer
Correto
Não sei dizer
Não sei dizer
Não sei dizer
Correto
Não sei dizer
Não sei dizer
Não sei dizer

Q1		Q2		Q3		Q4		Q5	
Correto	458	Correto	338	Correto	492	Correto	274	Correto	254
Errado	31	Errado	109	Errado	5	Errado	102	Errado	14
Não sei	14	Não sei	56	Não sei	6	Não sei	127	Não sei dize	235
Correto	91.05	Correto	67.20	Correto	97.81	Correto	54.47	Correto	50.50
Errado	6.16	Errado	21.67	Errado	0.99	Errado	20.28	Errado	2.78
Não sei	2.78	Não sei	11.13	Não sei	1.19	Não sei	25.25	Não sei	46.72

Q6		Q7		Q8		Q9		Q10	
Correto	166	Correto	129	Correto	218	Correto	257	Correto	306
Errado	32	Errado	34	Errado	25	Errado	7	Errado	26
Não sei dize	305	Não sei dize	340	Não sei dize	260	Não sei dize	239	Não sei dize	171
Correto	33.00	Correto	25.65	Correto	43.34	Correto	51.09	Correto	60.83
Errado	6.36	Errado	6.76	Errado	4.97	Errado	1.39	Errado	5.17
Não sei	60.64	Não sei	67.59	Não sei	51.69	Não sei	47.51	Não sei	34.00

Q11		Q12		Q13		Q14		Q15	
Correto	289	Correto	333	Correto	166	Correto	273	Correto	338
Errado	13	Errado	3	Errado	6	Errado	7	Errado	3
Não sei dize	201	Não sei dize	167	Não sei dize	331	Não sei dize	223	Não sei dize	162
Correto	57.46	Correto	66.20	Correto	33.00	Correto	54.27	Correto	67.20
Errado	2.58	Errado	0.60	Errado	1.19	Errado	1.39	Errado	0.60
Não sei	39.96	Não sei	33.20	Não sei	65.81	Não sei	44.33	Não sei	32.21

Q16	
Correto	168
Errado	9
Não sei dize	326
Correto	33.40
Errado	1.79
Não sei	64.81
