# Supplementary material for: Factors associated with university students’ knowledge about HIV and pre- and post-exposure prophylaxis
Source: Rev Bras Enferm. 2024 Aug 30;77(Suppl 2):e20240092. doi: 10.1590/0034-7167-2024-0092 (PMC11370771; doi:10.1590/0034-7167-2024-0092)
Supplement: Translation

# Translation: Fatores associados ao conhecimento de universitários acerca do HIV e das profilaxias pré e pós-exposição

**DOI:** 10.1590/0034-7167-2024-0092pt

**Authors:** Ana Luísa Serrano Lima, Heitor Hortensi Sesnik, Lucas Vinícius de Lima, Gabriel Pavinati, Maria de Fátima Garcia Lopes Merino, Marcelle Paiano, Nelly Lopes de Moraes Gil, Gabriela Tavares Magnabosco

**Affiliations:** I Universidade Estadual de Maringá. Maringá, Paraná, Brasil

**Keywords:** HIV, Conhecimento, Profilaxia Pré-Exposição, Profilaxia Pós-Exposição, Estudantes

## Abstract

**Objetivos:**

analisar os fatores associados ao conhecimento de universitários sobre o HIV e as profilaxias pré e pós-exposição.

**Métodos:**

estudo transversal realizado com 503 universitários de um estado do sul do Brasil, cujos dados foram coletados por meio de um instrumento de caracterização e um questionário contendo 16 afirmativas sobre o tema; na análise, utilizaram-se medidas descritivas e modelos de regressão de Poisson com variância robusta.

**Resultados:**

a prevalência de conhecimento adequado (ou seja, mais de 12 acertos) foi de 27,83%; os universitários com idade superior a 24 anos, dos cursos da área de saúde, que não tiveram relações sexuais no último trimestre, com histórico de teste rápido para HIV e que sabiam ou já tinham ouvido falar sobre as profilaxias apresentaram maior probabilidade de obter mais de 12 acertos.

**Conclusões:**

verificou-se que, de modo geral, o conhecimento dos jovens sobre o HIV e suas profilaxias foi inadequado e influenciado por fatores sociodemográficos, educacionais e comportamentais.

## INTRODUÇÃO

A infecção pelo vírus da imunodeficiência humana (HIV, *human immunodeficiency virus*) constitui um grave problema de saúde pública, com impacto social, econômico e epidemiológico, em indivíduos e coletivos, ao redor do globo. A Organização Pan-Americana da Saúde (OPAS) estimou que existiam, no mundo, 38 milhões de pessoas vivendo com HIV e que houve um aumento de 21% na incidência na região da América Latina em 2019, em comparação aos dados de 2010^(1)^.

No Brasil, em 2021, foram notificados mais de 40 mil novos diagnósticos de HIV; destes, 39% foram em pessoas de 20 a 29 anos^(2)^. Além disso, a infecção acometeu de forma desproporcional as pessoas do sexo masculino, com uma razão de 2,8 novos casos de HIV entre homens para cada mulher^(2)^. Dessa forma, percebem-se indícios do estabelecimento de grupos mais vulneráveis à infecção no país, que devem figurar, portanto, como prioridade para as políticas públicas.

A iniciação sexual precoce, a adoção de práticas sexuais desprotegidas e com múltiplas parcerias, e a baixa percepção de risco entre esse público são alguns dos fatores que conferem maior suscetibilidade à infecção pelo HIV^(3-4)^. Para mais, quando acometidas, as pessoas jovens com HIV se inserem em contextos de vulnerabilidade dinâmicos e subjetivos, vivenciando situações de estigma e discriminação, negação do diagnóstico e baixa adesão à terapia antirretroviral (TARV)^(5)^.

No rol das ações de enfrentamento ao HIV no decorrer dos anos, notaram-se avanços globais nas políticas de prevenção, diagnóstico e tratamento. Em 2010, o Programa das Nações Unidas sobre HIV/aids (UNAIDS) sugeriu que as estratégias deveriam se pautar na prevenção combinada do HIV, definida como um conjunto de intervenções biomédicas, comportamentais e estruturais para atender as necessidades de pessoas e comunidades, visando à redução de novas infecções^(6)^.

No Brasil, em 2017, o Ministério da Saúde propôs bases para a implementação da prevenção combinada no Sistema Único de Saúde (SUS), enfatizando grupos prioritários e populações-chave, dentre os quais se destacaram os homens gays, os homens que fazem sexo com homens (HSH) e a população jovem^(7)^. Com isso, almejava-se o fortalecimento na resposta ao HIV para alcançar as metas de eliminação até 2030, pactuadas nos Objetivos do Desenvolvimento Sustentável (ODS).

No âmbito da prevenção combinada do HIV, a profilaxia pré-exposição (PrEP) e a profilaxia pós-exposição (PEP) - classificadas como intervenções biomédicas - se mostram como potenciais ferramentas para o controle da infecção, sobretudo quando aliadas a ações comportamentais e estruturais^(7)^. A PrEP e a PEP estão disponíveis gratuitamente no SUS e consistem, respectivamente, no uso de antirretrovirais antes e após a exposição ao HIV como forma de prevenção^(7)^.

Para que as profilaxias sejam implementadas assertiva e efetivamente, os usuários devem estar informados sobre as estratégias para a escolha daquelas que mais se adequam às suas singularidades e necessidades em um dado momento^(8)^. No entanto, pesquisas recentes indicam que existe falta de divulgação e recomendação da PrEP e da PEP, e sugerem que o conhecimento dos jovens sobre as profilaxias é, por vezes, frágil e errôneo, dificultando a expansão do uso desses métodos^(9-11)^.

Nesse contexto, sabe-se que as escolas e universidades têm um papel crucial na disseminação de informações sobre as infecções sexualmente transmissíveis (IST) e suas medidas preventivas entre os estudantes^(9-10)^. Por meio de programas educacionais, campanhas de conscientização e serviços de saúde estudantil, esses ambientes podem apoiar o conhecimento sobre a prevenção, a detecção e o tratamento das IST, promovendo a saúde entre a comunidade estudantil.

É, portanto, indiscutível a necessidade de identificar os saberes apresentados por estudantes, visto que a (não) adoção de práticas preventivas depende disso. Nesse segmento, presume-se que esta pesquisa pode contribuir com a implementação de planos de ação focalizados, visando aumentar os saberes acerca da temática, difundir a adoção de práticas em consonância à prevenção combinada e, consequentemente, incidir na diminuição da circulação do HIV nesse público.

## OBJETIVOS

Analisar os fatores associados ao conhecimento de universitários sobre o HIV e as profilaxias pré e pós-exposição.

## MÉTODOS

### Aspectos éticos

Obteve-se aprovação do Comitê de Ética em Pesquisa da Universidade Estadual de Maringá (UEM). Foram observados os aspectos éticos das Resoluções n.º 466/2012 e n.º 674/2022 do Conselho Nacional de Saúde, bem como do Ofício Circular n.º 002/2021. O consentimento livre e esclarecido foi obtido de todos os indivíduos envolvidos no estudo por meio eletrônico, selecionando-se a opção: “declaro que fui esclarecido e concordo em participar voluntariamente desta pesquisa”.

### Desenho, período e local do estudo

É um estudo transversal^(12)^, de caráter descritivo e analítico, cujo relatório se norteou pelas recomendações do *checklist Strengthening the Reporting of Observational Studies in Epidemiology* (STROBE). A pesquisa foi realizada de setembro a outubro de 2023 junto a estudantes da UEM, localizada na região noroeste do estado do Paraná, Brasil. O município de Maringá conta com uma população de 409.657 habitantes, segundo dados censitários de 2022^(13)^.

A UEM é uma universidade pública estadual, que está presente em todo o Paraná por meio de ações ligadas a projetos de ensino, pesquisa e extensão desenvolvidas pelo campus sede, em Maringá, e por seis campi regionais. Com cerca de 15.000 alunos, a UEM oferece mais de 70 cursos de graduação, distribuídos entre os diferentes campi, que abrangem diversas áreas do conhecimento, tais como: agrárias, biológicas, exatas, humanas, tecnológicas, saúde e sociais^(14)^.

### População ou amostra; critérios de inclusão e exclusão

A população deste estudo foi composta por todos os estudantes vinculados a cursos de graduação presenciais da UEM. Como critérios de inclusão, definiram-se: ter idade igual ou superior a 18 anos e estar devidamente matriculado no ano letivo de 2023. O único critério de exclusão foi: não responder ao questionário de coleta de dados por completo. No total, compuseram a população desta pesquisa 15.199 alunos, dos quais 14.672 atenderam aos critérios de inclusão.

Em se considerando a população elegível para este estudo, procedeu-se ao cálculo amostral definindo (i) a margem de erro de 5%, (ii) o nível de confiança de 95% e (iii) a distribuição heterogênea da população, resultando em uma amostra de 375 participantes. Tendo-se em vista a possibilidade de perdas e erros no processo de coleta de dados, acresceu-se o quantitativo de 10% para a definição do número mínimo de participantes, que correspondeu a 413 estudantes.

### Protocolo do estudo

Empregou-se um questionário estruturado e autoaplicável, elaborado pelos autores com base em estudos semelhantes^(11,15-19)^, com afirmativas para avaliar os conhecimentos sobre HIV, PrEP e PEP; haviam três opções (concordo; discordo; não sei dizer) para cada questão (Quadro 1). Ademais, aplicou-se um questionário de caracterização dos participantes com as variáveis de interesse listadas abaixo^(11,15-19)^; salienta-se que existia, para cada uma delas, um campo aberto para exceções:

**Quadro 1:** Questionário estruturado e autoaplicável da coleta de dados dos conhecimentos sobre a infecção pelo HIV e as profilaxias pré e pós-exposição, Maringá, Paraná, Brasil, 2023

Variável		Categoria
HIV	Q1: A única forma que posso me infectar pelo HIV é por meio de relações sexuais desprotegidas.	Concordo; discordo^*^; não sei dizer
	Q2: O uso da camisinha é a única medida capaz de impedir a infecção pelo HIV.	Concordo; discordo^*^; não sei dizer
	Q3: A infecção pelo HIV depende do sexo e/ou orientação sexual da pessoa.	Concordo; discordo^*^; não sei dizer
	Q4: A pessoa que tem HIV deixa de transmitir o vírus com o uso correto dos medicamentos antirretrovirais orais.	Concordo^*^; discordo; não sei dizer
PrEP	Q5: A PrEP consiste no uso de antirretrovirais orais, antes da exposição ao vírus, como estratégia de prevenção para diminuir o risco de infecção.	Concordo^*^; discordo; não sei dizer
	Q6: Posso tomar a PrEP a qualquer momento antes de uma relação sexual.	Concordo; discordo^*^; não sei dizer
	Q7: A PrEP está disponível para uso de toda a população sexualmente ativa, acima de 15 anos.	Concordo^*^; discordo; não sei dizer
	Q8: A pessoa que faz uso da PrEP também está protegida de outras infecções sexualmente transmissíveis, além do HIV.	Concordo; discordo^*^; não sei dizer
	Q9: A PrEP é oferecida gratuitamente no Sistema Único de Saúde.	Concordo^*^; discordo; não sei dizer
	Q10: Considerando-se que a pessoa em uso da PrEP não está doente, ela não precisa de acompanhamento pelos profissionais da saúde.	Concordo; discordo^*^; não sei dizer
PEP	Q11: A PEP consiste no uso de antirretrovirais orais, após a exposição ao vírus, como estratégia de prevenção para diminuir o risco de infecção.	Concordo^*^; discordo; não sei dizer
	Q12: A PEP é indicada para situações, como: acidentes com perfurocortantes, violência sexual etc.; ou sob demanda após percepção de risco.	Concordo^*^; discordo; não sei dizer
	Q13: A PEP deve ser tomada por 28 dias de forma ininterrupta.	Concordo^*^; discordo; não sei dizer
	Q14: Se a camisinha romper durante a minha relação sexual, eu posso solicitar o uso da PEP pelo Sistema Único de Saúde.	Concordo^*^; discordo; não sei dizer
	Q15: Quanto mais cedo a PEP for iniciada, maiores são as chances de impedir a infecção pelo HIV.	Concordo^*^; discordo; não sei dizer
	Q16: O prazo máximo para início da PEP é de 72 horas após a exposição ao HIV.	Concordo^*^; discordo; não sei dizer

HIV - vírus da imunodeficiência humana; PrEP - profilaxia pré-exposição; PEP - profilaxia pós-exposição;

* resposta correta.

**Tabela 1:** Medidas descritivas e análise bivariada de regressão com razões de prevalência brutas dos fatores associados ao conhecimento adequado dos universitários sobre a infecção pelo HIV e as profilaxias pré e pós-exposição, Maringá, Paraná, Brasil, 2023

Variável	n (%)	Prevalência	RPb (IC95%)	Valor de *p* ^*^
Gênero				< 0,01
Masculino	170 (33,80)	37,65	Referência	
Feminino	333 (66,20)	22,82	0,89 (0,83-0,95)	
Faixa etária				< 0,01
18 a 21 anos	239 (47,52)	20,92	Referência	
22 a 24 anos	156 (31,01)	29,49	1,07 (0,99-1,14)	
> 24 anos	108 (21,47)	40,74	1,16 (1,07-1,25)	
Orientação sexual				< 0,01
Heterossexual	300 (59,65)	20,33	Referência	
Homossexual	72 (14,31)	51,39	1,25 (1,15-1,37)	
Bissexual/pansexual/assexual	131 (26,04)	32,06	1,09 (1,02-1,17)	
Raça/cor				0,53
Parda	91 (18,09)	21,98	Referência	
Preta	29 (5,77)	31,03	1,07 (0,92-1,24)	
Branca	361 (71,77)	28,81	1,05 (0,97-1,14)	
Amarela	22 (4,37)	31,82	1,08 (0,91-1,27)	
Crença religiosa				0,02
Tem religião	336 (66,80)	24,70	Referência	
Não tem religião	167 (33,20)	34,13	1,07 (1,00-1,14)	
Relacionamento amoroso				0,77
Não	275 (54,67)	28,36	Referência	
Sim	228 (45,33)	27,19	0,99 (0,93-1,05)	
Renda familiar^†^				0,40
< 2 salários mínimos	110 (21,87)	27,27	Referência	
2 a 4 salários mínimos	179 (35,59)	31,28	1,03 (0,94-1,21)	
> 4 salários mínimos	214 (42,54)	25,23	0,98 (0,90-1,06)	
Área do conhecimento				< 0,01
Saúde	107 (21,27)	49,53	Referência	
Agrárias e biológicas	61 (12,13)	18,03	0,78 (0,71-0,87)	
Exatas e tecnologia	116 (23,06)	19,83	0,80 (0,73-0,87)	
Humanas e sociais	219 (43,54)	24,20	0,83 (0,76-0,89)	
Campus da instituição				< 0,01
Principal	447 (88,87)	29,75	Referência	
Outro	56 (11,13)	12,50	0,86 (0,79-0,94)	
Tempo na instituição				0,20
< 1 ano	98 (19,48)	23,47	Referência	
1 a 2 anos	182 (36,18)	28,02	1,03 (0,95-1,12)	
3 a 5 anos	199 (39,57)	27,64	1,03 (0,95-1,12)	
> 5 anos	24 (4,77)	45,83	1,18 (1,01-1,37)	
Relação sexual (últimos três meses)				< 0,01
Teve relação sexual	156 (31,01)	17,31	Referência	
Não teve relação sexual	347 (68,99)	32,56	1,13 (1,06-1,20)	
Parceria sexual (últimos três meses)				< 0,01
Parceria fixa	263 (52,29)	27,76	Referência	
Múltiplas parcerias	121 (24,06)	41,32	1,10 (1,02-1,19)	
Não teve parceria	119 (23,66)	14,29	0,89 (0,83-0,95)	
Fez teste rápido do HIV				< 0,01
Não	347 (68,99)	17,87	Referência	
Sim	156 (31,01)	50,00	1,27 (1,19-1,35)	
Ouviu falar sobre a PrEP				< 0,01
Não	270 (53,68)	4,81	Referência	
Sim	233 (46,32)	54,51	1,47 (1,40-1,54)	
Ouviu falar sobre a PEP				< 0,01
Não	249 (49,50)	5,62	Referência	
Sim	254 (50,50)	49,61	1,41 (1,34-1,48)	
Fez uso da PrEP				0,02
Não	483 (96,02)	26,92	Referência	
Sim	20 (3,98)	50,00	1,18 (1,01-1,37)	
Fez uso da PEP				< 0,01
Não	483 (96,02)	25,88	Referência	
Sim	20 (3,98)	75,00	1,39 (1,24-1,55)	
Sabe dizer o que é o HIV				< 0,01
Não	6 (1,19)	0,00	Referência	
Sim	497 (98,81)	28,17	1,28 (1,24-1,32)	
Sabe dizer o que é a PrEP				< 0,01
Não	277 (55,07)	3,61	Referência	
Sim	226 (44,93)	57,52	1,52 (1,45-1,59)	
Sabe dizer o que é a PEP				< 0,01
Não	250 (49,70)	3,20	Referência	
Sim	253 (50,30)	52,17	1,47 (1,40-1,54)	

RPb - razão de prevalência bruta; IC95% - intervalo de confiança de 95% (limite inferior-limite superior); HIV - vírus da imunodeficiência humana; PrEP - profilaxia pré-exposição; PEP - profilaxia pós-exposição;

* Valor de p obtido pelo teste de Wald; †salário mínimo igual a 1.320,00 reais.

- dados sociodemográficos: gênero (masculino; feminino), faixa etária (em anos: 18 a 21; 22 a 24; > 24), orientação sexual (heterossexual; homossexual; bissexual, pansexual e assexual), raça/cor (parda; preta; branca; amarela), religião (tem; não tem), renda familiar (em salários mínimos: < 2; 2 a 4; > 4) e relacionamento amoroso (não; sim);
- dados educacionais: área do conhecimento (saúde; agrárias e biológicas; exatas e tecnologias; humanas e sociais), campus de estudo (principal; outro) e tempo na instituição (em anos: < 1; 1 a 2; 3 a 5; > 5);
- dados comportamentais: relação sexual no último trimestre (teve; não teve), parceria sexual no último trimestre (fixa; múltipla; não teve) e histórico de realização de teste rápido para HIV em algum momento da vida (não; sim);
- dados complementares: ouviu falar sobre PrEP (não; sim), ouviu falar sobre PEP (não; sim), fez uso da PrEP (não; sim), fez uso da PEP (não; sim), sabe dizer o que é HIV (não; sim), sabe dizer o que é PrEP (não; sim) e sabe dizer o que é PEP (não; sim).

Esse instrumento foi adequado por juízes quanto à (i) linguagem empregada ao público-alvo, (ii) não indução de respostas, (iii) abrangência do conteúdo, (iv) existência de única interpretação e (v) quantidade de questões^(20)^. Para tanto, consideraram-se dois profissionais de serviços de atendimento especializado (SAE); dois consultores-técnicos do Ministério da Saúde vinculados ao programa de HIV; e uma docente do curso de enfermagem da UEM.

Ressalta-se que o questionário (Quadro 1) foi elaborado pelos pesquisadores deste estudo, a partir de discussões no âmbito do Grupo de Estudos e Pesquisas em Vigilância do HIV/aids e da Tuberculose (GEPVHAT/UEM), junto a estudantes da graduação em enfermagem e da pós-graduação - em nível de mestrado e doutorado. Todavia, os juízes foram essenciais no processo de adequação do instrumento, gerando a versão final apresentada nesta pesquisa.

Os dados foram coletados pelo *Google Forms* ^®^ e, a fim de evitar a duplicidade, o formulário foi restrito a um único preenchimento por e-mail. Enviou-se um convite diretamente aos participantes via *Gmail* ^®^, utilizando-se a opção “cópia oculta” para garantir o sigilo. No *link* para o formulário, os universitários tiveram acesso ao termo de consentimento livre e esclarecido. No decorrer da coleta, foram compiladas 503 respostas; não houve exclusões devido a incompletudes do formulário.

### Análise dos resultados e estatística

Procedeu-se à análise descritiva por meio de média aritmética e frequências absolutas (n) e relativas (%) dos dados de caracterização e do questionário. As questões com respostas incorretas/incertas e corretas receberam, respectivamente, os escores zero e um, gerando uma pontuação de zero a 16. Com base em estudos^(11,18-19)^, o conhecimento foi categorizado pelos percentis em: inadequado (≤ 12 questões ou ≤ 75% de acerto) e adequado (> 12 questões ou > 75% de acerto).

A partir disso, calculou-se a prevalência de conhecimento adequado sobre HIV, PrEP e PEP entre os estudantes universitários, inclusive para cada categoria das variáveis independentes. O cálculo se deu pela divisão do número de pessoas que obtiveram 13 respostas corretas ou mais no questionário, pelo total de respondentes na respectiva questão e categoria da variável, e o resultado desta divisão foi multiplicado por 100.

Para identificar os fatores associados ao conhecimento, utilizou-se o modelo de regressão de Poisson com variância robusta^(21)^, no qual a variável dependente foi a categoria do conhecimento (inadequado; adequado) e os dados de caracterização foram as variáveis independentes. Inicialmente, conduziu-se análise bivariada (bruta) com cada variável e, para controlar eventuais fatores de confusão, realizou-se análise multivariada (ajustada) pelo método *stepwise backward* ^(22)^.

Antes de proceder aos modelos múltiplos, a multicolineariedade foi avaliada e descartada pelo fator de inflação da variância e pelo teste de tolerância^(22)^. Conjuntamente, as variáveis com p-valor ≤ 0,20 no teste de Wald em análise bivariada foram inseridas e, conforme significância, removidas uma a uma, até restarem aquelas com p-valor ≤ 0,05. Como medida de associação nos modelos, calcularam-se as razões de prevalência (RP) e seus intervalos de confiança de 95% (IC95%).

As RP representaram a probabilidade de uma categoria de resposta da variável independente influenciar, para mais ou menos, no conhecimento adequado - ou seja, mais que 12 acertos - dos estudantes, em relação à categoria de referência na mesma variável. Para o modelo final, avaliou-se a qualidade do ajuste pelo teste qui-quadrado da razão de verossimilhança (p-valor ≤ 0,05). Todas as análises foram realizadas no *software SPSS Statistics* ^®^, em sua versão 25.

## RESULTADOS

Ao analisar a porcentagem média das questões de cada eixo temático do questionário, percebeu-se que a maioria dos acertos foi relacionado ao HIV (77,63%), seguido pelos acertos sobre a PEP (51,92%) e, finalmente, sobre a PrEP (44,07%) (Figura 1). As respostas para cada questão do questionário foram apresentadas na Figura 2; as alternativas com o menor quantitativo de acertos foram: Q6 (n = 166; 33,00%), Q7 (n = 129; 25,65%), Q13 (n = 166; 33,00%) e Q16 (n = 168; 33,40%).

**Figura 1:** Distribuição das respostas dos universitários para as questões, segundo temas relacionados à infecção pelo HIV e às profilaxias pré e pós-exposição, Maringá, Paraná, Brasil, 2023 PEP - profilaxia pós-exposição; PrEP - profilaxia pré-exposição; HIV - vírus da imunodeficiência humana.

**Figura 2:** Distribuição das respostas dos universitários para as questões relacionadas à infecção pelo HIV e às profilaxias pré e pós-exposição, Maringá, Paraná, Brasil, 2023

As características dos 503 participantes foram apresentadas na Tabela 1. A prevalência de conhecimento adequado entre os participantes sobre a infecção pelo HIV, a PrEP e a PEP foi de 27,83%. Houve maior prevalência de acertos (mais que 12) no questionário entre pessoas do gênero masculino, com idade superior a 24 anos, de orientação homossexual, de raça/cor amarela e preta, sem crença religiosa e que não possuíam algum parceiro (Tabela 1).

A prevalência de conhecimento adequado também foi maior em pessoas com renda familiar de 2 a 4 salários mínimos, de cursos da saúde, do campus principal, com mais de 5 anos na instituição, entre aquelas que não tiveram relação sexual no último trimestre e aquelas com múltiplas parcerias. O histórico de teste rápido para HIV e já ter ouvido falar, saber o que é e ter feito uso da PrEP e da PEP também colaboraram para a maior prevalência de conhecimento adequado (Tabela 1).

No modelo final, jovens com idade superior a 24 anos mostraram maior conhecimento adequado (RP ajustada - RPa = 1,08; IC95% 1,02-1,15) em relação àqueles de 18 a 21 anos. Os estudantes da saúde tiveram mais acertos quando comparados aos de agrárias/biológicas (RPa = 0,90; IC95% 0,83-0,98) e de humanas/sociais (RPa = 0,93; IC95% 0,87-0,99). Os jovens que não tiveram relação sexual também apresentaram maior conhecimento (RPa = 1,07; IC95% 1,02-1,13) (Tabela 2).

**Tabela 2:** Análise multivariada de regressão com razões de prevalência ajustadas dos fatores associados ao conhecimento adequado dos universitários sobre a infecção pelo HIV e as profilaxias pré e pós-exposição, Maringá, Paraná, Brasil, 2023

Variável	RPa (IC95%)	Valor de *p* ^*^
Faixa etária		0,02
18 a 21 anos	Referência	
22 a 24 anos	1,01 (0,96-1,07)	
> 24 anos	1,08 (1,02-1,15)	
Área do conhecimento		0,04
Saúde	Referência	
Agrárias e biológicas	0,90 (0,83-0,98)	
Exatas e tecnologia	0,93 (0,86-1,00)	
Humanas e sociais	0,93 (0,87-0,99)	
Relação sexual (últimos três meses)		< 0,01
Teve relação sexual	Referência	
Não teve relação sexual	1,07 (1,02-1,13)	
Fez teste rápido do HIV		< 0,01
Não	Referência	
Sim	1,09 (1,03-1,15)	
Ouviu falar sobre a PrEP		< 0,01
Não	Referência	
Sim	1,11 (1,03-1,20)	
Sabe dizer o que é a PrEP		< 0,01
Não	Referência	
Sim	1,18 (1,08-1,30)	
Sabe dizer o que é a PEP		< 0,01
Não	Referência	
Sim	1,14 (1,07-1,22)	

RPa - razão de prevalência ajustada; IC95% - intervalo de confiança de 95% (limite inferior-limite superior); HIV: vírus da imunodeficiência humana; PrEP: profilaxia pré-exposição; PEP: profilaxia pós-exposição;

* Valor de p obtido pelo teste de Wald.

Os universitários que já fizeram o teste rápido para HIV tiveram maior prevalência de conhecimento (RPa = 1,09; IC95% 1,03-1,15) em detrimento dos que nunca o fizeram. Os jovens que tiveram a percepção de que sabiam o que era a PrEP (RPa = 1,18; IC95% 1,08-1,30) e a PEP (RPa = 1,14; IC95% 1,07-1,22), e aqueles que já tinham ouvido falar sobre a PrEP (RPa = 1,11; IC95% 1,03-1,20) também apresentaram maior probabilidade de possuir conhecimento adequado (Tabela 2).

## DISCUSSÃO

Este estudo transversal com mais de 500 universitários demonstrou baixa prevalência de conhecimento adequado sobre o HIV e suas profilaxias. Os saberes variaram segundo as características sociodemográficas, educacionais e comportamentais dos estudantes, sendo que aqueles com maior idade, de cursos da área da saúde, sem vida sexual ativa nos últimos três meses, que já tinham feito teste rápido para HIV e sabiam dizer o que era PrEP e PEP apresentaram maiores acertos.

O conhecimento incipiente acerca dessa temática já foi relatado na literatura. No contexto nacional, estudo transversal em uma universidade baiana verificou que apenas 28,5% dos estudantes tinham conhecimento da PEP^(15)^. Internacionalmente, em uma instituição rural na África do Sul, 42,0% dos estudantes apresentaram conhecimentos adequados sobre o HIV^(16)^; na Tailândia, pesquisa indicou que apenas 20,8% dos universitários já tinham ouvido falar sobre a PrEP^(17)^.

Esse cenário sinaliza um alerta para a possível educação limitada na educação superior, e até mesmo anterior a ela, no que concerne ao HIV e suas prevenções. Em que pese a inserção de políticas preventivas no rol de estratégias do SUS - com destaque para os preservativos internos e externos e as profilaxias do HIV^(23)^ -, é impreterível a expansão das campanhas de divulgação dessas tecnologias junto aos públicos-alvo, iniciando-se em seus ambientes escolares e universitários.

Isso pode se tornar ainda mais alarmante ao considerar que, neste estudo, a maioria dos jovens teve acertos relacionados majoritariamente ao HIV, e não sobre suas medidas preventivas. Os participantes não reconheceram a idade mínima para uso da PrEP e não souberam informar em qual momento os medicamentos poderiam ser ingeridos. E quanto à PEP, eles não conheciam a duração do uso e o prazo adequado para início das medicações após uma situação de risco.

Dentre os fatores associados, os universitários com idade maior que 24 anos tiveram mais acertos quando comparados aos de 18 a 21 anos. Tal dado pode se relacionar ao maior tempo na universidade, que constitui uma atmosfera para discussão sobre o tema^(10)^. Todavia, o interesse e o conhecimento sobre o HIV e suas formas de prevenção podem estar mais relacionados a componentes comportamentais e socioculturais, do que propriamente aos anos de vida.

Historicamente, a inserção de disciplinas sobre as doenças transmissíveis acompanhou o contexto político e ganhou maior destaque com o advento da epidemia do HIV^(24)^. Nesse cenário, as graduações na área da saúde têm se aproximado das políticas públicas relativas ao HIV^(24)^. Essa situação poderia justificar a maior probabilidade de estudantes pertencentes a cursos da área da saúde apresentarem conhecimento sobre essa temática superior a universitários de outras áreas^(15)^.

Um resultado curioso foi que jovens que não se relacionaram sexualmente nos últimos três meses tiveram melhor conhecimento no questionário. Contrariamente, estudo com HSH notou que ter relações sexuais com várias parcerias, em comparação à fixa ou inexistente, esteve associado ao melhor conhecimento sobre a PrEP^(25)^. Isso poderia se justificar pela maior difusão de informações junto a esse público ou pela maior preocupação quanto à prevenção devido à exposição recorrente.

Nesta pesquisa, presume-se que os universitários - que, em tese, dispõem de maior acesso à informação em detrimento da população geral, sobretudo aqueles em cursos da saúde^(26)^ - buscaram aprimorar seus conhecimentos sobre os métodos preventivos antes de ter relações sexuais. No entanto, embora o sigilo tenha sido garantido, não se pode descartar a possibilidade de um viés de informação frente ao constrangimento em confirmar o início da vida sexual aos pesquisadores.

O histórico de realização de testagem rápida para HIV também esteve associado ao maior número de acertos. Estudo português identificou que pessoas que fizeram o teste para HIV tiveram uma razão de chances de 3,68 vezes (IC95% 3,11-4,36) na obtenção de conhecimento sobre a PrEP^(27)^. Acredita-se que a aproximação com os serviços de testagem e aconselhamento pode favorecer o acesso a informações sobre o HIV e as estratégias de prevenção vigentes.

No Brasil, os centros de testagem e aconselhamento (CTA) preconizam, desde sua criação, a oferta de testes acompanhada por ações de educação em saúde sobre as IST e suas formas de prevenção^(28)^. Sendo assim, presume-se a efetividade dessa política pública nacional por meio deste estudo, indicando a importância de ampliá-la e fortalecê-la em todos os serviços da rede de atenção, com intuito de favorecer a divulgação da informação de forma oportuna e constante às pessoas.

Vale pontuar, ainda, que na instituição de ensino em questão, além da disponibilidade de um ambulatório com equipe médica e de enfermagem, têm sido realizadas campanhas educativas em parceria com o CTA do município. Essas estratégias podem focalizar a difusão de orientações sobre os métodos preventivos às IST e a expansão da oferta e do acesso aos testes rápidos, o que pode ter influído no maior conhecimento dos estudantes que já realizaram a testagem.

A percepção referida de conhecer a PrEP e a PEP também elevou a probabilidade de obter mais acertos no questionário. Esse achado, mesmo que consoante à literatura^(25,27)^, acende um alerta sinalizado em estudo prévio, no qual 91,1% dos participantes já tinham ouvido falar em PrEP; todavia, apenas 7,5% relataram que haviam feito o uso, embora o interesse em utilizá-la - desde que mais bem informados - estivesse presente em 52,1% da amostra^(25)^.

Nesse sentido, mais do que dialogar sobre as estratégias disponíveis para o enfrentamento do HIV, é preciso (re)pensar os comportamentos para oportunizar a escolha individual orientada para o uso da PrEP e da PEP. Isso implica, inclusive, na sensibilização e capacitação profissional, visto que, a despeito do potencial contributivo das orientações educativas concomitantes a exames e consultas, a atuação dos CTA tem sido inadequada em determinados locais do país^(29)^.

Além disso, deve-se levar em consideração que as ações a serem desenvolvidas com os estudantes precisam considerar a representação social das IST nesse grupo. Os jovens reconhecem as práticas sexuais desprotegidas como fatores de risco e percebem a necessidade de uso do preservativo como método de prevenção às infecções; no entanto, o medo e o estigma estão involucrados em seu imaginário, dificultando a adoção de outras práticas preventivas vigentes^(30)^.

Nesse contexto, incentiva-se a articulação da academia junto aos profissionais envolvidos na assistência, na gestão e na vigilância, com foco na promoção de campanhas que visem facilitar o acesso ao conhecimento no meio universitário. Enfatiza-se, ainda, a necessidade de revisão da atuação interna da universidade como educadora, visto que as ações precisam ser expandidas para abarcar estudantes de outras áreas, especialmente aqueles não inseridos em cursos da saúde.

### Limitações do estudo

É mister pontuar algumas limitações, além daquelas inerentes ao desenho transversal: (i) a restrição da amostra a um único local, (ii) o agrupamento dos estudantes de diferentes áreas nas sub-variáveis, (iii) a coleta por meio de questionário autoaplicável e on-line, (iv) o viés de informação nas respostas, (v) a não validação do instrumento de avaliação dos conhecimentos e (vi) a junção das questões sobre diferentes temas (HIV, PrEP e PEP) em uma variável dependente.

### Contribuições para a área da enfermagem, saúde ou política pública

Os achados apresentam grande relevância para a saúde pública e a educação, servindo de alerta a professores, gestores e profissionais de saúde no que se refere à necessidade de melhoria dos programas educativos e de conscientização no meio universitário, bem como de inclusão da temática na grade curricular obrigatória de todos os cursos. Isso é crucial para ampliar o conhecimento e promover maior acesso e adesão às ações preventivas do HIV nesse grupo.

Nesse sentido, esta pesquisa se destaca ao colocar em pauta uma temática de extrema relevância para adolescentes e jovens, tendo em vista a ascensão dos casos de HIV nessa população. Acredita-se que estratégias que visem à apropriação dos universitários sobre os métodos e as tecnologias da prevenção combinada constituem uma etapa indispensável para acelerar a resposta de enfrentamento do HIV, com foco no alcance das metas ambiciosas dos ODS até 2030.

## CONCLUSÕES

O conhecimento de jovens sobre HIV, PrEP e PEP foi, em geral, inadequado, o que requer a necessidade de promoção de educação na temática junto a esse grupo vulnerável. Observou-se que determinadas características influenciaram no conhecimento, sendo que jovens com maior idade, de cursos da saúde, sem vida sexual ativa nos últimos três meses, que já tinham feito teste rápido para HIV e sabiam dizer o que era PrEP e PEP apresentaram maiores escores.

Diante disso, é premente o desenvolvimento de estratégias educativas que almejem a conscientização da temática na universidade, que constitui um importante espaço de troca de saberes e práticas entre os pares. Para tanto, deve-se considerar as particularidades imbricadas ao contexto da juventude, a fim de que as estratégias sejam capazes de abarcar tabus e construções socioculturais que podem influenciar hábitos e comportamentos, em especial daqueles em maior vulnerabilidade.

## Data Availability

https://doi.org/10.17632/7jmv72wjxm.1
